# Proteolysis Targeting Chimera (PROTAC) for Macrophage Migration Inhibitory Factor (MIF) Has Anti‐Proliferative Activity in Lung Cancer Cells

**DOI:** 10.1002/anie.202101864

**Published:** 2021-06-26

**Authors:** Zhangping Xiao, Shanshan Song, Deng Chen, Ronald van Merkerk, Petra E. van der Wouden, Robbert H. Cool, Wim J. Quax, Gerrit J. Poelarends, Barbro N. Melgert, Frank J. Dekker

**Affiliations:** ^1^ Department Chemical and Pharmaceutical Biology Groningen Research Institute of Pharmacy (GRIP) University of Groningen Antonius Deusinglaan 1 9713 AV Groningen The Netherlands; ^2^ Molecular Pharmacology Groningen Research Institute of Pharmacy (GRIP) University of Groningen Antonius Deusinglaan 1 9713 AV Groningen The Netherlands; ^3^ University Medical Center Groningen Groningen Research Institute of Asthma and COPD University of Groningen Hanzeplein 1 9713 GZ Groningen The Netherlands

**Keywords:** A549 cells, cell cycle arrest, Macrophage migration inhibitory factor (MIF), Mitogen-activated protein kinase (MAPK) pathway, proteolysis targeting chimera (PROTACs)

## Abstract

Macrophage migration inhibitory factor (MIF) is involved in protein‐protein interactions that play key roles in inflammation and cancer. Current strategies to develop small molecule modulators of MIF functions are mainly restricted to the MIF tautomerase active site. Here, we use this site to develop proteolysis targeting chimera (PROTAC) in order to eliminate MIF from its protein‐protein interaction network. We report the first potent MIF‐directed PROTAC, denoted **MD13**, which induced almost complete MIF degradation at low micromolar concentrations with a DC_50_ around 100 nM in A549 cells. **MD13** suppresses the proliferation of A549 cells, which can be explained by deactivation of the MAPK pathway and subsequent induction of cell cycle arrest at the G2/M phase. **MD13** also exhibits antiproliferative effect in a 3D tumor spheroid model. In conclusion, we describe the first MIF‐directed PROTAC (**MD13**) as a research tool, which also demonstrates the potential of PROTACs in cancer therapy.

## Graphical abstract

Targeting macrophage migration inhibitory factor (MIF) is challenging, as it is involved in diseases via protein‐protein interactions. Here, the first potent MIF‐directed PROTAC **MD13** is reported, which eliminates MIF from its protein‐protein interaction network by inducing MIF degradation. **MD13** suppresses the proliferation of A549 cells by reducing MIF protein levels and MIF‐related signaling, demonstrating the potential of PROTACs in cancer therapy.

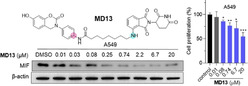

## Introduction

Cancer treatment has improved enormously over the past decades, but unfortunately cancer remains one of the leading health problems worldwide. Two important reasons that limit the success of cancer treatments are heterogeneity of the tumor and acquired therapy resistance.^[1]^ To address these problems, it is imperative to discover and exploit previously unrecognized molecular mechanisms that are involved in cell proliferation. The protein macrophage migration inhibitory factor (MIF) has been implicated in the pathogenesis of cancers.^[2]^ Overexpression of MIF has been detected in cancer types such as genitourinary cancer,^[3]^ melanoma,^[4]^ neuroblastoma,^[5]^ and lung carcinoma.^[6]^ Remarkably, down‐regulation of MIF expression by gene‐knockout^[7]^ or gene‐knockdown[^8^, ^9^] not only reduced tumor progression and metastases, but also induced antitumor immune responses.^[10]^ These results indicate that targeting MIF could be a promising strategy towards development of novel cancer therapeutics.

MIF exists as a homotrimer in which each monomer consists of a 114‐amino acid peptide.^[11]^ Initial evidence indicated an important role for MIF in inflammation and immune responses. Subsequently, MIF was also discovered to function as a hormone,^[12]^ a chemokine^[13]^ and as a molecular chaperone.^[14]^ MIF exerts its functions mainly through protein‐protein interactions with membrane‐bound receptors or intracellular signaling proteins. One of those receptors is cluster of differentiation 74 (CD74), which is the cognate receptor for MIF.[^15^, ^16^] The interaction between MIF and CD74 triggers activation of the mitogen‐activated protein kinase (MAPK) pathway and inhibits the p53 pathway, which results in cell growth.^[3]^ In addition, other non‐cognate binding partners such as CXCR4 also play key roles in cancer development.[^17^, ^18^] Therefore, the discovery of reagents that interfere with the interaction between MIF and CD74 or other binding partners is an attractive strategy to inhibit MIF‐induced cellular signaling in relevant disease models.

**Figure 1:**
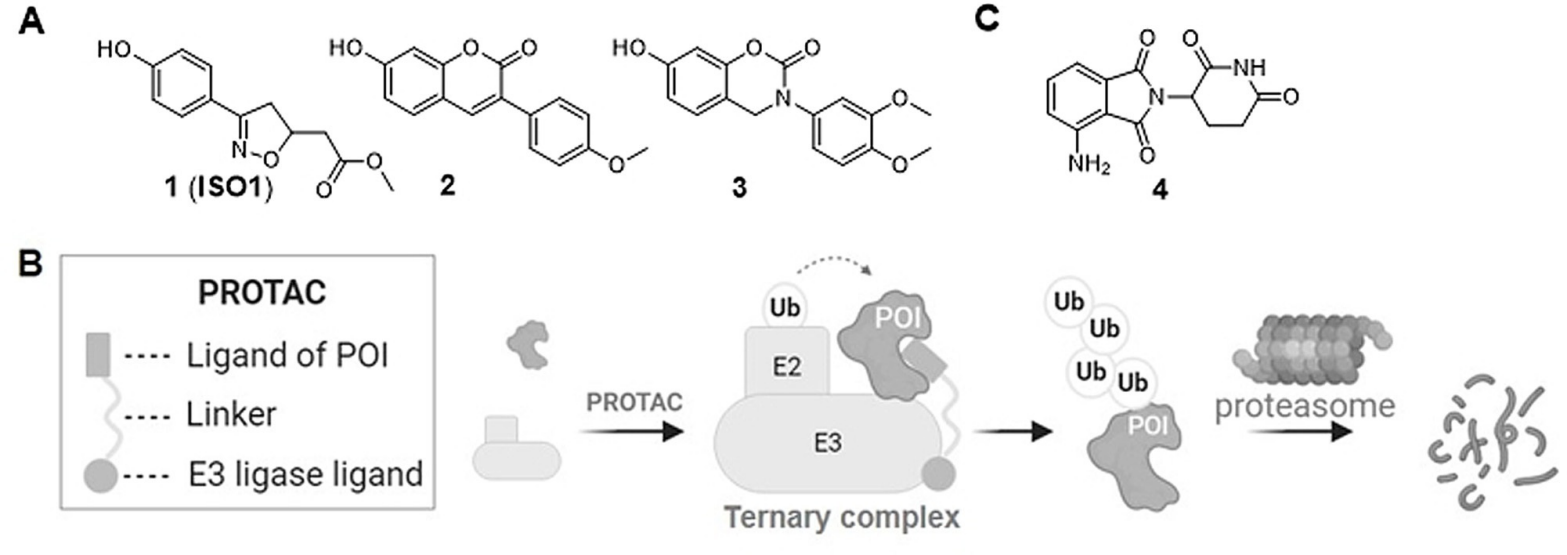
Small molecule inhibitors of MIF tautomerase activity and general mechanistic representation of PROTAC action. A) Structure of representative MIF tautomerase inhibitors **ISO 1**,^[40]^ **2** and **3**.^[27]^ B) Mechanism representation of the action of PROTACs. C) Chemical structure of pomalidomide **4**. POI: protein of interest. Ub: ubiquitin.

Apart from its function as a cytokine, MIF also harbors enzymatic activity to catalyze keto‐enol tautomerization of substrates such as D‐dopachrome and 4‐hydroxylphenylpyruvate (4‐HPP).^[19]^ MIF exerts the tautomerase activity through its proline‐1, which is a nucleophile.^[20]^ So far, the physiological function of the enzymatic activity remains elusive. Interestingly, some key amino acid residues in close proximity to the tautomerase active site are involved in binding to CD74 and CXCR4.[^21^, ^22^, ^23^] This implies that small molecule inhibitors of MIF tautomerase activity are able to interfere with the MIF‐receptor interactions. Based on this idea, several series of small‐molecule inhibitors for MIF tautomerase activity have been developed.[^24^, ^25^] One of the earliest discovered MIF inhibitors is **ISO 1** (Figure 1 A), which gained wide use as a reference compound in MIF research.^[26]^ However, the binding potency of **ISO 1** for MIF is only in the micromolar concentration range. Research over the past two decades yielded several MIF inhibitors with nanomolar potency. Previously, our group and others reported structure–activity relationships (SARs) for 7‐hydroxycoumarin derivatives based on inhibitor **2** (Figure 1 A).[^27^, ^28^] We also found that compounds containing a 7‐hydroxy‐3,4‐dihydrobenzoxazin‐2‐ones backbone such as **3** can also provide potent inhibition against MIF.[^27^, ^29^] Furthermore, the Jorgensen lab discovered potent MIF inhibitors that contain a biaryltriazole or pyrazole scaffold.[^30^, ^31^] However, the potency to inhibit MIF tautomerase activity does not always correlate well with the potency to inhibit the MIF‐CD74 interaction or MIF induced signaling in cell‐based studies.[^32^, ^33^] Altogether, this suggests that development of molecules that merely bind to the tautomerase enzyme active site may not be enough to effectively interfere with MIF protein‐protein interactions. In addition, proteasome‐dependent MIF degradation induced by HSP90 suppression proved to be correlated with the inhibition of MIF activity on cell proliferation.^[34]^ Therefore, we seek to use proteolysis‐targeting chimeras (PROTACs) as an alternative strategy to attenuate MIF functions by depletion of MIF protein.

The PROTAC strategy has emerged as a novel concept in small‐molecule drug discovery. This strategy employs a heterobifunctional molecule that binds both the protein of interest and an E3 ubiquitin ligase to form a ternary complex (Figure 1 B).^[35]^ This enables hijacking the E3 ubiquitin ligase activity to ubiquitinate the protein of interest that is subsequently degraded by the ubiquitin‐proteasome system. After degradation of the protein of interest the PROTAC can be recycled for a new round of targeted degradation of the protein of interest, thus providing a catalytic cycle. Importantly, the PROTAC strategy enables downregulation of intercellular protein levels of the protein of interest rather than just blocking one of its respective catalytic activities or interaction surfaces. After its development by the groups of Crews and Deshaies,^[36]^ the PROTAC strategy has progressed enormously by virtue of the identification of potent and selective E3 ligase ligands such as pomalidomide **4** (Figure 1 C).^[37]^ Over the past years, an increasing number of proteins have been targeted by PROTACs, including kinases, epigenetic editors, bromodomains, nuclear receptors and others.^[38]^ However, PROTAC development has been largely limited to clinically validated targets for which marketed drugs are available. The next step to unleash the full potential of PROTAC development is targeting the traditionally undruggable proteome, for example, proteins involved in protein‐protein interactions.^[39]^

In the present study, we report the first MIF‐directed PROTACs by linking potent MIF binding molecules to pomalidomide as a ligand for the cereblon Cullin RING E3 ubiquitin ligase complex. Through investigation of the structure–activity relationship, we discovered a potent PROTAC MIF degrader (**MD13**) with a DC_50_<100 nM and a D_max_>90 % in A549 cells. Control experiments were performed to demonstrate that **MD13** reduced the MIF level via cereblon ligand‐induced degradation. Moreover, **MD13** inhibited the growth of cancer cells in a 2D and a 3D cell culture systems. Altogether, development of these MIF degraders indicates a new strategy for treatment of cancers and also provides a new class of tools to study MIF.

## Results and Discussion

### PROTAC Design and Synthesis

**Figure 2:**
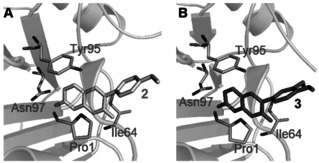
Design of MIF targeting PROTACs. Optimal binding poses of MIF with **2** (A, PDB 1GCZ)^[28]^ and **3** (B, PDB 5HVT).^[29]^ MIF is shown as a pale‐green cartoon and the key residues forming the binding pocket are represented as sticks. Docking studies were performed with Discovery Studio and models were prepared with Pymol.

Compound **2** and **3** (Figure 1 A) are inhibitors of MIF tautomerase activity with nanomolar potency. Based on the known pharmacophoric features of MIF tautomerase inhibitors derived from crystal structures[^28^, ^29^] and docking studies (Figure 2), we presume that the aromatic hydroxyl functionality is deeply embedded in the tautomerase active site, where it is involved in two key hydrogen‐bonding interactions with Asn97. Consequently, the methoxyphenyl functionality in **2** and the ortho‐dimethoxyphenyl functionality in **3** protrude out of the pocket and are solvent‐exposed. Therefore, we replaced the methoxy‐functionalities by an amine to enable attachment of a linker by an amidation reaction. Both MIF tautomerase inhibitor **2** and **3** were used as MIF binding ligand in PROTACs for MIF degradation. Compound **2** was linked to the E3 ligase ligand pomalidomide to provide PROTACs indicated as group 1 and compound **3** was linked to pomalidomide to provide PROTACs indicated as group 2 (Table S2).

### PROTACs Retain Their Inhibitory Potency for MIF Tautomerase Activity

To verify binding of the resulting PROTACs to MIF, we measured their ability to inhibit MIF tautomerase activity. The candidate PROTACs inhibited MIF enzymatic activity with nanomolar inhibition constants (*K*_i_). The group 1 PROTACs that contain a 7‐hydroxycoumarin MIF binding core provided *K*_i_ values between 117 to 999 nM, which is in the same range as the *K*_i_ of MIF inhibitor **2** that was reported to be 370 nM (Table S2).^[27]^ Interestingly, the PROTACs **MD1**–**4** with one carbon atom between the triazole and the amide functionality are more potent MIF tautomerase inhibitors compared to PROTACs with two (**MD5**) or three carbon atoms (**MD6**) in this position. The *K_i_* values for the PROTACs of group 2 with a 7‐hydroxy‐3,4‐dihydrobenzoxazin‐2‐one MIF binding core were all around 100 nM. This is very well in line with the potency of their parent inhibitor **3**, which has a *K*_i_ value of 150 nM.^[27]^ The results demonstrated that our design strategy for linkers did not or minimally perturb target engagement.

### PROTACs Induce MIF Degradation

Previous studies have demonstrated that A549 cells express a high‐level of MIF.^[41]^ In addition, A549 cells have been successfully used for assessing activity of cereblon ligand‐based PROTACs previously.^[42]^ Therefore, A549 is a suitable cell line for evaluating the effect of our putative MIF‐directed PROTACs **MD1** to **MD12** on MIF protein levels. The reduction in MIF levels were monitored in A549 cells that were treated with two different PROTAC concentrations (20 and 2 μM) for 12 h in order to estimate the dose dependency, which is important for PROTACs because of the Hook effect.^[35]^ The MIF levels in cell lysates were analyzed using an enzyme‐linked immunosorbent assay (ELISA).

Treatment with 20 μM of any of the putative MIF PROTACs resulted in lower MIF protein levels in A549 cells compared to vehicle‐treated controls (Table S2 and Figure S1). The only exception is **MD6** that did not trigger MIF reduction. The series of PROTACs with **2** as warhead showed increasing potency with increasing linker length to reach more than 50 % reduction in the MIF protein levels upon treatment with 20 μM **MD4** and **MD5**. However, at a concentration of 2 μM no significant degradation of MIF was observed for this series of compounds.

In the new series of PROTACs using **3** as MIF binding ligand, treatment with 20 μM of either of the three compounds with aliphatic linkers (**MD7**–**9**) resulted in more than 50 % lower MIF‐protein levels compared to control, whereas this was only observed for **MD10** for the series of compounds with a triazole in the linker (Table 1 and Table S2). Subsequently, the potency of the PROTACs at 2 μM was investigated, which demonstrated the highest potency for **MD9** and **MD10**. Based on these data **MD9** was selected as the most promising starting point to develop MIF‐directed PROTACs further.

**Table 1:** Optimization of linker length of **MD9** and control compound with impaired cereblon‐binding ligand. 
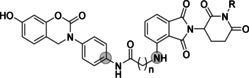

ID	*n*	R	*K*_i_ [nM]^[a]^	degradation [%]^[b]^	
				2 μM	0.2 μM
**3**	–	–	103±10	–	–
**MD7**	3	H	55±3	n.s.	–
**MD8**	4	H	86±5	21±7	–
**MD9**	6	H	51±3	55±4	24±4
**MD13**	7	H	71±5	91±5	71±7
**MD14**	9	H	65±4	64±2	51±2
**MD15**	7	CH_3_	55±12	n.s.	n.s.

[a] Measured by MIF catalyzed 4‐HPP tautomerization assay using the method as previously reported by our group (*n*=3). [b] Degradation percentage is represented as mean±SD (*n*=3). Not significant (n.s.) *P*>0.05.

The cellular effect of **MD9** as a PROTAC was further investigated. In comparison to the vehicle control, **MD9** reduced the MIF levels in a dose‐dependent manner to provide a maximal degradation of more than 90 % with a half‐maximal degradation concentration (DC_50_) around 1.5 μM measured by both ELISA and western‐blot (Figure S2). The action mode of **MD9** induced MIF degradation is investigated. MIF degradation becomes visible after 3 hours of treatment and reached its maximum effect after 6 and 9 hours with 10 μM **MD9** treatment. The degradation can be rescued with pretreatment of **1**, **3**, **4** or proteasome inhibitor Bortezomib,^[44]^ which indicates that the action of **MD9** depends on MIF binding as well as on CRBN E3 ligase binding, which triggers proteasome‐mediated degradation (Figure S3).

### Development of a MIF‐Directed PROTAC with Improved Potency

Although **MD9** was identified as an effective MIF‐directed PROTAC, its potency remains limited to the micromolar concentration range. The structure–activity relationship (SAR) analysis of the PROTAC linkers of the second group suggests that a longer aliphatic linker (**MD7**–**9**) is more favorable for MIF degradation. To further explore the SARs and to improve the efficacy of MIF PROTACs, we designed and synthesized **MD13** and **MD14**, which contain longer linkers than **MD9**. Both new PROTACs showed MIF binding constants (*K*_i_) in a range similar to the parental ligand **3** (Table 1). Subsequently, the reduction of MIF levels upon treatment with **MD13** and **MD14** at concentration of 2 and 0.2 μM was investigated. **MD13** treatment resulted in 91 % and 71 % lower MIF protein levels at 2 and 0.2 μM, respectively. This indicates that **MD13** is the most potent MIF PROTAC in this series. In line with current knowledge, the length of the linker appears to be critical for the potency of MIF‐directed PROTACs and the linker length of **MD13** seems to be optimal.

To further confirm the action of **MD13** as a MIF‐degrading PROTAC, we synthesized a control compound for **MD13** containing a CRBN ligand with impaired CRBN binding. The imide nitrogen of the piperidine‐2,6‐dione functionality in the CRBN ligand is involved in a crucial hydrogen bond with CRBN.^[45]^ Methylation of this imide nitrogen will abolish CRBN binding.^[46]^ We synthesized control compound **MD15** with a methylated pomalidomide as CRBN ligand. **MD15** preserved the MIF binding potency with a *K*_i_ of 55 nM (Table 1). However, **MD15** was not capable of inducing MIF degradation at both 2 and 0.2 μM, whereas **MD13** was. Collectively, this result confirms that **MD13** induced MIF degradation through binding to E3 ligase cereblon.

### Characterization of MD13 as a MIF‐Directed PROTAC

Since PROTACs are relatively large heterobifunctional molecules, the efficacy of these compounds may be limited by poor cell permeability.^[47]^ In order to estimate the cellular uptake of PROTAC **MD13**, the intrinsic fluorescence properties of the pomalidomide part of **MD13** were employed for visualization of its subcellular localization. Clear localization of **MD13** in the cytoplasm of A549 cells was observed after one‐hour incubation (Figure S5). In parallel, the subcellular localization of MIF and its decrease in situ upon **MD13** treatment was visualized by confocal fluorescence microscopy using a fluorescent secondary antibody. Treatment with 1 μM **MD13** significantly depleted MIF in A549 cells (Figure S5). Taken together, microscopic analysis revealed that **MD13** can enter cells to effectively induce MIF degradation.

**Figure 3:**
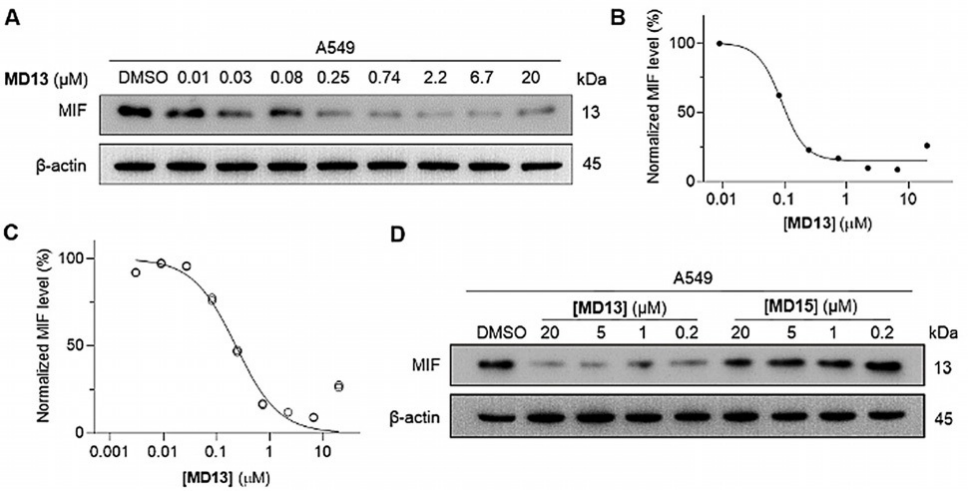
The MIF‐directed PROTAC **MD13**, but not **MD15**, causes depletion of MIF protein in A549 cells. A) A549 cells were treated with the indicated concentrations of **MD13** for 12 hours and MIF protein level was detected by western‐blot. B) Quantification of MIF level in (A) compared with DMSO treated control. C) **MD13** induced MIF degradation in A549 cells measured by ELISA. R^2^=0.98. D) MIF level was determined after cells were treated with the indicated concentrations of **MD13** or **MD15** for 12 hours. (*n*=2)

The concentration dependence of PROTAC **MD13‐**mediated induction of MIF degradation in A549 cells was investigated using western‐blot. **MD13** effectively induced MIF degradation at nanomolar concentrations (Figure 3 A). The MIF levels were normalized to the vehicle treated control and plotted to the respective **MD13** concentrations. This provided a DC_50_ of around 100 nM and a maximal degradation of around 90–95 % at concentrations higher than 1 μM (Figure 3 B). The DC_50_ of **MD13** measured by ELISA assay was about 200 nM (Figure 3 C), which is in line with the result from western‐blot. Interestingly, a “Hook effect” was observed in both two assays at 20 μM of **MD13**.

A control experiment was performed to compare **MD13** as an active PROTAC and **MD15** as an inactive PROTAC (Figure 3 D). This demonstrated that **MD15** was not able to reduce the MIF levels relative to the control, thus indicating that CRBN binding is involved in the effect of PROTAC **MD13**.

**Figure 4:**
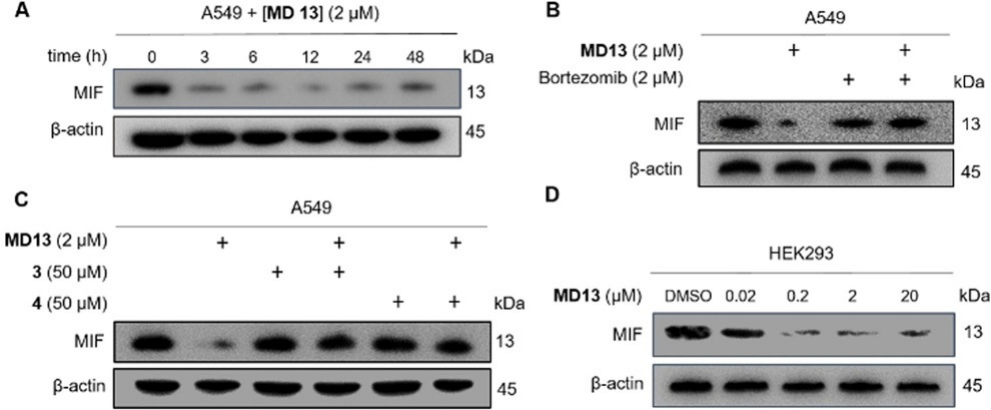
Characterization of the PROTAC activity of **MD13**. A) Time‐dependence of MIF degradation upon treatment with **MD13** in A549 cells. B) Rescue of MIF from degradation upon co‐treatment with **MD13** and Bortezomib. C) Rescue from **MD13** mediated MIF degradation after 1 hour pretreatment with MIF inhibitor **3** or CRBN inhibitor **4**. D) Concentration‐dependent MIF degradation was also observed in HEK293 cells (*n*=2).

The ability of PROTAC **MD13** to reduce the MIF levels in A549 cells was investigated further. The kinetics of MIF degradation proved to be relatively fast. Degradation was already visible after 3‐hour treatment, reaching >92 % degradation after 6 h (Figure 4 A). Only a slight recovery of the MIF levels was observed after 48 h. Combined treatment with PROTAC **MD13** and the proteasome inhibitor Bortezomib^[44]^ inhibited the degradation of MIF (Figure 4 B). Pretreatment of cells with either MIF inhibitor **3** or CRBN inhibitor **4** to outcompete the formation of ternary E3 ligase—MIF complex rescued MIF from degradation (Figure 4 C). The PROTAC **MD13** also proved to be active in HEK293 cells, where it induced more than 90 % MIF degradation at a concentration of 200 nM (Figure 4 D). Taken together, the results demonstrate that the activity of **MD13** depends on binding to both MIF and CRBN as well as on proteasome activity and that near complete MIF degradation is observed in the low micromolar range, which indicates that **MD13** is a potent MIF‐directed PROTAC.

### Anti‐Proliferative Effect of MD13

**Figure 5:**
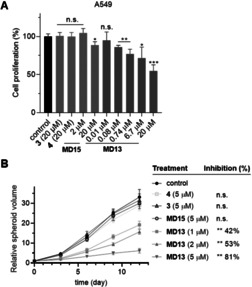
**MD13** treatment inhibits A549 cell growth. A) Proliferation of A549 cells were inhibited by treatment of **MD13** for 72 hours. The resulting cells were quantified by CyQUANT^®^ assays and compared with the vehicle control. B) The growth of A549 cell spheroids were inhibited by treatment of **MD13** for every three days. The growth curves were obtained relative to the untreated spheroids (day 0). Values are shown as means ± SD (*n*=3 spheroids/time point, *p<0.05, **p<0.01 and ***p<0.001 vs. control).

After having identified **MD13** acts as a PROTAC that effectively reduces the MIF levels, we employed this PROTAC to verify the role of MIF in proliferation of A549 cells. As a first step, the toxicity of **MD13** was investigated using the MTS assay, which indicated that **MD13** did not inhibit cell viability at concentrations below 20 μM for a treatment of 24 hours (Figure S7). We next evaluated its effects on cell proliferation, which indicated that **MD13** inhibited the growth of A549 cells in a dose‐dependent manner (Figure 5 A). The inhibitory effect became visible at nanomolar concentrations and reached about 50 % inhibition of cell proliferation at a concentration of 20 μM. In contrast, the inactive control compound **MD15** showed almost no inhibition of the proliferation of A549 cells. MIF inhibitor **3** and CRBN inhibitor **4** were also included as controls, both of which had no effect on the proliferation of cells with concentrations up to 20 μM. Taken together, these experiments indicates that the MIF‐directed PROTAC **MD13** inhibited cell proliferation of A549 cancer cells.

A 3D spheroid model was employed to investigate the effect of longer term **MD13** treatment in a more complex model for tumor growth. The 3D spheroid model was established using A549 cancer cells by a method adapted from Feng et al.^[48]^ The spheroids were grown over a 12‐day period in absence or presence of PROTAC **MD13**. Each spheroid was prepared from about 1000 A549 cells. After three‐day incubation, these spheroids were treated with 1, 2, or 5 μM of **MD13** with 72 hours intervals over 12 days. Spheroid growth was monitored by measuring the diameter and this was compared to day 0 of the treatment. The tumor spheroids in **MD13** treated groups were significant smaller compared to the control group (Figure 5 B and Figure S9). With continuous exposure to 1, 2, or 5 μM of **MD13** for 12 days, the growth of the spheroid tumor volume was inhibited by 42 %, 53 %, and 81 % compared with control group, respectively. In contrast, 5 μM of the PROTAC‐inactive control compound **MD15**, **3**, or **4** showed no significant influence on the spheroid tumor growth. Collectively, our results indicate that the MIF‐directed PROTAC **MD13** effectively inhibits proliferation of A549 cancer cells in a spheroid tumor model.

### MD13 Arrests Cells at G2/M Phase of the Cell Cycle

**Figure 6:**
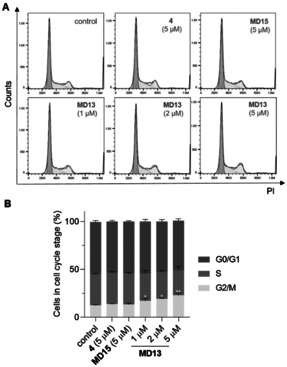
Induction of cell cycle arrest by the MIF‐directed PROTAC **MD13** in A549 cells. A) A549 cells were treated with **MD13**, **4** or **MD15** at the indicated concentrations for 48 h. The graphs show the representative cell cycle distribution of propidium‐iodide stained cells assessed by flow cytometry. B) Quantification of cells in each stage of the cell cycle by FlowJo. Data are shown as mean±SD of three replicates. *p<0.05 and **p<0.01 vs. vehicle group.

The effect of MIF‐directed PROTAC **MD13** on cell cycle progression was further analyzed using flow cytometry (Figure 6). A549 cells were treated with **MD13** at concentrations of 1, 2, or 5 μM for the duration of 48 h before analysis using flow cytometry. Our results showed that **MD13** dose‐dependently induced cell cycle arrest at the G2/M phase in A549 cells. The proportion of cells at the G2/M phase is 12 % for the control group. This percentage increases to 17 %, 19 %, and 23 % upon treatment with 1, 2, and 5 μM **MD13**, respectively. In contrast, little or no effect on the cell cycle was observed upon treatment with 5 μM of the inactive control **MD15**. These results indicate **MD13** induces inhibition of cell cycle progression, which can explain the observed inhibition of cell proliferation.

### MD13 Inhibits ERK Signaling

**Figure 7:**
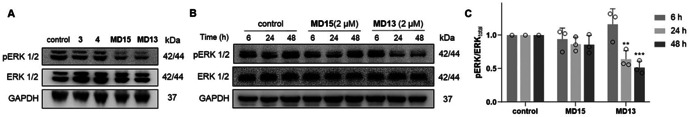
Effect of the MIF‐directed PROTAC on ERK phosphorylation in A549 cells. A) A549 cells were treated with 2 μM of **MD13**, **MD15**, **3**, **4**, or DMSO for 24 h, the pERK, total ERK and GAPDH was examined by immunoblots. B) A549 cells were treated with **MD13**, **MD15** or DMSO for 6, 24 or 48 h, the pERK, total ERK and GAPDH was examined. C) Quantification of the pERK level using pERK:ERK ratio, normalized to control group at time points indicated. GAPDH was used as a loading control on western blots. Data are shown as mean±SD of three replicates. **p<0.01 and ***p<0.001 vs. vehicle group.

The effect of treatment with MIF‐directed PROTAC **MD13** on MIF‐related signaling pathways was investigated by assessment of ERK phosphorylation using western blot analysis. Treatment with 2 μM **MD13** proved to inhibit ERK phosphorylation in A549 by about 50 % after 24‐hour treatment, which persisted at 48 h. In contrast, the cells exhibited no significant decrease on the pERK levels after incubation with the control compound **3**, **4** for 24 h or **MD15** for 6 h, 24 h, or 48 h (Figure 7). Thus treatment with the MIF‐directed PROTAC **MD13** inhibits ERK phosphorylation as a MIF‐related signaling event.

## Conclusion

Overexpression of MIF was found to stimulate proliferation of cancer cells via activation of the ERK/MAPK pathway and inhibition of the p53 pathway.[^49^, ^50^] Therefore, a number of MIF targeting modalities have been reported as potential treatments, including mAbs,^[51]^ peptides,^[52]^ small‐molecule inhibitors[^24^, ^25^] etc. These modalities have been successfully applied in animal models for MIF‐related diseases.^[52]^ However, there is no clinically approved MIF‐directed drug available yet. Use of PROTACs that trigger degradation of the MIF protein provides novel opportunities that might be particularly relevant for MIF. Importantly, MIF is involved in protein‐protein interactions, such as the MIF‐CD74 receptor interaction for which the interactions site is known to be located in close proximity of the MIF tautomerase active site.^[3]^ However, other protein‐protein interactions might occur at different locations of the MIF protein, thus making approaches aimed at MIF tautomerase activity ineffective. In this perspective the value of MIF‐directed PROTACs becomes clear, because the high affinity ligands identified for the MIF tautomerase active site can be employed to induce degradation of the MIF protein as a whole, thus diminishing MIF from its effector network.

The development of MIF‐directed PROTACs requires the synthesis of heterobifunctional ligands that are able to bind both MIF and E3 ubiquitin ligase. Optimization of the linker is required to achieve a proper orientation to trigger ubiquitination and subsequent degradation. To synthesize MIF‐targeting PROTACs, we tethered MIF binder **2**[^27^, ^28^] or **3**[^27^, ^29^] with the cereblon E3 ligase ligand pomalidomide by a variety of linkers constructed by click reactions or amidation coupling reactions. Upon exploration of the structure–activity relationships for MIF degradation, we identified **MD9** as the first MIF‐directed PROTAC. Further optimization of the linker length provides **MD13** as a MIF‐directed PROTAC with improved potency, which proved to trigger almost complete (90–95 %) degradation of MIF in the low micromolar range and a DC_50_ of around 100 nM on A549 cells. The potency of **MD13** is comparable to PROTACs directed at other protein targets.^[38]^ Fluorescence microscopy demonstrated that **MD13** effectively entered the cytosol and reduced the MIF protein levels by about 80 % within 3 hours. The reduction in MIF protein levels upon treatment with 2 μM of the MIF‐directed PROTAC **MD13** was still observed after 48 hours. As a bona fide MIF‐targeting PROTAC, **MD13** should induce the degradation through the formation of a ternary complex, which is followed by ubiquitination and proteasome‐mediated proteolysis. Accordingly, rescue assays were conducted using **3** as a competitor for MIF binding, **4** as a competitor of E3 ligase binding and bortezomib as a proteasome inhibitor. Our results showed that **3**, **4**, and bortezomib were all able to abolish the **MD13** triggered degradation, thus indicating that the activity of **MD13** depends on MIF binding, CRBN‐binding, and proteasome mediated degradation. Importantly, the control compound **MD15**, which contains an impaired E3 ligase ligand, has no effect on MIF protein level. Taken together, **MD13** proved to be an effective and potent MIF‐directed PROTAC.

The effect of **MD13** on cell proliferation was evaluated using cell culture assays on A549 cells. A monolayer cell culture assay demonstrated that **MD13** inhibited proliferation of A549 cells to a maximum of about 50 % at 20 μM. In contrast, the CRBN inactive control **MD15**, the MIF tautomerase inhibitor **3**, or the E3 ligase ligand **4** had no or little effect on cell proliferation. Also a spheroid cell culture assay was employed because such assays mimic the main features of solid human tumors, such as their structural organization, cellular layered assembling, hypoxia, and nutrient gradients.^[53]^ In this spheroid assay, **MD13** inhibited the growth of the spheroid volume by 53 % and 81 % upon treatment with 2 and 5 μM of **MD13** respectively. These results indicate that depletion of MIF using MIF‐directed PROTACs provides a strong reduction of cell proliferation, which is consistent with the results of siRNA mediated MIF silencing.[^9^, ^54^]

Growth of cancer cells is characterized by ordered progression of the cell cycle.^[55]^ MIF coordinates the cell cycle through the association with the Jab1/CSN5 subunit of the COP9/CSN signalosome,^[56]^ which plays a central role in the assembly of SCF complexes by removal of Nedd8 from Cullin.[^57^, ^58^, ^59^] MIF knockout leads to DNA damage and stalled replication.^[60]^ Treatment of A549 cancer cells with the MIF‐directed PROTAC **MD13** increased the number of cells in the G2/M phase thus indicating inhibition of cell cycle progression. MIF as a growth factor stimulates cell cycle progression through the MAPK pathway.[^61^, ^62^] Our results also demonstrate that **MD13** treatment attenuates the MAPK signaling by reducing ERK phosphorylation. This result is again in line with the effect observed upon siRNA‐mediated downregulation of the MIF protein levels.^[54]^ Collectively, these results indicate that the MIF‐directed PROTAC **MD13** reduces the MIF protein levels and inhibits cell proliferation in both 2D‐ and 3D‐ cell culture, which can be explained by inhibition of ERK phosphorylation and cell cycle progression.

In conclusion, we have developed a potent MIF‐directed PROTAC **MD13** that induces MIF degradation in A549 and HEK 293 cells. **MD13** effectively reduces the MIF protein level in A549 cells in a time‐, cereblon‐, and proteasome‐ dependent manner. Fluorescence microscopy demonstrates that **MD13** enters A549 cells with concomitant reduction of the MIF levels. **MD13** inhibited proliferation by about 50 % at micromolar concentrations in a 2D cell culture assay using A549 cells. A 3D cell culture also using A549 cells showed an even more pronounced effect with 80 % reduction of cell proliferation at 5 μM **MD13**. FACS analysis demonstrated that **MD13** treatment induced cell cycle arrest in the G2/M phase. **MD13** treatment also inhibited ERK phosphorylation, thus indicating that MIF degradation also inhibits signaling pathways that respond to MIF signaling and promote cell proliferation. In conclusion, the MIF‐directed PROTAC **MD13** mediates MIF degradation, which consequently results in inhibition of cell proliferation in 2D and 3D cell cultures, which can be explained by cell cycle arrest and inhibition of the MAPK signaling pathway. Altogether, this study demonstrates that MIF‐directed PROTACs are novel modalities in MIF‐directed drug discovery for oncology and other MIF related diseases.

## Conflict of interest

The authors declare no conflict of interest.

## Supporting information

As a service to our authors and readers, this journal provides supporting information supplied by the authors. Such materials are peer reviewed and may be re‐organized for online delivery, but are not copy‐edited or typeset. Technical support issues arising from supporting information (other than missing files) should be addressed to the authors.

Supporting Information
