# Supplementary material for: Proteolysis Targeting Chimera (PROTAC) for Macrophage Migration Inhibitory Factor (MIF) Has Anti‐Proliferative Activity in Lung Cancer Cells
Source: Angew Chem Int Ed Engl. 2021 Jun 26;60(32):17514–21. doi: 10.1002/anie.202101864 (PMC8362126; doi:10.1002/anie.202101864)
Supplement: Supplementary file 1 — Supporting Information [file ANIE-60-17514-s001.pdf]

Angewandte Chemie
Eine Zeitschrift der Gesellschaft Deutscher Chemiker

Supporting Information

Proteolysis Targeting Chimera (PROTAC) for Macrophage Migration Inhibitory Factor (MIF) Has Anti-Proliferative Activity in Lung Cancer Cells

*Zhangping Xiao+, Shanshan Song+, Deng Chen, Ronald van Merkerk, Petra E. van der Wouden, Robbert H. Cool, Wim J. Quax, Gerrit J. Poelarends, Barbro N. Melgert, and Frank J. Dekker**

anie_202101864_sm_miscellaneous_information.pdf

S1. Synthesis

S2. MIF Tautomerase activity IC_{50} study

S3. ELISA screening of MIF PROTACs

S4. Characterization of **MD9**

S5. Subcellular colocalization study

S6. Western-blot uncropped images

S7. Cell viability assay of **MD13**

S8. Cell growth, cell cycle and pERK signaling study

S9. HRMS and purity

S10**.** NMR spectra

S1. Synthesis

General. All the reagents and solvents were purchased from Sigma-Aldrich, AK Scientific, Fluorochem or Acros and were used without further purification unless stated otherwise. Reactions were monitored by thin layer chromatography (TLC) by the use of Merck silica gel 60 F_{254} plates. Spots were visualized with UV light. MP Ecochrom silica 32-63, 60 Å was used for flash column chromatography. Nuclear magnetic resonance spectra, ^{1}H NMR (500 MHz) and ^{13}C NMR (126 MHz), were recorded on a Bruker Avance 500 spectrometer. ^{1}H NMR spectra were reported in parts per million (ppm) referenced to $CDCl_3$: δ = 7.26 ppm (^{1}H) and 77.05 ppm (^{13}C) or DMSO-d6: δ = 2.50 ppm (^{1}H) and 39.52 ppm (^{13}C). The following abbreviations were used for spin multiplicity: s (singlet), d (doublet), t (triplet), q (quartet), dd (double of doublets), and m (multiplet). Coupling constants were reported in Hertz (Hz). High-resolution mass spectra were recorded using Fourier Transform Mass Spectrometry (FTMS) and electrospray ionization (ESI) on an Applied Biosystems/SCIEX API3000-triple quadrupole mass spectrometer. Purity of the compounds was determined by C18 reverse-phase high-performance liquid chromatography (HPLC) analysis to be >95%.

To prepare a focused collection of putative MIF-directed PROTACs with linkers of diverse lengths, we coupled azide functionalized MIF binders with correspondingly alkyne functionalized pomalidomide analogues using the copper(I)-catalyzed alkyne-azide cycloaddition (CuAAC) reaction. Alternatively, pomalidomide was functionalized with an appropriate carboxylic acid linker that was linked to an amine-functionalized MIF binder using an EDCI-mediated amidation reaction. The synthetic routes towards the various MIF degraders are outlined in Scheme 1-4. A series of intermediates containing MIF ligands were prepared first (Scheme 1). The amine-functionalized MIF ligand **5** was synthesized according to literature procedures[1] and coupled with carboxylic acids to provide **6a-c** in yield of 52-96%. The tert-butyloxycarbonyl (Boc) protected precursor **7** and **10** were synthesized as reported

previously.[2] Subsequently, **7** and **10** were subjected to TFA treatment for Boc deprotection to give **8** and **11** as key intermediates in yields of 34% and 13%, respectively. **9** was prepared through the amidation between **8** and 3-azidopropanoic acid in a yield of 74%. **13** was prepared from **12** by following a reported method.[2]

Scheme 1. Synthesis of intermediates for the MIF ligands part of the MIF-directed PROTACs.[a]

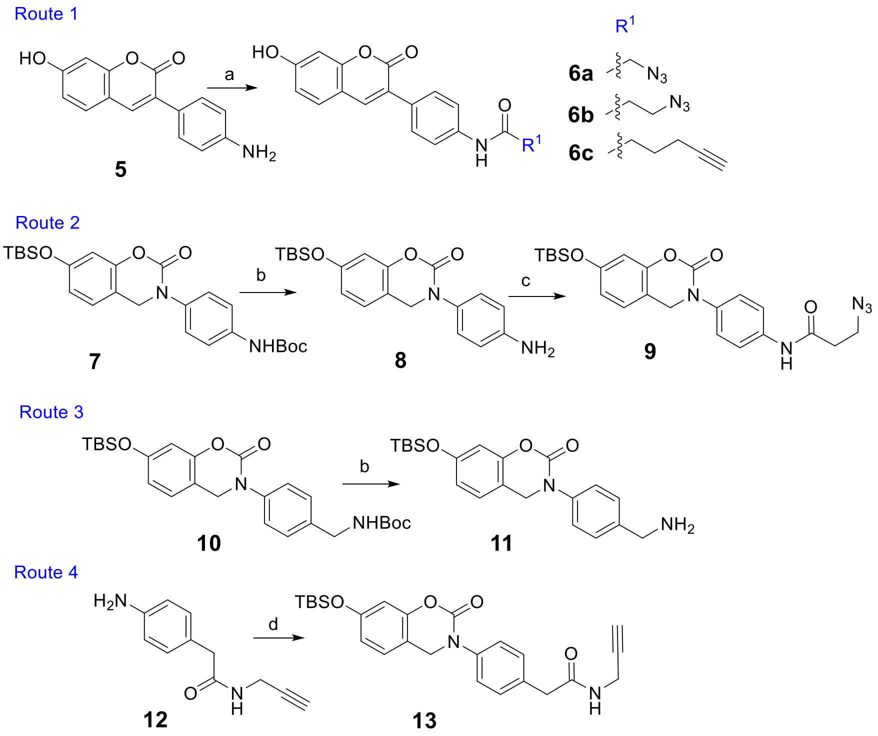

[a] Reagents and conditions: (a) carboxylic acids, EDCI·HCl, HOBt, DMF, rt; (b) TFA, CH_2Cl_2, 0 °C to rt; (c) 3-azidopropanoic acid, EDCI·HCl, HOBt, CH_2Cl_2, rt; (d) i. 4-((*tert*-butyldimethylsilyl)oxy)-2-hydroxybenzaldehyde, methanol, rt; ii. $NaBH_4$, rt; iii. Carbonyldiimidazole, CH_2Cl_2, rt.

The synthesis of the CRBN ligand containing intermediates was shown in Scheme 2. 4-Fluoro-substituted thalidomide **14** was used as starting material to react with different amines to provide **15a-e**. [3] Methylation of **14** provided **16** as a precursor for synthesis of **17**, which has an impaired CRBN binding moiety.[4] Intermediates **18a-d** were constructed through amidation of pomalidomide **4** with different alkyne or azide linked carboxylic acids.[5]

Scheme 2. Synthesis of intermediates containing the CRBN ligands for E3 ligase recruitment by the MIF-directed PROTACs.[a]

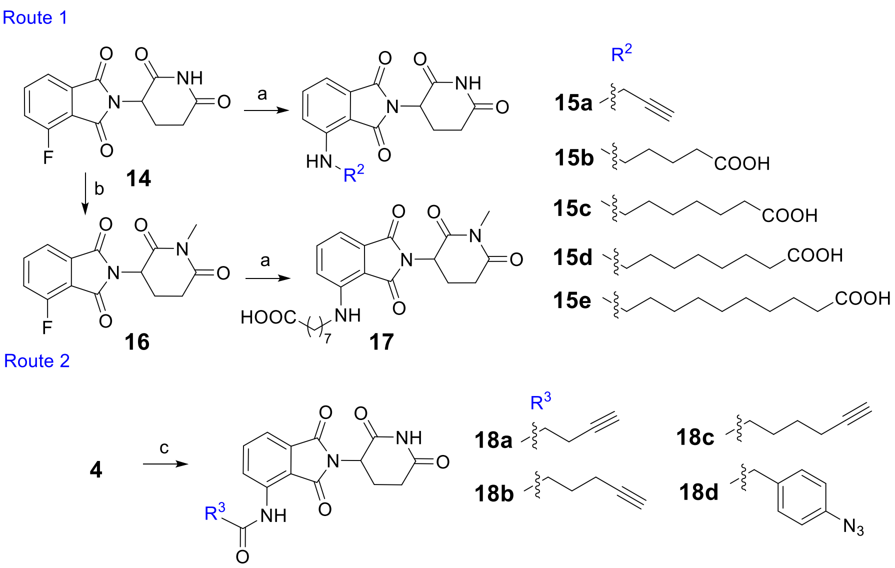

[a] Reagents and conditions: (a) Corresponding amines, DIPEA, DMF, 90 °C; (b) MeI, DMF, rt; (c) i. Carboxylic acids, $SOCl_2$, reflux; ii. tetrahydrofuran, reflux.

The preparation of MIF degraders and control compound are shown in Scheme 3 and Scheme 4. The PROTACS in Group 1 were produced by linkage of corresponding alkynes and azides using the CuAAC reaction. **MD10-12** were also synthesized via CuAAC reactions, whereas **MD7-9**, **13**, **14**, and control compound **MD15** were prepared by amidation reactions of corresponding amines and carboxylic acids. The synthesis involved, as a final step, removal of the tert-Butyldimethylsilyl protection group, which proceeded in high yields. All final compounds were purified with flash column chromatography and characterized by ^{1}H and ^{13}C NMR spectroscopy, and HRMS (Supporting Information).

Scheme 3. Synthesis of MIF degraders containing7-hydroxycoumarin as MIF ligand part. [a]

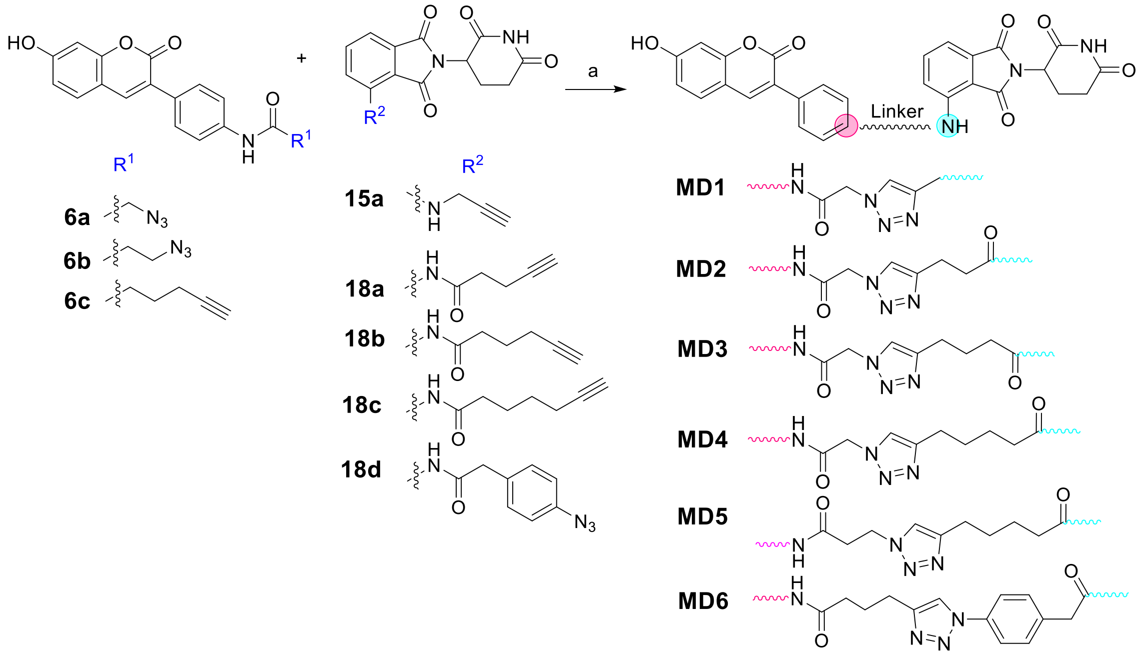

[a] Reagents and conditions: (a) Azides, alkynes, $CuSO_4$, sodium ascorbate, methanol, rt.

Scheme 4. Synthesis of MIF degraders containing 7-hydroxy-3,4-dihydrobenzoxazin-2-ones as MIF ligand part. [a]

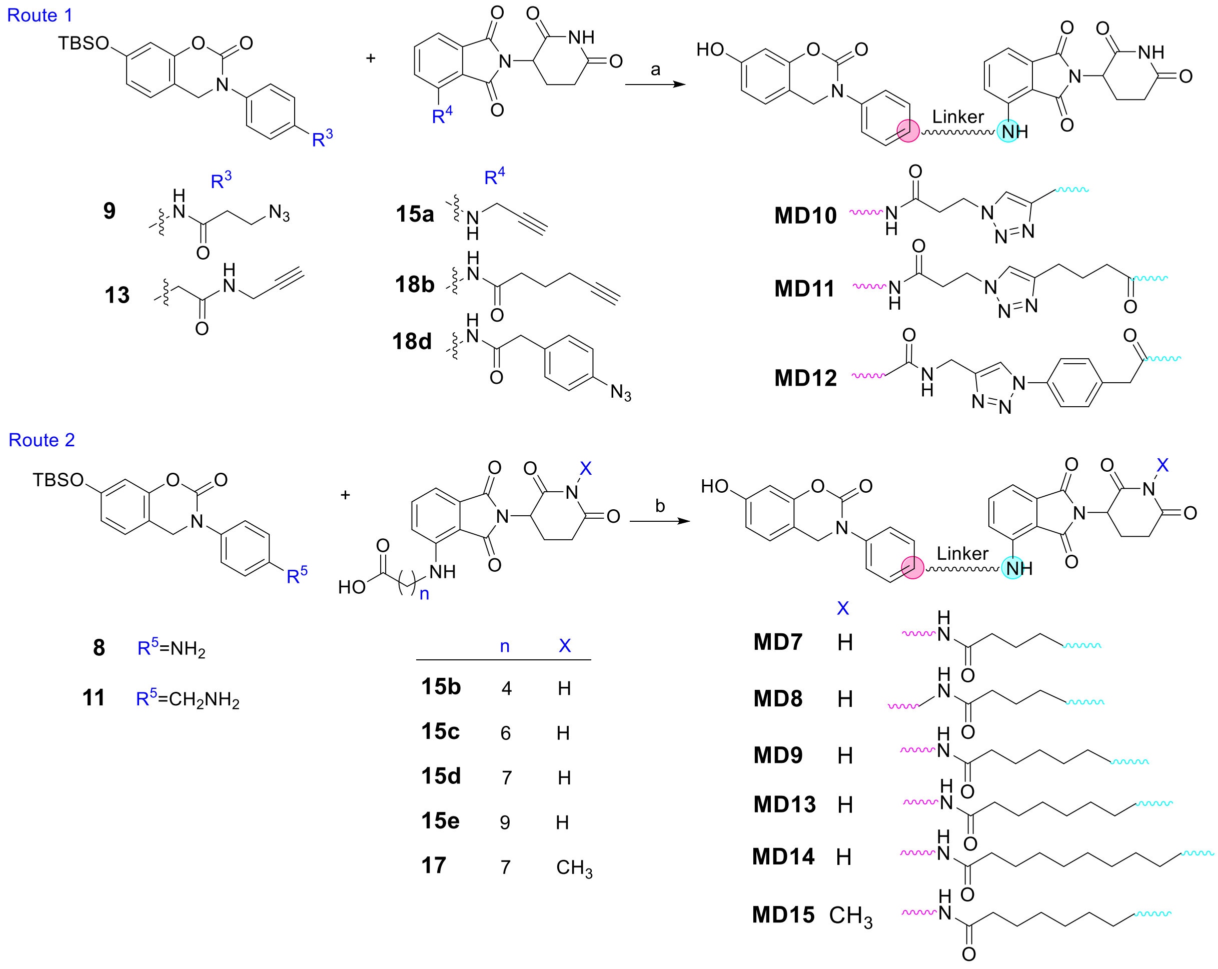

[a] Reagents and conditions: (a) azides, alkynes, $CuSO_4$, sodium ascorbate, methanol, rt; (b) i. amines, carboxylic acids, EDCI·HCl, HOBt, CH_2Cl_2, rt; ii. TBAF, THF, rt.

3-(4-Aminophenyl)-7-hydroxy-2*H*-chromen-2-one (5). 7-Hydroxy-3-(4-nitrophenyl)-coumarin (0.85 g, 3.0mmol) was prepared according to literature[1] and dissolved in ethanol (10 mL) in a three-neck flask and Pd/C (10% wt, 40 mg) was added as a catalyst. The resulting suspension was charged with H_2 gas and stirred at room temperature for 4 hour. The suspension was filtered to remove the catalyst and the solvent was evaporated. The residue was recrystallized in methanol to provide 0.68 g of the desired product as yellow powder, yield 89%. ^{1}H NMR (500 MHz, DMSO-d_6) δ 10.45 (s, 1H), 7.94 (s, 1H), 7.53 (d, J = 8.4 Hz, 1H), 7.42 (d, J = 8.4 Hz, 2H), 6.78 (dd, J = 8.5, 1.8 Hz, 1H), 6.72 (d, J = 2.0 Hz, 1H), 6.65 – 6.55 (m, 2H), 5.33 (s, 2H). ^{13}C NMR (126 MHz, DMSO) δ 160.8, 160.8, 154.7, 149.4, 137.9, 129.8, 129.4, 123.2, 122.5, 113.7, 113.6, 112.8, 102.1. EIS-MS: 254.24.

2-azido-N-(4-(7-Hydroxy-2-oxo-2*H*-chromen-3-yl)phenyl)acetamide (6a). The synthesis proceeded in two steps. The first step is the synthesis of 2-azidoacetic acid. Towards this aim 2-bromoacetic acid (1.75 g, 12.7 mmol) was dissolved in H_2O (20 mL) and sodium azide (1.46 g, 22.5 mmol) was added portionwise over 10 minutes and the mixture was stirred overnight at room temperature. The reaction mixture was acidified with concentrated HCl to pH 2.0 and extracted with ethyl acetate (3 × 40 mL). The combined organic layers were washed with brine (30 mL). The solvent was dried over Mg_2SO_4 and evaporated to obtain 2-azidoacetic acid as transparent oil, which was used without further purification (yield was estimated to be quantitative). ^{1}H NMR (500 MHz, Chloroform-d) δ 7.16 (s, 1H), 3.99 (s, 1H). ^{13}C NMR (126 MHz, $CDCl_3$) δ 173.54, 50.05. Compound **5** (200 mg, 0.8 mmol) and 2-azidoacetic acid (90 mg, 1.0 mmol) were dissolved in anhydrous DMF (2 mL). EDCI·HCl (200 mg, 1.0 mmol) and HOBt (40 mg, 0.3 mmol) were added into the mixture. The resulting suspension was stirred under argon atmosphere at room temperature overnight. H_2O (15 mL) was used to dilute the

reaction solution. The mixture was washed with ethyl acetate (20 mL × 3). The combined organic layers were washed with brine (20 mL) and dried over $MgSO_4$. The product was purified by column chromatography using CH_2Cl_2:MeOH 80:1 (*v/v*) as eluent. 0.2 g product was obtained as pale yellow solid, yield 75%. ^{1}H NMR (500 MHz, DMSO-d_6) δ 10.61 (s, 1H), 10.29 (s, 1H), 8.15 (s, 1H), 7.70 (d, J = 8.7 Hz, 2H), 7.66 (d, J = 8.8 Hz, 2H), 7.60 (d, J = 8.6 Hz, 1H), 6.83 (dd, J = 8.5, 2.1 Hz, 1H), 6.76 (d, J = 1.8 Hz, 1H), 4.08 (s, 2H). ^{13}C NMR (126 MHz, DMSO) δ 166.9, 161.6, 160.6, 155.2, 140.8, 138.7, 130.7, 130.4, 129.2, 122.1, 119.3, 113.9, 112.5, 102.2, 51.8.

3-Azido-*N*-(4-(7-hydroxy-2-oxo-2*H*-chromen-3-yl)phenyl)propanamide (6b). 3-Azidopropanoic acid was prepared using the same method for preparing 2-azidoacetic acid by using 3-bromopropanoic acid as starting material. ^{1}H NMR (500 MHz, Chloroform-*d*) δ 3.62 (s, 2H), 2.67 (s, 2H). ^{13}C NMR (126 MHz, $CDCl_3$) δ 176.93, 46.41, 33.73. Compound **5** (70 mg, 0.3 mmol) and 3-Azidopropanoic acid (50 mg, 0.4 mmol) were mixed in DMF (2 mL), followed by adding EDCI·HCl (80 mg, 0.4 mmol) and HOBt (40 mg, 0.3 mmol). The suspension was stirred at room temperature overnight. The same purification procedure as **6b** was applied to provide **6a** as pale yellow powder with yield of 52%. ^{1}H NMR (500 MHz, DMSO-d_6) δ 10.59 (s, 1H), 10.19 (s, 1H), 8.14 (s, 1H), 7.71 – 7.64 (m, 4H), 7.61 (d, J = 8.5 Hz, 1H), 6.83 (d, J = 8.4 Hz, 1H), 6.76 (s, 1H), 3.69 – 3.59 (m, 2H), 2.69 – 2.61 (m, 2H). ^{13}C NMR (126 MHz, DMSO) δ 169.2, 161.5, 160.6, 155.2, 140.6, 139.3, 130.3, 129.1, 122.2, 119.1, 115.1, 113.9, 112.5, 102.1, 47.3, 36.1. EIS-MS: 273.23.

***N*-(4-(7-hydroxy-2-oxo-*2H*-chromen-3-yl)phenyl)hex-5-ynamide (6c). 6c** was synthesized as described for **6b** by using compound **5** (15 mg) and 5-Hexynoic acid (10 mg) as starting materials with yield of 96%. ^{1}H NMR (500 MHz, DMSO-d_6) δ 10.59 (s, 1H), 10.07 (s, 1H), 8.13 (s, 1H), 7.66 (s, 4H), 7.60 (d, J = 8.5 Hz, 1H), 6.83 (dd, J = 8.5, 2.2 Hz, 1H), 6.76 (d, J = 2.1 Hz, 1H), 2.84 (t, J = 2.6 Hz, 1H), 2.45 (t, J = 7.5 Hz, 2H), 2.24 (td, J = 7.0, 2.6 Hz, 2H),

1.79 (q, J = 7.2 Hz, 2H). ^{13}C NMR (126 MHz, DMSO) δ 171.2, 161.5, 160.6, 155.2, 140.6, 139.5, 130.3, 130.0, 129.0, 122.2, 119.0, 113.8, 112.5, 84.5, 72.1, 35.6, 24.3, 17.8.

***tert*-Butyl (4-(7-((tert-butyldimethylsilyl)oxy)-2-oxo-2*H*-benzo[*e*][1,3]oxazin-3(4*H*)-yl)phenyl) carbamate (7).** The procedures for synthesis of compound **7** was adapted from reported methods.[6] *tert*-Butyl (4-aminophenyl)carbamate (0.80 g, 3.8 mmol) and 3,4-dimethoxyaniline (1.0 g, 4.0 mmol) were dissolved in ethanol (20 mL) and stirred at room temperature for 1 hour. The suspension turned deep yellow. Subsequently, the mixture was cooled on an ice-bath and $NaBH_4$ (0.25 mg, 6.6 mmol) was added portionwise to the reaction mixture. The resulting mixture was stirred at room temperature for 1 hour until the suspension became transparent. Subsequently, H_2O (25 mL) was poured into the mixture followed by extraction with CH_2Cl_2 (40 mL × 3). The combined organic layers were collected and dried over $MgSO_4$. After filtration, the solution was evaporated. The remaining solid was dissolved in CH_2Cl_2 (20 mL). Then, carbonyldiimidazole (0.16g, 1.0 mmol) was added and the mixture stirred for 16 hours at room temperature. CH_2Cl_2 (20 mL) was added to dilute the mixture. Subsequently, the mixture was washed with HCl solution (1 N, 50 mL), a saturated $NaHCO_3$ solution (50 mL) and brine (50 mL), dried over $MgSO_4$ and concentrated under vacuum. The resulting mixture was purified with column chromatography by using petroleum ether : ethyl acetate 6:1 (v/v) as eluent. The product was obtained as pale yellow solid in a yield of 46% (0.82 g). ^{1}H NMR (500 MHz, Chloroform-*d*) δ 7.45 (d, J = 8.6 Hz, 2H), 7.33 (d, J = 8.9 Hz, 2H), 6.99 (d, J = 8.3 Hz, 1H), 6.66 (dd, J = 8.2, 2.3 Hz, 1H), 6.63 (d, J = 2.3 Hz, 1H), 6.59 (s, 1H), 4.76 (s, 2H), 1.55 (s, 9H), 1.01 (s, 9H), 0.24 (s, 6H). ^{13}C NMR (126 MHz, $CDCl_3$) δ 156.4, 152.7, 150.4, 137.5, 136.6, 130.6, 126.1, 125.8, 119.3, 116.6, 110.5, 107.9, 64.5, 50.6, 28.3, 25.6, 18.2, 14.2, -4.4.

3-(4-Aminophenyl)-7-((*tert*-butyldimethylsilyl)oxy)-3,4-dihydro-2*H*-benzo[*e*][1,3]oxazin-2-one (8). Compound **7** (0.20 g, 0.40 mmol) was dissolved in CH_2Cl_2 (5

mL) and cooled on an ice-bath. TFA (0.40 mL, 5.2 mmol) was added into the solution portionwise in 5 minutes. The suspension was stirred at room temperature for 16 hours. Subsequently, H_2O (20 mL) was added into the suspension to quench the reaction. The resulting mixture was extracted with CH_2Cl_2 (20 mL × 3). The organic layer was collected and the product was purified with chromatography using petroleum ether : ethyl acetate 4:1 (v/v) as eluent. The product was obtained as pale yellow solid in a yield of 34% (0.13 g). ^{1}H NMR (500 MHz, Chloroform-*d*) δ 7.29 (s, 1H), 7.17 (d, *J* = 8.6 Hz, 2H), 6.97 (d, *J* = 8.2 Hz, 1H), 6.73 (d, *J* = 8.6 Hz, 2H), 6.65 (dd, *J* = 8.2, 2.3 Hz, 1H), 6.62 (d, *J* = 2.2 Hz, 1H), 4.73 (s, 2H), 1.01 (s, 9H), 0.24 (s, 6H). ^{13}C NMR (126 MHz, $CDCl_3$) δ 156.3, 150.6, 150.5, 145.9, 132.7, 126.7, 125.8, 116.5, 115.6, 110.6, 107.9, 51.0, 25.6, 18.3, -4.4.

3-azido-*N*-(4-(7-((*tert*-butyldimethylsilyl)oxy)-2-oxo-2*H*-benzo[*e*][1,3]oxazin-3(4*H*)-yl)phenyl)propanamide (9). To synthesize compound **9**, the same protocol was followed as synthesis of **6a**. **8** (0.2 g, 0.5 mmol) and 3-azidopropanoic acid (75 mg, 0.6 mmol) was applied as starting materials for amidation step. The product was purified with chromatography using CH_2Cl_2:MeOH 60:1 (*v/v*) as eluent. 0.19 g white solid was obtained as product, yield 74%. ^{1}H NMR (500 MHz, Chloroform-*d*) δ 8.32 (s, 1H), 7.52 (d, *J* = 8.7 Hz, 2H), 7.31 (d, *J* = 8.8 Hz, 2H), 6.98 (d, *J* = 6.8 Hz, 1H), 6.67 (m, 2H), 4.76 (s, 2H), 3.71 (t, *J* = 6.2 Hz, 2H), 2.65 (t, *J* = 6.4 Hz, 2H), 1.01 (s, 9H), 0.25 (s, 6H).

***tert*-Butyl (4-(7-((*tert*-butyldimethylsilyl)oxy)-2-oxo-2*H*-benzo[*e*][1,3]oxazin-3(4*H*)-yl)benzyl)carbamate (10).** Compound **10** was synthesized using the same method as the preparation of compound **7**. tert-Butyl (4-aminobenzyl)carbamate (0.41 g, 1.8 mmol) was used as starting material. The product was purified using chromatography with petroleum ether : ethyl acetate 5:1 (v/v). 0.58 g white solid was obtained, yield 67%. ^{1}H NMR (500 MHz, Chloroform-*d*) δ 7.38 (s, 4H), 6.99 (d, *J* = 8.2 Hz, 1H), 6.67 (dd, *J* = 8.2, 2.3 Hz, 1H), 6.64 (d, *J* = 2.2 Hz, 1H), 4.78 (s, 2H), 4.35 (s, 2H), 1.62 (s, 2H), 1.49 (s, 9H), 1.01 (s, 9H), 0.24 (s, 6H).

^{13}C NMR (126 MHz, $CDCl_3$) δ 156.5, 150.4, 140.9, 138.2, 128.5, 125.8, 125.5, 116.6, 110.4, 108.0, 50.4, 44.2, 28.4, 25.6, 18.2, -4.4.

3-(4-(Aminomethyl)phenyl)-7-((*tert*-butyldimethylsilyl)oxy)-3,4-dihydro-2*H*-benzo[*e*][1,3]oxazin-2-one (11). The method for preparing **7** was applied to synthesize **11**. Compound **11** (0.48 g, 1.0 mmol) was used as starting material. The product was purified with chromatography with CH_2Cl_2 : MeOH 20:1 (v/v) as eluent. 50 mg white solid was obtained as product, yield 13%. ^1H NMR (500 MHz, Chloroform-*d*) δ 7.63 (d, *J* = 7.7 Hz, 2H), 7.41 – 7.19 (m, 3H), 6.97 (d, *J* = 8.0 Hz, 1H), 6.63 (d, *J* = 8.0 Hz, 1H), 6.58 (s, 1H), 4.68 (s, 2H), 4.09 (s, 2H), 1.00 (s, 9H), 0.22 (s, 6H). ^{13}C NMR (126 MHz, $CDCl_3$) δ 156.4, 150.7, 150.0, 141.6, 133.0, 130.9, 126.1, 125.6, 116.8, 110.4, 107.8, 50.0, 43.3, 25.6, 18.2, -4.5.

2-(4-Aminophenyl)-*N*-(prop-2-yn-1-yl)acetamide (12). 2-(4-Nitrophenyl)acetic acid (1.2 g, 6.6 mmol) was added into a mixture of CH_2Cl_2 (10 mL) and $SOCl_2$ (2 mL). The suspension was refluxed for 1 hour. The volatiles were removed by evaporation under reduced pressure and the residue was dissolved in anhydrous THF (10 mL).2-Propargylamine (0.55 mL, 8.6 mmol) was added to the solution, which was subsequently refluxed for 2 hours. After cooling down to room temperature, the amide product was precipitated and collected by filtration. The obtained solid was dissolved in a mixture of ethanol (10 mL), EtOAc (5 mL) H_2O (3 mL) and AcOH (2 mL). Then, a powder of iron (1.6 g) was added into the mixture, which was refluxed for 4 hours. The resulting mixture was filtered and washed with EtOAc (20 mL × 2). The filtrate was collected and washed with saturated $NaHCO_3$ (30 mL) and brine (30 mL). A rotary evaporator was used to remove the solvent and the residue was purified with chromatography with CH_2Cl_2 : MeOH 30:1. 0.80 g white solid was obtained as product, yield 65%. ^1H NMR (500 MHz, Chloroform-*d*) δ 7.05 (d, *J* = 8.0 Hz, 2H), 6.70 (d, *J* = 8.0 Hz, 2H), 5.67 (s, 1H), 4.01 (dd, *J* = 5.0, 1.9 Hz, 2H), 3.77 (s, 2H), 3.51 (s, 2H), 2.30 – 2.10 (m, 1H).

2-(4-(7-((*tert*-butyldimethylsilyl)oxy)-2-oxo-2*H*-benzo[*e*][1,3]oxazin-3(4*H*)-yl)phenyl)-*N*-(prop-2-yn-1-yl)acetamide (13). Compound **13** was prepared by using the same method as used for synthesis of **7**. **12** (340 mg, 1.8 mmol) was used as starting material and 0.20 g white solid was obtained as product, overall yield 24%. ^{1}H NMR (500 MHz, Chloroform-*d*) δ 7.38 (d, *J* = 8.4 Hz, 2H), 7.34 (d, *J* = 8.5 Hz, 2H), 6.99 (d, *J* = 8.3 Hz, 1H), 6.66 (dd, *J* = 8.2, 2.3 Hz, 1H), 6.63 (d, *J* = 2.3 Hz, 1H), 6.17 (t, *J* = 5.4 Hz, 1H), 4.77 (s, 2H), 4.01 (dd, *J* = 5.4, 2.5 Hz, 2H), 3.56 (s, 2H), 1.00 (s, 9H), 0.24 (s, 6H). ^{13}C NMR (126 MHz, $CDCl_3$) δ 170.3, 156.5, 150.4, 150.3, 140.8, 133.8, 130.4, 125.9, 125.8, 116.7, 110.4, 107.9, 79.5, 71.5, 50.3, 42.7, 29.3, 25.6, 18.2, -4.4. EIS-MS: 359.27.

2-(2,6-Dioxopiperidin-3-yl)-4-fluoroisoindoline-1,3-dione (14). [3] In a round-bottom flask, 3-Fluorophthalic anhydride (3.8 g, 26.7 mmol), 3-Aminoperidine-2,6-dione hydrochloride (4.0 g, 26.9 mmol), and sodium acetate (2.6 g, 31.7 mmol) were mixed in AcOH (35 mL). The resulting reaction mixture was heated to reflux overnight. After cooling to room temperature, the AcOH was evaporated and the residue was purified by column chromatography with CH_2Cl_2 : MeOH 50:1 (v/v) to obtain compound **14** as a slightly yellow solid (6.1 g, 83% yield). ^{1}H NMR (500 MHz, DMSO-*d*$_6$) δ 11.16 (s, 1H), 7.99 – 7.92 (m, 1H), 7.80 (d, *J* = 7.3 Hz, 1H), 7.75 (t, *J* = 8.9 Hz, 1H), 5.17 (dd, *J* = 12.9, 5.4 Hz, 1H), 2.95 – 2.85 (m, 1H), 2.66 – 2.52 (m, 2H), 2.08 (dd, *J* = 10.2, 4.8 Hz, 1H). ^{13}C NMR (126 MHz, DMSO-*d*$_6$) δ 157.3 (d, *J* = 262.3 Hz), 138.5, 133.9, 123.4, 120.5 (d, *J* = 29.6 Hz), 117.5 (d, *J* = 12.4 Hz), 49.6, 31.3, 22.3.

General Procedure for Synthesis of Compounds 15a-e and 17. The method used here was reported in literature.[3] Corresponding amines (1.0 mmol, 1.0 equiv) of different lengths were added to a stirred solution of compound **10** (0.28 g, 1.0 mmol, 1.0 equiv) or **17** (0.29 g, 1.0 mmol, 1.0 equiv) in DMF (5.0 mL) and DIPEA (0.36 mL, 2.0 mmol). The reaction mixture was stirred at 90 °C for 12 h. Then the mixture was cooled to room temperature, poured into H_2O (20 mL), and extracted with EtOAc (20 mL × 3). The combined organic layer was washed

with brine (40 mL), dried over anhydrous $MgSO_4$. After filtration and evaporation, the crude residue was purified by column chromatography to give the intermediates.

8-((2-(2,6-Dioxopiperidin-3-yl)-1,3-dioxoisoindolin-4-yl)amino)octanoic acid (**15a**). ^{1}H NMR (500 MHz, Chloroform-*d*) δ 8.33 (s, 1H), 7.52 (dd, *J* = 8.5, 7.2 Hz, 1H), 7.15 – 7.08 (m, 1H), 6.90 (d, *J* = 8.5 Hz, 1H), 6.26 (t, *J* = 5.3 Hz, 1H), 4.94 (dd, *J* = 12.3, 5.4 Hz, 1H), 3.29 (q, *J* = 6.8 Hz, 2H), 2.95 – 2.73 (m, 3H), 2.38 (t, *J* = 7.4 Hz, 2H), 2.19 – 2.12 (m, 1H), 1.71 – 1.65 (m, 4H), 1.48 – 1.43 (m, 2H), 1.40 (dt, *J* = 7.6, 3.6 Hz, 4H). ^{13}C NMR (126 MHz, $CDCl_3$) δ 177.9, 171.2, 169.5, 168.5, 167.6, 147.0, 136.1, 132.5, 116.7, 111.4, 109.8, 48.8, 42.6, 33.6, 31.4, 29.1, 28.9, 28.8, 26.7, 24.5, 22.8. EIS-MS: 438.16.

2-(2,6-Dioxopiperidin-3-yl)-4-(prop-2-yn-1-ylamino)isoindoline-1,3-dione (**15b**). ^{1}H NMR (500 MHz, Chloroform-*d*) δ 8.44 (s, 1H), 7.53 (d, *J* = 7.2 Hz, 1H), 7.32 – 7.28 (m, 1H), 7.11 (d, *J* = 8.5 Hz, 1H), 4.99 (dd, *J* = 12.1, 5.4 Hz, 1H), 2.99 – 2.70 (m, 6H), 2.15 – 2.11 (m, 1H). ^{13}C NMR (126 MHz, DMSO) δ 173.2, 170.2, 164.4, 158.3, 156.2, 150.3, 138.5, 133.9, 123.1, 113.5, 74.2, 49.5, 43.4, 36.2, 31.4, 22.3.

5-((2-(2,6-Dioxopiperidin-3-yl)-1,3-dioxoisoindolin-4-yl)amino)pentanoic acid (**15c**). ^{1}H NMR (500 MHz, Chloroform-*d*) δ 8.31 (s, 1H), 7.52 (dd, *J* = 8.4, 7.2 Hz, 1H), 7.12 (d, *J* = 7.1 Hz, 1H), 6.91 (d, *J* = 8.5 Hz, 1H), 6.28 (t, *J* = 5.4 Hz, 1H), 4.94 (dd, *J* = 12.4, 5.3 Hz, 1H), 3.33 (q, *J* = 6.3 Hz, 2H), 2.94 – 2.76 (m, 3H), 2.43 (t, *J* = 6.9 Hz, 2H), 2.19 – 2.13 (m, 1H), 1.81 – 1.75 (m, 4H). ^{13}C NMR (126 MHz, $CDCl_3$) δ 177.4, 171.6, 169.5, 168.8, 167.6, 146.9, 136.2, 132.5, 116.6, 111.5, 110.0, 48.9, 42.3, 33.4, 31.4, 28.6, 22.8, 22.0.

7-((2-(2,6-Dioxopiperidin-3-yl)-1,3-dioxoisoindolin-4-yl)amino)heptanoic acid (**15d**). ^{1}H NMR (500 MHz, Chloroform-*d*) δ 8.34 (s, 1H), 7.60 – 7.45 (m, 1H), 7.11 (d, *J* = 7.0 Hz, 1H), 6.90 (d, *J* = 8.5 Hz, 1H), 6.26 (s, 1H), 4.94 (dd, *J* = 12.3, 5.4 Hz, 1H), 3.30 (q, *J* = 6.8 Hz, 2H),

2.94 – 2.72 (m, 3H), 2.40 (t, *J* = 7.3 Hz, 2H), 2.17 – 2.12 (m, 1H), 1.74 – 1.67 (m, 4H), 1.46 (td, *J* = 11.0, 10.1, 5.8 Hz, 4H).

10-((2-(2,6-Dioxopiperidin-3-yl)-1,3-dioxoisoindolin-4-yl)amino)decanoic acid (**15e**). ^{1}H NMR (500 MHz, Chloroform-*d*) δ 8.54 (s, 1H), 7.51 (dd, *J* = 8.5, 7.2 Hz, 1H), 7.10 (d, *J* = 7.1 Hz, 1H), 6.90 (d, *J* = 8.6 Hz, 1H), 6.25 (t, *J* = 5.2 Hz, 1H), 4.94 (dd, *J* = 12.3, 5.4 Hz, 1H), 3.28 (q, *J* = 6.8 Hz, 2H), 2.93 – 2.73 (m, 3H), 2.35 (t, *J* = 7.5 Hz, 2H), 2.18 – 2.12 (m, 1H), 1.67 (m, 4H), 1.38 (m, 10H). ^{13}C NMR (126 MHz, $CDCl_3$) δ 178.3, 171.4, 169.5, 168.6, 167.7, 147.0, 136.13, 132.5, 116.7, 111.4, 109.8, 77.3, 48.8, 42.6, 33.9, 31.42, 29.2, 29.2, 29.1, 29.1, 29.0, 26.8, 24.7, 22.8. EIS-MS: 466.40.

8-((2-(1-Methyl-2,6-dioxopiperidin-3-yl)-1,3-dioxoisoindolin-4-yl)amino)octanoic acid (**17**). ^{1}H NMR (500 MHz, Chloroform-*d*) δ 8.34 (s, 1H), 7.51 (dd, *J* = 8.5, 7.1 Hz, 1H), 7.10 (d, *J* = 7.0 Hz, 1H), 6.90 (d, *J* = 8.5 Hz, 1H), 6.64 (s, 1H), 4.94 (dd, *J* = 11.9, 6.0 Hz, 1H), 3.28 (t, *J* = 7.0 Hz, 2H), 3.21 (s, 3H), 2.83 – 2.75 (m, 3H), 2.36 (d, *J* = 7.5 Hz, 2H), 2.14 – 2.09 (m, 1H), 1.66 (dd, *J* = 13.2, 6.0 Hz, 4H), 1.45 – 1.38 (m, 6H). ^{13}C NMR (126 MHz, $CDCl_3$) δ 178.5, 171.3, 169.1, 167.8, 161.3, 147.0, 136.1, 132.5, 116.6, 111.4, 109.9, 50.5, 49.6, 42.6, 33.8, 31.5, 29.1, 28.9, 27.3, 24.6, 24.2, 22.1.

4-Fluoro-2-(1-methyl-2,6-dioxopiperidin-3-yl)isoindoline-1,3-dione (16). To a round-bottom flask containing **14** (0.55 g, 2.0 mmol) in anhydrous DMF (4 mL), MeI (0.16 mL, 2.5 mmol) and K_2CO_3 (0.32 g, 2.4 mmol) were added. The suspension was stirred at room temperature for 12 hours. The resulting mixture was diluted with water (20 mL) to precipitate the product. The slurry mixture was filtrated and washed with water (10 mL × 3). The white solid residue was collected as product (0.52 g, yield 89%). ^{13}C NMR (126 MHz, DMSO-d_6) δ 172.18, 169.90, 166.55, 164.42, 157.30 (d, *J* = 262.1 Hz), 138.5, 133.9, 123.4 (d, *J* = 21.1 Hz), 120.5 (d, *J* = 25.5 Hz), 117.5 (d, *J* = 12.6 Hz), 50.1, 31.5, 27.1, 21.5. EIS-MS: 313.28.

General Procedure for Synthesis of Compounds 18a-d. An appropriate carboxylic acid (1.2 mmol) was dissolved in CH_2Cl_2 (4 mL). $SOCl_2$ (1 mL) was added into the solution dropwise in 5 minutes. The resulting mixture was refluxed for 4 hour. Subsequently, the solvent and excessive $SOCl_2$ were removed under vacuum. The residue was dissolved in anhydrous tetrahydrofuran (6 mL) and **4** (260 mg, 1.1 mmol) was added into the solution. The suspension was refluxed overnight. Water (20 mL) was used to quench the reaction and followed by extraction with EtOAc (20 mL × 3). The organic lays were collected and washed with brine (40 mL) and dried over $MgSO_4$. The residue was purified with column chromatography by using CH_2Cl_2 as eluence. Product was obtained as slightly yellow solid.

N-(2-(2,6-Dioxopiperidin-3-yl)-1,3-dioxoisoindolin-4-yl)pent-4-ynamide (**18a**). ^{1}H NMR (500 MHz, DMSO-d_6) δ 11.17 (s, 1H), 9.80 (s, 1H), 8.46 (d, *J* = 8.4 Hz, 1H), 7.85 (t, *J* = 7.9 Hz, 1H), 7.64 (d, *J* = 7.2 Hz, 1H), 5.16 (dd, *J* = 12.9, 5.4 Hz, 1H), 2.95 – 2.86 (m, 1H), 2.83 (t, *J* = 2.5 Hz, 1H), 2.70 (t, *J* = 7.2 Hz, 2H), 2.65 – 2.54 (m, 2H), 2.49 (dd, *J* = 7.4, 2.6 Hz, 2H), 2.12 – 2.04 (m, 1H). ^{13}C NMR (126 MHz, DMSO) δ 173.3, 170.8, 170.3, 167.9, 167.1, 136.7, 136.5, 132.0, 127.0, 119.1, 117.7, 83.7, 72.2, 49.4, 35.8, 31.4, 22.5, 14.3. EIS-MS: 376.30.

N-(2-(2,6-Dioxopiperidin-3-yl)-1,3-dioxoisoindolin-4-yl)hex-5-ynamide (**18b**). ^{1}H NMR (500 MHz, DMSO-d_6) δ 11.17 (s, 1H), 9.77 (s, 1H), 8.43 (d, *J* = 8.4 Hz, 1H), 7.84 (t, *J* = 7.9 Hz, 1H), 7.63 (d, *J* = 7.2 Hz, 1H), 5.16 (dd, *J* = 12.9, 5.4 Hz, 1H), 2.95 – 2.87 (m, 1H), 2.84 (t, *J* = 2.6 Hz, 1H), 2.65 – 2.53 (m, 4H), 2.27 (td, *J* = 7.1, 2.6 Hz, 2H), 2.07 (dtd, *J* = 13.0, 5.3, 2.3 Hz, 1H), 1.83 – 1.77 (m, 2H). EIS-MS: 404.31.

N-(2-(2,6-Dioxopiperidin-3-yl)-1,3-dioxoisoindolin-4-yl)hept-6-ynamide (**18c**). ^{1}H NMR (500 MHz, DMSO-d_6) δ 11.17 (s, 1H), 9.73 (s, 1H), 8.50 – 8.41 (m, 1H), 7.84 (d, *J* = 7.0 Hz, 1H), 7.62 (d, *J* = 6.7 Hz, 1H), 5.16 (dd, *J* = 11.1, 5.9 Hz, 1H), 3.40 – 3.26 (m, 2H), 2.94 – 2.86

(m, 1H), 2.79 (s, 1H), 2.66 – 2.56 (m, 2H), 2.23 – 2.17 (m, 2H), 1.74 – 1.68 (m, 2H), 1.52 (s, 2H).

2-(4-Azidophenyl)-N-(2-(2,6-dioxopiperidin-3-yl)-1,3-dioxoisoindolin-4-yl)acetamide (**18d**). Compound **18d** was obtained from a two-step synthesis. The starting material 2-(4-Azidophenyl)acetic acid was prepared according to literature[7] using 2-(4-aminophenyl)acetic acid (0.50 mg, 3.3 mmol) as starting material to obtain 2-(4-azidophenyl)acetic acid as a light yellow-brown solid (0.55 g, 94%). Next, **7d** was synthesized by following the general procedure to provide 0.37 g yellow solid as product, yield 86%. ^{1}H NMR (500 MHz, DMSO-d_6) δ 11.17 (s, 1H), 9.83 (s, 1H), 8.47 (d, J = 8.4 Hz, 1H), 7.83 (t, J = 7.9 Hz, 1H), 7.62 (d, J = 7.3 Hz, 1H), 7.43 (d, J = 8.4 Hz, 2H), 7.12 (d, J = 8.4 Hz, 2H), 5.14 (dd, J = 12.9, 5.4 Hz, 1H), 3.86 (s, 2H), 2.92 (s, 1H), 2.59 (dd, J = 29.4, 13.6 Hz, 2H), 2.10 – 2.04 (m, 1H).

General Procedure for Synthesis of MIF degrader MD1-6. A azide (0.2 mmol, 1 equiv.) and a corresponding alkyne (0.2 mmol, 1 equiv.) were dissolved in MeOH (5 mL). To this solution, an aqueous solution (0.5 mL) of $CuSO_4{\cdot}5H_2O$ (10 mg, 0.04 mmol, 0.2 equiv.) and sodium ascorbate (20 mg, 0.1 mmol, 0.5 equiv.) was added. The suspension was stirred with argon at room temperature overnight. The resulting slurry mixture was filtrated and washed with MeOH (5 mL × 2). The filtrate was diluted with CH_2Cl_2 (20 mL) and washed with saturated NH_4Cl solution (20 mL) and brine (20 mL). The organic layer was collected and dried with $MgSO_4$ before evaporation. The crude mixture was purified with chromatography to provide the desired product.

2-(4-(((2-(2,6-Dioxopiperidin-3-yl)-1,3-dioxoisoindolin-4-yl)amino)methyl)-1H-1,2,3-triazol-1-yl)-N-(4-(7-hydroxy-2-oxo-2H-chromen-3-yl)phenyl)acetamide (**MD1**). Yield 51%. ^{1}H NMR (500 MHz, DMSO-d_6) δ 11.12 (s, 1H), 10.59 (s, 2H), 8.15 (d, J = 5.7 Hz, 1H), 8.09 (d, J = 5.7 Hz, 1H), 7.71 (d, J = 6.7 Hz, 2H), 7.66 – 7.58 (m, 4H), 7.24 (t, J = 7.2 Hz, 1H), 7.16

– 7.10 (m, 1H), 7.07 (t, *J* = 6.5 Hz, 1H), 6.83 (d, *J* = 7.7 Hz, 1H), 6.76 (s, 1H), 5.34 (s, 2H), 5.08 (d, *J* = 12.5 Hz, 1H), 4.66 (s, 2H), 2.96 – 2.83 (m, 1H), 2.65 – 2.53 (m, 2H), 2.12 – 1.93 (m, 1H). ^{13}C NMR (126 MHz, DMSO) δ 173.3, 170.5, 169.2, 167.8, 164.81, 161.6, 160.6, 155.2, 146.3, 144.9, 140.8, 138.7, 136.7, 132.6, 130.8, 130.4, 129.1, 125.1, 122.0, 119.4, 118.1, 113.9, 112.5, 111.4, 110.2, 102.2, 54.4, 52.7, 49.0, 38.0, 31.5. HRMS, calculated for $C_{33}H_{26}N_7O_8$ [M+H]$^+$: 648.1798, found 648.1848. Retention time 9.3 min. Purity 98.8%.

N-(2-(2,6-Dioxopiperidin-3-yl)-1,3-dioxoisoindolin-4-yl)-3-(1-(2-((4-(7-hydroxy-2-oxo-2H-chromen-3-yl)phenyl)amino)-2-oxoethyl)-1H-1,2,3-triazol-4-yl)propanamide (**MD2**). Yield 39%. ^{1}H NMR (500 MHz, DMSO-*d*$_6$) δ 11.17 (s, 1H), 10.60 (d, *J* = 9.1 Hz, 2H), 9.84 (s, 1H), 8.48 (d, *J* = 8.3 Hz, 1H), 8.15 (s, 1H), 7.97 (s, 1H), 7.84 (t, *J* = 7.9 Hz, 1H), 7.71 (d, *J* = 8.5 Hz, 2H), 7.67 – 7.61 (m, 3H), 7.60 (d, *J* = 8.5 Hz, 1H), 6.83 (d, *J* = 8.3 Hz, 1H), 6.76 (s, 1H), 5.32 (s, 2H), 5.16 (dd, *J* = 12.6, 5.3 Hz, 1H), 3.06 – 2.98 (m, 2H), 2.91 (m, 3H), 2.59 (m, 2H), 2.12 – 1.98 (m, 1H). ^{13}C NMR (126 MHz, DMSO) δ 173.2, 171.6, 170.3, 168.0, 167.1, 164.9, 161.6, 160.6, 155.2, 140.8, 138.7, 136.9, 136.6, 132.0, 130.8, 130.5, 129.4, 129.1, 127.0, 122.0, 119.3, 119.1, 118.8, 117.7, 113.9, 112.5, 102.1, 52.6, 49.4, 36.2, 31.4, 22.4, 21.2. HRMS, calculated for $C_{35}H_{28}O_9N_7$ [M+H]$^+$: 690.1904, found 690.1951. Retention time 9.0 min. Purity 96.5%.

N-(2-(2,6-Dioxopiperidin-3-yl)-1,3-dioxoisoindolin-4-yl)-4-(1-(2-((4-(7-hydroxy-2-oxo-2H-chromen-3-yl)phenyl)amino)-2-oxoethyl)-1H-1,2,3-triazol-4-yl)butanamide (**MD3**). Yield 51%. ^{1}H NMR (500 MHz, DMSO-*d*$_6$) δ 11.17 (s, 1H), 10.60 (s, 2H), 9.77 (s, 1H), 8.54 – 8.41 (m, 1H), 8.16 (s, 1H), 7.96 (s, 1H), 7.84 (t, *J* = 7.9 Hz, 1H), 7.71 (d, *J* = 8.6 Hz, 2H), 7.68 – 7.59 (m, 4H), 6.83 (d, *J* = 8.6 Hz, 1H), 6.76 (s, 1H), 5.32 (s, 2H), 5.16 (dd, *J* = 12.8, 5.4 Hz, 1H), 2.95 – 2.85 (m, 1H), 2.75 (t, *J* = 7.4 Hz, 2H), 2.65 – 2.53 (m, 4H), 2.12 – 2.05 (m, 1H), 1.99 (p, *J* = 6.8 Hz, 2H). ^{13}C NMR (126 MHz, DMSO) δ 173.3, 172.2, 170.3, 168.1, 167.2, 165.0, 161.6, 160.6, 155.2, 146.6, 140.8, 138.7, 136.9, 136.5, 131.9, 130.8, 130.4, 129.3, 127.0,

124.2, 122.0, 119.2, 118.9, 117.7, 113.9, 112.5, 102.2, 52.6, 49.4, 36.4, 31.4, 25.19, 2.88, 22.5. HRMS, calculated for $C_{36}H_{30}O_9N_7$ [M+H]$^+$: 704.2060, found 704.2098. Retention time 9.3 min. Purity 97%.

N-(2-(2,6-Dioxopiperidin-3-yl)-1,3-dioxoisoindolin-4-yl)-5-(1-(2-((4-(7-hydroxy-2-oxo-2H-chromen-3-yl)phenyl)amino)-2-oxoethyl)-1H-1,2,3-triazol-4-yl)pentanamide (**MD4**). Yield 57%. ^{1}H NMR (500 MHz, DMSO-d_6) δ 11.17 (s, 1H), 10.62 (s, 1H), 10.60 (s, 1H), 9.74 (s, 1H), 8.48 (d, *J* = 8.3 Hz, 1H), 8.15 (s, 1H), 7.92 (s, 1H), 7.83 (t, *J* = 7.8 Hz, 1H), 7.70 (d, *J* = 8.6 Hz, 2H), 7.65 (d, *J* = 8.6 Hz, 2H), 7.61 (dd, *J* = 11.2, 8.0 Hz, 2H), 6.83 (d, *J* = 8.5 Hz, 1H), 6.76 (s, 1H), 5.31 (s, 2H), 5.16 (dd, *J* = 12.7, 5.2 Hz, 1H), 2.90 (td, *J* = 16.9, 15.1, 6.5 Hz, 1H), 2.75 – 2.53 (m, 6H), 2.13 – 2.02 (m, 1H), 1.70 (s, 4H). ^{13}C NMR (126 MHz, DMSO) δ 173.2, 172.4, 170.3, 168.7, 168.1, 167.1, 161.5, 160.6, 155.2, 151.5, 140.6, 139.2, 137.0, 136.4, 131.9, 130.4, 130.3, 129.2, 129.0, 126.7, 122.1, 119.2, 119.0, 117.4, 113.8, 112.5, 102.2, 49.36, 45.8, 37.0, 36.6, 31.4, 28.9, 25.1, 22.5. HRMS, calculated for $C_{37}H_{32}O_9N_7$ [M+H]$^+$: 718.2217, found 718.2257. Retention time 9.5 min. Purity 95.2%.

N-(2-(2,6-Dioxopiperidin-3-yl)-1,3-dioxoisoindolin-4-yl)-5-(1-(3-((4-(7-hydroxy-2-oxo-2H-chromen-3-yl)phenyl)amino)-3-oxopropyl)-1H-1,2,3-triazol-4-yl)pentanamide (**MD5**). Yield 31%. ^{1}H NMR (500 MHz, DMSO-d_6) δ 11.17 (s, 1H), 10.59 (s, 1H), 10.17 (s, 1H), 9.69 (s, 1H), 8.46 (d, *J* = 8.4 Hz, 1H), 8.11 (s, 1H), 7.85 (s, 1H), 7.81 (t, *J* = 7.9 Hz, 1H), 7.66 (d, *J* = 8.7 Hz, 2H), 7.63 – 7.56 (m, 4H), 6.81 (d, *J* = 8.4 Hz, 1H), 6.75 (s, 1H), 5.15 (dd, *J* = 12.8, 5.4 Hz, 1H), 4.62 (t, *J* = 6.6 Hz, 2H), 2.99 (t, *J* = 6.6 Hz, 2H), 2.94 – 2.83 (m, 1H), 2.67 – 2.53 (m, 4H), 2.48 (d, *J* = 4.5 Hz, 2H), 2.08 (dd, *J* = 9.6, 6.0 Hz, 1H), 1.64 (s, 4H). ^{13}C NMR (126 MHz, DMSO) δ 173.2, 172.4, 170.3, 168.8, 168.1, 167.1, 161.5, 160.5, 155.2, 140.6, 139.2, 137.0, 136.5, 131.9, 130.3, 129.1, 126.7, 122.8, 122.1, 119.1, 119.1, 118.7, 117.4, 113.8, 113.5, 112.5, 102.1, 49.4, 45.8, 37.0, 36.6, 31.4, 28.9, 25.1, 24.7, 22.5. HRMS, calculated for $C_{38}H_{34}N_7O_9$ [M+H]$^+$: 732.2373, found 732.2432. Retention time 9.0 min. Purity 95.3%.

4-(1-(4-(2-((2-(2,6-Dioxopiperidin-3-yl)-1,3-dioxoisoindolin-4-yl)amino)-2-oxoethyl)phenyl)-1H-1,2,3-triazol-4-yl)-N-(4-(7-hydroxy-2-oxo-2H-chromen-3-yl)phenyl)butanamide (**MD6**). Yield 54%. ^{1}H NMR (500 MHz, DMSO-d_6) δ 11.17 (s, 1H), 10.59 (s, 1H), 10.05 (s, 1H), 9.92 (s, 1H), 8.63 (s, 1H), 8.47 (d, *J* = 8.3 Hz, 1H), 8.12 (s, 1H), 7.88 (d, *J* = 8.2 Hz, 2H), 7.84 (t, *J* = 7.8 Hz, 1H), 7.69 – 7.57 (m, 8H), 6.82 (d, *J* = 8.2 Hz, 1H), 6.75 (s, 1H), 5.14 (dd, *J* = 12.9, 5.4 Hz, 1H), 3.96 (s, 2H), 2.96 – 2.84 (m, 1H), 2.79 (t, *J* = 6.8 Hz, 2H), 2.66 – 2.53 (m, 2H), 2.45 (t, *J* = 7.1 Hz, 2H), 2.05 (dt, *J* = 14.7, 8.3 Hz, 3H). ^{13}C NMR (126 MHz, DMSO) δ 173.2, 171.5, 170.2, 168.0, 167.1, 161.5, 160.6, 155.2, 148.1, 140.6, 140.5, 139.6, 139.6, 136.7, 136.6, 136.2, 135.6, 132.0, 131.4, 130.3, 130.0, 129.0, 126.8, 122.2, 120.7, 120.4, 119.1, 117.7, 113.8, 112.5, 102.2, 49.4, 43.1, 36.2, 31.4, 25.1, 25.1, 22.4. HRMS, calculated for $C_{42}H_{34}O_9N_7$ $[M+H]^+$: 780.2373, found 780.2423. Retention time 10 min. Purity 96.3%.

General Procedure for Synthesis of MIF degrader MD7-9 and 13-15. To a round-bottom flask, an appropriate carboxylic acid (0.2 mmol, 1 equiv.) was added into CH_2Cl_2 (6 mL). Then, EDCI·HCl (50 mg, 0.25 mmol) and HOBt (30 mg, 0.2 mmol) was added and stirred for 5 minutes prior to being added to the corresponding amine (0.2 mmol, 1 equiv.). The mixture was stirred at room temperature for 12 hours and reaction was monitored with TLC. After the completion of the reaction, the solvent was removed under reduced-pressure. The residue was dissolved in THF (6 mL) and a solution of TBAF (0.2 mL, 0.2 mmol, 1M in THF) was added to the residue. The mixture was reacted at room temperature for 2 hours prior to being diluted with CH_2Cl_2 (20 mL). The resulting suspension was washed with water (20 mL) and brine (20 mL). The organic layer was collected and dried with $MgSO_4$ before evaporation. The crude mixture was purified with chromatography using CH_2Cl_2 : MeOH 40:1 as eluence.

5-((2-(2,6-Dioxopiperidin-3-yl)-1,3-dioxoisoindolin-4-yl)amino)-N-(4-(7-hydroxy-2-oxo-2H-benzo[e][1,3]oxazin-3(4H)-yl)phenyl)pentanamide (**MD7**). Yield 49%. ^{1}H NMR (500

MHz, DMSO-d_6) δ 11.09 (s, 1H), 10.01 (s, 1H), 9.78 (s, 1H), 7.62 (s, 2H), 7.59 – 7.53 (m, 1H), 7.35 (d, *J* = 7.5 Hz, 2H), 7.13 – 7.04 (m, 2H), 7.01 (s, 1H), 6.59 (d, *J* = 5.1 Hz, 2H), 6.47 (s, 1H), 5.05 (dd, *J* = 8.5, 4.0 Hz, 1H), 4.73 (s, 2H), 3.45 – 3.35 (m, 2H), 2.95 – 2.77 (m, 1H), 2.63 – 2.51 (m, 2H), 2.40 – 2.31 (m, 2H), 2.06 – 1.96 (m, 1H), 1.71 – 1.53 (m, 4H). ^{13}C NMR (126 MHz, DMSO) δ 173.2, 171.6, 170.5, 169.4, 167.8, 158.4, 150.5, 150.1, 146.9, 138.3, 137.4, 136.7, 132.7, 127.0, 126.4, 119.9, 117.7, 112.1, 110.9, 109.6, 109.3, 102.6, 50.1, 49.0, 42.0, 36.5, 31.4, 28.8, 22.9, 22.6. HRMS, calculated for $C_{32}H_{30}O_8N_5$ [M+H]$^+$: 612.2050, found 612.2060. Retention time 9.5 min. Purity 95.3%.

5-((2-(2,6-Dioxopiperidin-3-yl)-1,3-dioxoisoindolin-4-yl)amino)-N-(4-(7-hydroxy-2-oxo-2H-benzo[e][1,3]oxazin-3(4H)-yl)benzyl)pentanamide (**MD8**). Yield 42%. ^{1}H NMR (500 MHz, DMSO-d_6) δ 11.10 (s, 1H), 9.81 (s, 1H), 8.38 (s, 1H), 7.62 – 7.55 (m, 1H), 7.38 (d, *J* = 8.5 Hz, 2H), 7.30 (d, *J* = 8.4 Hz, 2H), 7.10 (d, *J* = 8.6 Hz, 1H), 7.07 (d, *J* = 8.4 Hz, 1H), 7.02 (d, *J* = 7.0 Hz, 1H), 6.62 – 6.55 (m, 2H), 6.49 (d, *J* = 2.3 Hz, 1H), 5.06 (dd, *J* = 12.7, 5.4 Hz, 1H), 4.74 (s, 2H), 4.28 (d, *J* = 5.9 Hz, 2H), 3.33 (t, *J* = 6.5 Hz, 2H), 2.88 (td, *J* = 16.4, 13.5, 5.3 Hz, 1H), 2.64 – 2.52 (m, 2H), 2.21 (t, *J* = 7.0 Hz, 2H), 2.09 – 1.98 (m, 1H), 1.61 (dq, *J* = 11.6, 6.2 Hz, 4H). ^{13}C NMR (126 MHz, DMSO) δ 173.3, 172.5, 170.58, 169.4, 167.8, 158.3, 150.5, 150.0, 146.9, 141.1, 138.8, 136.8, 132.7, 128.2, 127.5, 125.9, 117.6, 112.1, 110.8, 109.5, 109.3, 102.6, 55.4, 49.9, 49.0, 42.0, 35.4, 31.4, 28.8, 23.1, 22.6. HRMS, calculated for $C_{33}H_{32}O_8N_5$ [M+H]$^+$: 626.2206, found 626.2221. Retention time 9.1 min. Purity 98%.

7-((2-(2,6-Dioxopiperidin-3-yl)-1,3-dioxoisoindolin-4-yl)amino)-N-(4-(7-hydroxy-2-oxo-2H-benzo[e][1,3]oxazin-3(4H)-yl)phenyl)heptanamide (**MD9**). Yield 61%. ^{1}H NMR (500 MHz, DMSO-d_6) δ 11.08 (s, 1H), 9.95 (s, 1H), 9.75 (s, 1H), 7.65 – 7.54 (m, 3H), 7.35 (d, *J* = 8.4 Hz, 2H), 7.11 – 6.99 (m, 3H), 6.59 (d, *J* = 8.1 Hz, 1H), 6.55 – 6.49 (m, 1H), 6.48 (s, 1H), 5.05 (dd, *J* = 11.6, 4.7 Hz, 1H), 4.73 (s, 2H), 3.30 – 3.25 (m, 2H), 2.93 – 2.81 (m, 1H), 2.64 – 2.51 (m, 2H), 2.36 – 2.26 (m, 2H), 2.08 – 1.96 (m, 1H), 1.60 (dd, *J* = 17.0, 4.1 Hz, 4H), 1.42 –

1.31 (m, 4H). ^{13}C NMR (126 MHz, DMSO) δ 172.7, 171.3, 170.0, 168.9, 167.3, 157.8, 150.0, 149.6, 146.4, 137.8, 136.8, 136.3, 132.2, 126.5, 125.9, 119.3, 117.1, 111.5, 110.3, 109.0, 108.8, 102.1, 49.6, 48.5, 41.8, 36.3, 30.9, 28.5, 28.3, 26.1, 25.0, 22.1. HRMS, calculated for $C_{34}H_{34}O_8N_5$ [M+H]$^+$: 640.2363, found 640.2373. Retention time 10.3 min. Purity 98%.

8-((2-(2,6-Dioxopiperidin-3-yl)-1,3-dioxoisoindolin-4-yl)amino)-N-(4-(7-hydroxy-2-oxo-2H-benzo[e][1,3]oxazin-3(4H)-yl)phenyl)octanamide (**MD13**). Yield 43%. ^{1}H NMR (500 MHz, DMSO-d_6) δ 11.09 (s, 1H), 9.96 (s, 1H), 9.77 (s, 1H), 7.63 (d, *J* = 8.8 Hz, 2H), 7.61 – 7.56 (m, 1H), 7.36 (d, *J* = 8.8 Hz, 2H), 7.12 – 7.06 (m, 2H), 7.03 (d, *J* = 7.0 Hz, 1H), 6.60 (dd, *J* = 8.3, 2.3 Hz, 1H), 6.54 (t, *J* = 5.9 Hz, 1H), 6.48 (d, *J* = 2.3 Hz, 1H), 5.06 (dd, *J* = 12.7, 5.4 Hz, 1H), 4.73 (s, 2H), 3.29 (d, *J* = 6.9 Hz, 2H), 2.93 – 2.84 (m, 1H), 2.65 – 2.52 (m, 2H), 2.32 (t, *J* = 7.4 Hz, 2H), 2.06 – 1.98 (m, 1H), 1.57 (d, *J* = 7.9 Hz, 4H), 1.35 (s, 6H). ^{13}C NMR (126 MHz, DMSO) δ 173.3, 171.8, 170.6, 169.4, 167.8, 158.3, 150.5, 150.1, 146.9, 138.4, 137.3, 132.7, 127.1, 126.5, 126.4, 119.9, 119.7, 117.6, 112.0, 110.9, 109.5, 109.3, 58.0, 50.1, 49.0, 42.3, 31.4, 29.1, 29.0, 26.7, 23.5, 19.7, 14.0. HRMS, calculated for $C_{35}H_{36}O_8N_5$ [M+H]$^+$: 654,2558, found 654,2557. Retention time 12 min. Purity 98.9%.

10-((2-(2,6-Dioxopiperidin-3-yl)-1,3-dioxoisoindolin-4-yl)amino)-N-(4-(7-hydroxy-2-oxo-2H-benzo[e][1,3]oxazin-3(4H)-yl)phenyl)decanamide (**MD14**). Yield 30%. ^{1}H NMR (500 MHz, DMSO-d_6) δ 11.11 (s, 1H), 9.98 (s, 1H), 9.79 (s, 1H), 7.63 (d, *J* = 8.8 Hz, 2H), 7.61 – 7.56 (m, 1H), 7.36 (d, *J* = 8.8 Hz, 2H), 7.09 (t, *J* = 9.2 Hz, 2H), 7.02 (d, *J* = 7.0 Hz, 1H), 6.60 (dd, *J* = 8.3, 2.3 Hz, 1H), 6.54 (t, *J* = 6.0 Hz, 1H), 6.48 (d, *J* = 2.3 Hz, 1H), 5.06 (dd, *J* = 12.8, 5.5 Hz, 1H), 4.73 (s, 2H), 3.31 – 3.26 (m, 2H), 2.97 – 2.84 (m, 1H), 2.69 – 2.54 (m, 2H), 2.31 (t, *J* = 7.4 Hz, 2H), 2.05 – 2.00 (m, 1H), 1.59 – 1.55 (m, 4H), 1.36 – 1.30 (m, 10H). ^{13}C NMR (126 MHz, DMSO) δ 173.3, 171.8, 170.6, 169.4, 167.8, 158.3, 150.5, 150.1, 146.9, 138.4, 137.3, 132.6, 127.1, 126.4, 119.9, 119.7, 117.7, 112.0, 110.8, 109.5, 109.3, 102.6, 58.0, 50.1,

49.0, 42.3, 36.9, 31.4, 29.2, 29.0, 26.8, 25.5, 23.5, 22.6, 19.7. HRMS, calculated for $C_{37}H_{40}O_8N_5$ $[M+H]^+$: 682,2871, found 682,2870. Retention time 8.3 min. Purity 93.5%.

N-(4-(7-Hydroxy-2-oxo-2H-benzo[e][1,3]oxazin-3(4H)-yl)phenyl)-8-((2-(1-methyl-2,6-dioxopiperidin-3-yl)-1,3-dioxoisoindolin-4-yl)amino)octanamide (**MD15**). Yield 54%. ^{1}H NMR (500 MHz, DMSO-d_6) δ 9.99 (s, 1H), 9.81 (s, 1H), 7.63 (d, *J* = 8.8 Hz, 2H), 7.61 – 7.56 (m, 1H), 7.36 (d, *J* = 8.8 Hz, 2H), 7.09 (dd, *J* = 11.7, 8.6 Hz, 2H), 7.03 (d, *J* = 7.0 Hz, 1H), 6.60 (dd, *J* = 8.3, 2.2 Hz, 1H), 6.56 (t, *J* = 5.7 Hz, 1H), 6.48 (d, *J* = 2.2 Hz, 1H), 5.13 (dd, *J* = 13.0, 5.4 Hz, 1H), 4.73 (s, 2H), 3.29 (t, *J* = 6.5 Hz, 2H), 3.02 (s, 3H), 3.00 – 2.89 (m, 1H), 2.62 – 2.52 (m, 2H), 2.32 (t, *J* = 7.3 Hz, 2H), 2.08 – 2.01 (m, 1H), 1.65 – 1.55 (m, 4H), 1.34 (dd, *J* = 10.3, 5.6 Hz, 6H). ^{13}C NMR (126 MHz, DMSO) δ 172.3, 171.8, 170.3, 169.4, 167.7, 158.3, 150.5, 150.1, 146.9, 138.4, 137.3, 136.9, 132.6, 129.8, 127.1, 126.4, 119.9, 119.7, 117.7, 110.9, 109.4, 109.3, 50.0, 49.5, 42.3, 36.8, 34.0, 31.6, 30.8, 29.1, 29.0, 27.1, 26.7, 25.5. HRMS, calculated for $C_{36}H_{38}O_8N_5$ $[M+H]^+$: 668,2715, found 668,2715. Retention time 10.1 min. Purity 99%.

General Procedure for Synthesis of MIF PROTACs MD10-12. The corresponding azide (0.2 mmol, 1 equiv.) and alkyne (0.2 mmol, 1 equiv.) were dissolved in MeOH (5 mL). To this solution, an aqueous solution (0.5 mL) of $CuSO_4 \cdot 5H_2O$ (10 mg, 0.04 mmol, 0.2 equiv.) and sodium ascorbate (20 mg, 0.1 mmol, 0.5 equiv.) were added. The suspension was stirred under argon atmosphere at room temperature overnight. The resulting slurry mixture was filtered and washed with MeOH (5 mL × 2). The filtrate was evaporated and the residue was dissolved in THF (6 mL). Subsequently, a solution of TBAF (0.2 mL, 0.2 mmol, 1 M in THF) was added to the solution. The mixture was reacted at room temperature for 2 hours prior to being diluted with CH_2Cl_2 (20 mL). The resulting suspension was washed with water (20 mL) and brine (20 mL). The organic layer was collected and dried with $MgSO_4$ before evaporation. The crude

mixture was purified with chromatography using CH_2Cl_2 : MeOH 40:1 (*v/v*) as eluent to provide the desired products.

3-(4-(((2-(2,6-Dioxopiperidin-3-yl)-1,3-dioxoisoindolin-4-yl)amino)methyl)-1H-1,2,3-triazol-1-yl)-N-(4-(7-hydroxy-2-oxo-2H-benzo[e][1,3]oxazin-3(4H)-yl)phenyl)propanamide (**MD10**). Yield 62%. ^{1}H NMR (500 MHz, DMSO-d_6) δ 11.11 (s, 1H), 10.14 (s, 1H), 9.79 (s, 1H), 8.01 (s, 1H), 7.58 (d, *J* = 8.3 Hz, 2H), 7.53 – 7.47 (m, 1H), 7.37 (d, *J* = 8.2 Hz, 2H), 7.16 (d, *J* = 8.2 Hz, 1H), 7.07 (dd, *J* = 21.4, 7.0 Hz, 3H), 6.61 (d, *J* = 7.7 Hz, 1H), 6.49 (s, 1H), 5.07 (dd, *J* = 12.0, 5.6 Hz, 1H), 4.74 (s, 2H), 4.68 – 4.55 (m, 4H), 3.02 – 2.92 (m, 2H), 2.88 (d, *J* = 13.9 Hz, 1H), 2.64 – 2.52 (m, 2H), 2.06 – 2.01 (m, 1H). ^{13}C NMR (126 MHz, DMSO) δ 172.8, 170.0, 168.7, 168.1, 167.3, 157.8, 150.0, 149.6, 145.8, 137.3, 137.1, 136.1, 132.1, 126.6, 125.9, 123.2, 119.5, 117.5, 111.6, 110.9, 109.6, 109.6, 108.8, 102.1, 49.5, 48.6, 45.5, 37.5, 36.3, 31.0, 22.1. HRMS, calculated for $C_{33}H_{29}O_8N_8$ $[M+H]^+$: 665.2064, found 665.2064. Retention time 8.3 min. Purity 98.5%.

N-(2-(2,6-Dioxopiperidin-3-yl)-1,3-dioxoisoindolin-4-yl)-4-(1-(3-((4-(7-hydroxy-2-oxo-2H-benzo[e][1,3]oxazin-3(4H)-yl)phenyl)amino)-3-oxopropyl)-1H-1,2,3-triazol-4-yl)butanamide (**MD11**). Yield 47%. ^{1}H NMR (500 MHz, DMSO-d_6) δ 11.17 (s, 1H), 10.17 (s, 1H), 9.81 (s, 1H), 9.72 (s, 1H), 8.46 (d, *J* = 8.3 Hz, 1H), 7.88 (s, 1H), 7.83 (t, *J* = 7.8 Hz, 1H), 7.66 – 7.53 (m, 3H), 7.37 (d, *J* = 8.6 Hz, 2H), 7.07 (d, *J* = 8.3 Hz, 1H), 6.60 (d, *J* = 8.2 Hz, 1H), 6.48 (s, 1H), 5.16 (dd, *J* = 12.7, 5.3 Hz, 1H), 4.72 (s, 2H), 4.63 (t, *J* = 6.4 Hz, 2H), 2.99 (t, *J* = 6.8 Hz, 2H), 2.95 – 2.83 (m, 1H), 2.72 – 2.54 (m, 4H), 2.08 (d, *J* = 12.9 Hz, 1H), 1.95 (q, *J* = 7.7 Hz, 2H). ^{13}C NMR (126 MHz, DMSO) δ 173.2, 172.2, 170.2, 168.7, 168.1, 167.1, 158.3, 150.5, 150.0, 146.6, 137.9, 137.6, 137.0, 136.6, 131.9, 127.0, 126.9, 126.4, 122.9, 120.0, 118.8, 117.6, 112.1, 109.3, 102.6, 50.0, 49.4, 45.9, 36.9, 36.3, 31.4, 25.1, 24.8, 22.5. HRMS, calculated for $C_{36}H_{33}N_8O_9$ $[M+H]^+$: 721.2326, found 721.2385. Retention time 8.3 min. Purity 97.3%.

N-(2-(2,6-Dioxopiperidin-3-yl)-1,3-dioxoisoindolin-4-yl)-2-(4-(4-((2-(4-(7-hydroxy-2-oxo-2H-benzo[e][1,3]oxazin-3(4H)-yl)phenyl)acetamido)methyl)-1H-1,2,3-triazol-1-yl)phenyl)acetamide (**MD12**). Yield 72%. ^{1}H NMR (500 MHz, DMSO-d_6) δ 11.17 (s, 1H), 9.94 (s, 1H), 9.81 (s, 1H), 8.69 (s, 1H), 8.63 (s, 1H), 8.47 (d, J = 8.1 Hz, 1H), 7.86 (dd, J = 25.2, 7.2 Hz, 3H), 7.62 (dd, J = 17.1, 7.1 Hz, 3H), 7.37 (q, J = 7.0 Hz, 4H), 7.07 (d, J = 7.9 Hz, 1H), 6.60 (d, J = 7.9 Hz, 1H), 6.49 (s, 1H), 5.21 – 5.08 (m, 1H), 4.76 (s, 2H), 4.41 (s, 2H), 3.97 (s, 2H), 3.52 (s, 2H), 2.90 (m, 10.5 Hz, 1H), 2.59 (m, 2H), 2.11 – 2.03 (m, 1H). ^{13}C NMR (126 MHz, DMSO) δ 173.2, 170.4, 170.3, 170.2, 168.0, 167.1, 158.3, 150.5, 150.0, 146.3, 140.9, 136.7, 136.6, 136.0, 135.9, 135.3, 132.0, 131.4, 130.1, 127.0, 126.9, 125.8, 121.5, 120.5, 119.1, 117.8, 112.1, 109.3, 102.6, 49.9, 49.4, 43.1, 42.1, 34.8, 31.4, 22.4. HRMS, calculated for $C_{40}H_{33}O_9N_8$ $[M+H]^+$: 769.2326, found 769.2378. Retention time 9.4 min. Purity 95.1%.

S2. MIF Tautomerase activity IC_{50} study

The protocol for measuring inhibition of MIF tautomerase enzyme activity and enzyme kinetics was described previously.[8] Briefly, 180 µL of a 500 nM MIF solution in boric acid buffer (435 mM, pH 6.2) was mixed with 10 µL of a 20 mM EDTA solution in demiwater and 10 µL of a solution of the desired compound dissolved in DMSO or blanc DMSO. This mixture was pre-incubated at room temperature for 10 min. Next, 50 µL of this mixture was mixed with 50 µL of a 1 mM 4-HPP solution in ammonium acetate buffer (50 mM, pH 6.0). Subsequently, MIF tautomerase activity was monitored by measuring the increase of UV absorbance at 306 nm over time. MIF tautomerase activity in the presence a blank DMSO dilution was set to 100% enzyme activity. Non-catalyzed conversion of the substrate in absence of MIF was set to 0%. Data from the first three minutes were used to calculate the initial velocities. All experiments were repeated three times and calculations were performed with the program GraphPad Prism.

Table S1. 4-HPP conversion assay results of MIF PROTACs

<table>
<tr>
<td>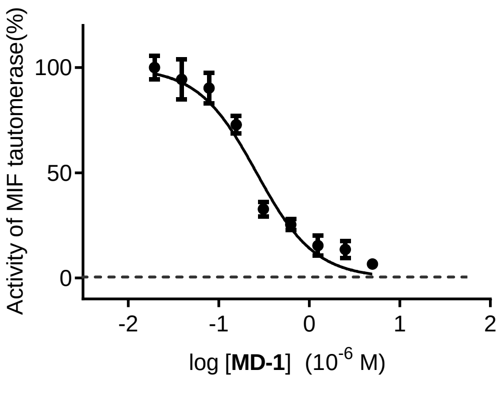
</td>
<td>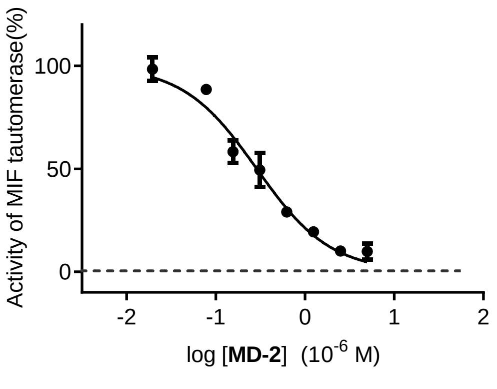
</td>
</tr>
<tr>
<td>MD1: IC_{50} (nM)=262±35</td>
<td>MD2: IC_{50} (nM)=288±25</td>
</tr>
</table>

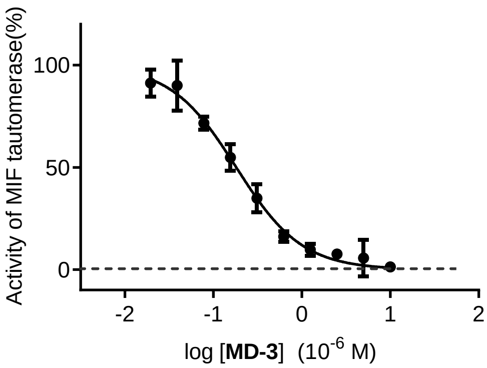

MD3: IC_{50} (nM)=182±16

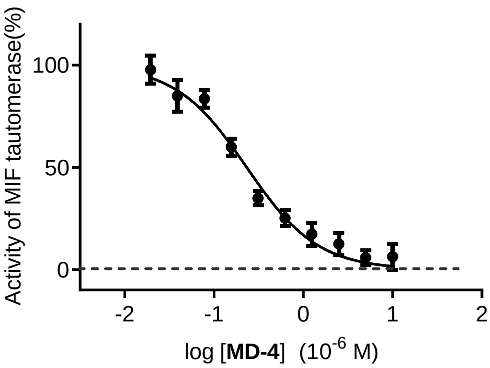

MD4: IC_{50} (nM)=233±26

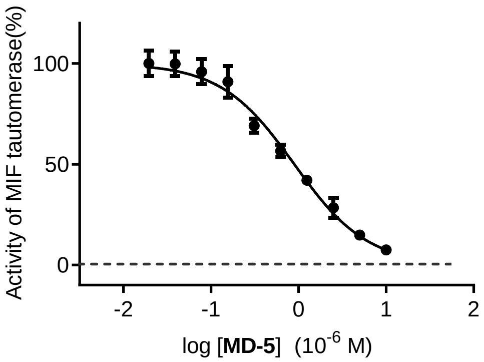

MD5: IC_{50} (nM)=895±82

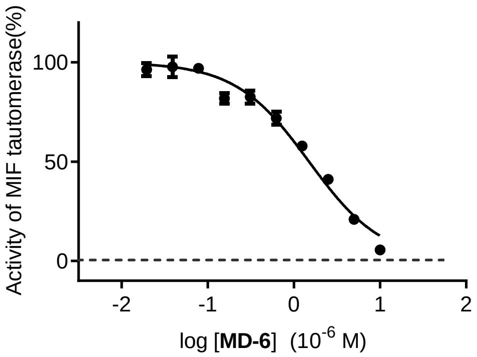

MD6: IC_{50} (nM)=1499±130

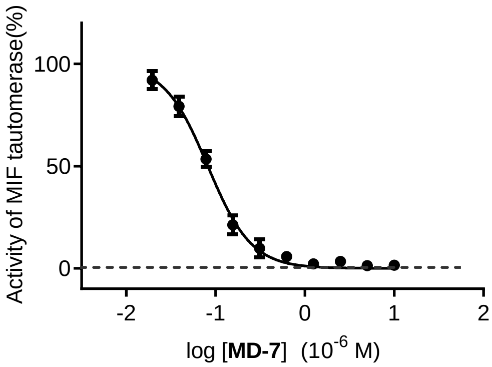

MD7: IC_{50} (nM)=82±4

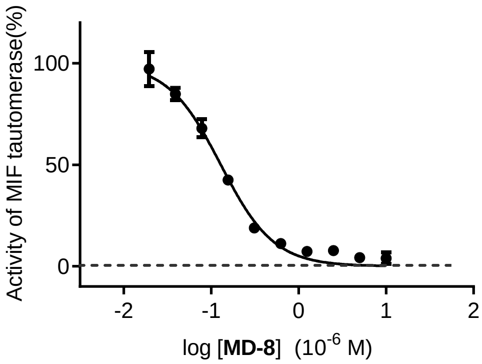

MD8: IC_{50} (nM)=129±8

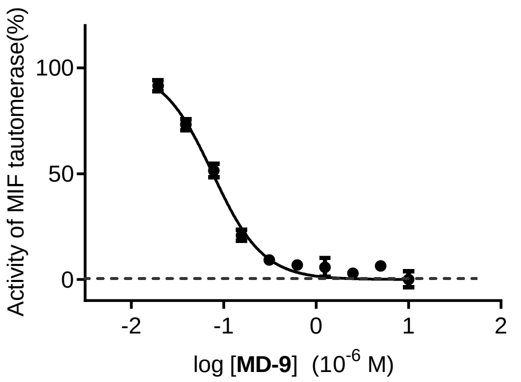

MD9: IC_{50} (nM)=76±4

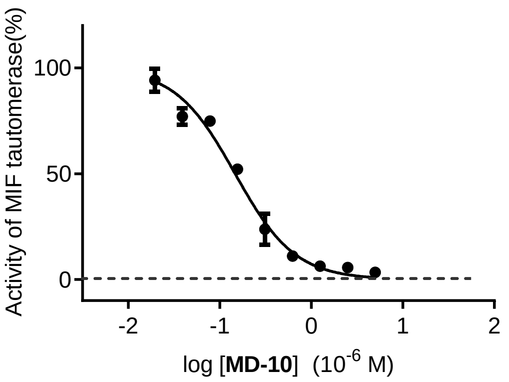

MD10: IC_{50} (nM)=147±13

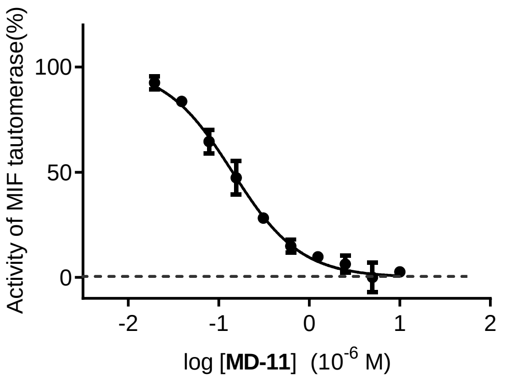

MD11: IC_{50} (nM)=143±11

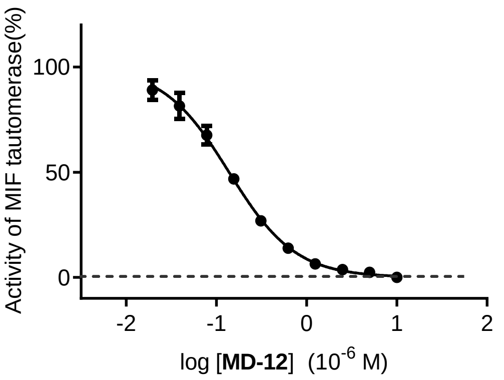

MD12:IC_{50} (nM)=138±6

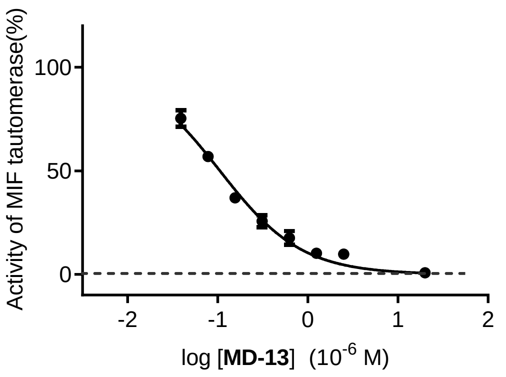

MD13: IC_{50} (nM)=106±7

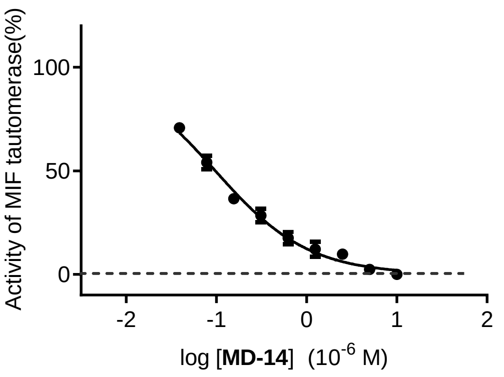

MD14: IC_{50} (nM)=97±6

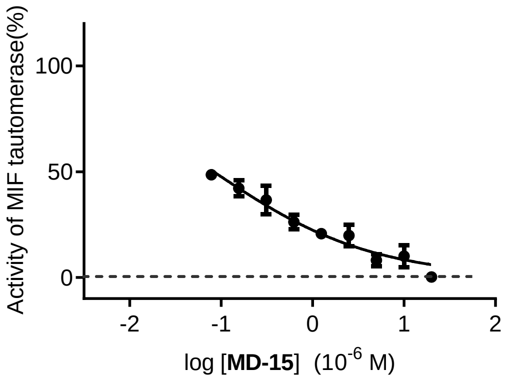	
**MD15**: IC_{50} (nM)=83±18	

S3. ELISA screening of MIF protein level on MIF-directed PROTACs

Cell culture. Human A549 lung cancer cells (ATCCCCL-185) or HEK293 kidney cancer cells (ATCCCRL-1573) were cultured in RPMI 1640 Medium (GibcoTM #61870-010) containing 10% (v/v) fetal bovine serum (FBS), 100 U/mL penicillin/streptomycin (GibcoTM#10378016) at 37°C with 5% CO_2 in humidified air.

ELISA. 7×10^5 A549 cells per well were plated in 6-well plates. The next day, cells were treated with a solution of compounds or the vehicle for 12 h. Subsequently, the culture medium was removed and cells were washed twice with cold PBS and collected after trypsin (0.5 mL) degestion. Cells were then lyzed in lysis buffer (150 mM NaCl, 50 mM Hepes pH 7.5, 5 mM EDTA, 0.1% *(w/v)* NP40) containing protease inhibitor cocktail (Roche, #11836153001) and resuspended vigourously. The supernatant was collected after spinning down at 16 000g at 4 °C for 30 min. The total protein concentration was determined with Pierce™ BCA protein assay kit (ThermoFisher, #23225). The protein concentration was normalized between samples. The relative MIF levels from 1μg total protein were determined by ELISA with the human MIF DuoSet ELISA kit according to the instructions of the manufacturer (R&D systems, #DY289).

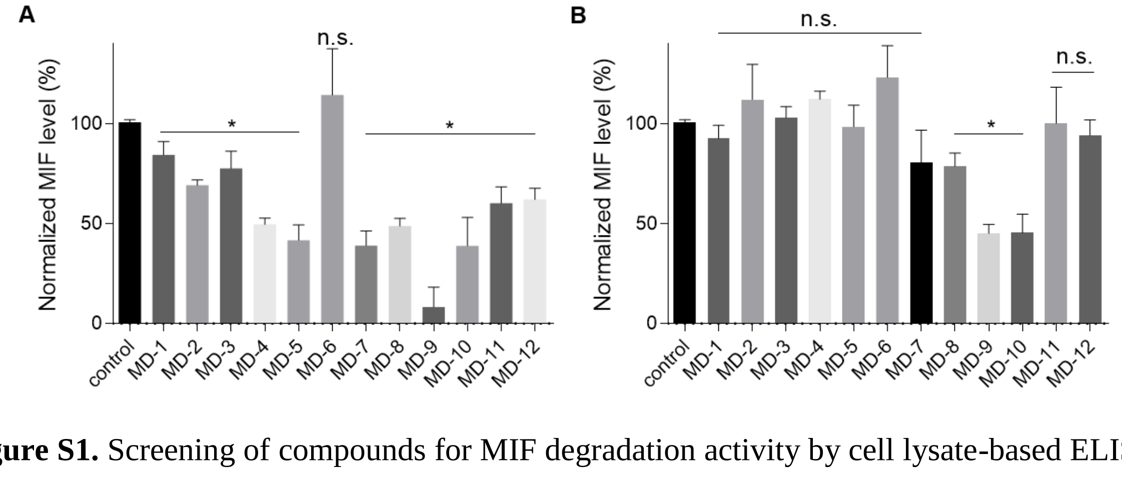

Figure S1. Screening of compounds for MIF degradation activity by cell lysate-based ELISA. A549 cells were treated with compounds at (A) 20 or (B) 2 μM for 12 h. Data was normalized

to vehicle (DMSO)-treated group and the bar graph represented as a mean of relative MIF protein level (n = 3) with ±SD as the error bar. Not significant (n.s.) $P > 0.05$, * $P \leq 0.05$.

Table S2. Table Caption MIF-directed PROTACs in which a 7-hydroxycoumarin MIF binding ligand or a 7-hydroxy-3,4-dihydrobenzoxazin-2-one MIF binding ligand is connected to pomalidomide as CRBN ligand.

Group **1**

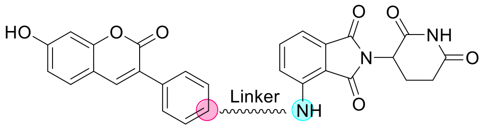

ID	Linker	K_i (nM) [a]	degradation (%) [b]	
			20 µM	2 µM
**2**	--	370±30	--	--
**MD1**	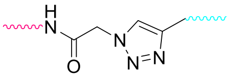	175±23	16±7	n.s.
**MD2**	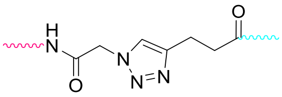	192±17	31±3	n.s.
**MD3**	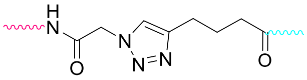	121±11	22±9	n.s.
**MD4**	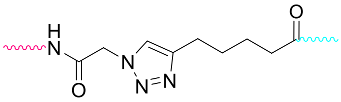	155±17	50±3	n.s.
**MD5**	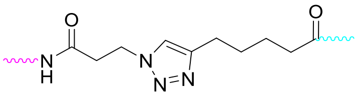	597±55	58±8	n.s.
**MD6**	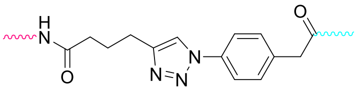	999±87	n.s.	n.s.

Group **2**

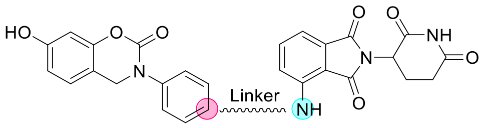

**3**	--	103±10	--	--
**MD7**	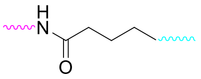	55±3	61±7	n.s.
**MD8**	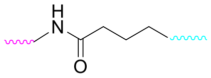	86±5	51±4	21±7
**MD9**	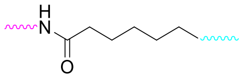	51±3	92±10	55±4
**MD10**	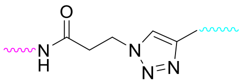	98±9	61±14	55±9
**MD11**	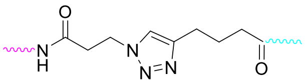	95±7	40±8	n.s.
**MD12**	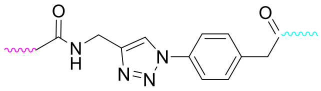	92±4	38±6	n.s.

[a] Measured by MIF catalyzed 4-HPP tautomerization assay using the method previously reported by our group (n=3).[43] [b] Degradation percentage of MIF by putative MIF degraders. A549 cells were treated with compounds at 20 or 2 μM for 12 h. Cell lysates were standardized for protein concentration. ELISA assay was applied to measure the protein level of MIF, which was normalized to vehicle (DMSO)-treated group. Data were represented as mean ± SD (n = 3). No significant degradation (n.s.) P > 0.05 *vs* vehicle control.

S4. Characterization of MD9

Cell lysates were prepared using the protocol described above. About 10−40 μg of total protein was mixed with the 4× Laemmli Loading Dye (250 mM Tris, pH 6.8, 40% glycerol, 5% SDS, 0.005% bromophenol blue, 4% β-mercaptoethanol) and heated at 95−100 °C for 5 min and loaded onto a 12% Bis-Tris gel (Bio-Rad Laboratories, #4561084) and proteins were separated at 120V. Afterwards, proteins were transferred to polyvinylidene fluoride (PVDF) membranes, subsequently non-specific antibody binding was blocked with 5% nonfat dry milk (BIO-RAD, #1706404) in TBST buffer and incubated overnight at 4 °C with one of the following primary antibodies: anti-MIF (1:1000, ThermoFisher, #PA5-27343) or anti-β-actin (1:10000 Cell Signalling, #8457). The membranes were further incubated with a goat anti-rabbit HRP-conjugated secondary antibody (1:2000, DAKO, p0448) for 2 h at room temperature. Proteins were visualized with an ECL™ Prime Western Blotting System (GE Healthcare #RPN2232). The immunoblot was generated by ChemiDoc MP Imaging Systems (Bio-Rad) and analyzed by the ImageJ software. All expression levels were normalized to β-actin expression.

In comparison to the vehicle control, **MD9** reduced the MIF levels in a dose-dependent manner to provide a maximal degradation of more than 90% with a half-maximal degradation concentration (DC_{50}) of 1.5 μM. As a control, the same experiment was repeated and analyzed by a western-blot assay (Figure 3B). The potency estimated from the Western blot assay was 1 μM (Figure 3C), which is consistent with the result from the ELISA assay. The "Hook effect" was observed at 20 μM of MD9, which indicated that **MD9-**induced MIF protein level reduction depends on ternary complex formation. [35] Collectively, this demonstrate that **MD9** is an effective MIF-directed PROTAC with micromolar potency.

Action of MD9 as a PROTAC. The cellular effect of **MD9** as a PROTAC was further investigated. Firstly, the time dependence was investigated for treatment of A549 cells with 10 µM **MD9** (Figure 4A). This demonstrated that MIF degradation becomes visible after 3 hours of treatment and reached its maximum effect after 6 and 9 hours. It indicates that **MD9** induces MIF degradation in a time-dependent manner.

The involvement of both MIF and CRBN in **MD9** mediated MIF degradation was confirmed by rescue experiments with ligands that compete with MIF binding, CRBN binding or proteasomal degradation (Figure 4B). A549 cells were pretreated with MIF competitive binder **1** (100 µM) or **3** (50 µM), or CRBN competitive binder **4** (50 µM) for 2 hours, followed by co-treatment with **MD9** (10 µM) for 12 hours. For all treatments partial to complete rescue of MIF expression levels were observed, indicating that the binding of the PROTAC to both MIF and CRBN E3 ligase is required to induce protein degradation. We also used proteasome inhibitor Bortezomib[44] to block the down-stream proteasome activity (Figure 4B). With pretreatment of Bortezomib, the MIF degradation potency of **MD9** was considerably decreased, which indicates that **MD9-**induced degradation takes effect via the proteasome. Collectively, the data indicate that the action of **MD9** depends on MIF binding as well as on CRBN E3 ligase binding, which triggers proteasome-mediated degradation.

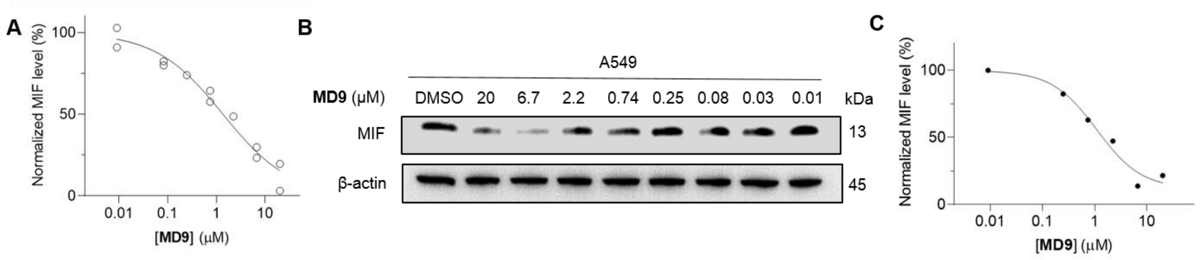

Figure S2. Treatment with **MD9** causes efficient and sustained depletion of MIF protein levels in A549 cells. (A) A549 cells were treated with the indicated concentrations of **MD9** for 12

hours. Total cell lysates were prepared and standardized for the total protein concentration. MIF protein concentration was measured by ELISA and normalized to a vehicle (DMSO)-treated control group (n=2). Nonlinear fitting of the sigmoidal curve was done by the use of GraphPad Prism with R^2 of 0.97. (B) A549 cells were treated with the indicated concentrations of **MD9** for 12 h. Immunoblot analysis on total cell lysates was standardized for the levels of β-actin as the loading control. Data are representatives of two replicates. (C) Quantification of MIF level compared with DMSO treated control in (B) using ImageJ.

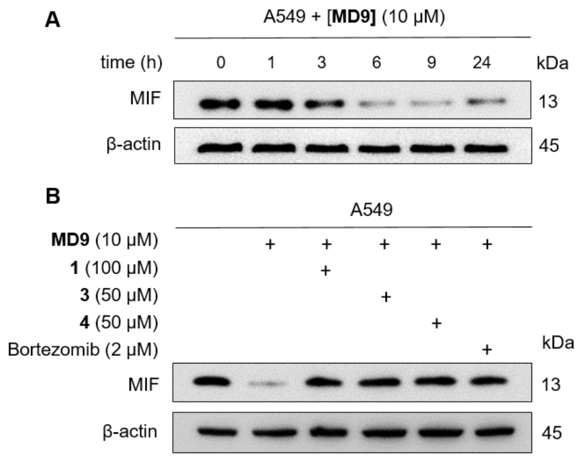

Figure S3. Time dependence and mechanism of **MD9** induced MIF degradation in A549 cells analyzed using Western blot analysis using the β-actin levels as a loading control. (A) Time-dependent MIF degradation upon treatment with 10 µM **MD9**. (B) MIF degradation after 2 hours pretreatment with MIF inhibitor **1 (ISO 1)** and **3**, E3 ligase ligand pomalidomide **4**, proteasome inhibitor Bortezomib or vehicle (DMSO) followed by treatment with 10 µM **MD9** or vehicle (DMSO) for 12 h. Data are representatives of two replicates.

S5. Subcellular colocalization study

To visualize the intracellular location of **MD13** and MIF protein, immunofluorescence staining was applied. A549 cells were seeded on glass coverslips in a 6-well plate at a density of 1x10^5 cells per well. After overnight incubation, cells were treated with 1 µM **MD13** for 1 hour to study the internalization of **MD13** or 12 hours to study the degradation of MIF protein. The cells with 1-hour treatment were fixed with methanol before coverslips were mounted onto slides with anti-fading mounting medium with DAPI stain (Invitrogen, Waltham, MA. USA). To visualize MIF protein, cells after 12-hour treatment were fixed with methanol and then unspecific binding was blocked with blocking buffer (1% BSA, 22.52 mg/mL glycine in 0.1%PBST). Cells were incubated with a MIF antibody (1:500, ThermoFisher, #PA5-27343) for 1 hour at room temperature. Subsequently, cells were treated with Alexa Fluor Plus 488 conjugated goat anti-rabbit secondary antibody (1:1000, #A32732, Invitrogen, Waltham, MA. USA). Next, coverslips were mounted onto slides with anti-fading mounting medium with DAPI stain (Invitrogen, Waltham, MA. USA). The pictures were acquired using a Leica SP8 confocal laser scanning microscope and analyzed by ImageJ.

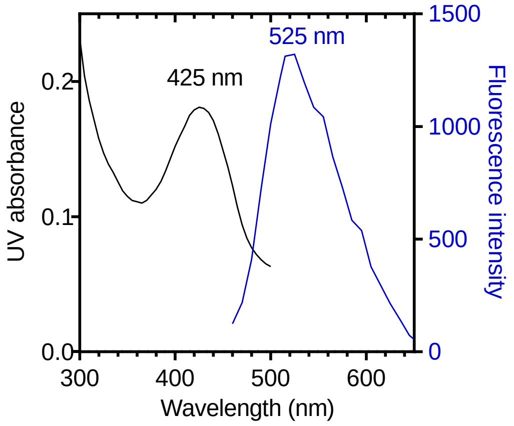

Figure S4. UV absorbance and fluorescence emission spectra of compound **MD13**. UV absorbance spectrum was measure at 20 µM in PBS (pH7.4). Fluorescence emission was measured at 1 µM in PBS (pH7.4) with excitation wavelength of 425 nm.

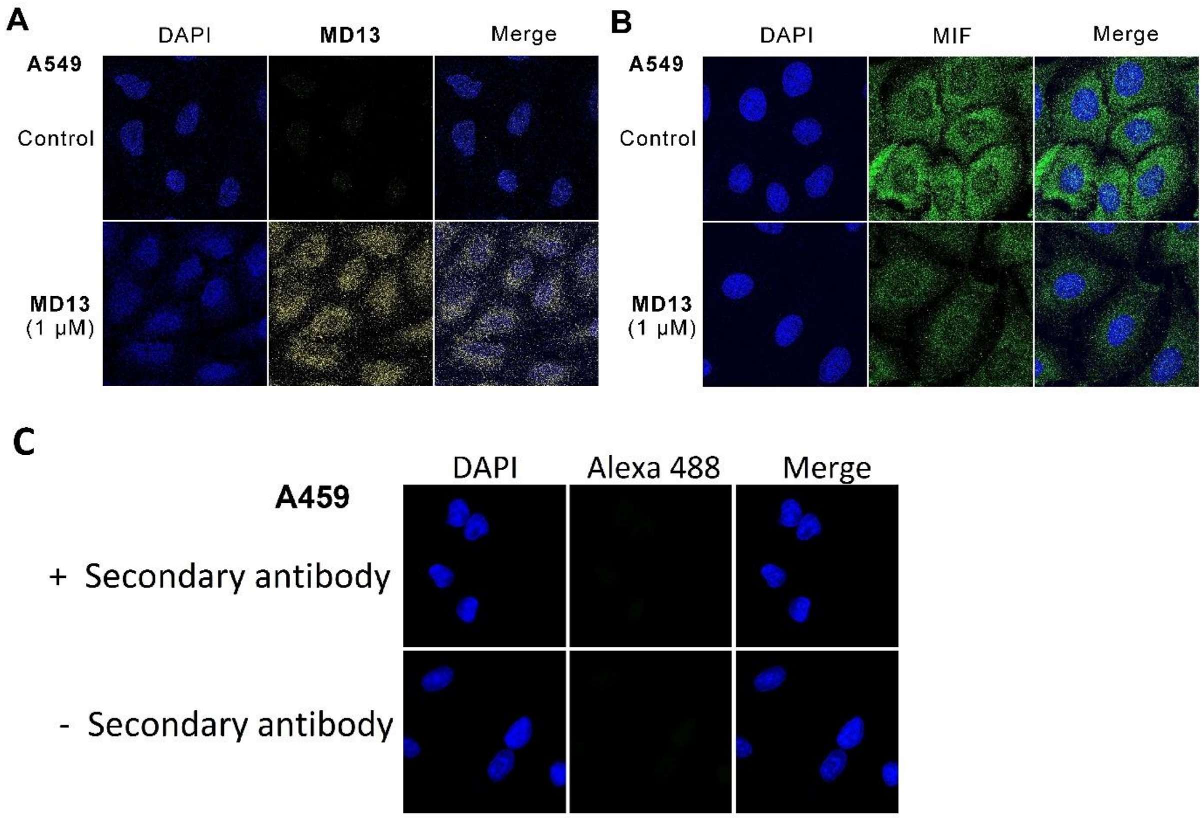

Figure S5. Visualization of the subcellular localization of MIF and **MD13** indicates that **MD13** can enter cells and can induce MIF degradation. (A) A549 cells were seeded onto glass slides and treated with **MD13** (1 µM) or vehicle for 1 hour, followed by fixing and counterstaining with DAPI. **MD13** was visualized through the Leica/Alexa 430 channel with an excitation wavelength of 405 nm. (B) A549 cells were seeded onto glass slides and treated with **MD13** (1 µM) or vehicle for 12 hours. Cells were fixed and immunostained for MIF levels using a MIF antibody and subsequently a secondary goat anti-rabbit antibody conjugated with Alexa Fluor Plus 488. Cells were counterstained with DAPI and pictures were acquired using a Leica SP8 confocal laser scanning microscope and analyzed by ImageJ. Original magnification is ×400. (C) Control staining without primary antibody for (B).

S6. Western-blot uncropped images

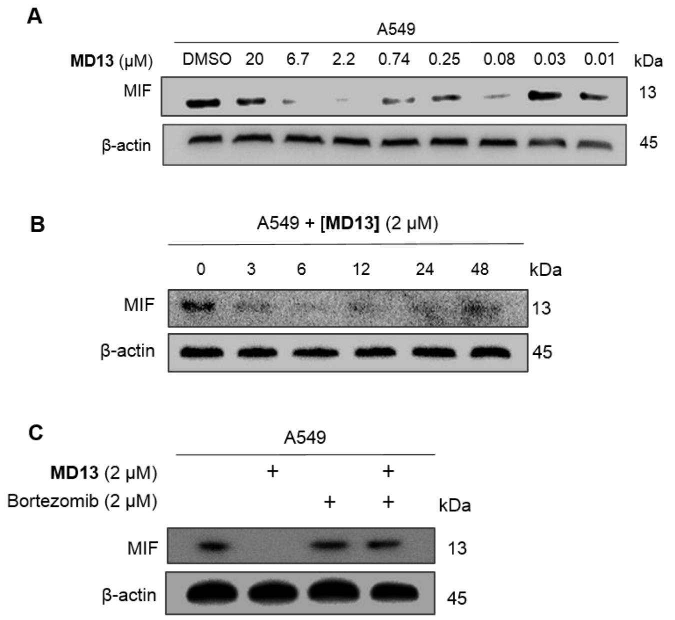

Figure S6. Western-blot replicates of **MD13**. (A) Dose-dependent assay of **MD13** on MIF level; (B) Time-dependent assay of **MD13** on MIF level; (C) Rescue assay by proteasome inhibitor. Experimental details are depicted in the main part of this article.

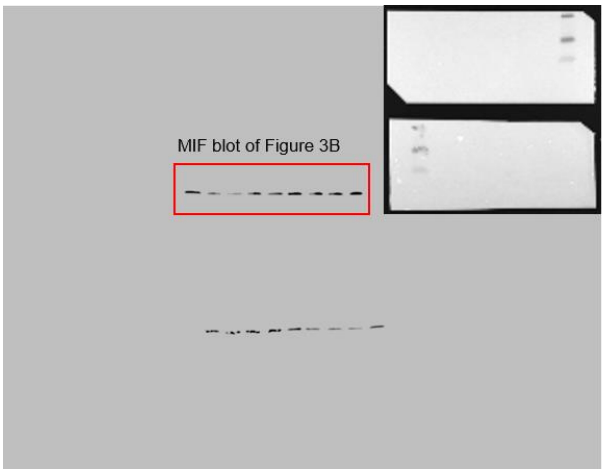

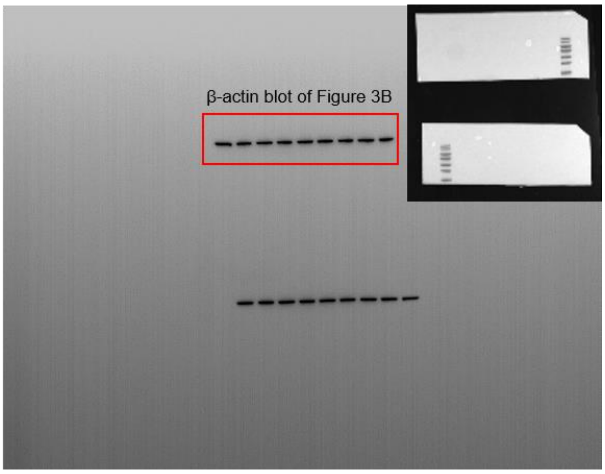

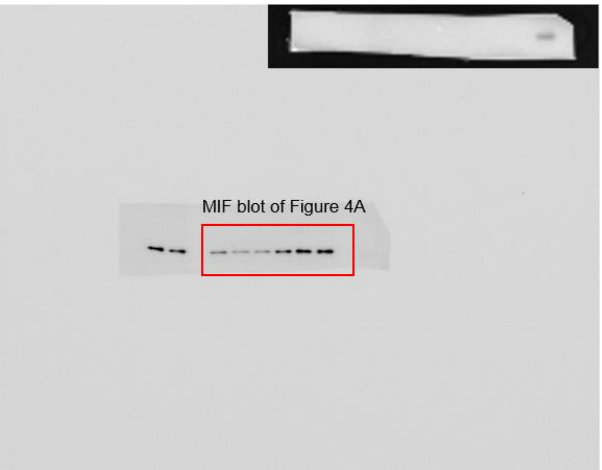
MIF blot of Figure 4A
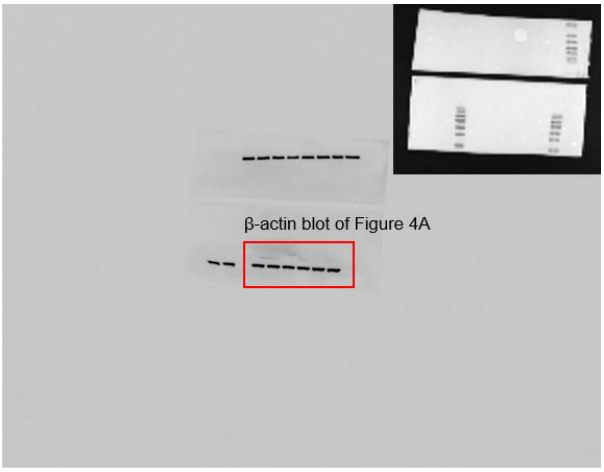
β-actin blot of Figure 4A
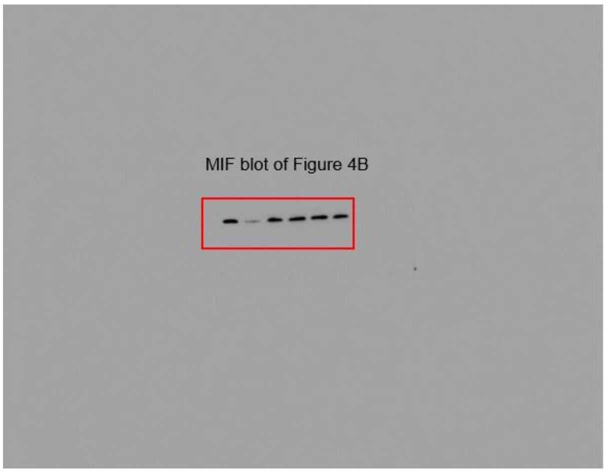
MIF blot of Figure 4B
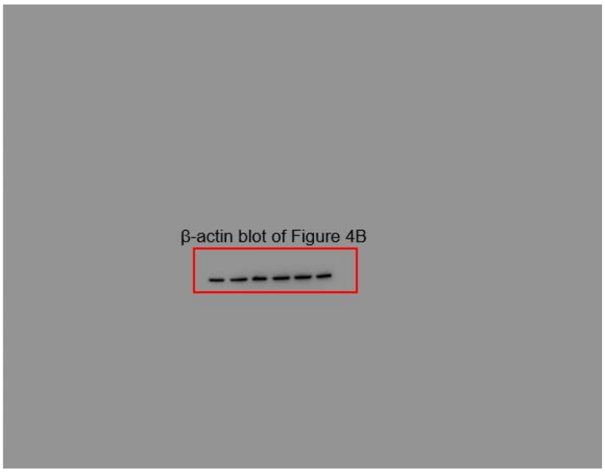
β-actin blot of Figure 4B
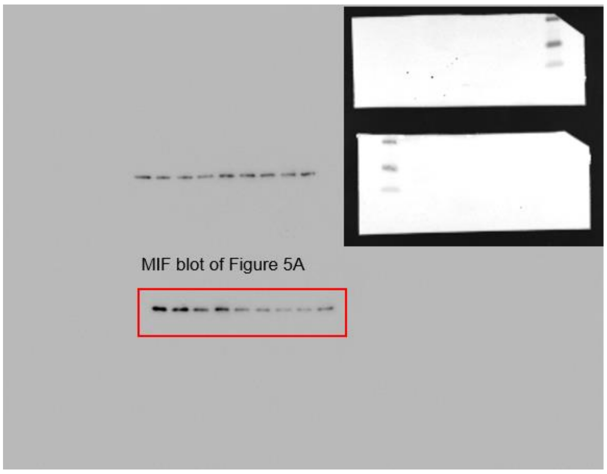
MIF blot of Figure 5A
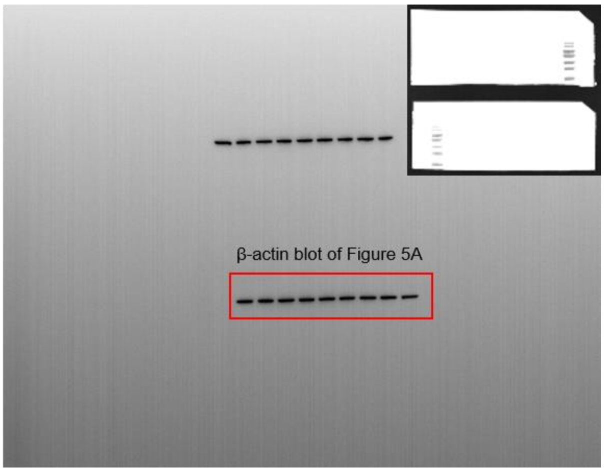
β-actin blot of Figure 5A

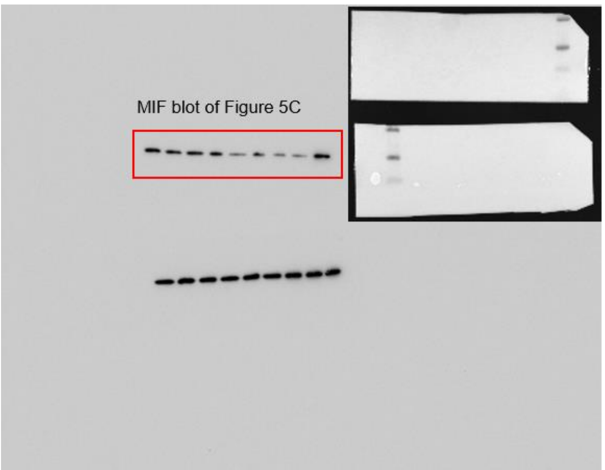
MIF blot of Figure 5C

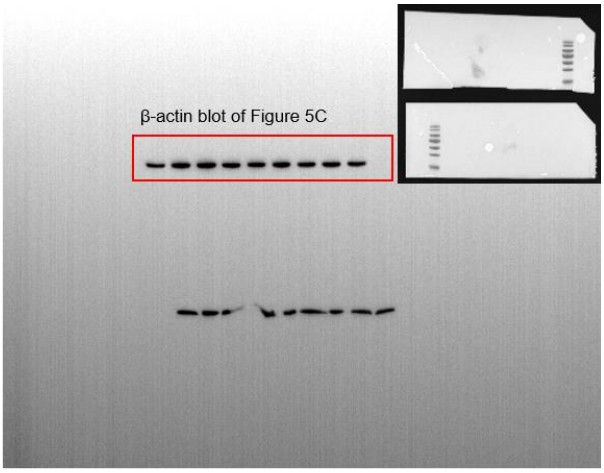
β-actin blot of Figure 5C

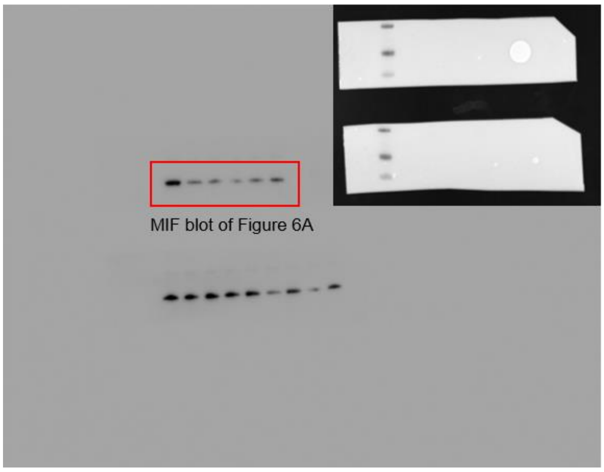
MIF blot of Figure 6A

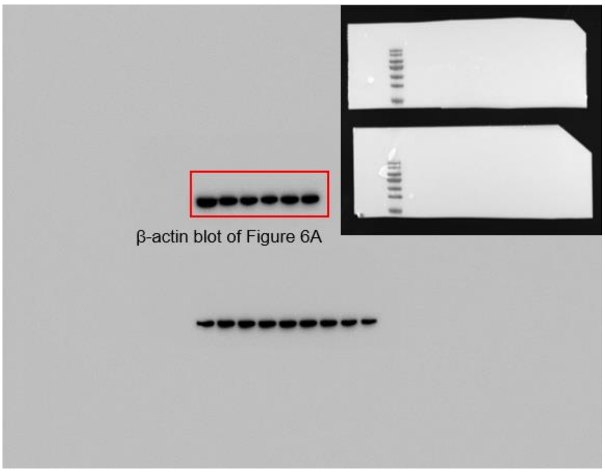
β-actin blot of Figure 6A

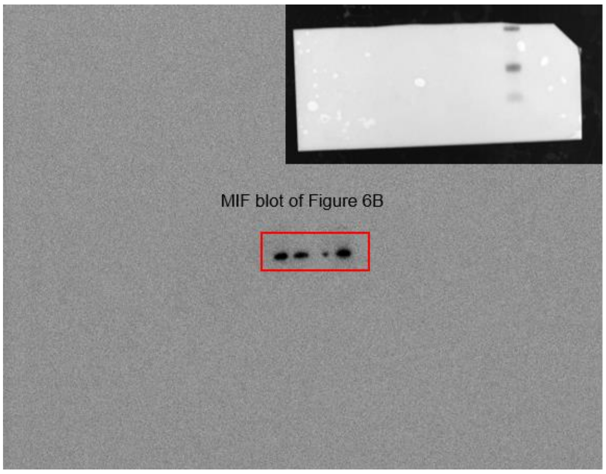
MIF blot of Figure 6B

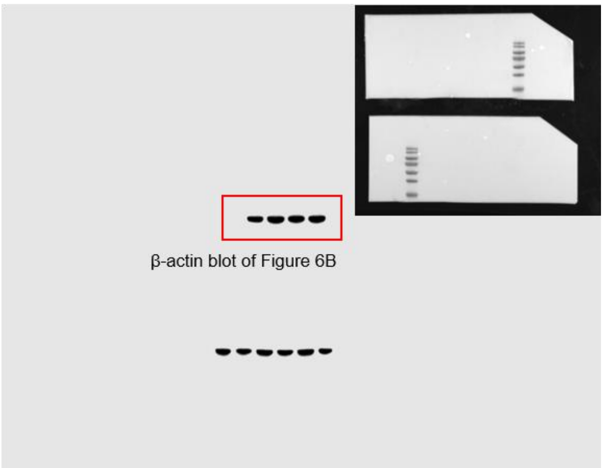
β-actin blot of Figure 6B

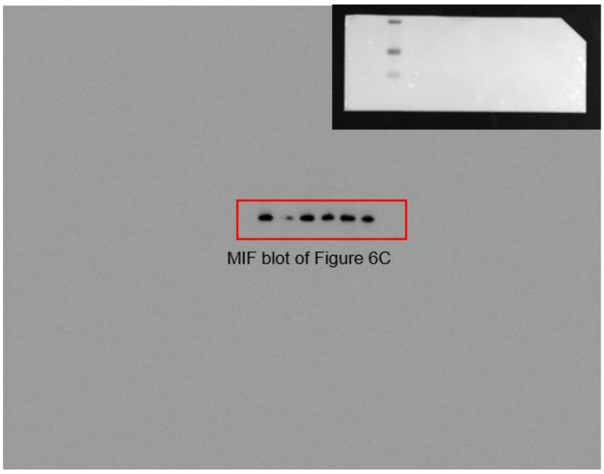
MIF blot of Figure 6C

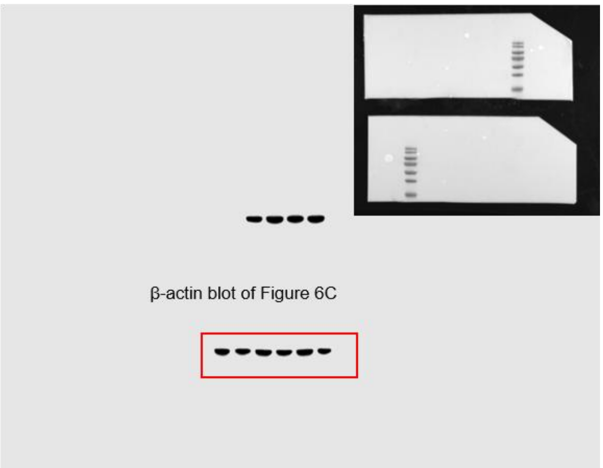
β-actin blot of Figure 6C

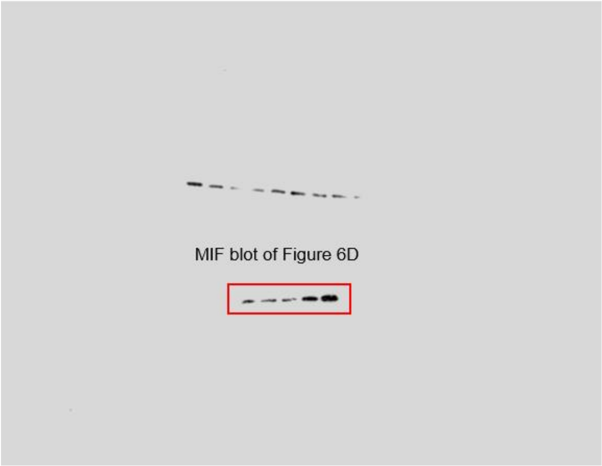
MIF blot of Figure 6D

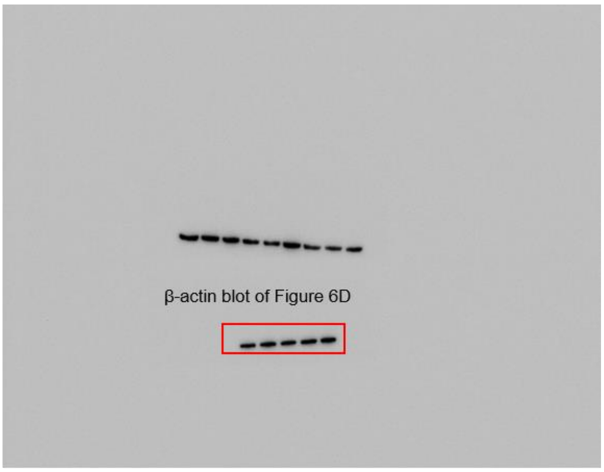
β-actin blot of Figure 6D

S7. Cell viability assay of MD13

A total of 1500 cells were seeded per well in 96-well plates and cultured for 24 h. Cells were then treated with the respective drugs or vehicle for 72 h. Next, cells were incubated at 37 ˚C = with a medium containing MTS for 90 min following the protocol of CellTiter 96 AQueous One Solution Reagent (Promega, Madison, WI, USA). The absorbance was determined at a wavelength of 490 nm using a Synergy H1 plate reader (BioTek, Winooski, VT, USA). Experiments were performed in triplicate and repeated at least three times.

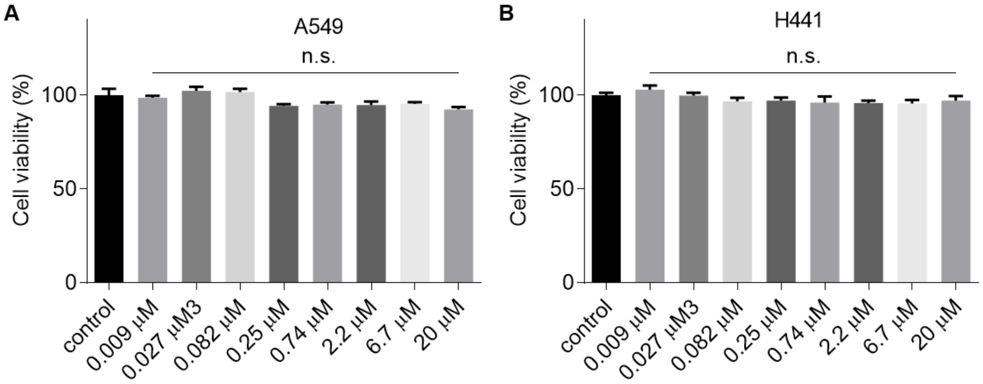

Figure S7. Cell viability assay of **MD13**. The compound **MD13** was incubated with (A) A549 cells or (B) H441 cells (3000 per well) at concentrations from 0.009 to 20 µM for 24 hour. Cell viability was measure by using MTS assay and normalized by set the vehicle (DMSO) group as 100% (n=3, n.s., p>0.05, *p<0.05 *vs* control group.)

S8. Cell growth, cell cycle and pERK signaling study

Cell proliferation was measured with the CyQUANT® Direct Cell Proliferation Assay Kit (Thermo Fisher, #C35011). A549 cells were cultured in 96-well plates at a density of 1,000 cells/well and treated with different concentrations of MIF PROTAC (0.02-20 μM) for 72 h. Cells were incubated with detection reagent (100 μL) for 60 min at 37 ˚C with 5% CO_2. The fluorescence of each well was read at 485/535nm by plate reader (BioTek).

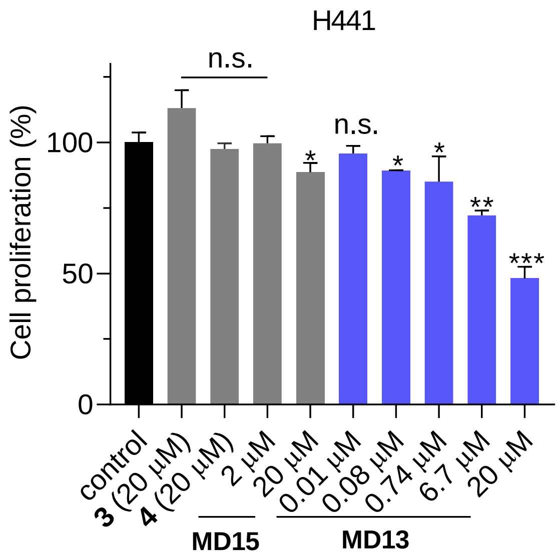

Figure S8. Cells were seeded with a density of 1000 cells per well in a 96-well plate. After overnight incubation, the cells were treated with various concentration of **MD13** for 72 hours. The resulting cells were quantified by CyQUANT® cell proliferation assays and compared with the vehicle (DMSO) control (n=3).

Tumor spheroid assay. A549 cells (1000 cells/well) were seeded onto a 96-well round-bottomed ultra low attachment plate (Corning). After 2 days of incubation without disturbance, the spheroid was treated with indicated compound every 3 days. Images were captured and the diameter of each tumor spheroid were measured on the indicated days post-treatment using an

inverted microscope (Nikon Eclipse Ti) connected with a NIS-elements software. The data were analyzed and plotted with Graphpad Prism8.

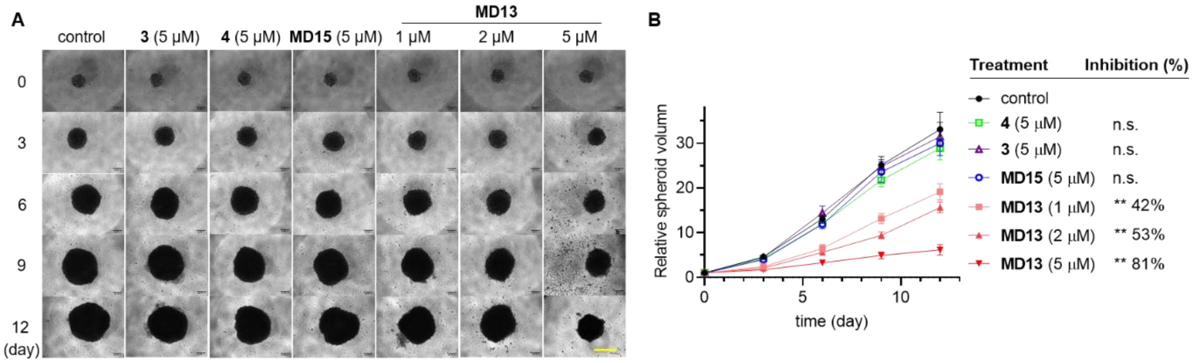

Figure S9. MD13 treatment inhibits growth of A549 cancer cells in a spheroid model. A549 cells were seeded in an ultralow attachment 96-well round-bottomed plate (1000 cells/well) to generate tumor spheroids (a single spheroid per well). After initiation, the spheroids were treated with different compounds at the indicated concentrations every three days. DMSO was used as vehicle control. The day of the first treatment was indicated as day 0. (A) Representative images were obtained at the indicated intervals using an inverted microscope. Scale bar: 500 μm. (B) Analysis was carried out using NIS-Elements AR 3.1 software, and growth curves were obtained relative to the untreated spheroids (day 0) and plotted with GraphPad Prism 8. Values are shown as means ± SD (n = 3 spheroids/time point, $^{**}p < 0.01$ *vs* control).

Flow cytometry. For cell cycle analysis, A549 cells were seeded in 6-well plates at a density of $1x10^5$ per well. The next day, the cells were treated with the appropriate compound or vehicle for 48h. Subsequently, the cells were washed with PBS (3×) and then harvested after trypinzation. After centrifugation at 300g, the cells were incubated with a solution containing 20 μg/ml propidium iodide (PI) (Sigma, P4864) and 0.1% (v/v) Triton-X100 (Sigma, T8787) for 15 min at room temperature. Fluorescence was detected by a Cytoflex flow cytometer

(Beckman Coulter, Woerden, the Netherlands) immediately. 30,000 cells were collected for each sample. Data were analyzed using FlowJo software (Tree start, Ashland, USA).

ERK signaling pathway study. A549 cells (2x10^5 cells per well) were seeded into each well of a 6-well plate with 2 mL RPMI-1640 medium containing 10% FBS (Costar Europe, Badhoevedorp, The Netherlands), and 1% penicillin/streptomycin solution (Corning). After overnight culturing, the cells were treated with **MD13**, **MD15**, or DMSO for the time indicated. After that, cells were lysed by RIPA buffer containing 1× PhosSTOP and protease inhibitor (PI) cocktail (Roche, Mannheim, Germany). The BCA Protein Assay Kit (Pierce, Rockford IL, USA) was used to determine the protein concentration. 30 µg protein was separated by a pre-cast 10% NuPAGE Bis-Tris gel (Invitrogen, USA) and then transferred to a polyvinylidene difluoride (PVDF) membrane. After blocking with 5% of skimmed milk for 1 hour at room temperature and incubation with the appropriate primary antibody (pERK, #9101, Cell Signaling, 1:1000; GAPDH, #97166, Cell Signaling, 1:10000) overnight at 4 °C, the membrane was treated with an HRP-conjugated secondary goat anti-rabbit antibody (#P0448, Dako, 1:2000) or rabbit anti-mouse antibody (#P0260, Dako, 1:2000) at room temperature for 1 hour. The protein bands were visualized with enhanced chemiluminescence (ECL) solution (GE Healthcare). The figures were quantified with imageJ software based on grayscale.

Reference

[1] M. Daśko, M. Przybyłowska, J. Rachon, M. Masłyk, K. Kubiński, M. Misiak, A. Składanowski, S. Demkowicz, *Eur. J. Med. Chem.* **2017**, *128*, 79–87.

[2] Z. Xiao, D. Chen, S. Song, R. van der Vlag, P. E. van der Wouden, R. van Merkerk, R. H. Cool, A. K. H. Hirsch, B. N. Melgert, W. J. Quax, G. J. Poelarends, F. J. Dekker, *J. Med. Chem.* **2020**, *63*, 11920–11933.

[3] X. Zhang, D. Thummuri, X. Liu, W. Hu, P. Zhang, S. Khan, Y. Yuan, D. Zhou, G.

Zheng, *Eur. J. Med. Chem.* **2020**, *192*, 112186.

[4] C. Qin, Y. Hu, B. Zhou, E. Fernandez-Salas, C. Y. Yang, L. Liu, D. McEachern, S. Przybranowski, M. Wang, J. Stuckey, J. Meagher, L. Bai, Z. Chen, M. Lin, J. Yang, D. N. Ziazadeh, F. Xu, J. Hu, W. Xiang, L. Huang, S. Li, B. Wen, D. Sun, S. Wang, *J. Med. Chem.* **2018**, *61*, 6685–6704.

[5] W. Li, C. Gao, L. Zhao, Z. Yuan, Y. Chen, Y. Jiang, *Eur. J. Med. Chem.* **2018**, *151*, 237–247.

[6] J. A. Cisneros, M. J. Robertson, M. Valhondo, W. L. Jorgensen, *J. Am. Chem. Soc.* **2016**, *138*, 8630–8638.

[7] S. Chamni, Q. L. He, Y. Dang, S. Bhat, J. O. Liu, D. Romo, *ACS Chem. Biol.* **2011**, *6*, 1175–1181.

[8] Z. Xiao, M. Fokkens, D. Chen, T. Kok, G. Proietti, R. van Merkerk, G. J. Poelarends, F. J. Dekker, *Eur. J. Med. Chem.* **2020**, *186*, 111849–111862.

S9. HRMS and purity.

Table S2. Mass spectra and HPLC of key compounds.

Control (DMSO)

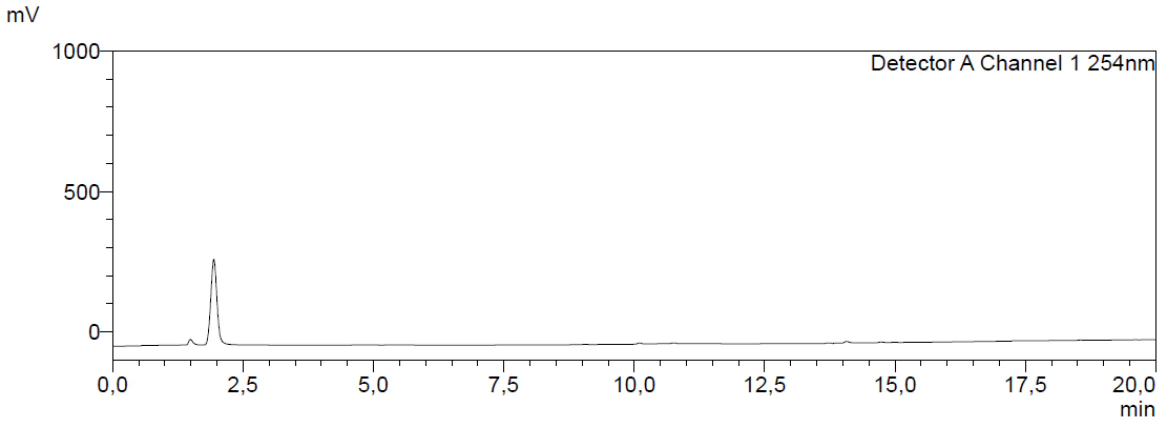

Compound **3**, retention yime 10.2 min, purity 99.6%.

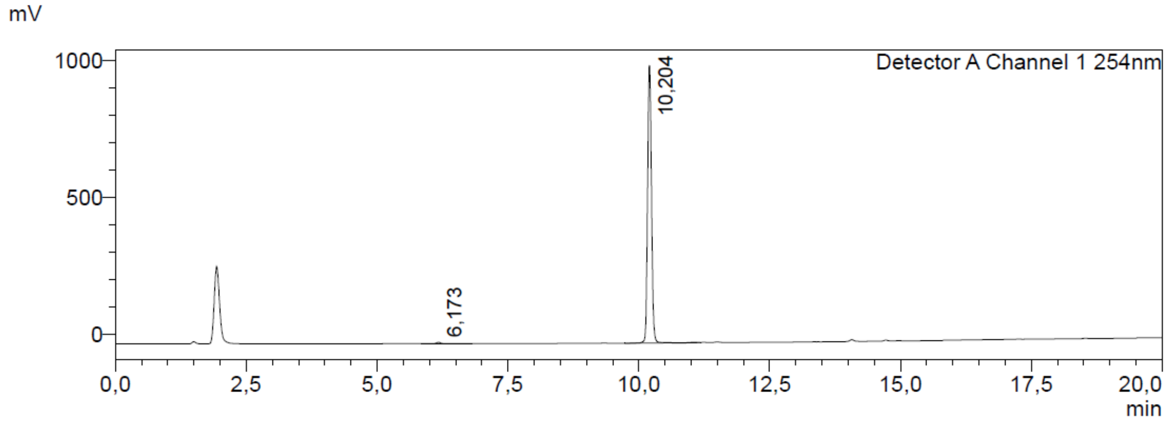

Compound **4**, retention yime 6.5 min, purity 98.9%.

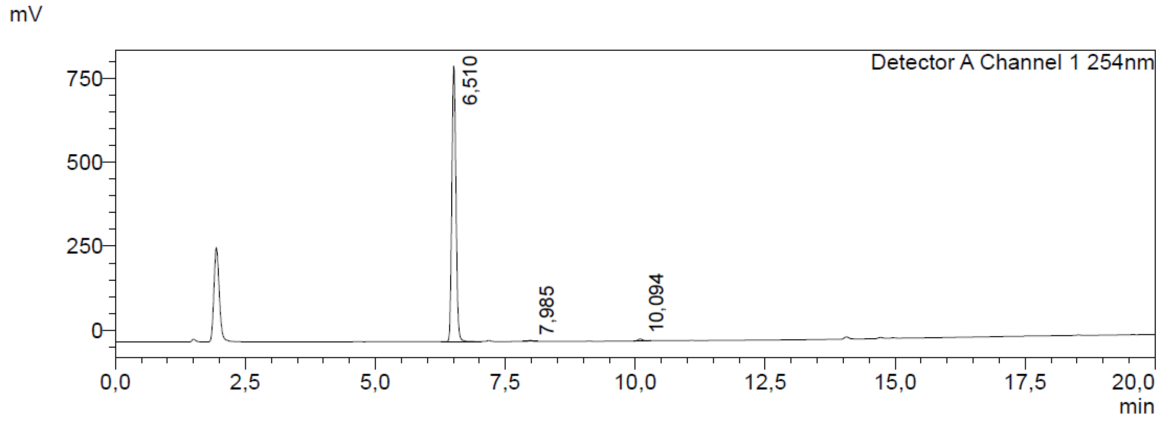

MD1, $C_{33}H_{26}N_7O_8$, calculated mass 648.1798, found 648.1848. Retention time 9.3 min. Purity 98.8%.

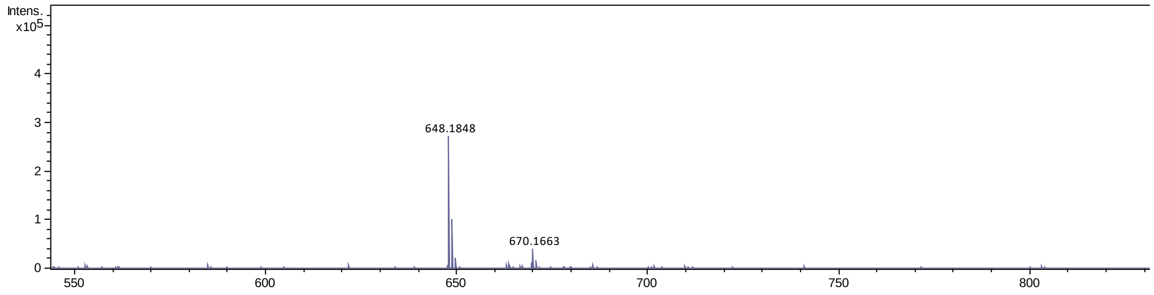

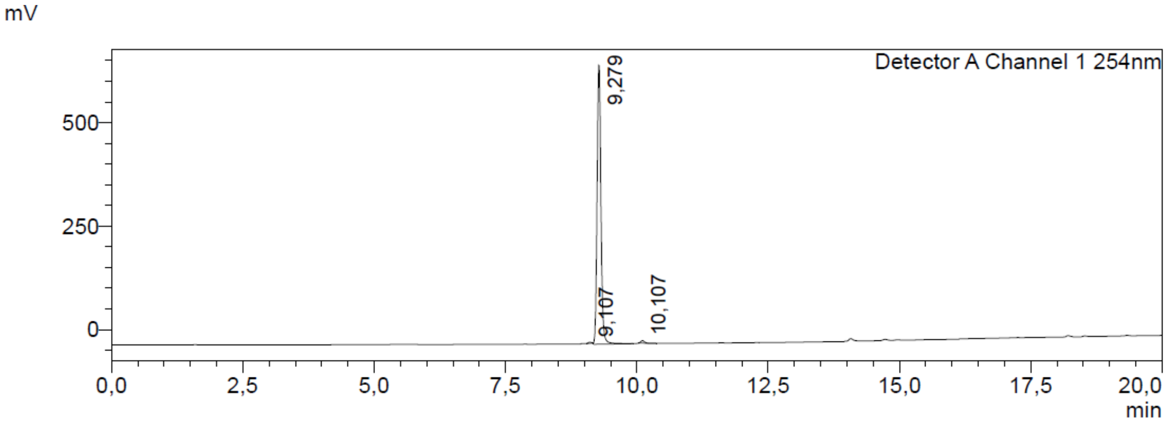

MD2, $C_{35}H_{28}O_9N_7$, calculated mass 690.1904, found 690.1951. Retention time 9.0 min. Purity 96.5%.

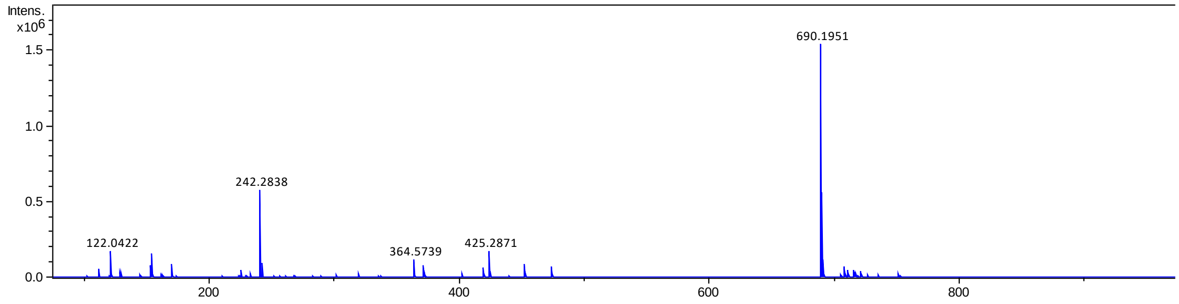

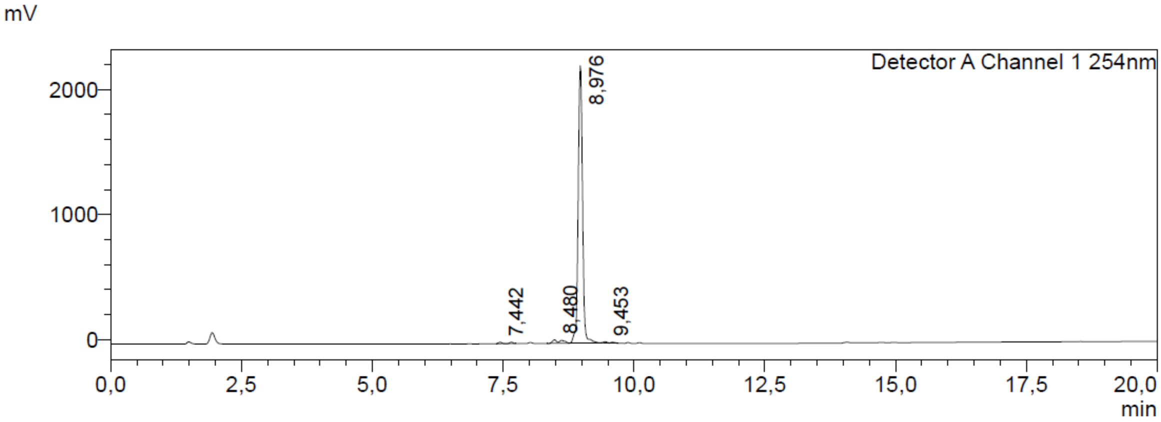

MD3, $C_{36}H_{30}O_9N_7$, calculated mass 704.2060, found 704.2098. Retention time 9.3 min. Purity 97%.

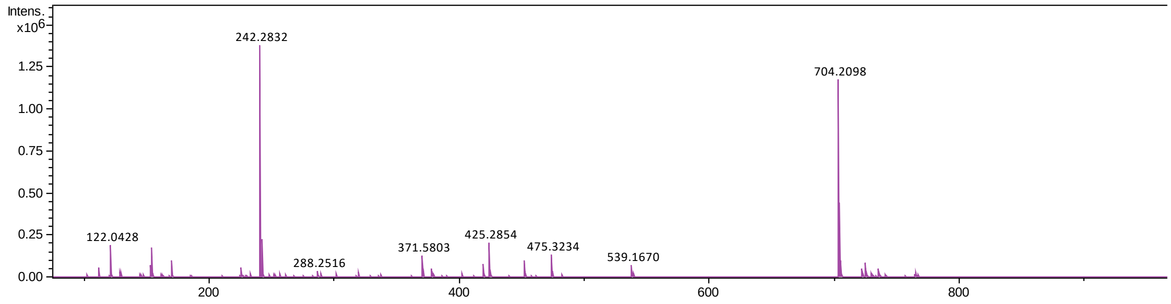

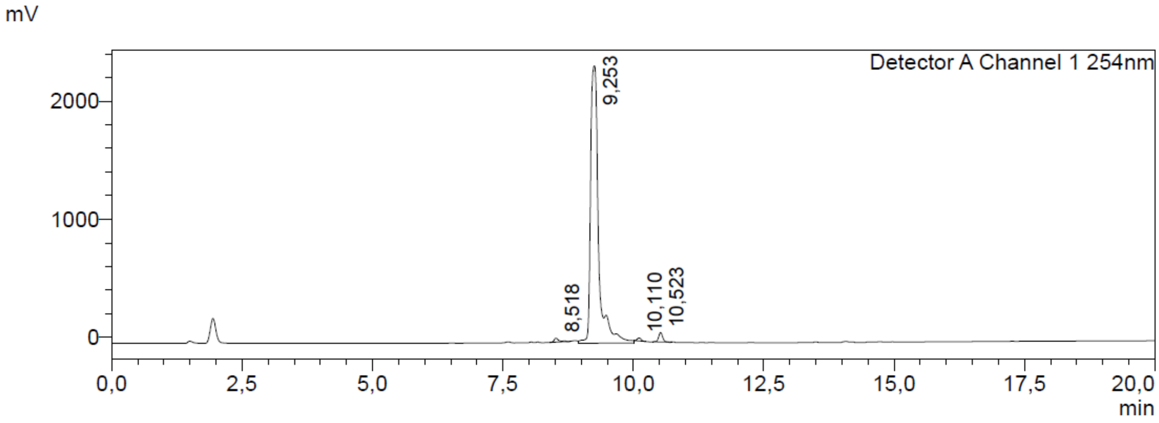

MD4, $C_{37}H_{32}O_9N_7$, calculated mass 718.2217, found 718.2257. Retention time 9.5 min. Purity 95.2%.

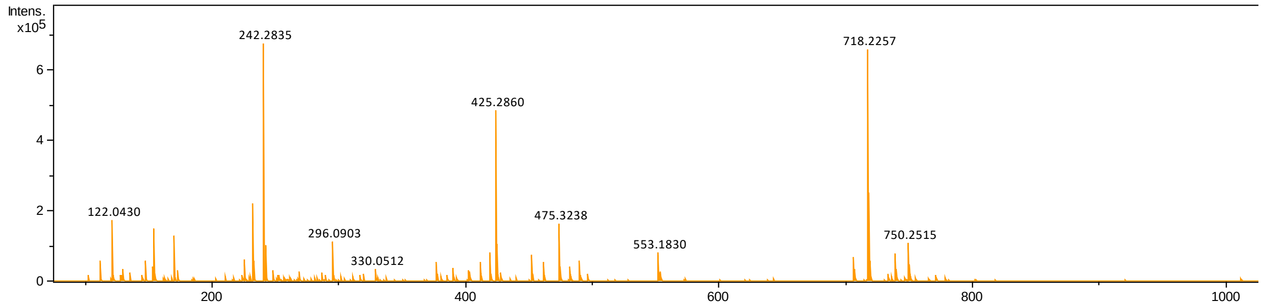

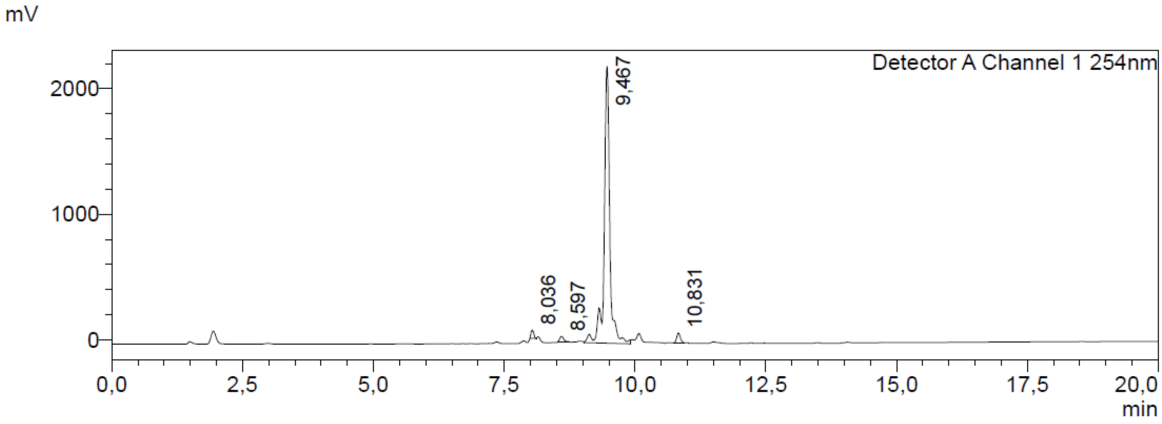

MD5, $C_{38}H_{34}N_7O_9$, calculated mass 732.2373, found 732.2432. Retention time 9.0 min. Purity 95.3%.

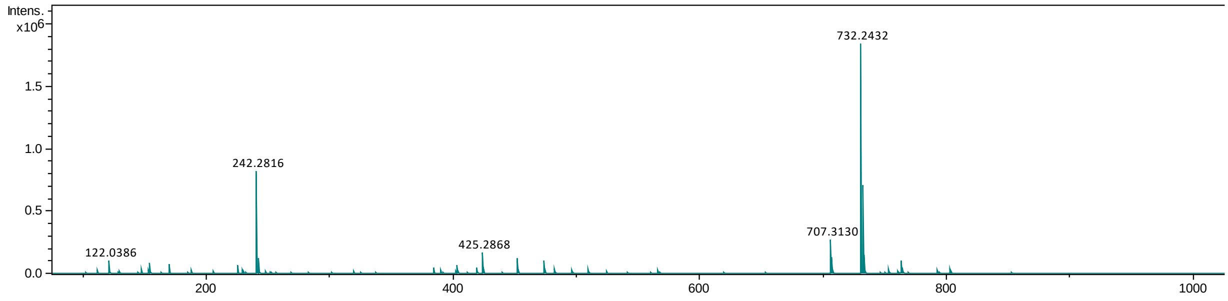

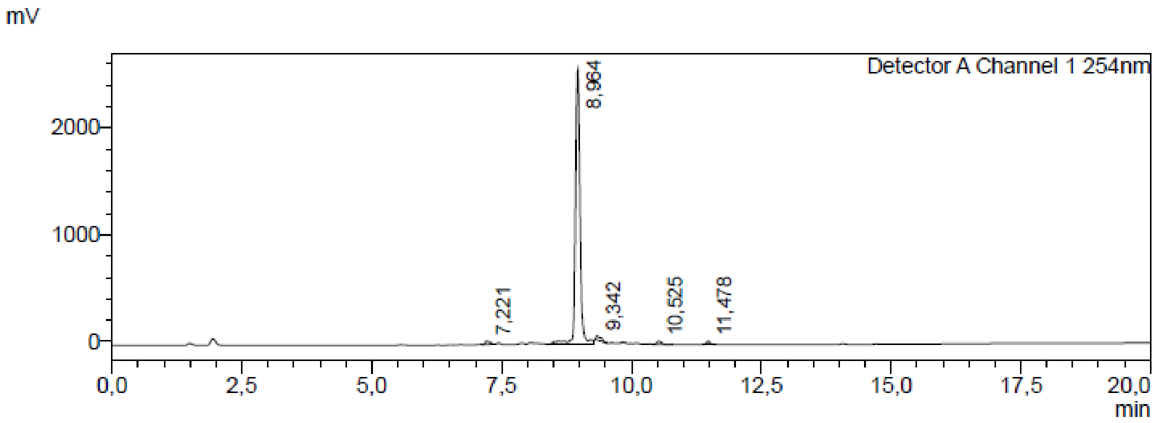

MD6, $C_{42}H_{34}O_9N_7$, calculated mass 780.2373, found 780.2423. Retention time 10 min. Purity 96.3%.

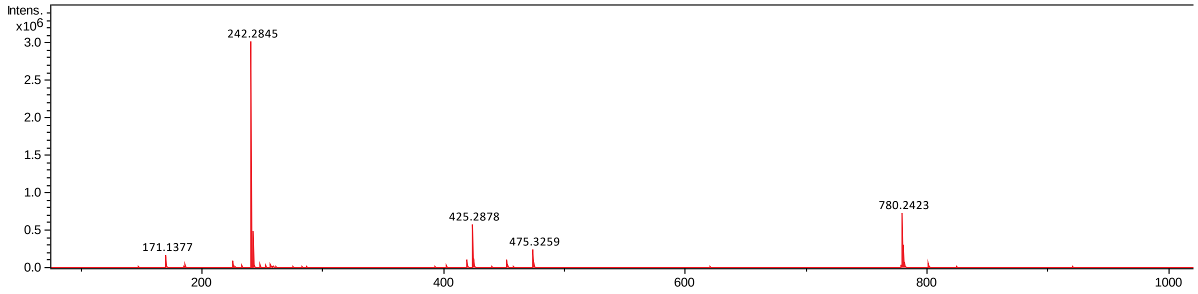

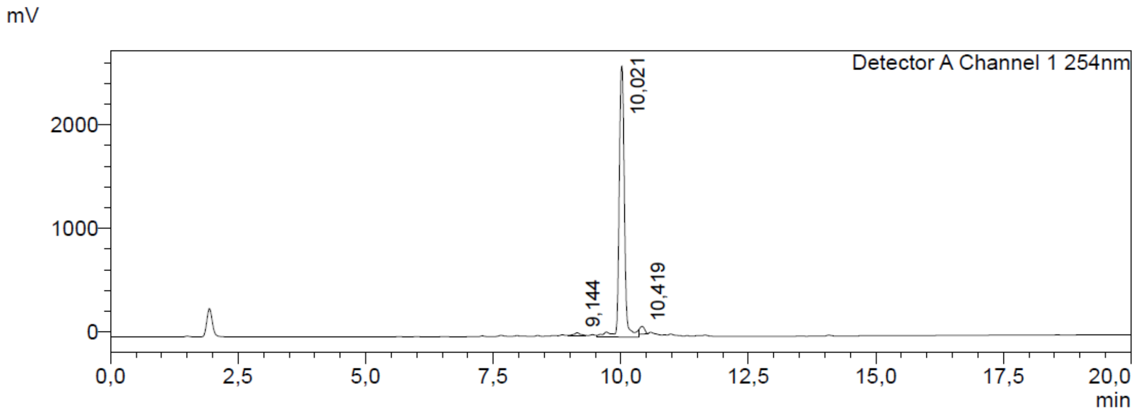

MD7, $C_{32}H_{30}O_8N_5$, calculated mass 612.2050, found 612.2060. Retention time 9.5 min. Purity 95.3%.

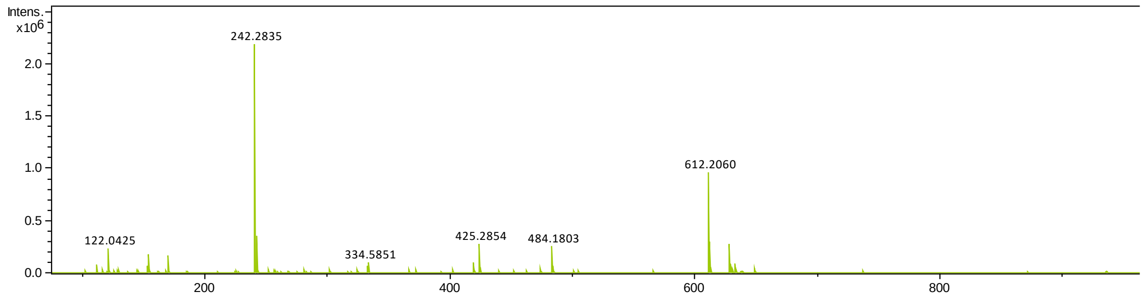

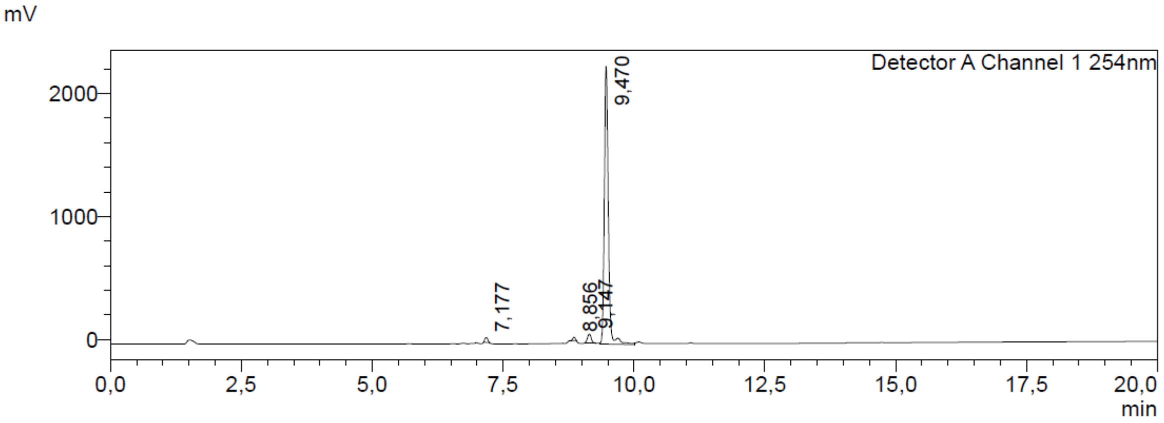

MD8, $C_{33}H_{32}O_8N_5$, calculated mass 626.2206, found 626.2221. Retention time 9.1 min. Purity 98%.

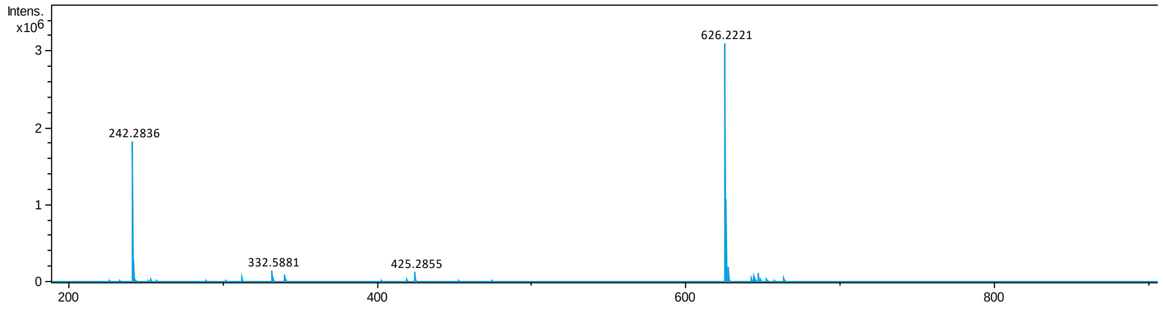

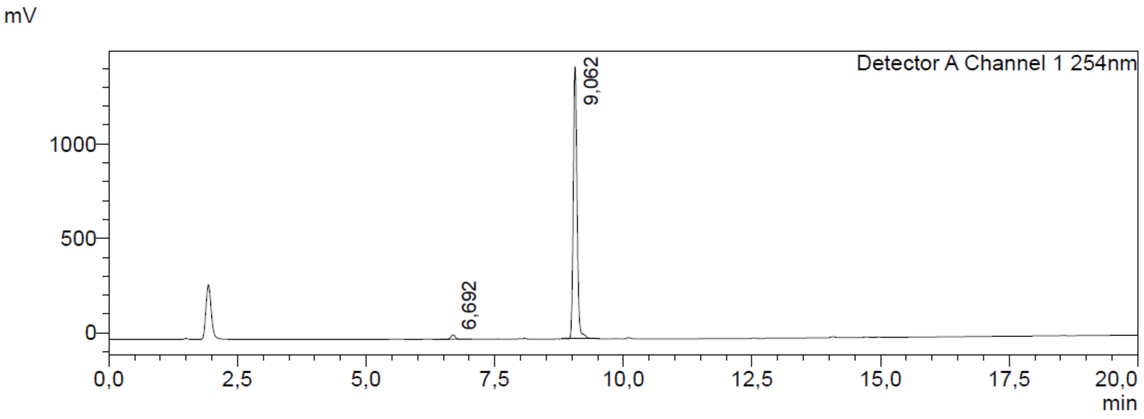

MD9, $C_{34}H_{34}O_8N_5$, calculated mass 640.2363, found 640.2373. Retention time 10.3 min. Purity 98%.

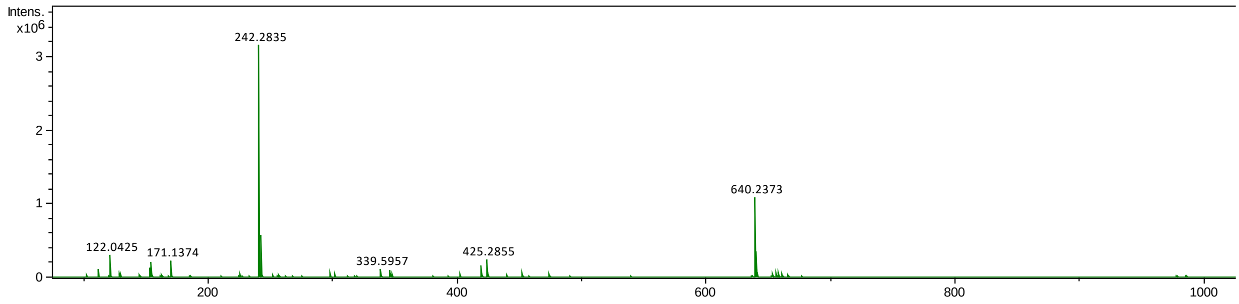

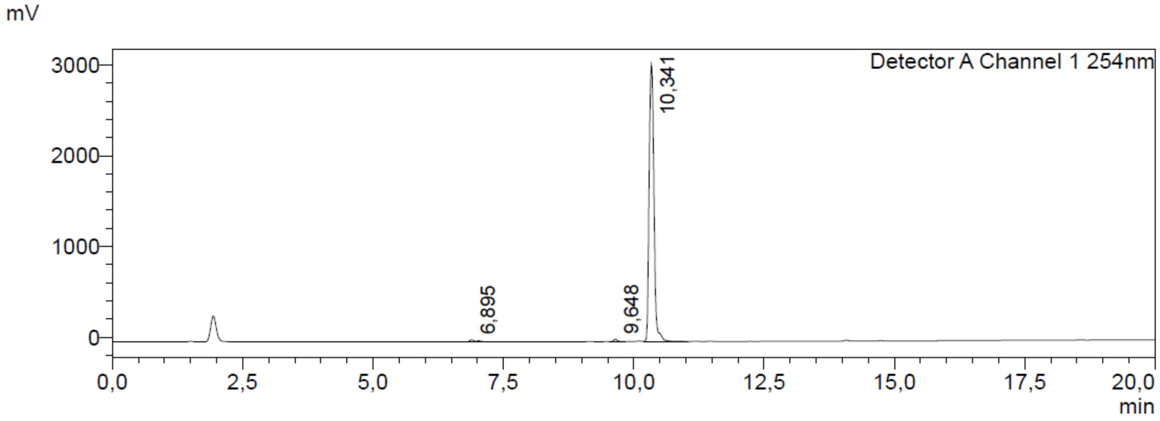

MD10, $C_{33}H_{29}O_8N_8$, calculated mass 665.2064, found 665.2064. Retention time 8.3 min. Purity 98.5%.

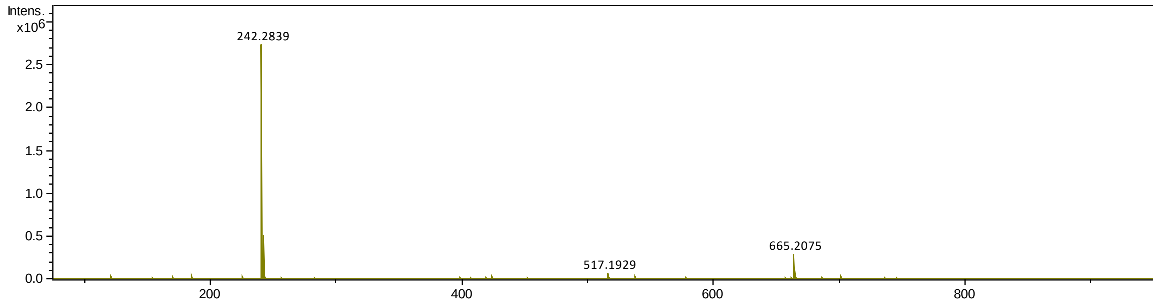

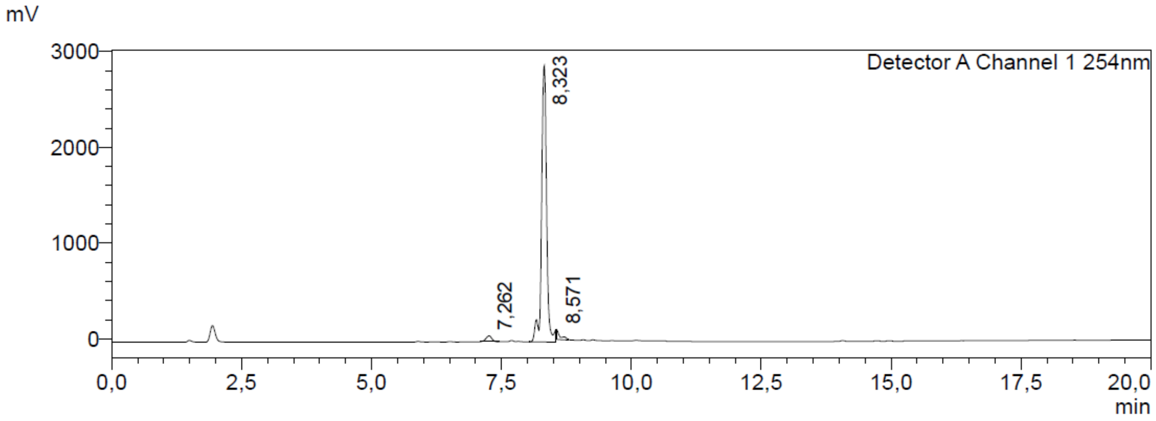

MD11, $C_{36}H_{33}N_8O_9$, calculated mass 721.2326, found 721.2385. Retention time 8.3 min. Purity 97.3%.

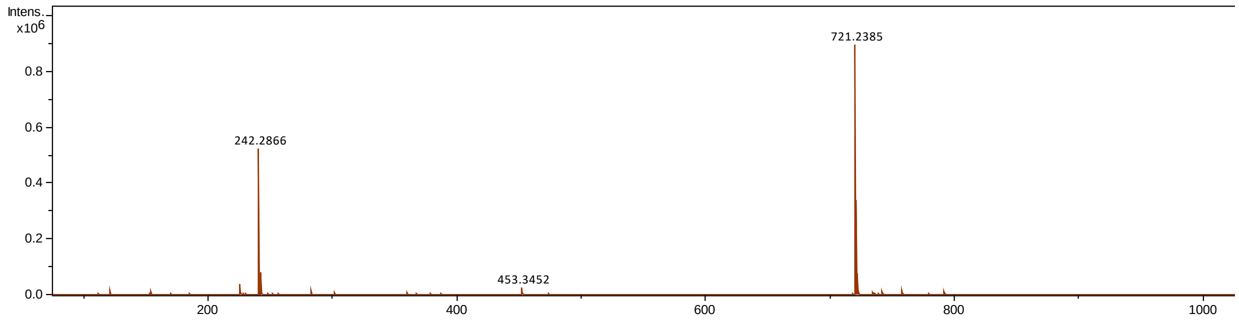

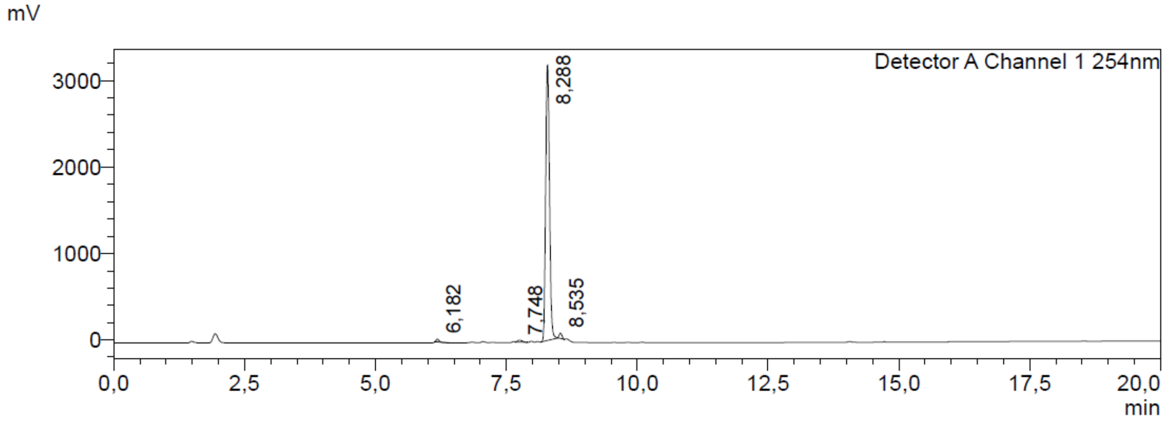

MD12, $C_{40}H_{33}O_9N_8$, calculated mass 769.2326, found 769.2378. Retention time 9.4 min. Purity 95.1%.

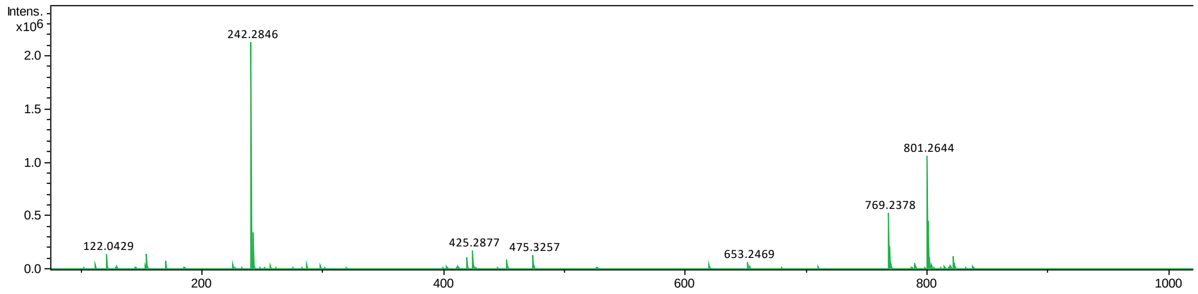

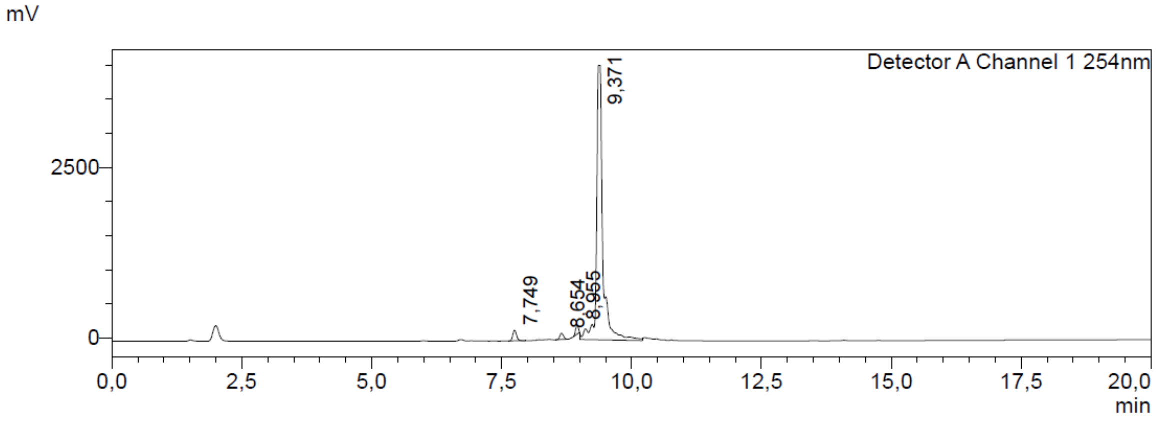

MD13, $C_{35}H_{36}O_8N_5$, calculated mass 654,2558, found 654,2557. Retention time 12 min. Purity 98.9%.

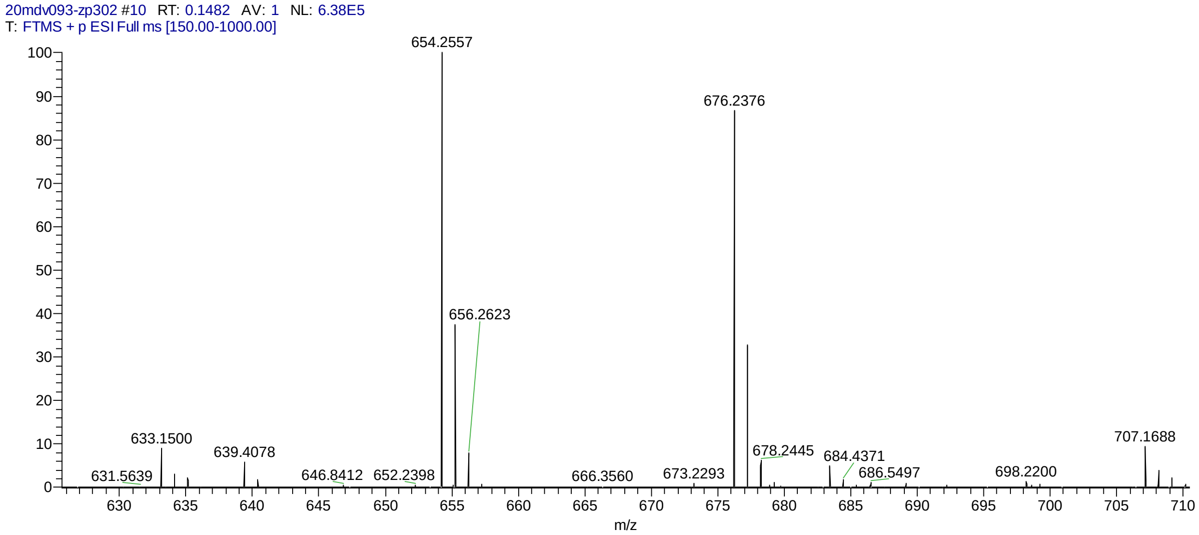

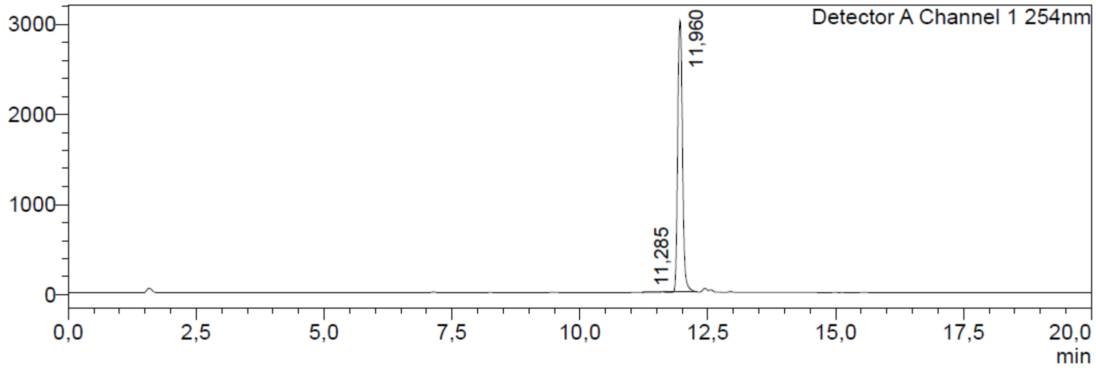

MD14, $C_{37}H_{40}O_8N_5$, calculated mass 682,2871, found 682,2870. Retention time 8.3 min. Purity 93.5%.

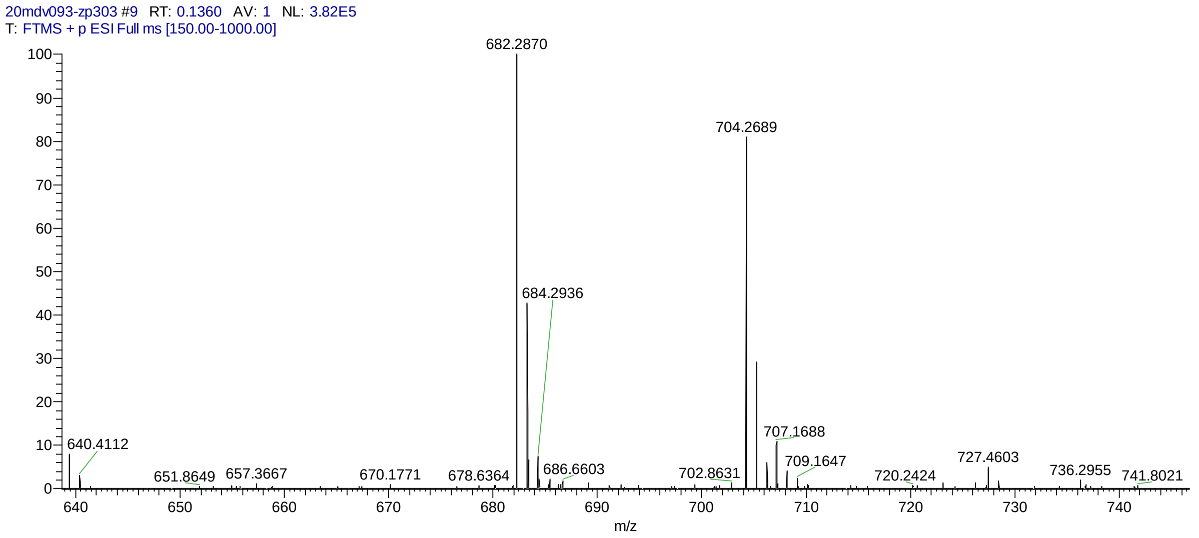

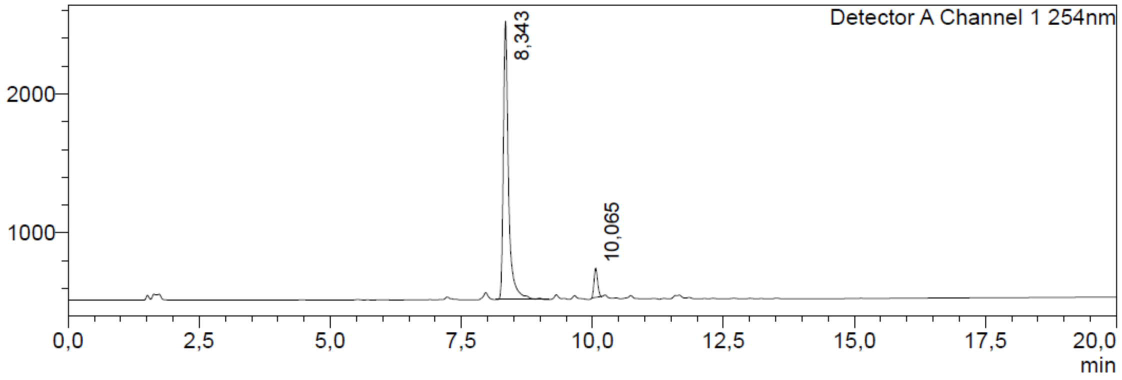

MD15, $C_{36}H_{38}O_8N_5$, calculated mass 668,2715, found 668,2715. Retention time 10.1 min. Purity 99%.

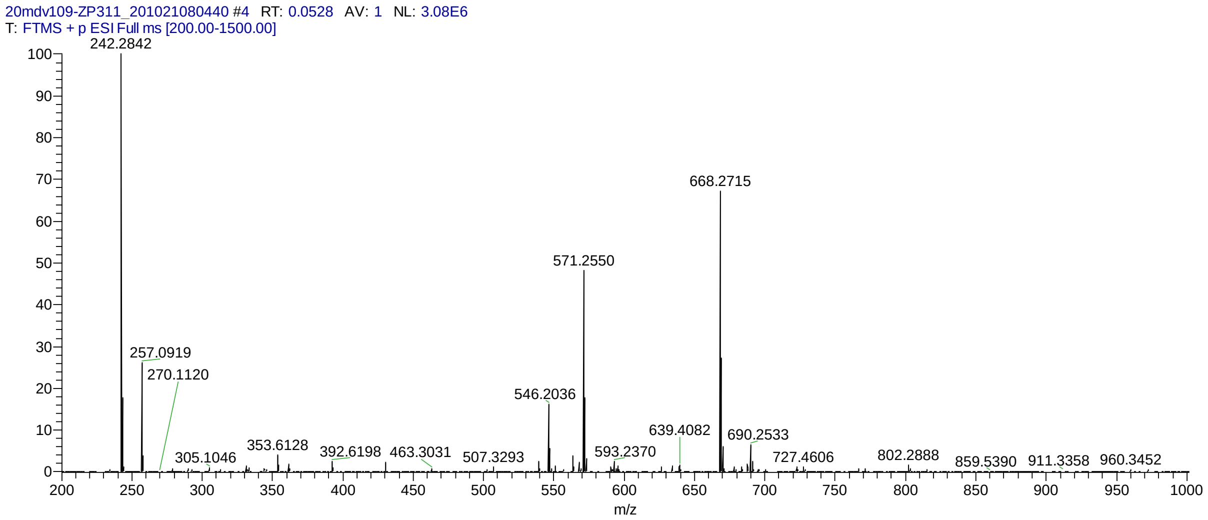

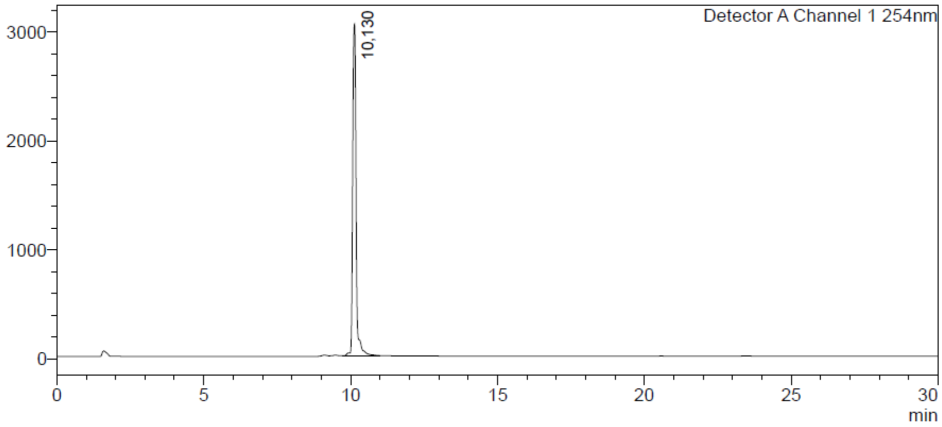

S10. NMR spectra

5

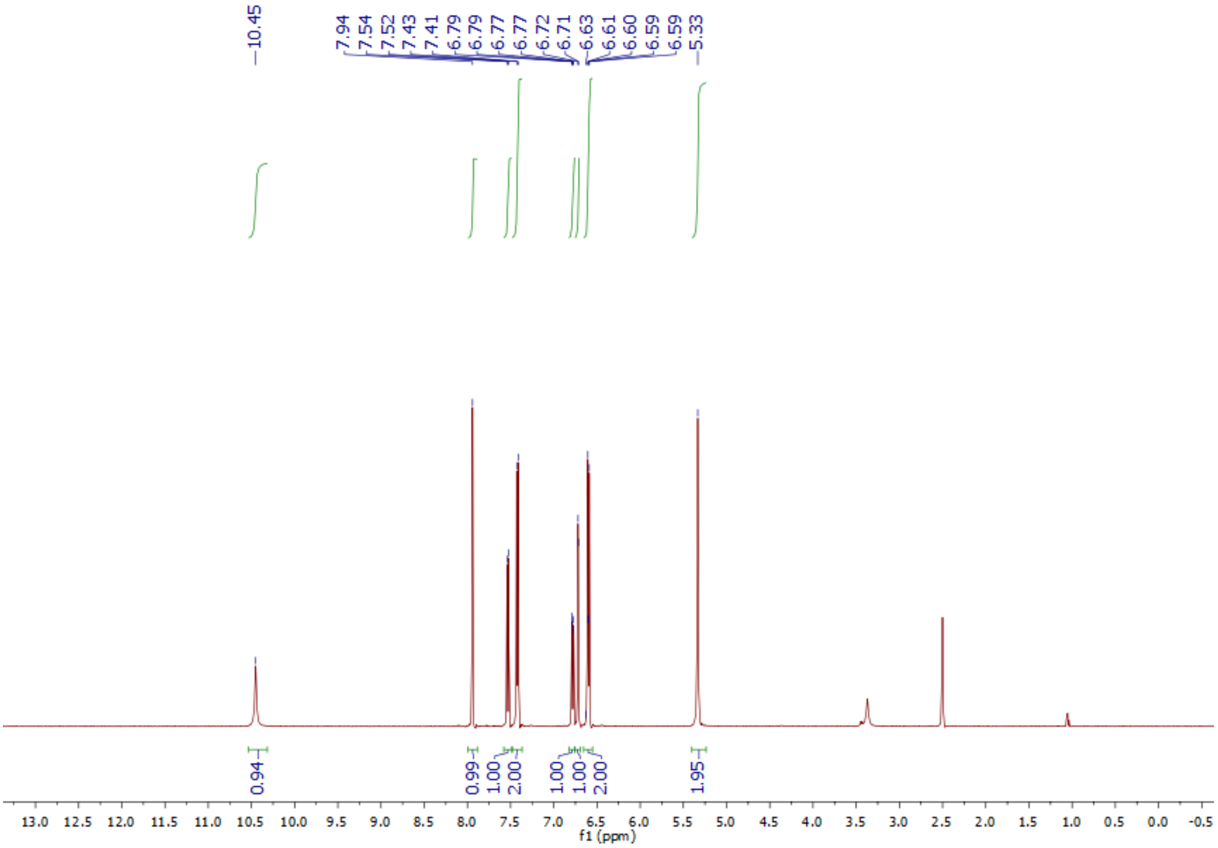

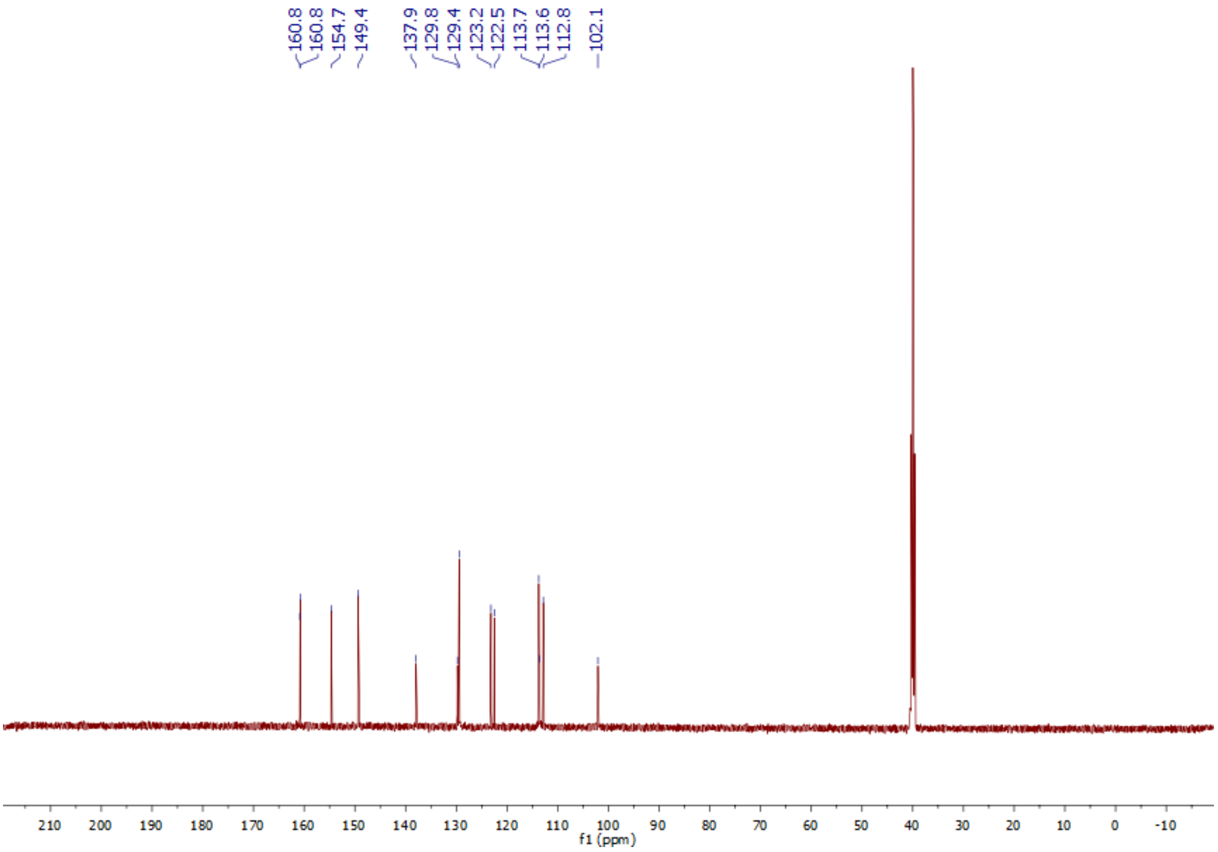

6a

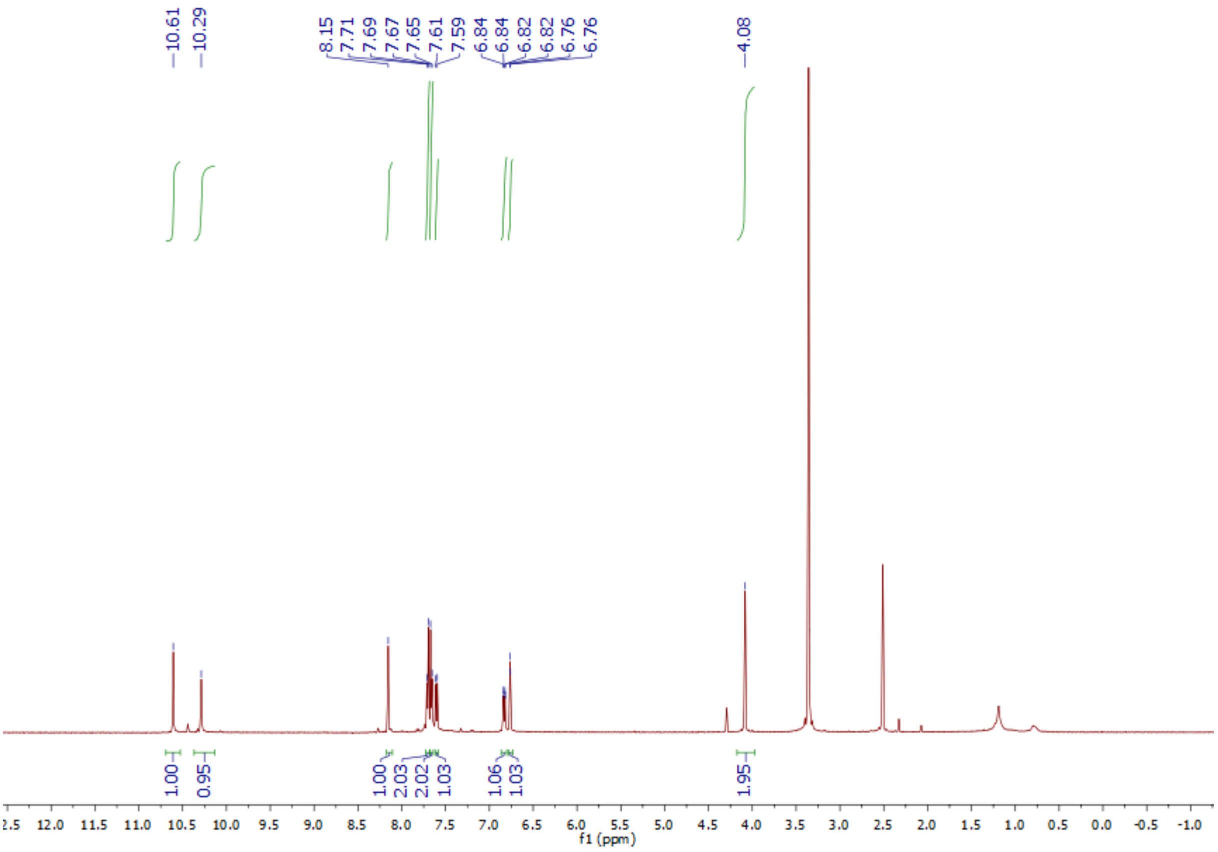
10.61
10.29
8.15
7.71
7.69
7.67
7.65
7.61
7.59
6.84
6.84
6.82
6.82
6.76
6.76
4.08
1.00
0.95
1.00
2.03
2.02
1.03
1.06
1.03
1.95
f1 (ppm)

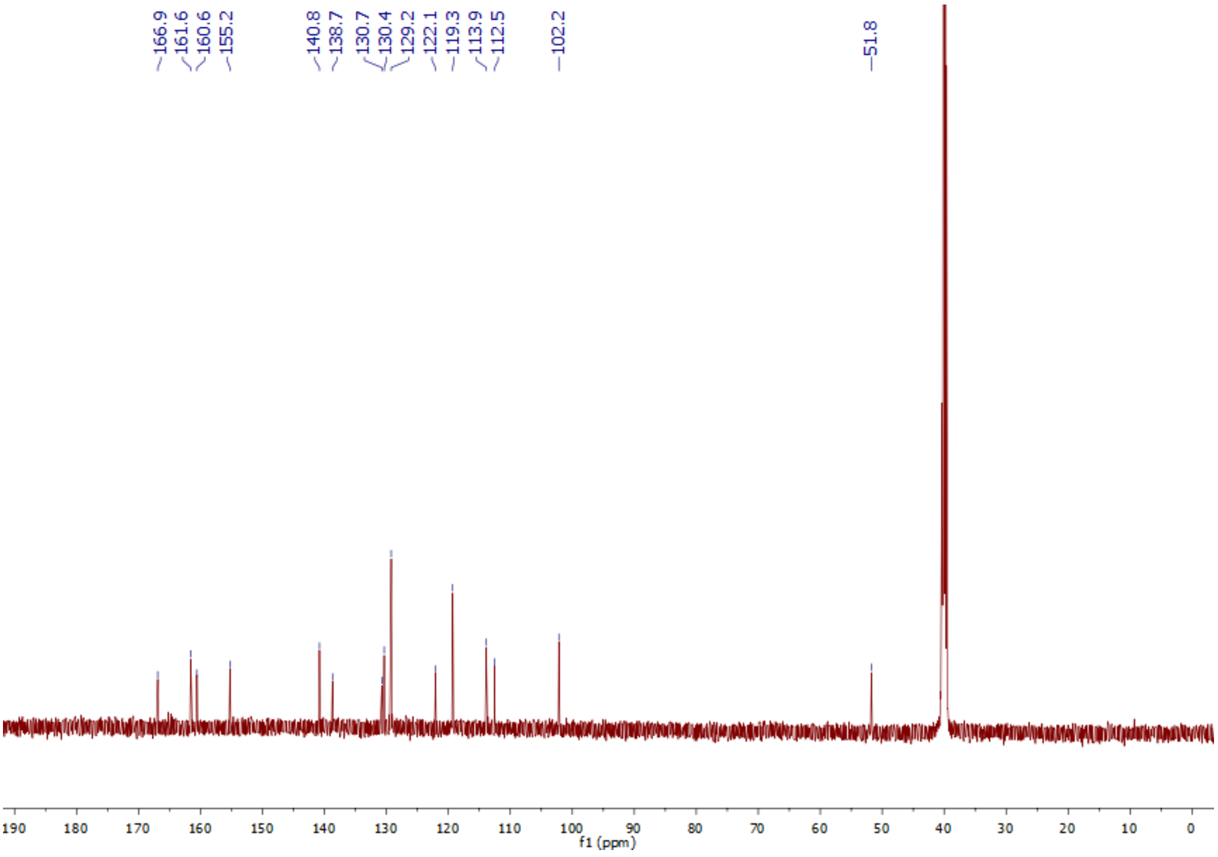
166.9
161.6
160.6
155.2
140.8
138.7
130.7
130.4
129.2
122.1
119.3
113.9
112.5
102.2
51.8
f1 (ppm)

6b

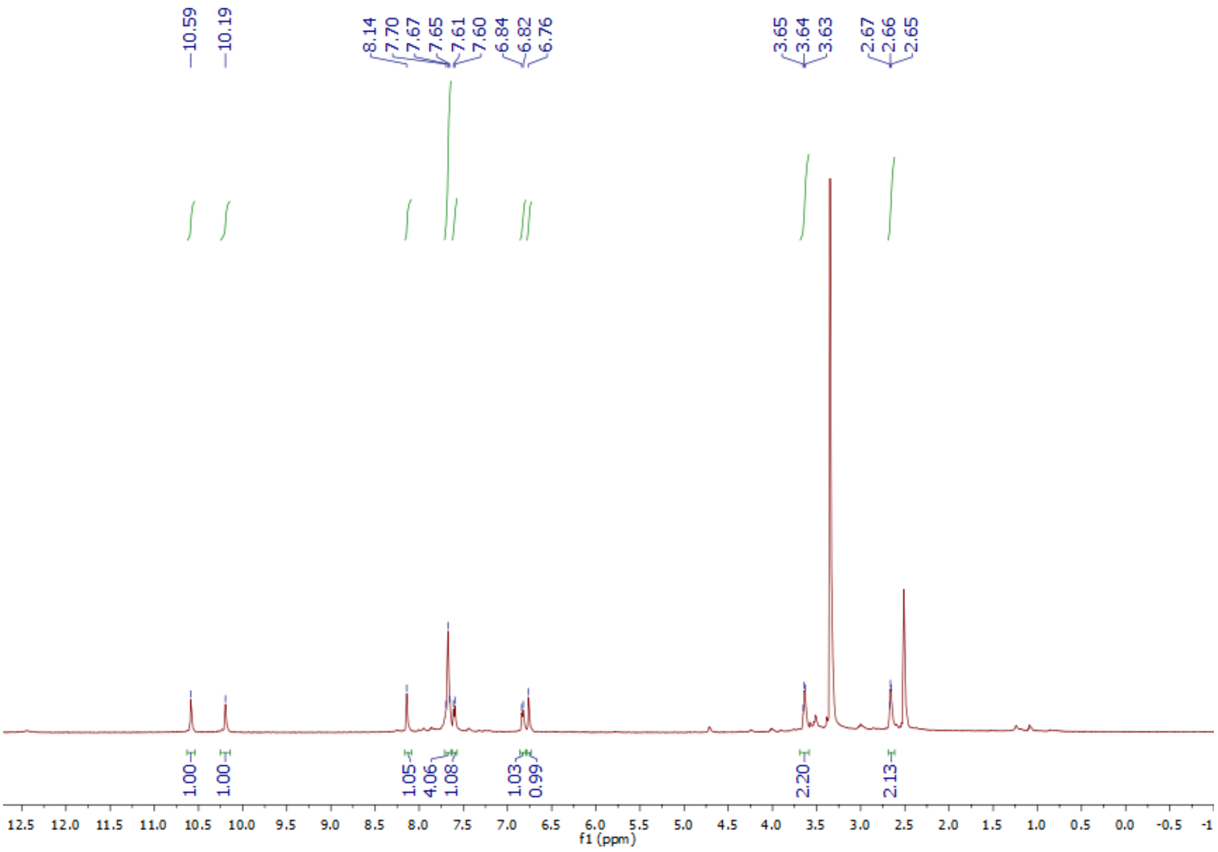
10.59
10.19
8.14
7.70
7.67
7.65
7.61
7.60
6.84
6.82
6.76
3.65
3.64
3.63
2.67
2.66
2.65
1.00
1.00
1.05
4.06
1.08
1.03
0.99
2.20
2.13
f1 (ppm)

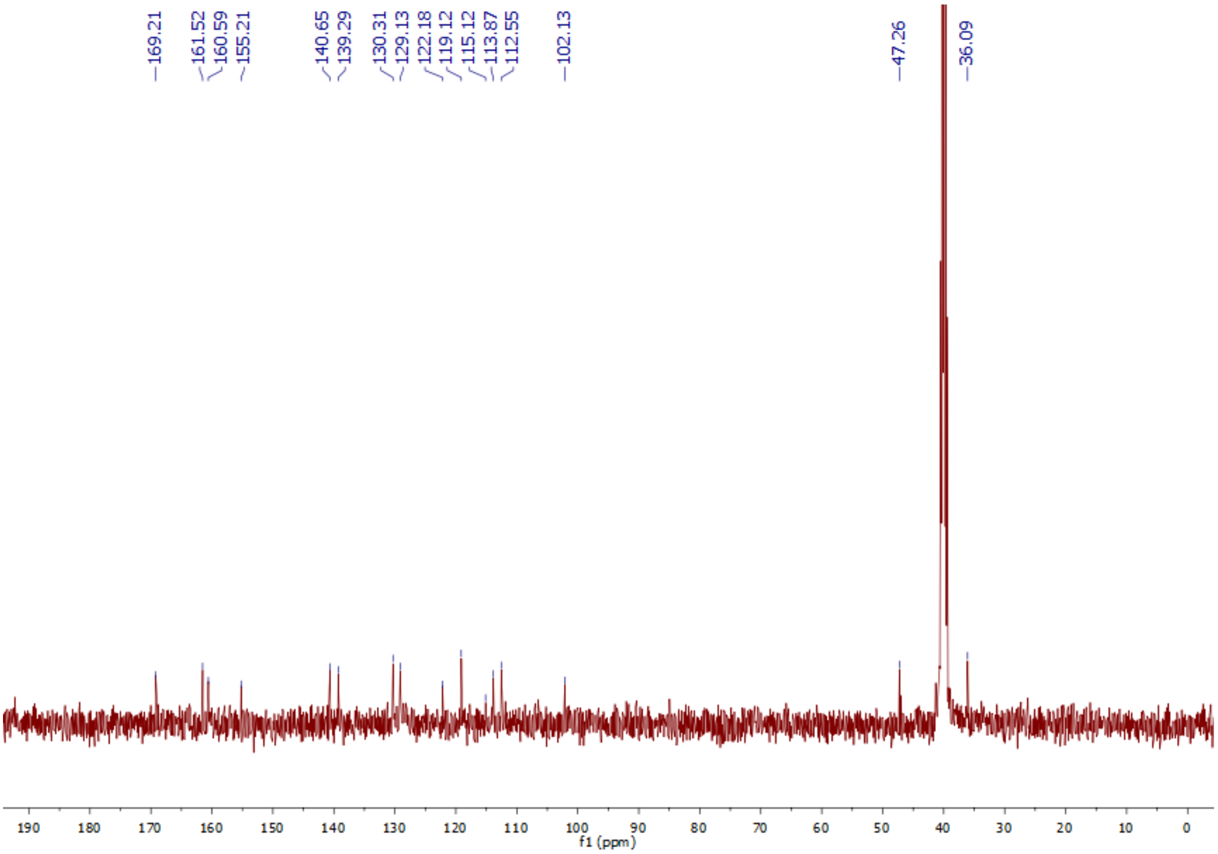
169.21
161.52
160.59
155.21
140.65
139.29
130.31
129.13
122.18
119.12
115.12
113.87
112.55
102.13
47.26
36.09
f1 (ppm)

6c

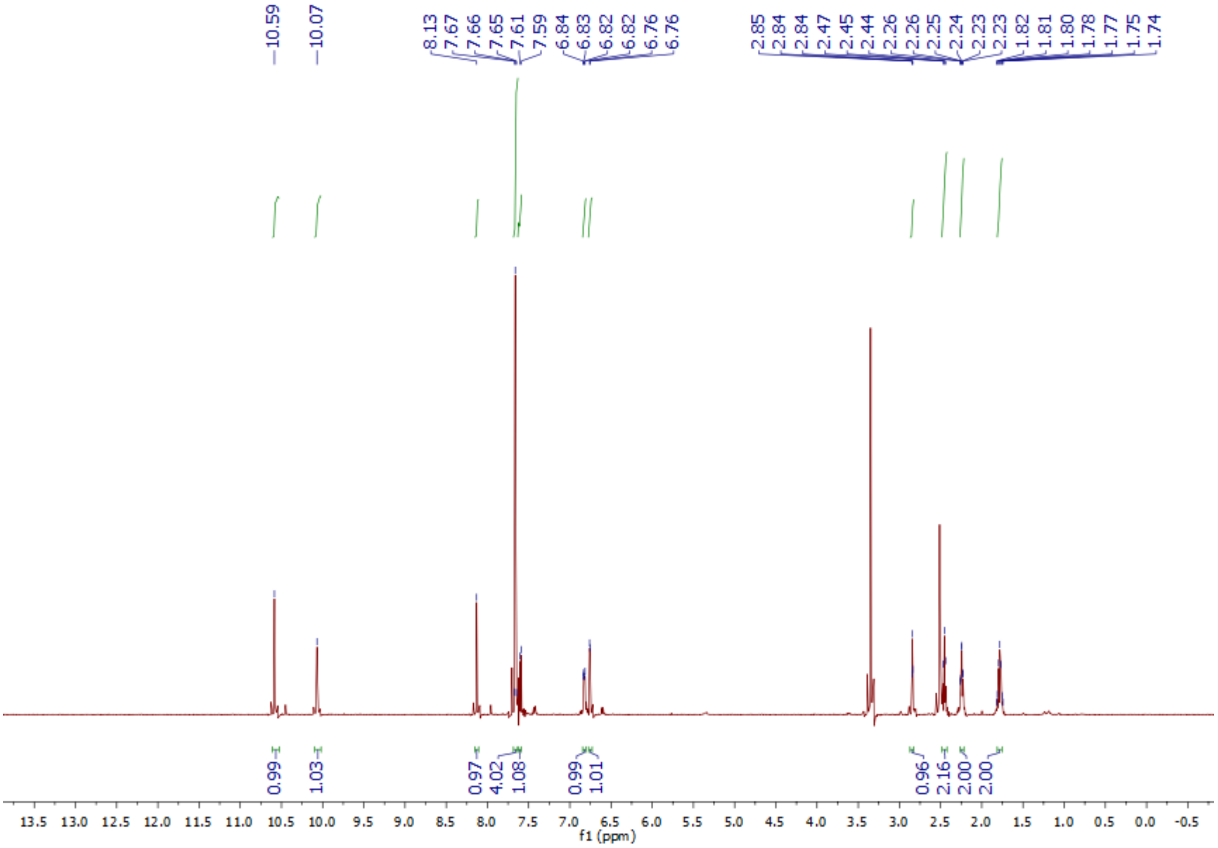

10.59
10.07
8.13
7.67
7.66
7.65
7.61
7.59
6.84
6.83
6.82
6.76
6.76
2.85
2.84
2.84
2.47
2.45
2.44
2.26
2.26
2.25
2.24
2.23
2.23
1.82
1.81
1.80
1.78
1.77
1.75
1.74
0.99
1.03
0.97
4.02
1.08
0.99
1.01
0.96
2.16
2.00
2.00
f1 (ppm)

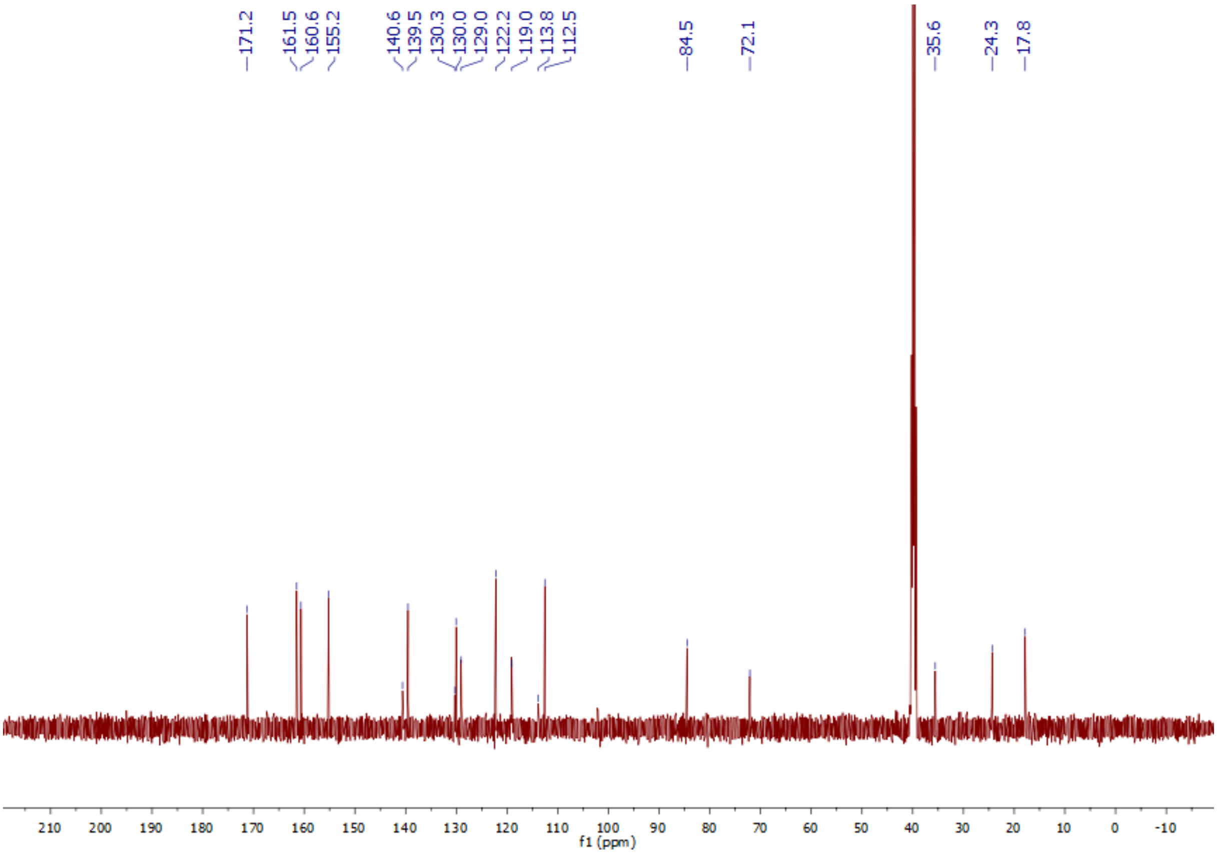

171.2
161.5
160.6
155.2
140.6
139.5
130.3
130.0
129.0
122.2
119.0
113.8
112.5
84.5
72.1
35.6
24.3
17.8
f1 (ppm)

7

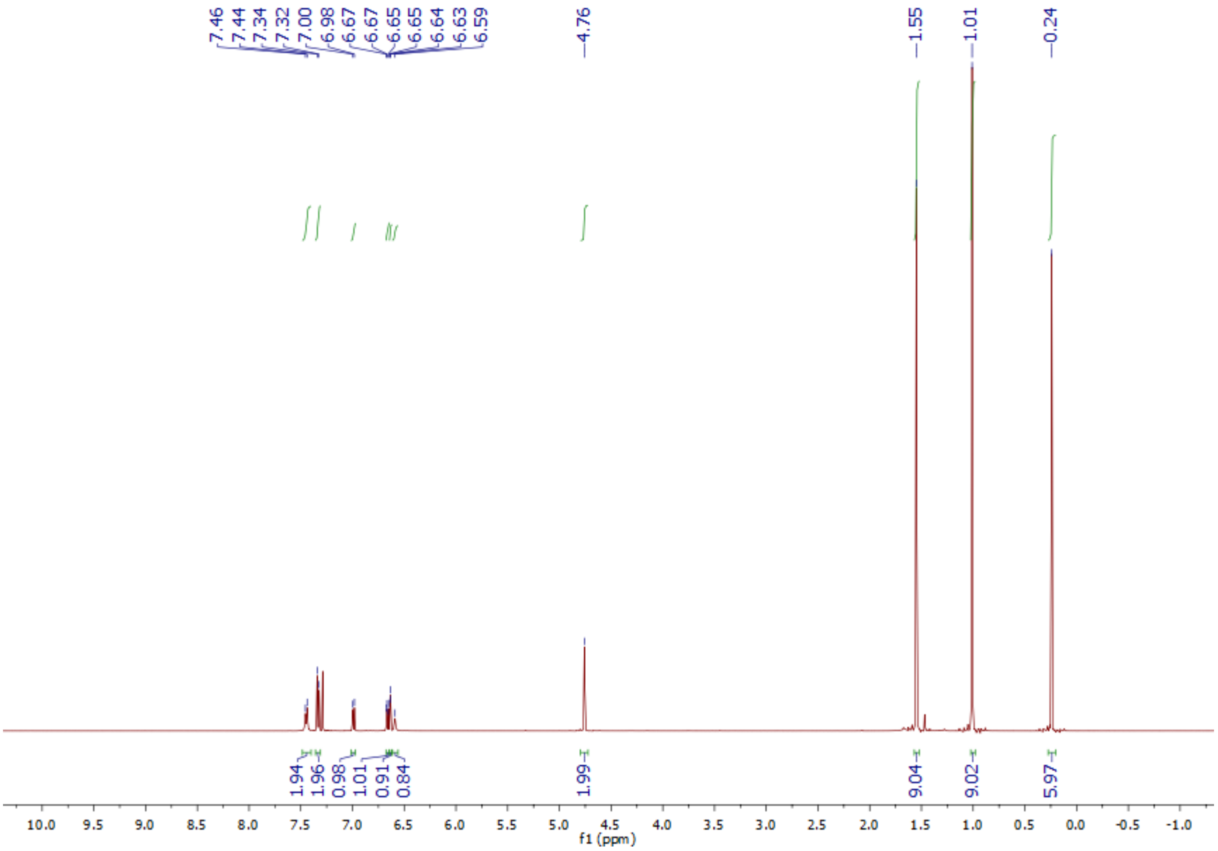
7.46
7.44
7.34
7.32
7.00
6.98
6.67
6.67
6.65
6.65
6.64
6.63
6.59
4.76
1.55
1.01
0.24
1.94
1.96
0.98
1.01
0.91
0.84
1.99
9.04
9.02
5.97
f1 (ppm)

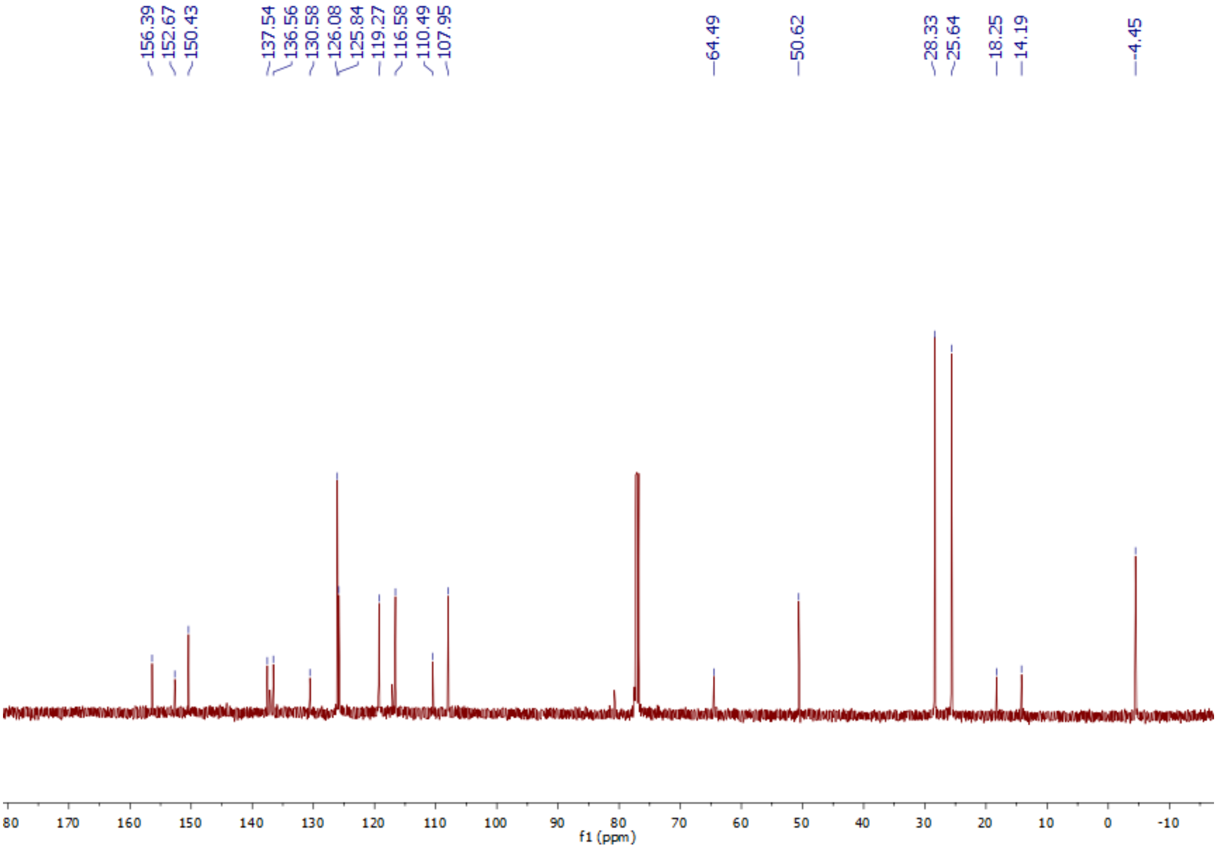
156.39
152.67
150.43
137.54
136.56
130.58
126.08
125.84
119.27
116.58
110.49
107.95
64.49
50.62
28.33
25.64
18.25
14.19
-4.45
f1 (ppm)

8

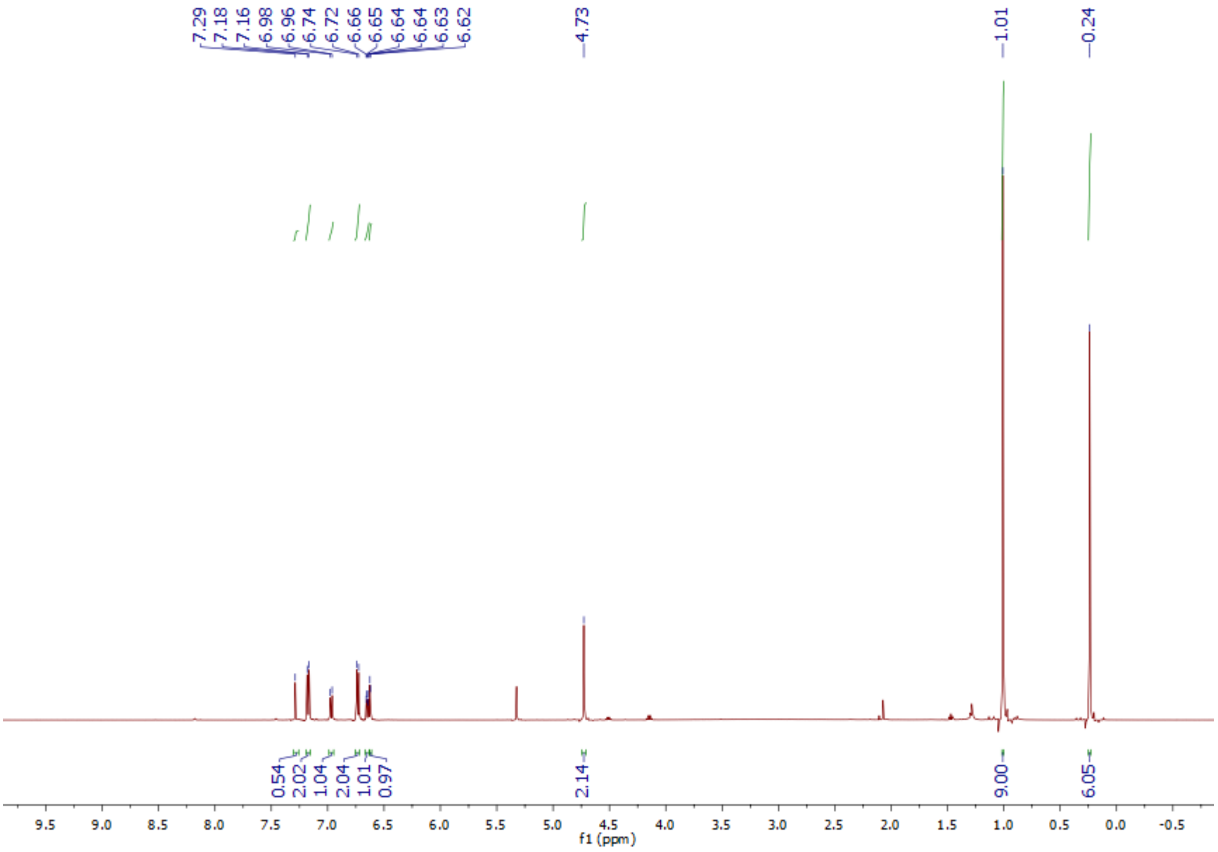
7.29
7.18
7.16
6.98
6.96
6.74
6.72
6.66
6.65
6.64
6.64
6.63
6.62
4.73
1.01
0.24
0.54
2.02
1.04
2.04
1.01
0.97
2.14
9.00
6.05
9.5 9.0 8.5 8.0 7.5 7.0 6.5 6.0 5.5 5.0 4.5 4.0 3.5 3.0 2.5 2.0 1.5 1.0 0.5 0.0 -0.5
f1 (ppm)

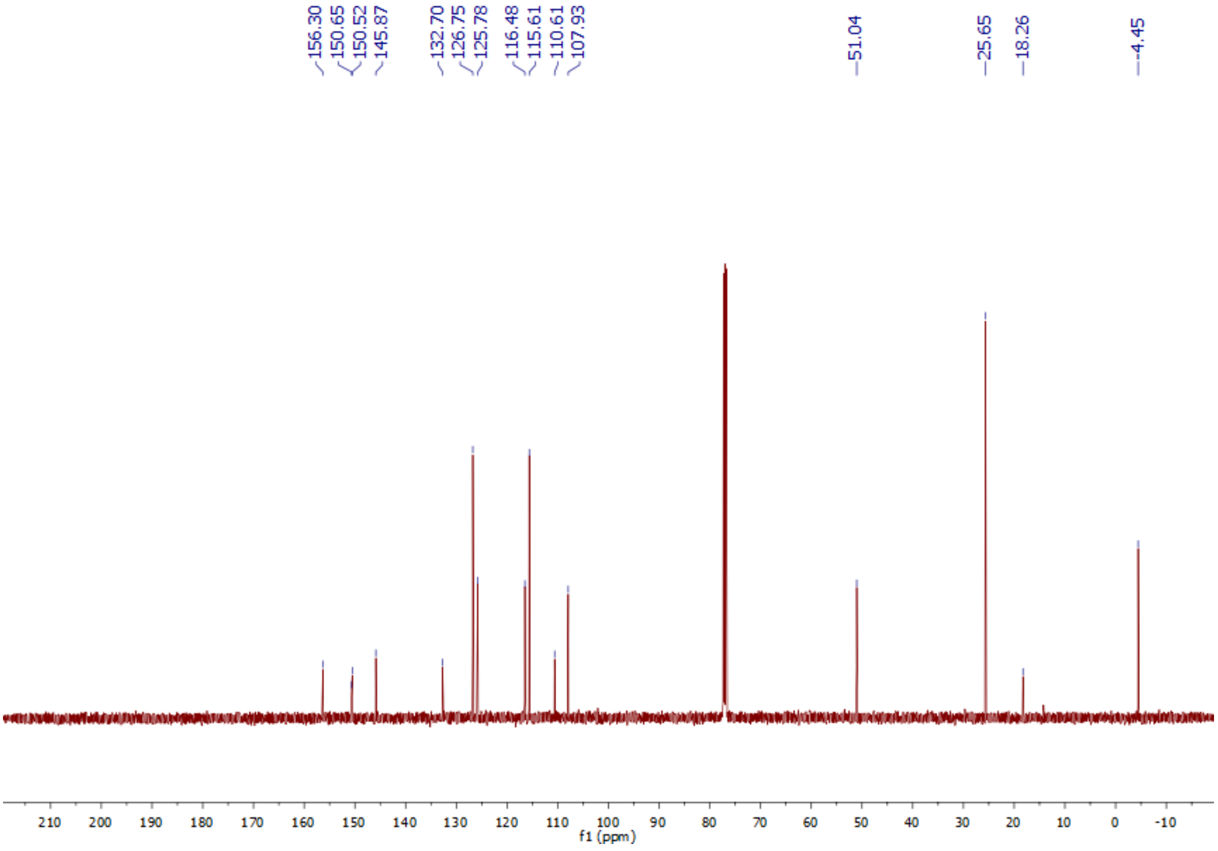
156.30
150.65
150.52
145.87
132.70
126.75
125.78
116.48
115.61
110.61
107.93
51.04
25.65
18.26
-4.45
210 200 190 180 170 160 150 140 130 120 110 100 90 80 70 60 50 40 30 20 10 0 -10
f1 (ppm)

9

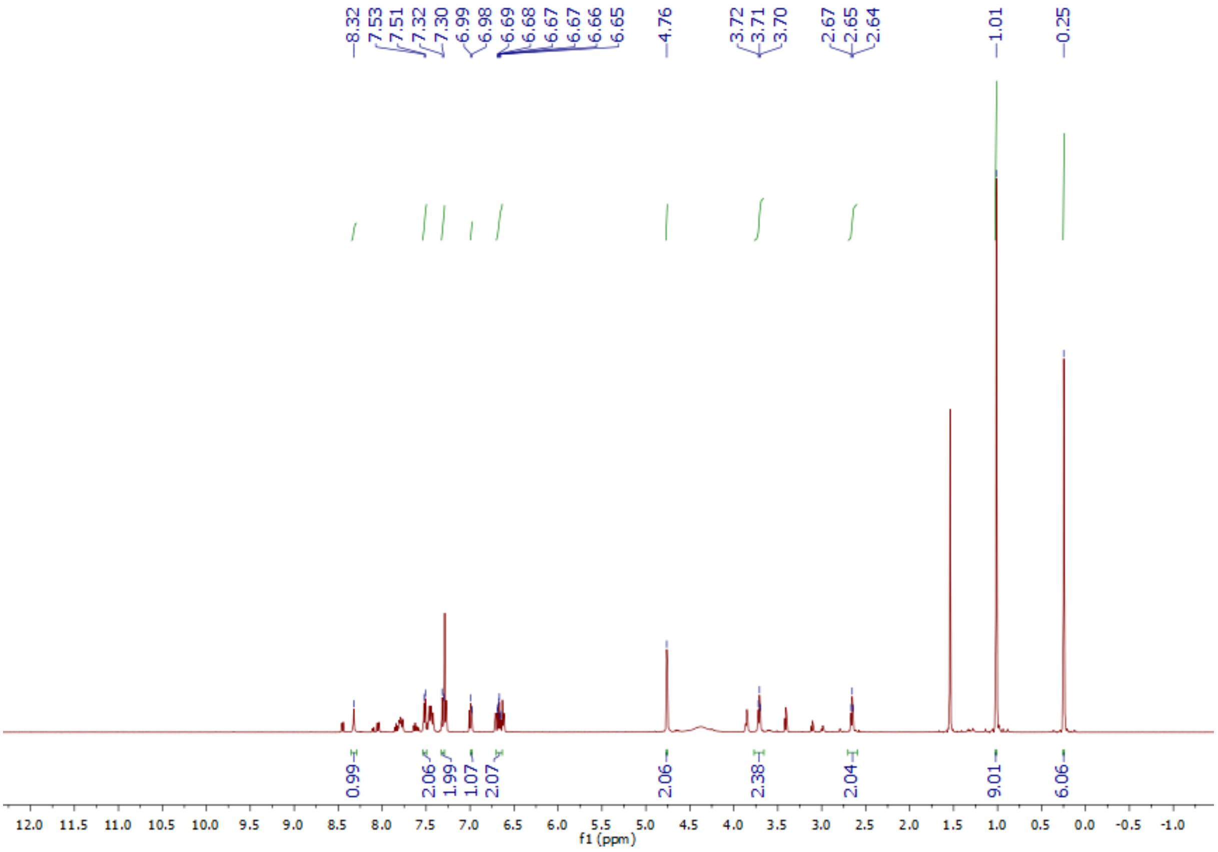
8.32
7.53
7.51
7.32
7.30
6.99
6.98
6.69
6.68
6.67
6.67
6.66
6.65
4.76
3.72
3.71
3.70
2.67
2.65
2.64
1.01
0.25
0.99
2.06
1.99
1.07
2.07
2.06
2.38
2.04
9.01
6.06
12.0 11.5 11.0 10.5 10.0 9.5 9.0 8.5 8.0 7.5 7.0 6.5 6.0 5.5 5.0 4.5 4.0 3.5 3.0 2.5 2.0 1.5 1.0 0.5 0.0 -0.5 -1.0
f1 (ppm)

10

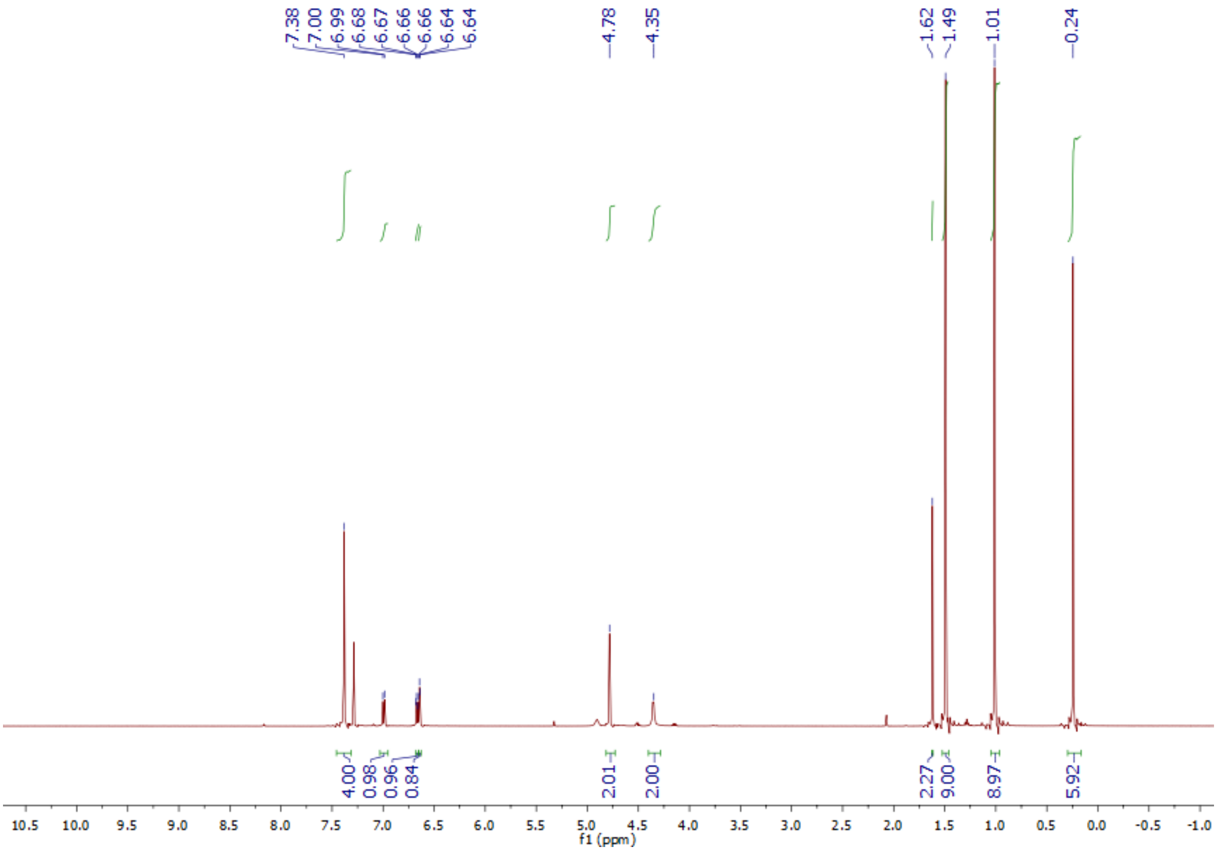
7.38
7.00
6.99
6.68
6.67
6.66
6.66
6.64
6.64
4.78
4.35
1.62
1.49
1.01
0.24
4.00
0.98
0.96
0.84
2.01
2.00
2.27
9.00
8.97
5.92
10.5 10.0 9.5 9.0 8.5 8.0 7.5 7.0 6.5 6.0 5.5 5.0 4.5 4.0 3.5 3.0 2.5 2.0 1.5 1.0 0.5 0.0 -0.5 -1.0
f1 (ppm)

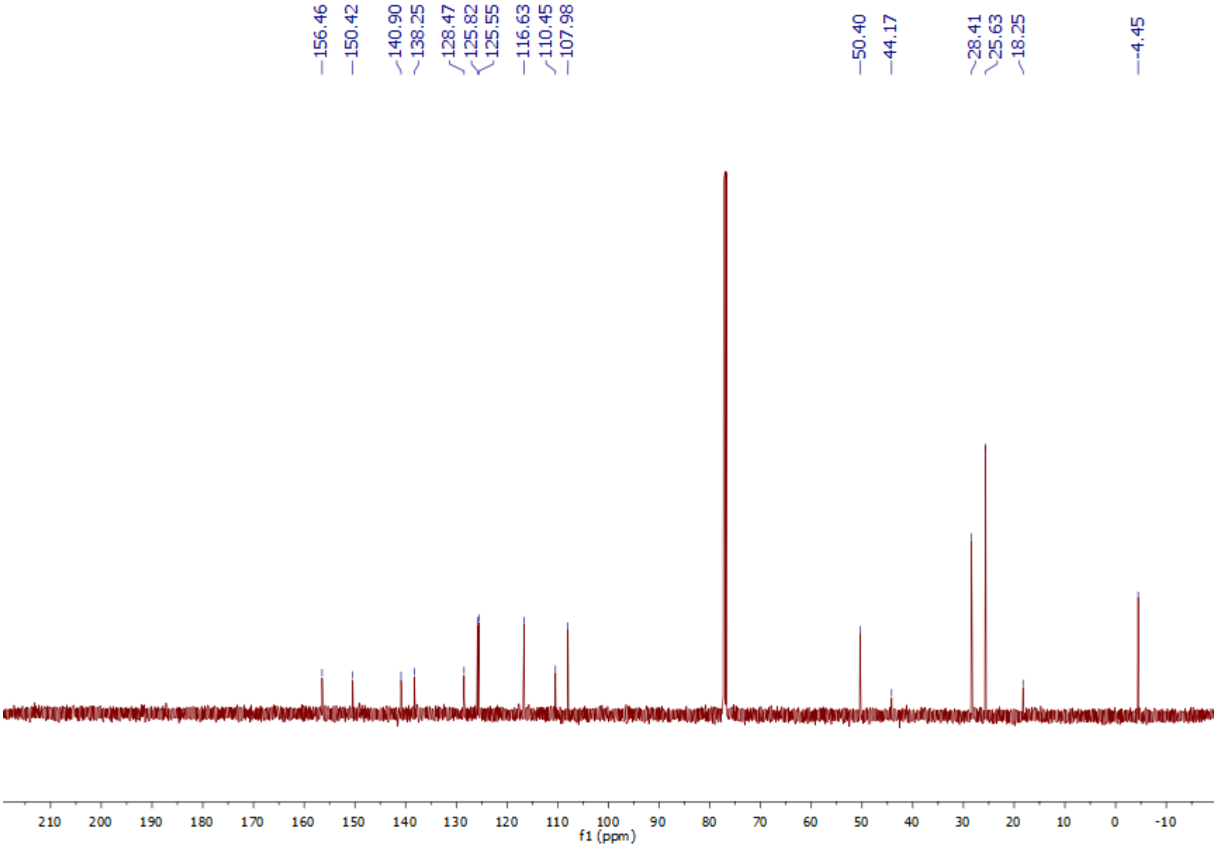
156.46
150.42
140.90
138.25
128.47
125.82
125.55
116.63
110.45
107.98
50.40
44.17
28.41
25.63
18.25
-4.45
f1 (ppm)

11

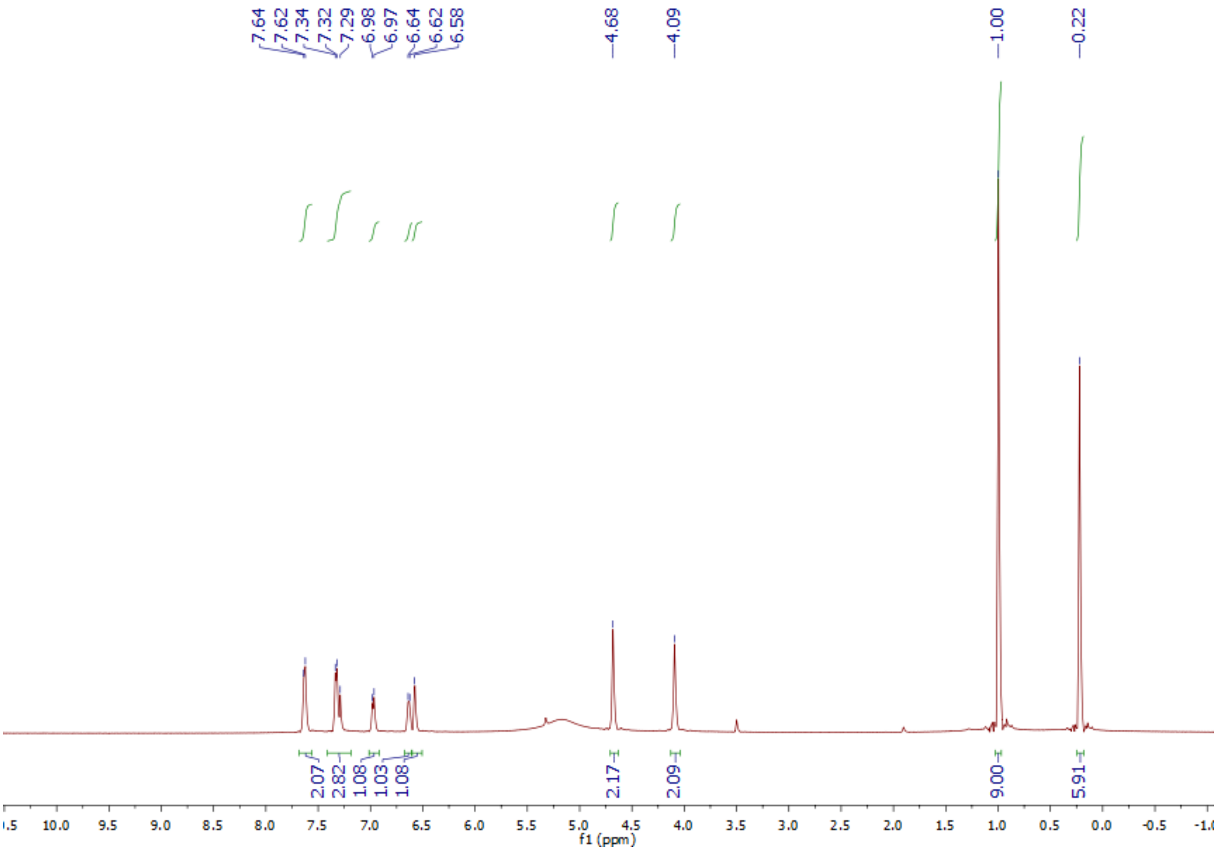
7.64
7.62
7.34
7.32
7.29
6.98
6.97
6.64
6.62
6.58
4.68
4.09
1.00
0.22
2.07
2.82
1.08
1.03
1.08
2.17
2.09
9.00
5.91
f1 (ppm)

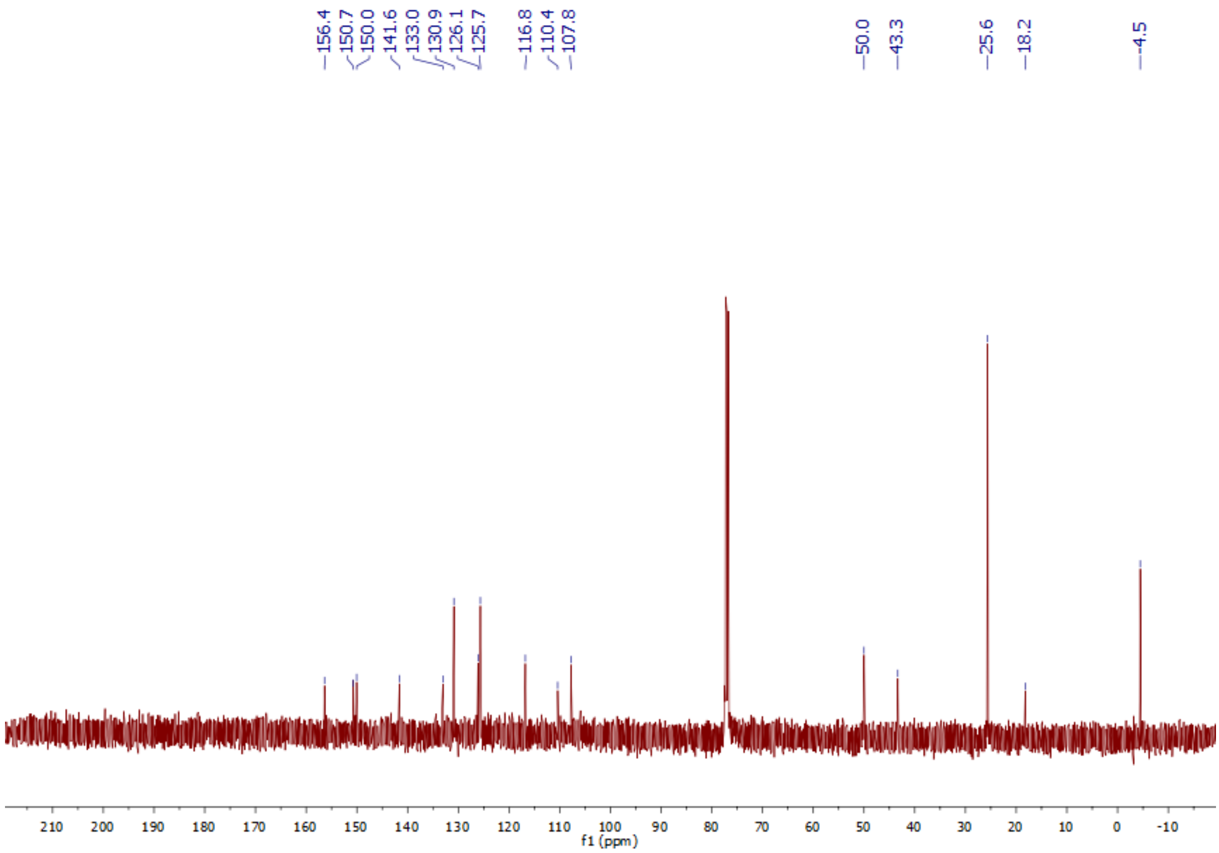
156.4
150.7
150.0
141.6
133.0
130.9
126.1
125.7
116.8
110.4
107.8
50.0
43.3
25.6
18.2
-4.5
210 200 190 180 170 160 150 140 130 120 110 100 90 80 70 60 50 40 30 20 10 0 -10
f1 (ppm)

12

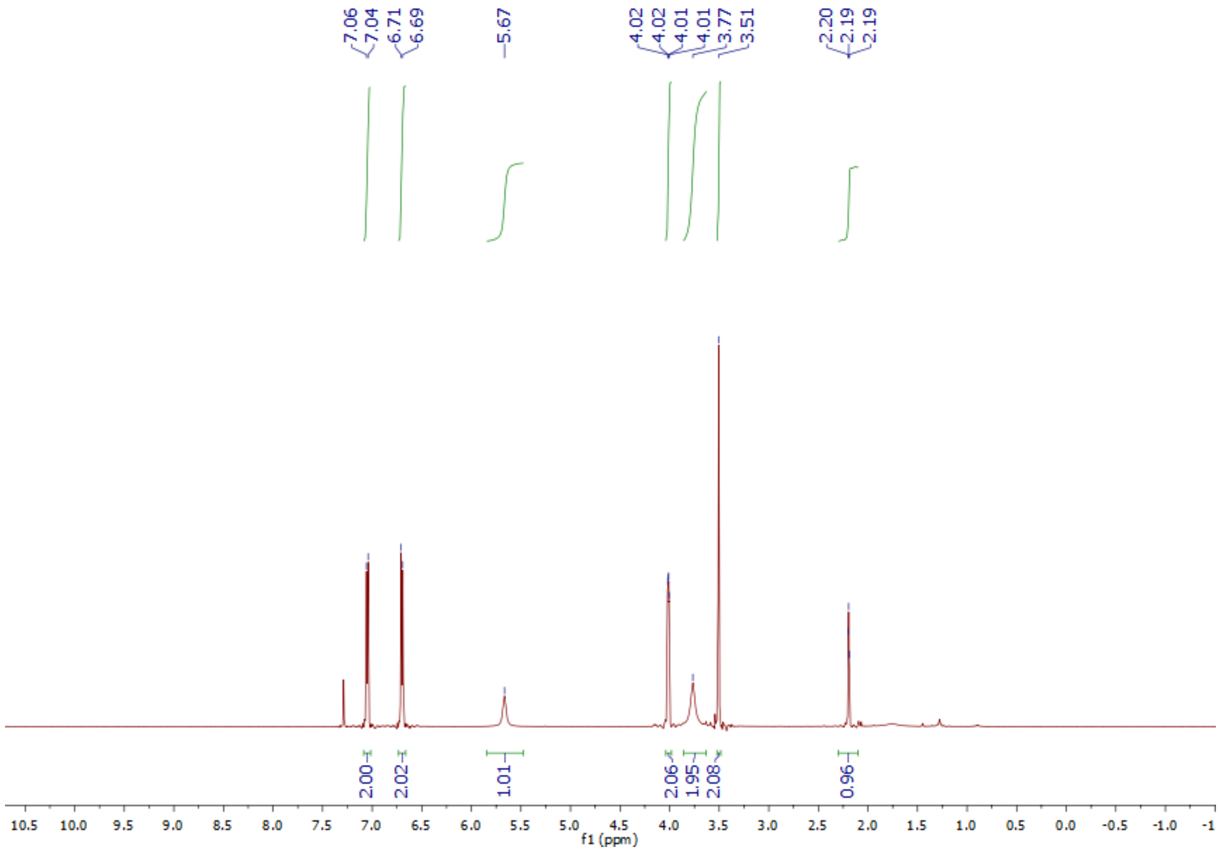
7.06
7.04
6.71
6.69
5.67
4.02
4.02
4.01
4.01
3.77
3.51
2.20
2.19
2.19
2.00
2.02
1.01
2.06
1.95
2.08
0.96
10.5 10.0 9.5 9.0 8.5 8.0 7.5 7.0 6.5 6.0 5.5 5.0 4.5 4.0 3.5 3.0 2.5 2.0 1.5 1.0 0.5 0.0 -0.5 -1.0 -1
f1 (ppm)

13

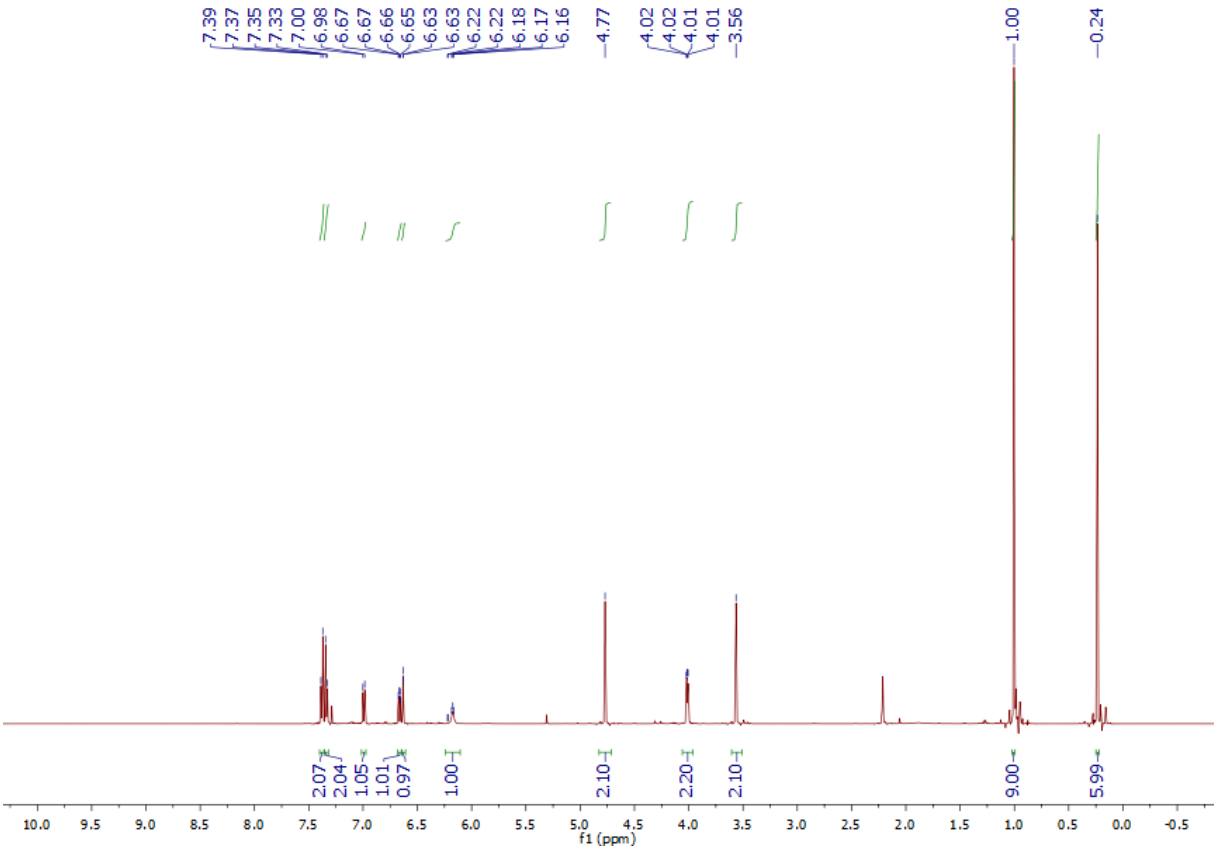
7.39
7.37
7.35
7.33
7.00
6.98
6.67
6.67
6.66
6.65
6.63
6.63
6.22
6.22
6.18
6.17
6.16
4.77
4.02
4.02
4.01
4.01
3.56
1.00
0.24
2.07
2.04
1.05
1.01
0.97
1.00
2.10
2.20
2.10
9.00
5.99
f1 (ppm)

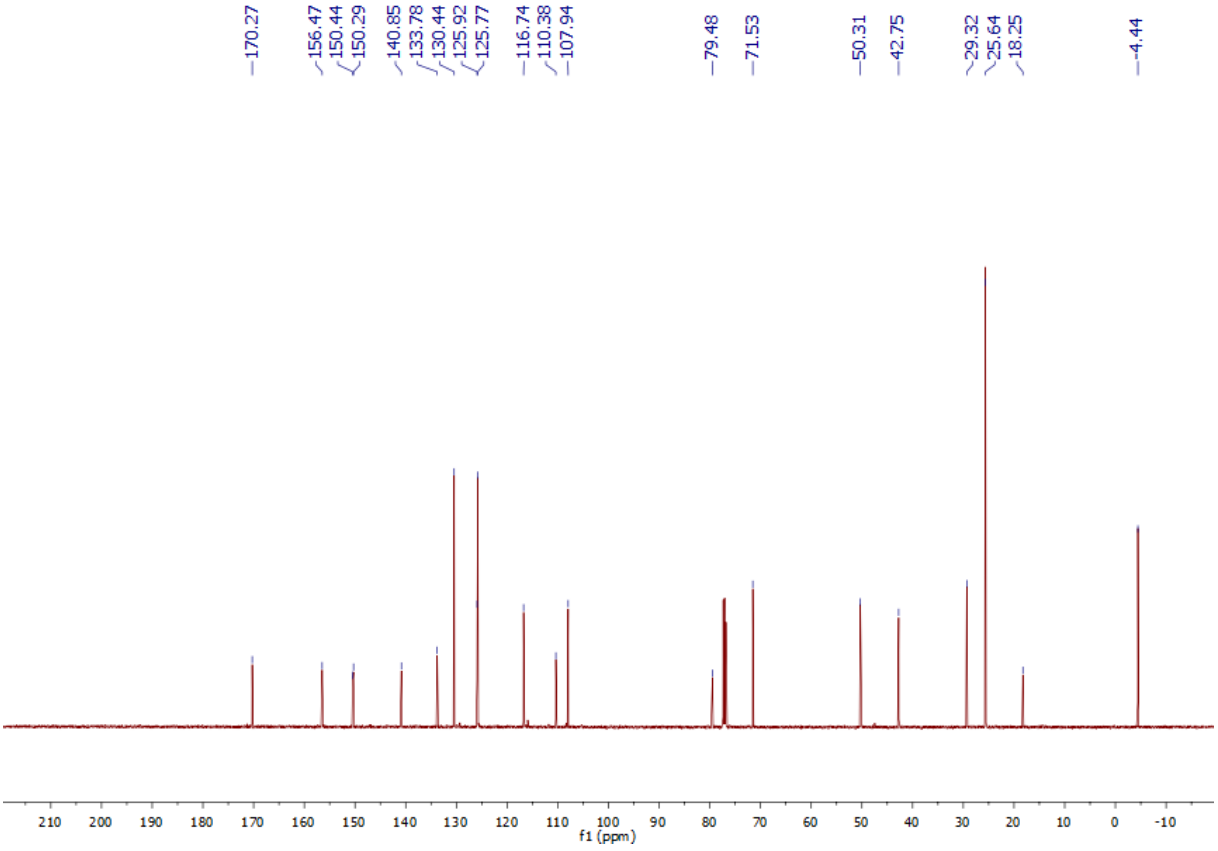
170.27
156.47
150.44
150.29
140.85
133.78
130.44
125.92
125.77
116.74
110.38
107.94
79.48
71.53
50.31
42.75
29.32
25.64
18.25
-4.44
f1 (ppm)

14

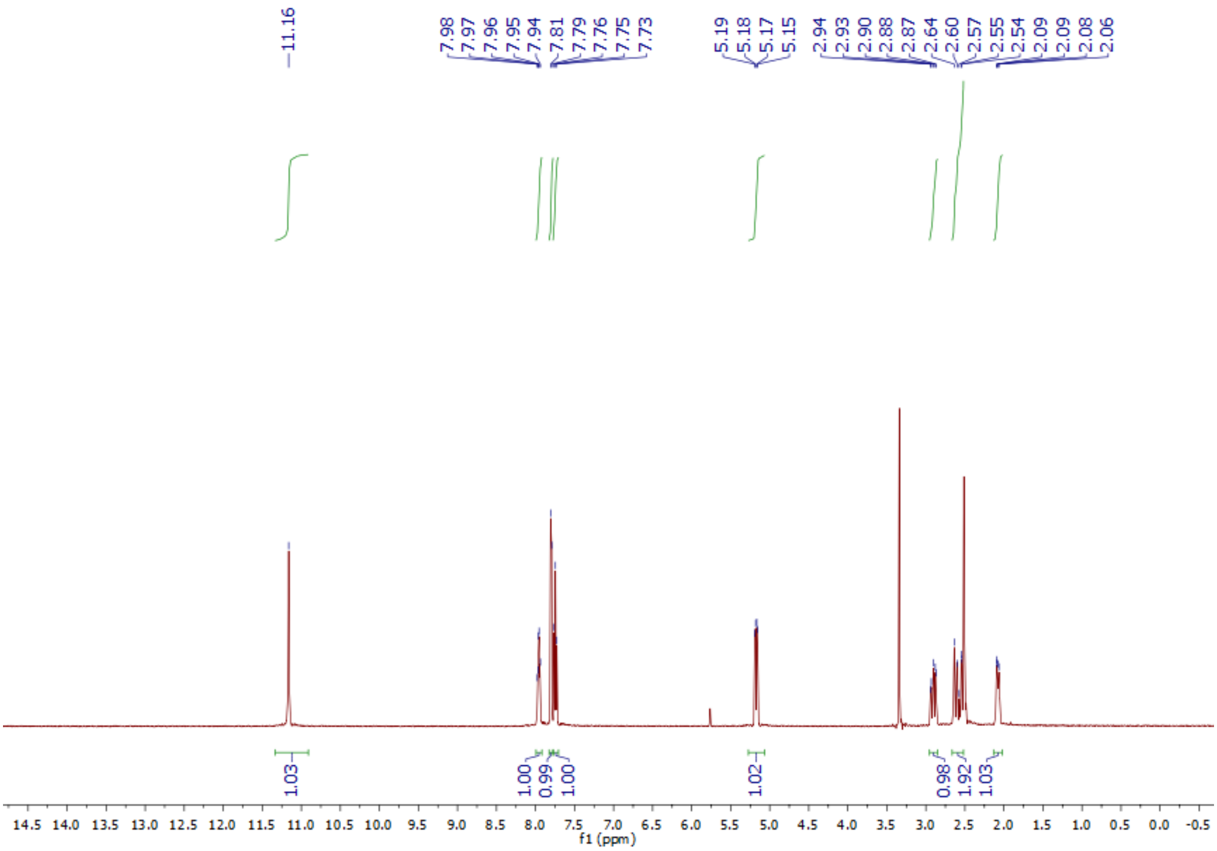
11.16
7.98
7.97
7.96
7.95
7.94
7.81
7.79
7.76
7.75
7.73
5.19
5.18
5.17
5.15
2.94
2.93
2.90
2.88
2.87
2.64
2.60
2.57
2.55
2.54
2.09
2.09
2.08
2.06
1.03
1.00
0.99
1.00
1.02
0.98
1.92
1.03
f1 (ppm)

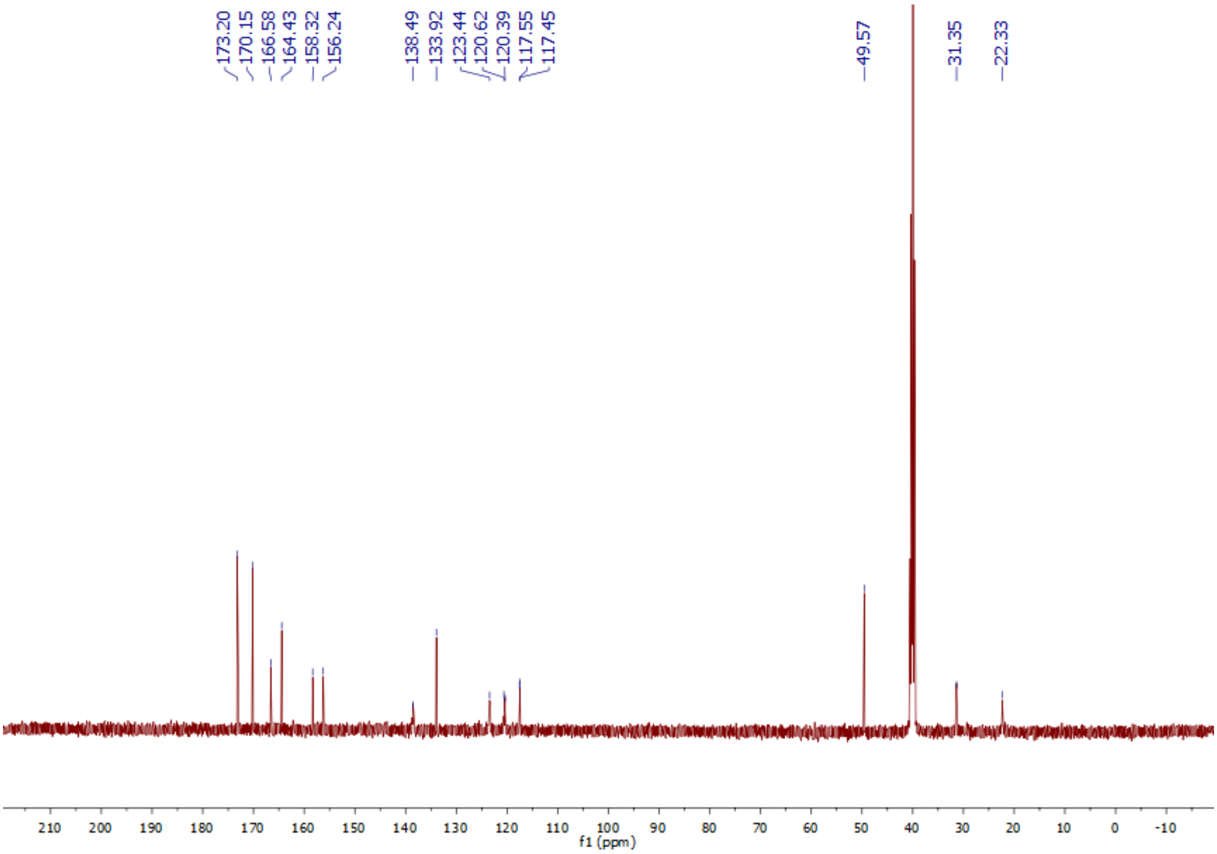
173.20
170.15
166.58
164.43
158.32
156.24
138.49
133.92
123.44
120.62
120.39
117.55
117.45
49.57
31.35
22.33
f1 (ppm)

15a

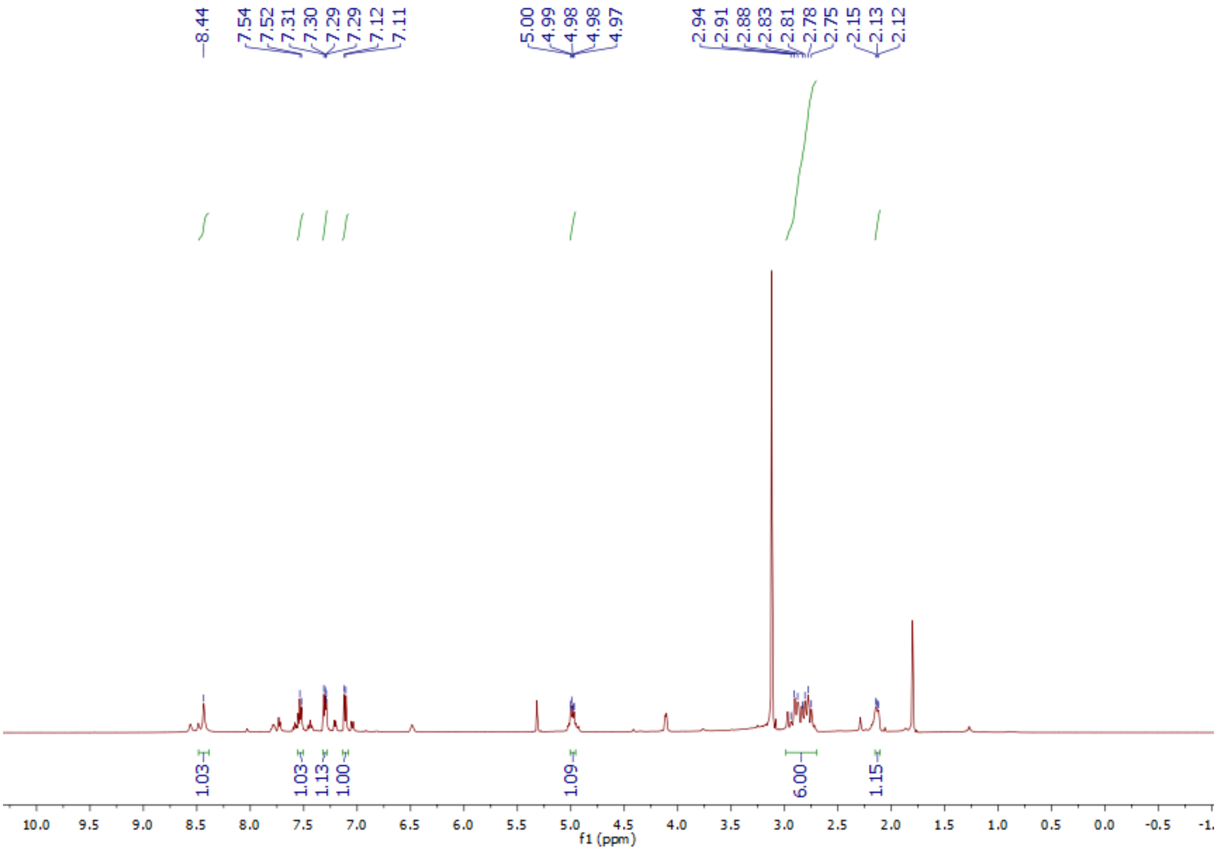

8.44
7.54
7.52
7.31
7.30
7.29
7.29
7.12
7.11
5.00
4.99
4.98
4.98
4.97
2.94
2.91
2.88
2.83
2.81
2.78
2.75
2.15
2.13
2.12
1.03
1.03
1.13
1.00
1.09
6.00
1.15
f1 (ppm)

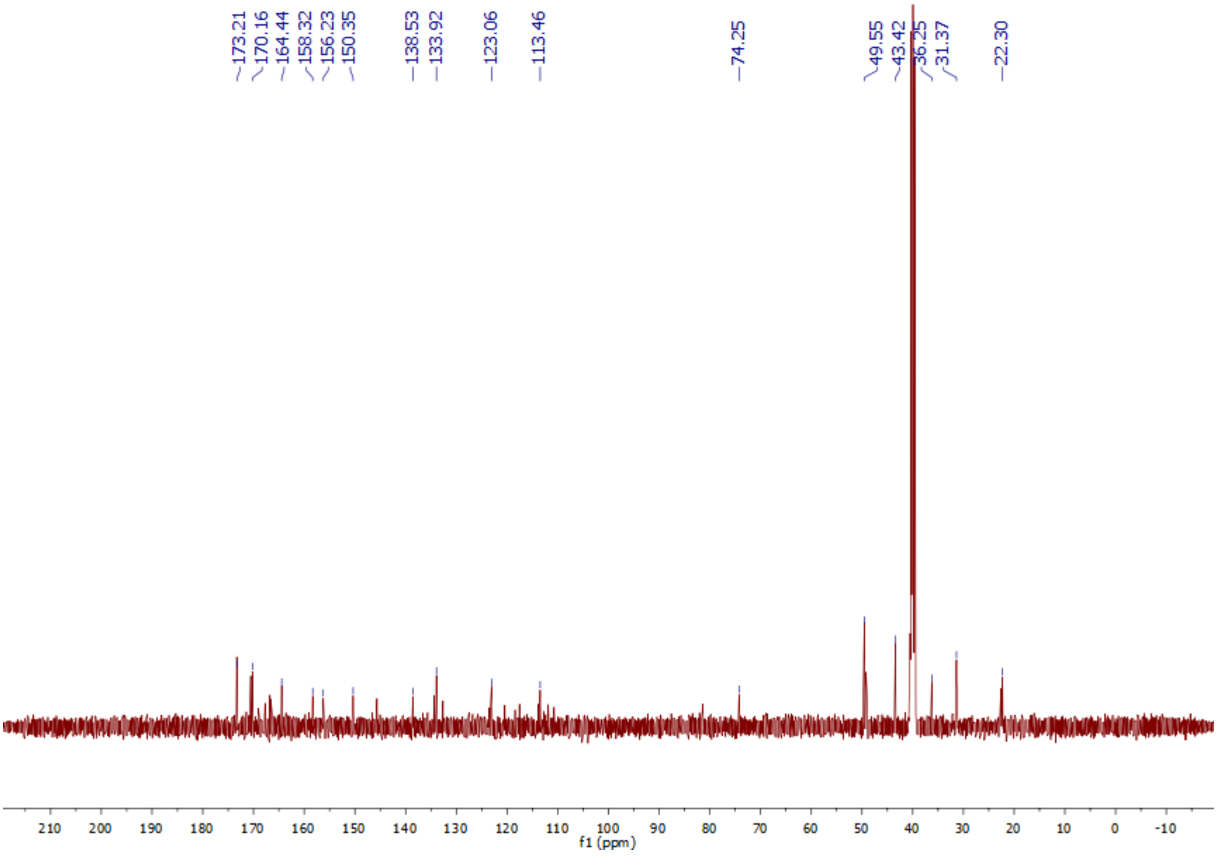

173.21
170.16
164.44
158.32
156.23
150.35
138.53
133.92
123.06
113.46
74.25
49.55
43.42
36.25
31.37
22.30
f1 (ppm)

15b

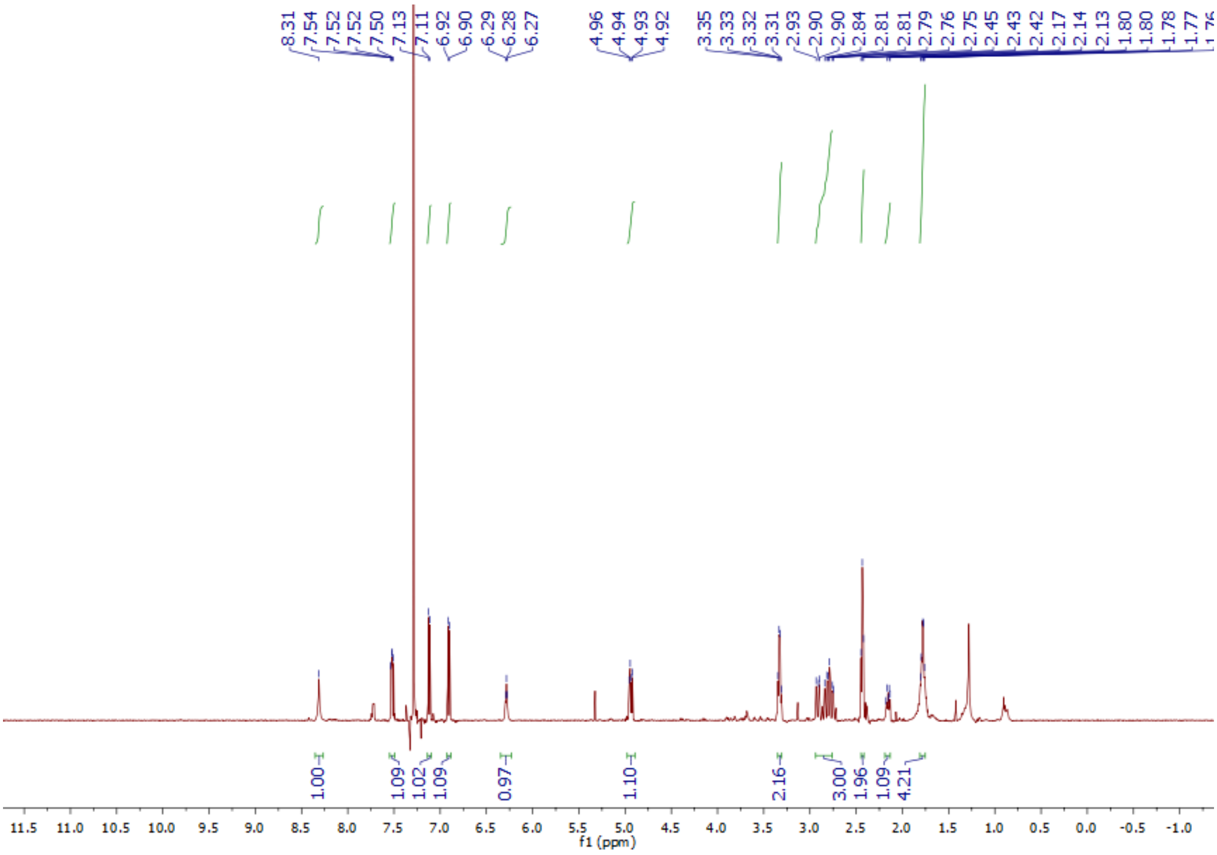

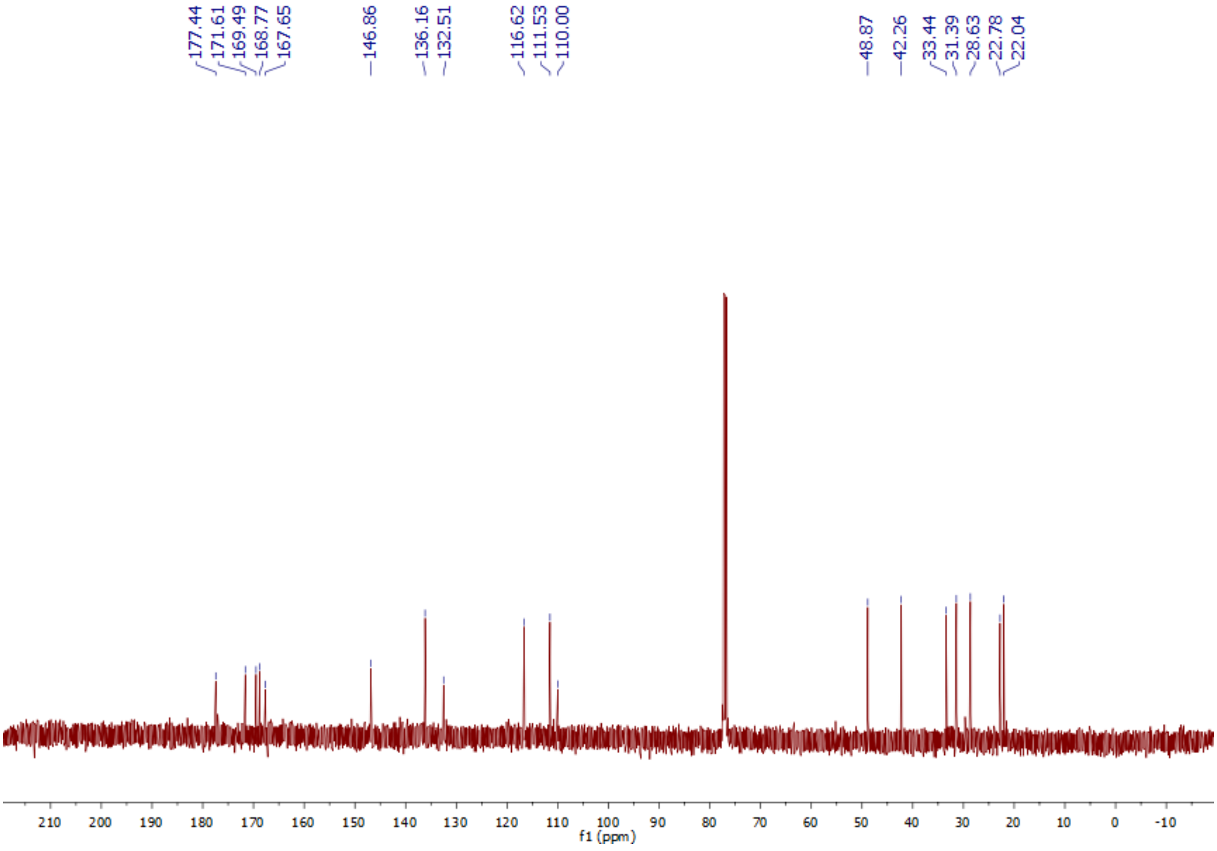

15c

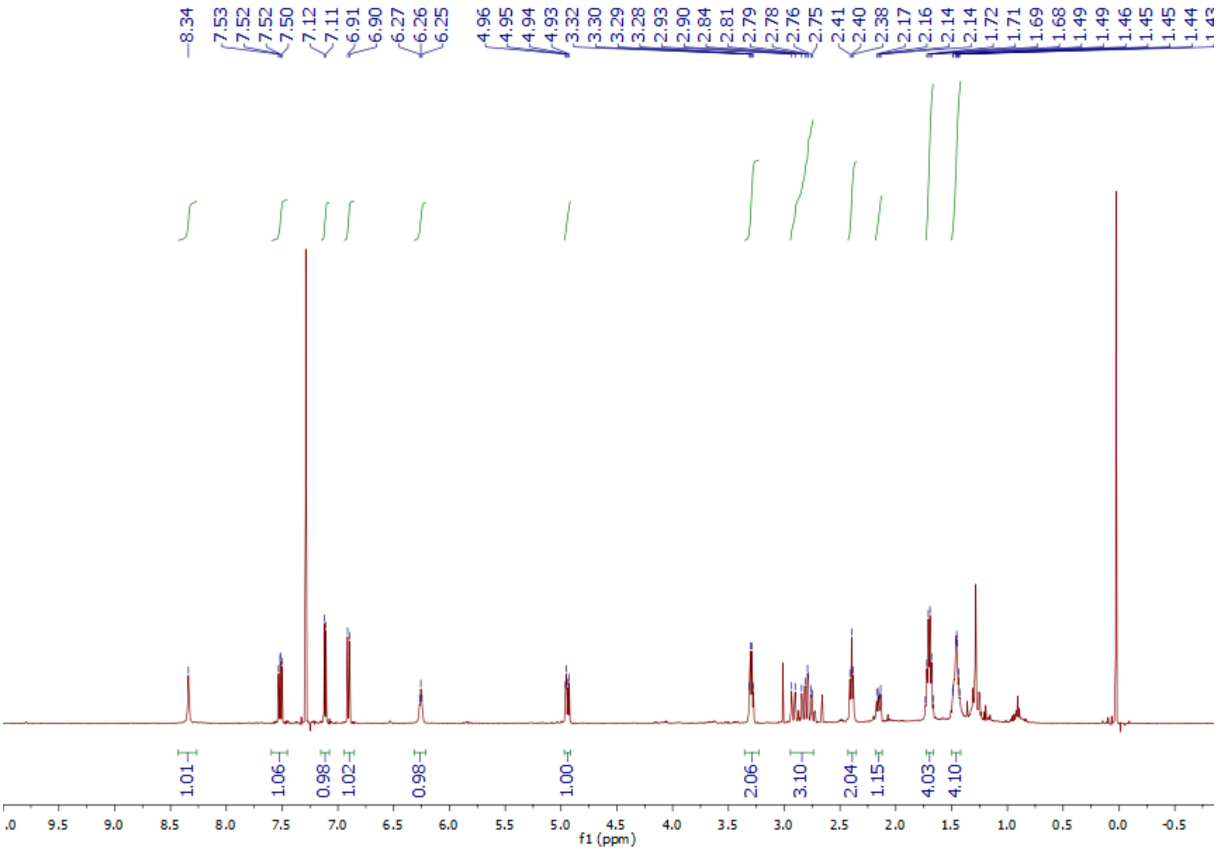

15d

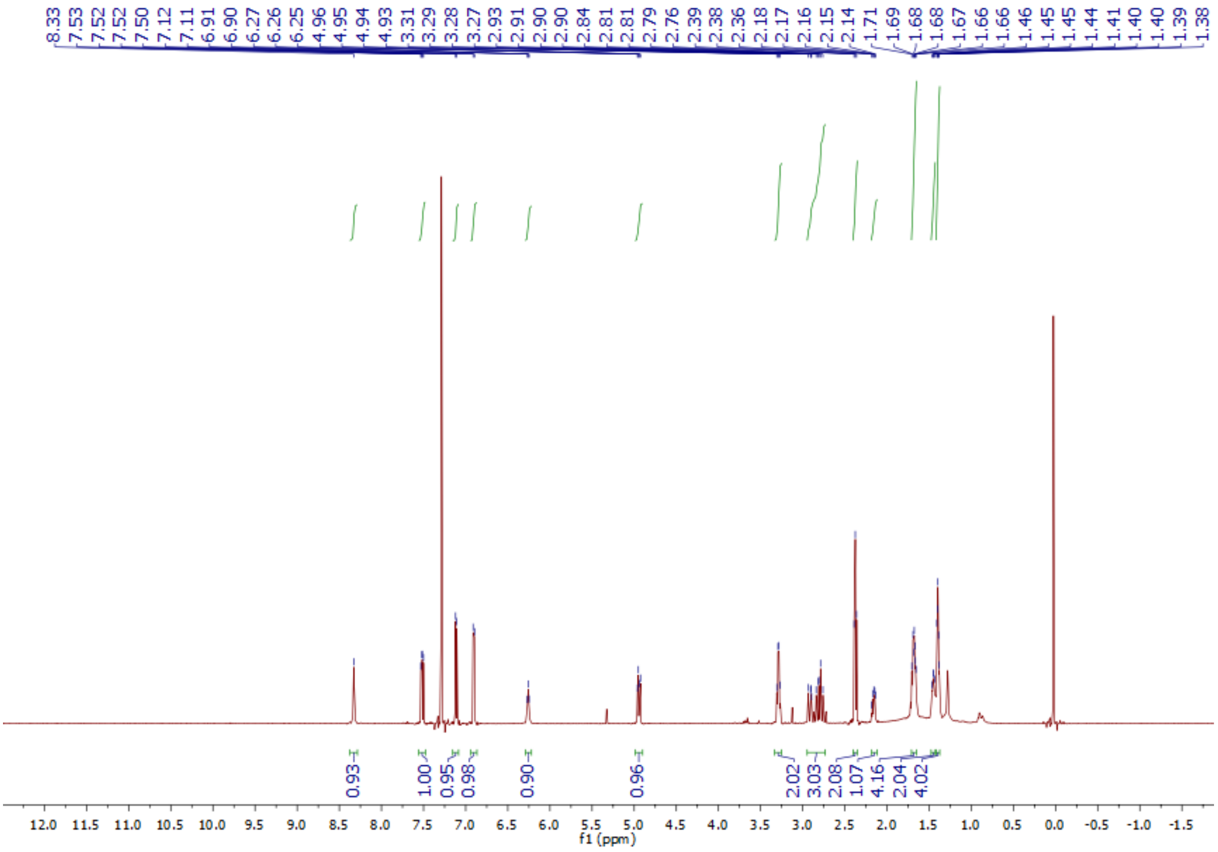
8.33 7.53 7.52 7.52 7.50 7.12 7.11 6.91 6.90 6.27 6.26 6.25 4.96 4.95 4.94 4.93 3.31 3.29 3.28 3.27 2.93 2.91 2.90 2.90 2.84 2.81 2.81 2.79 2.76 2.39 2.38 2.36 2.18 2.17 2.16 2.15 2.14 1.71 1.69 1.68 1.68 1.67 1.66 1.66 1.46 1.45 1.45 1.44 1.41 1.40 1.40 1.39 1.38
0.93 1.00 0.95 0.98 0.90 0.96 2.02 3.03 2.08 1.07 4.16 2.04 4.02
f1 (ppm)

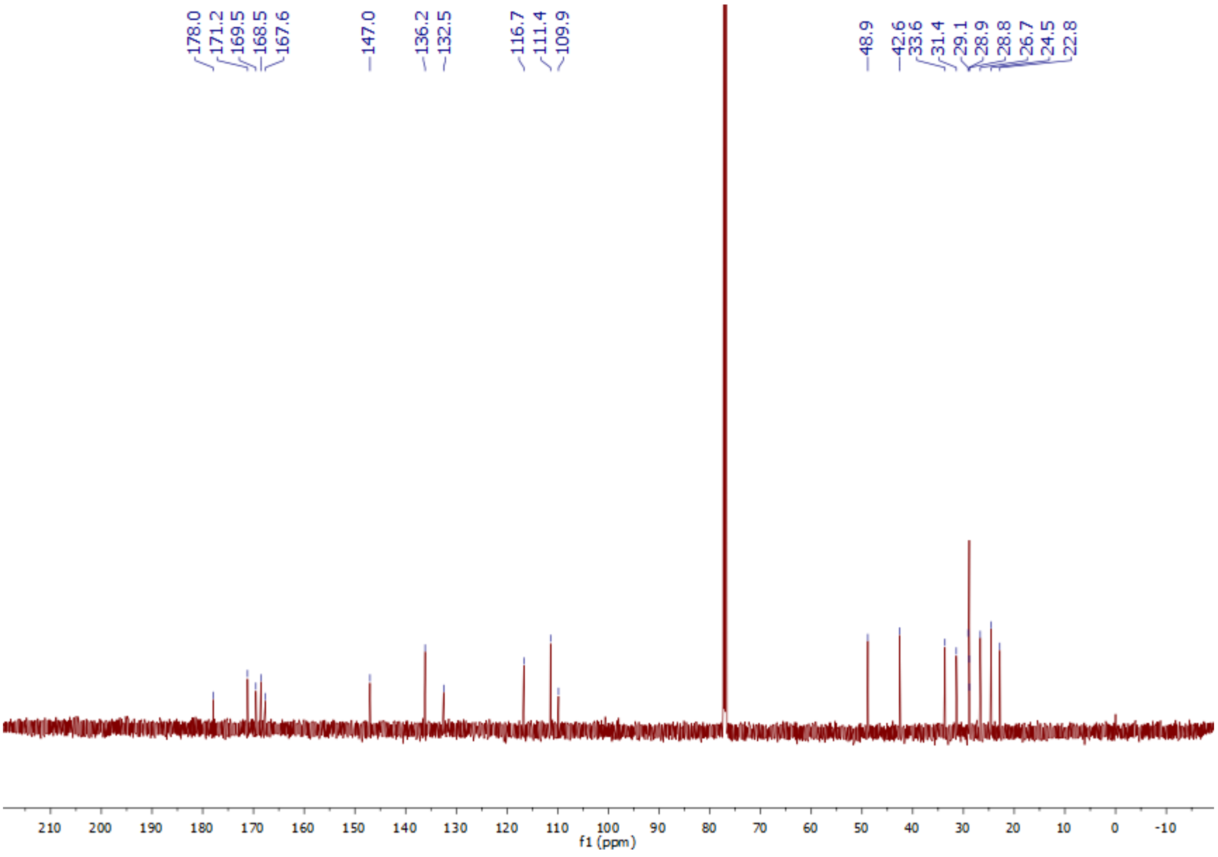
178.0 171.2 169.5 168.5 167.6 147.0 136.2 132.5 116.7 111.4 109.9 48.9 42.6 33.6 31.4 29.1 28.9 28.8 26.7 24.5 22.8
f1 (ppm)

15e

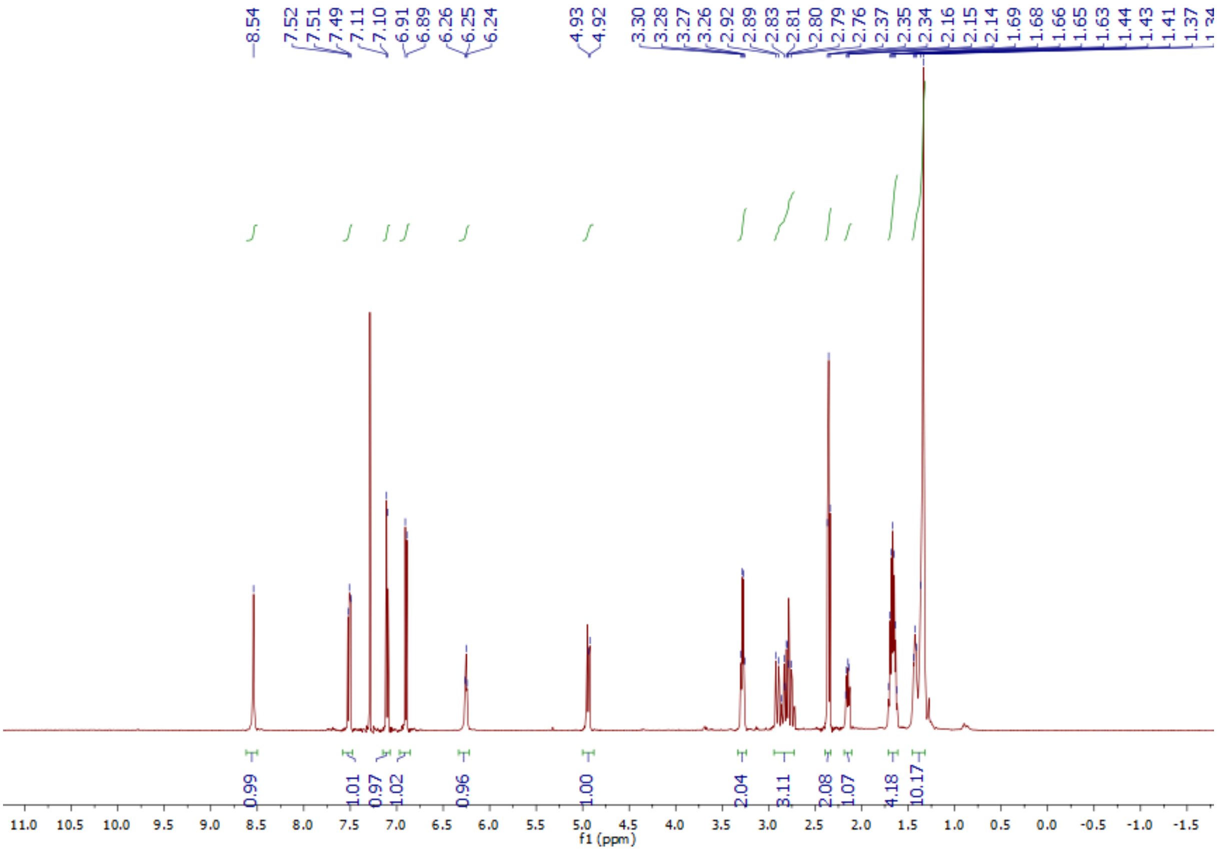

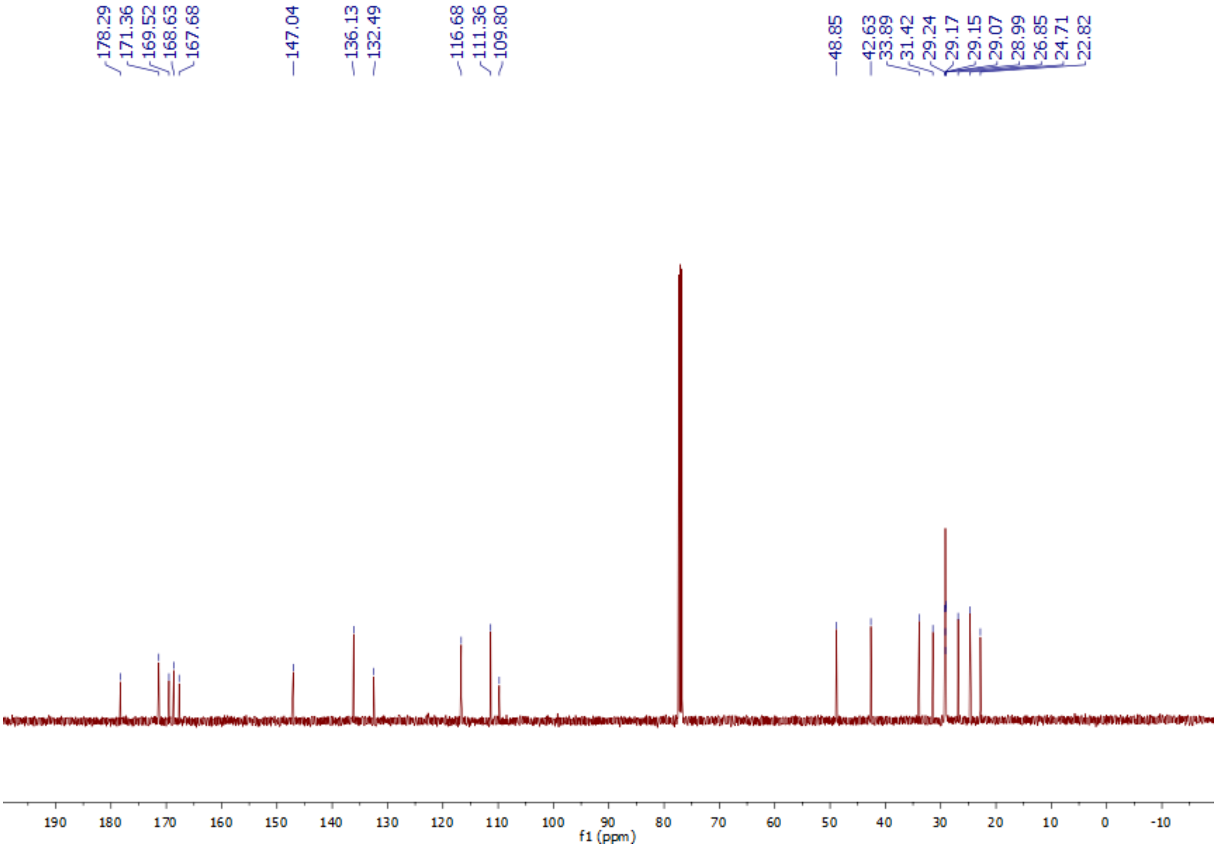

16

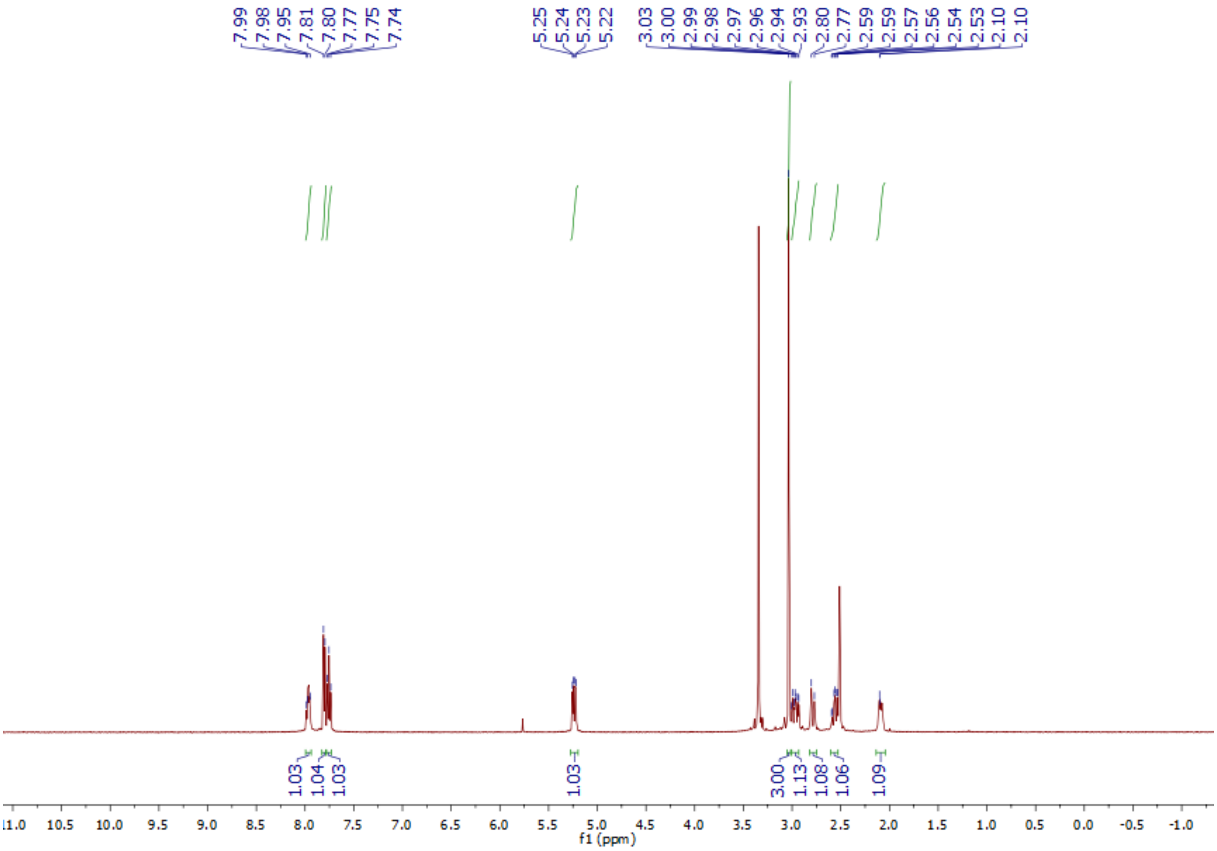
7.99 7.98 7.95 7.81 7.80 7.77 7.75 7.74
5.25 5.24 5.23 5.22
3.03 3.00 2.99 2.98 2.97 2.96 2.94 2.93 2.80 2.77 2.59 2.59 2.57 2.56 2.54 2.53 2.10 2.10
1.03 1.04 1.03 1.03 3.00 1.13 1.08 1.06 1.09
f1 (ppm)

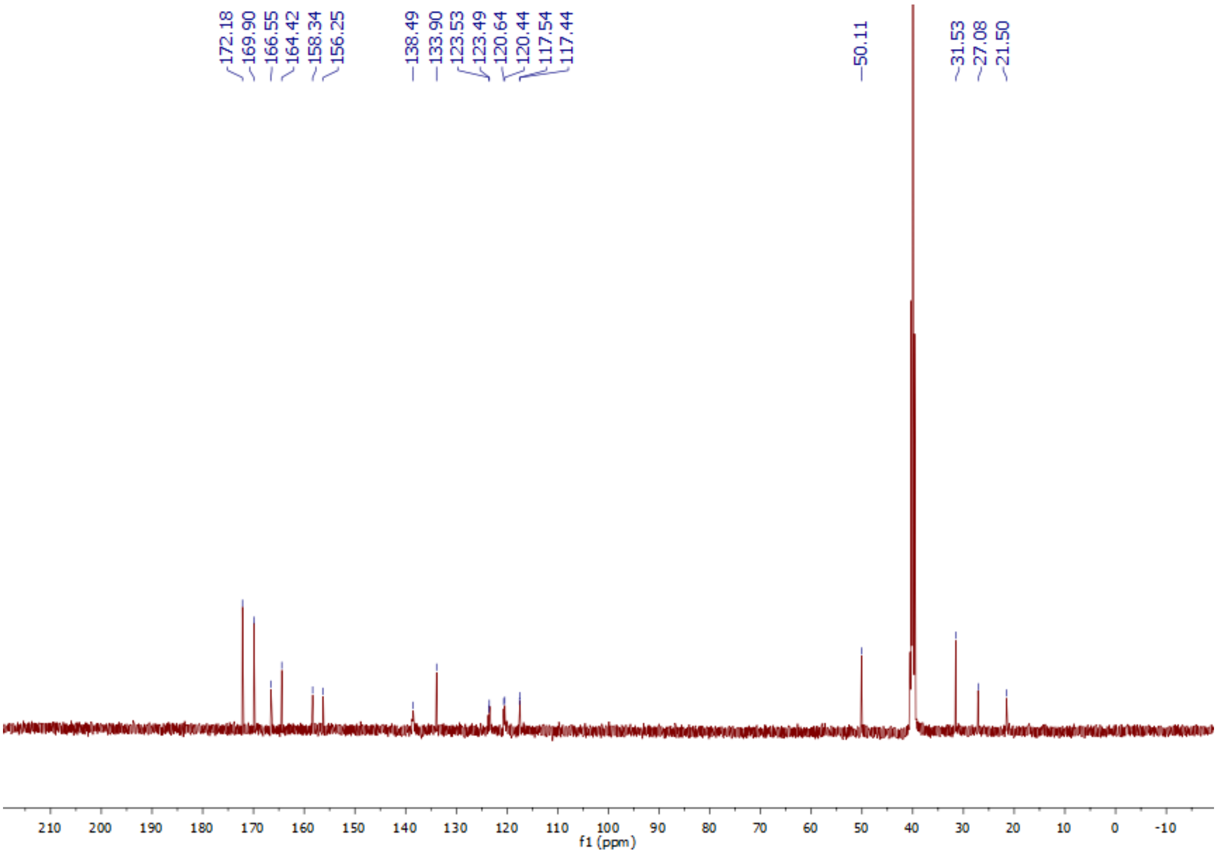
172.18 169.90 166.55 164.42 158.34 156.25
138.49 133.90 123.53 123.49 120.64 120.44 117.54 117.44
50.11
31.53 27.08 21.50
f1 (ppm)

17

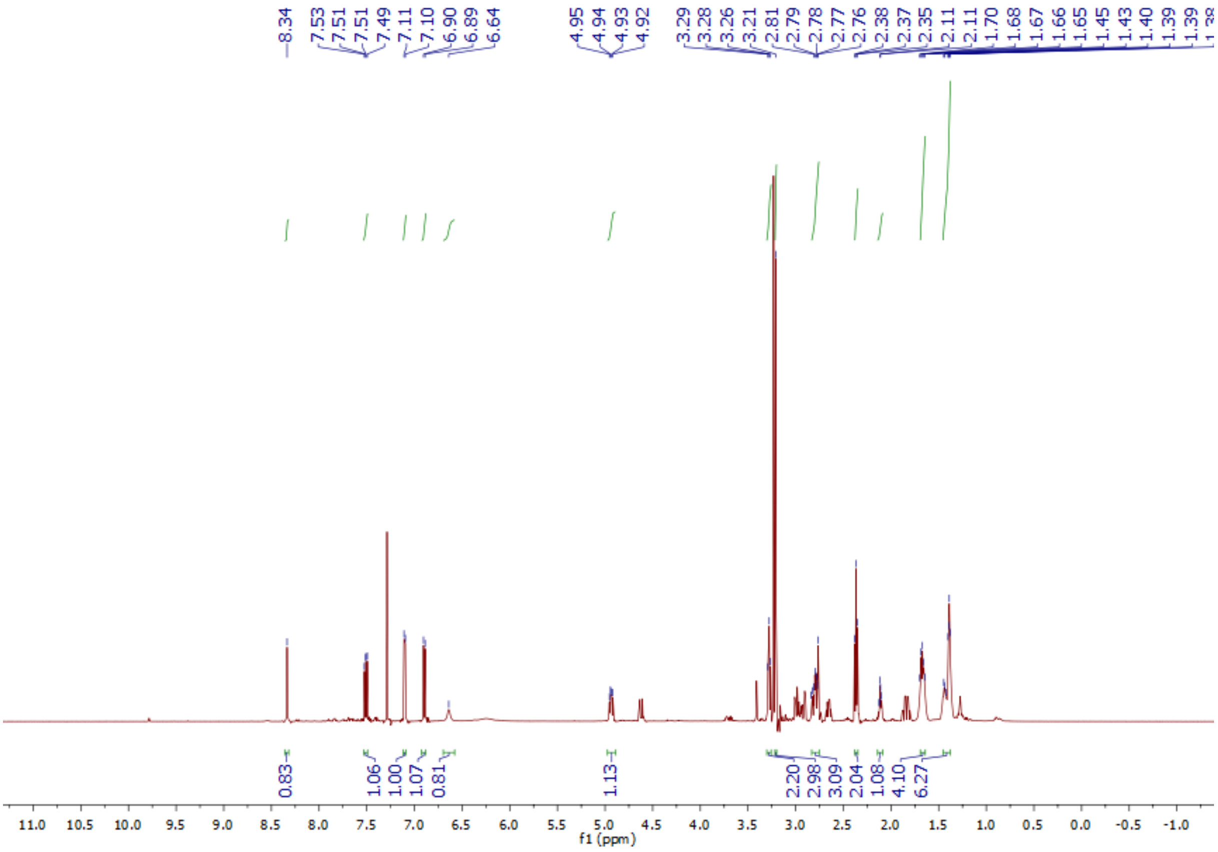

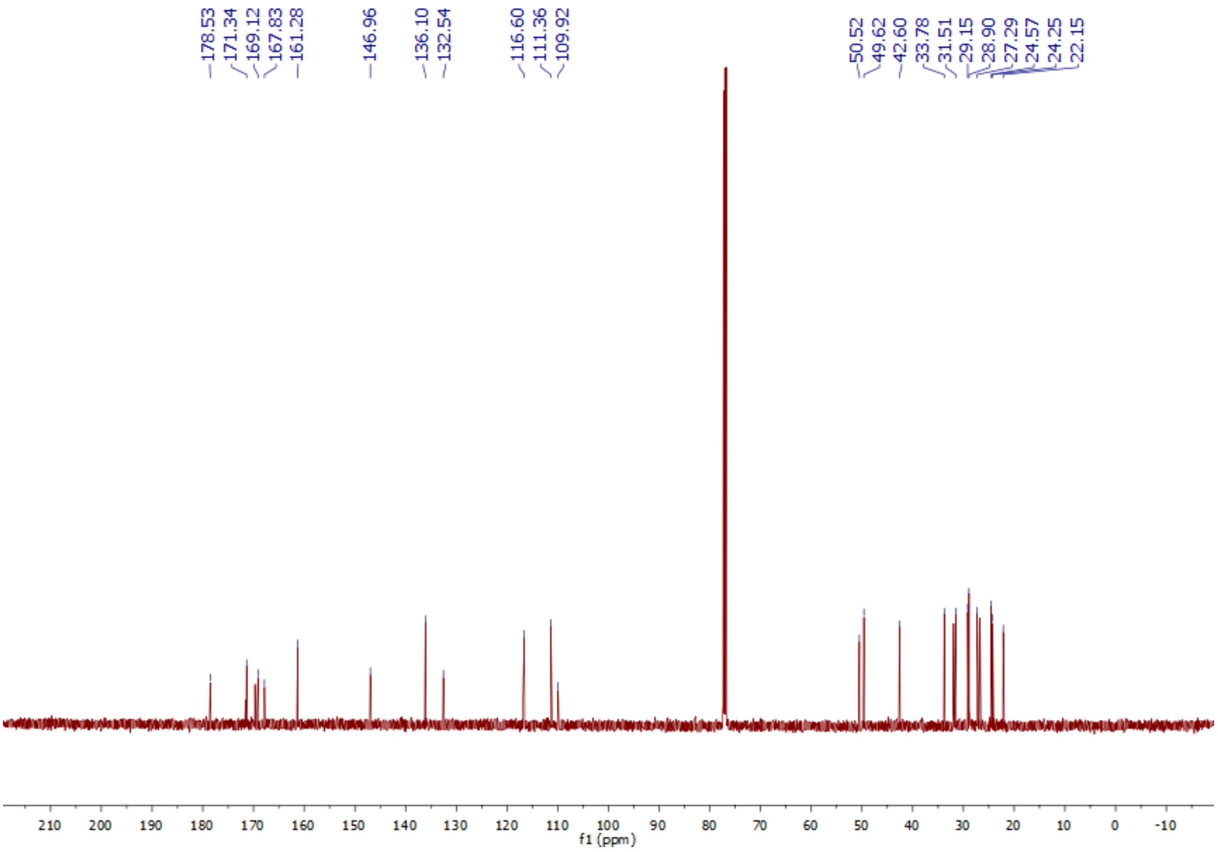

18a

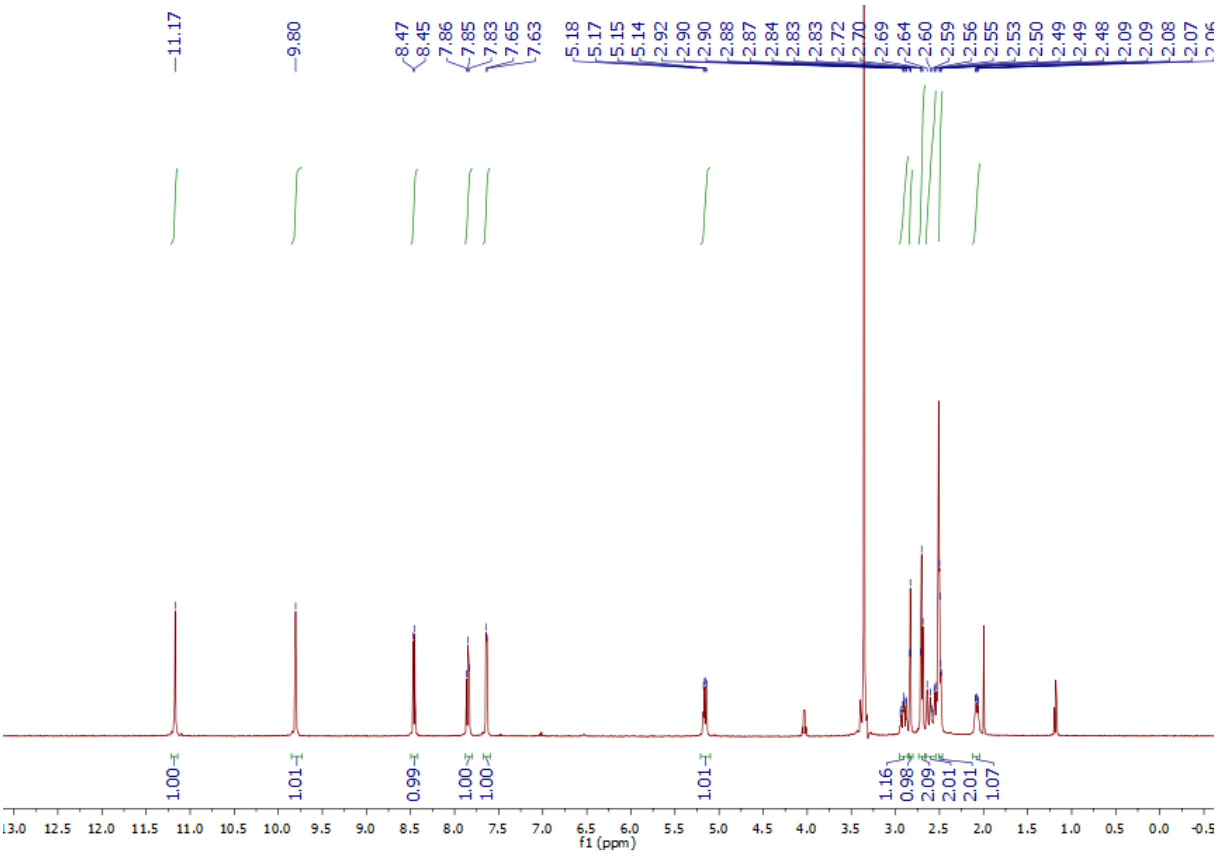

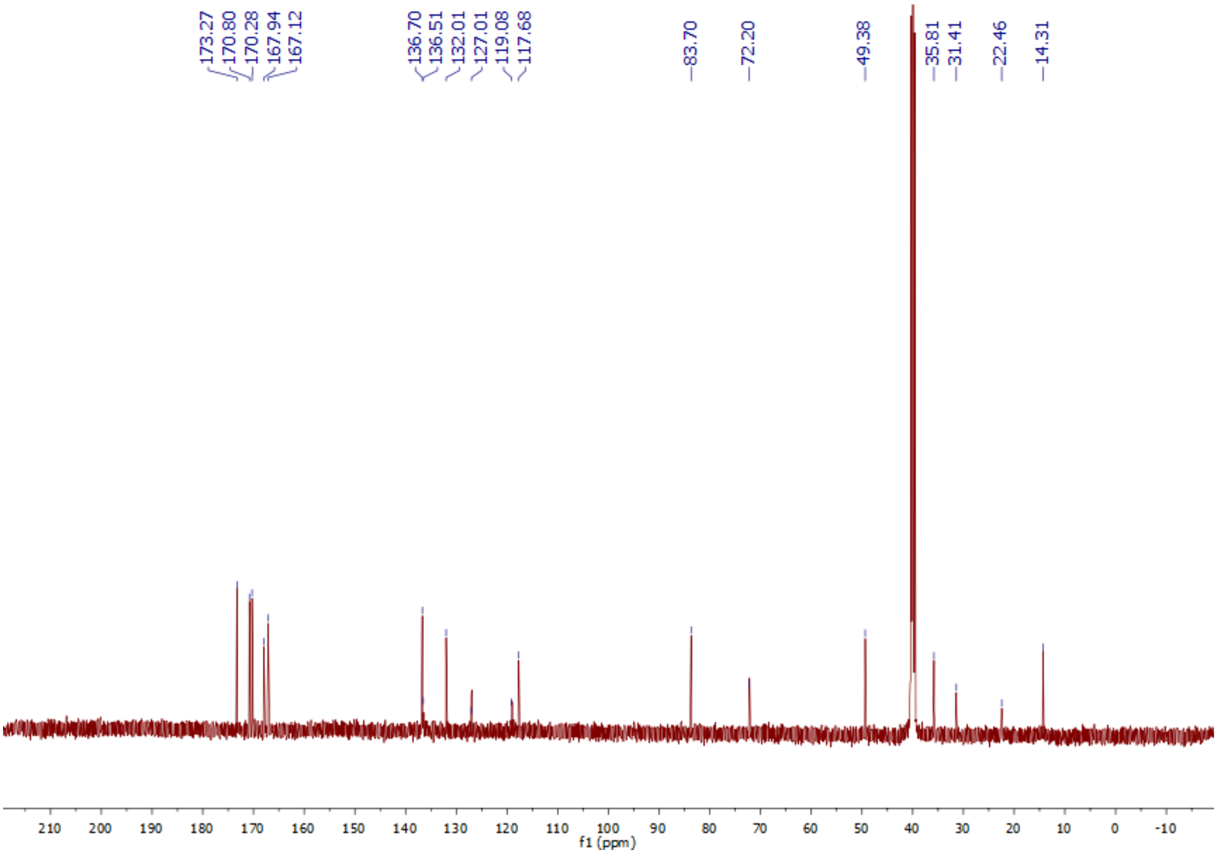

18b

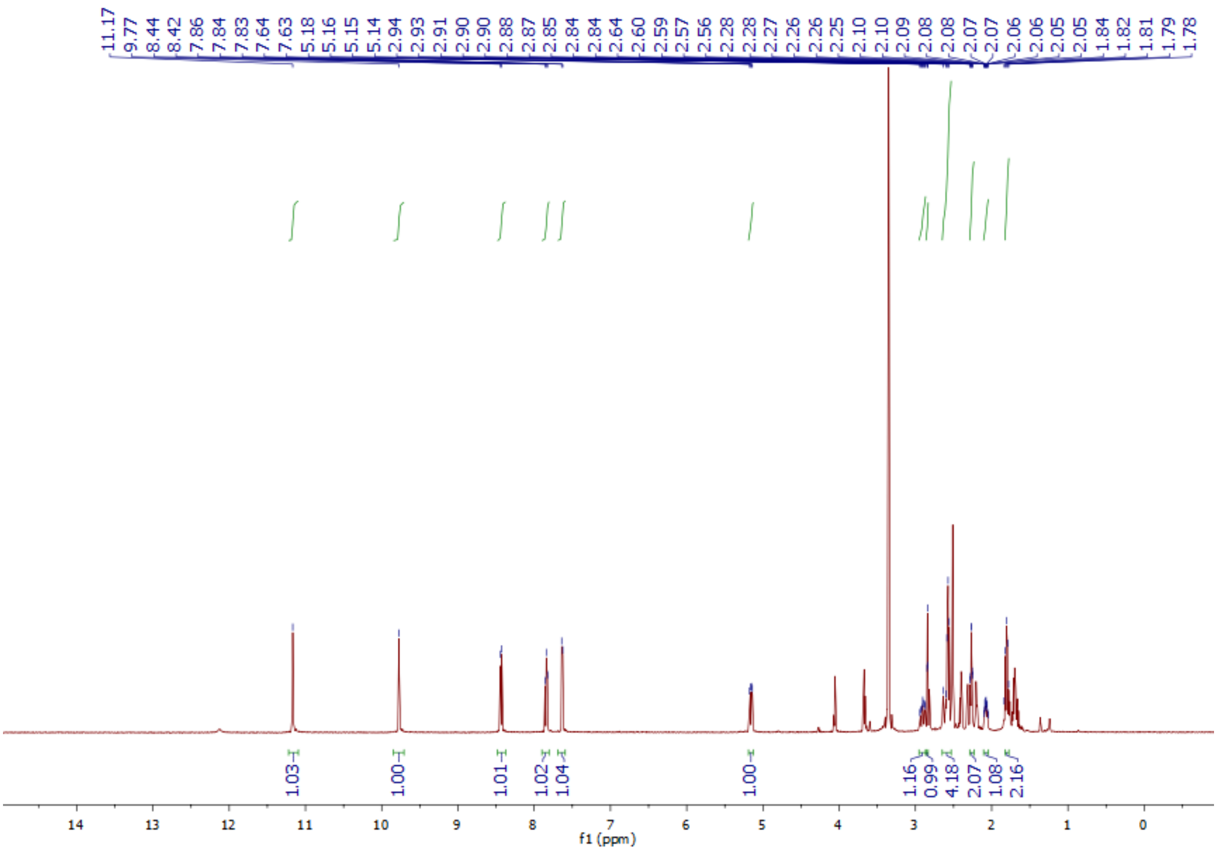

18c

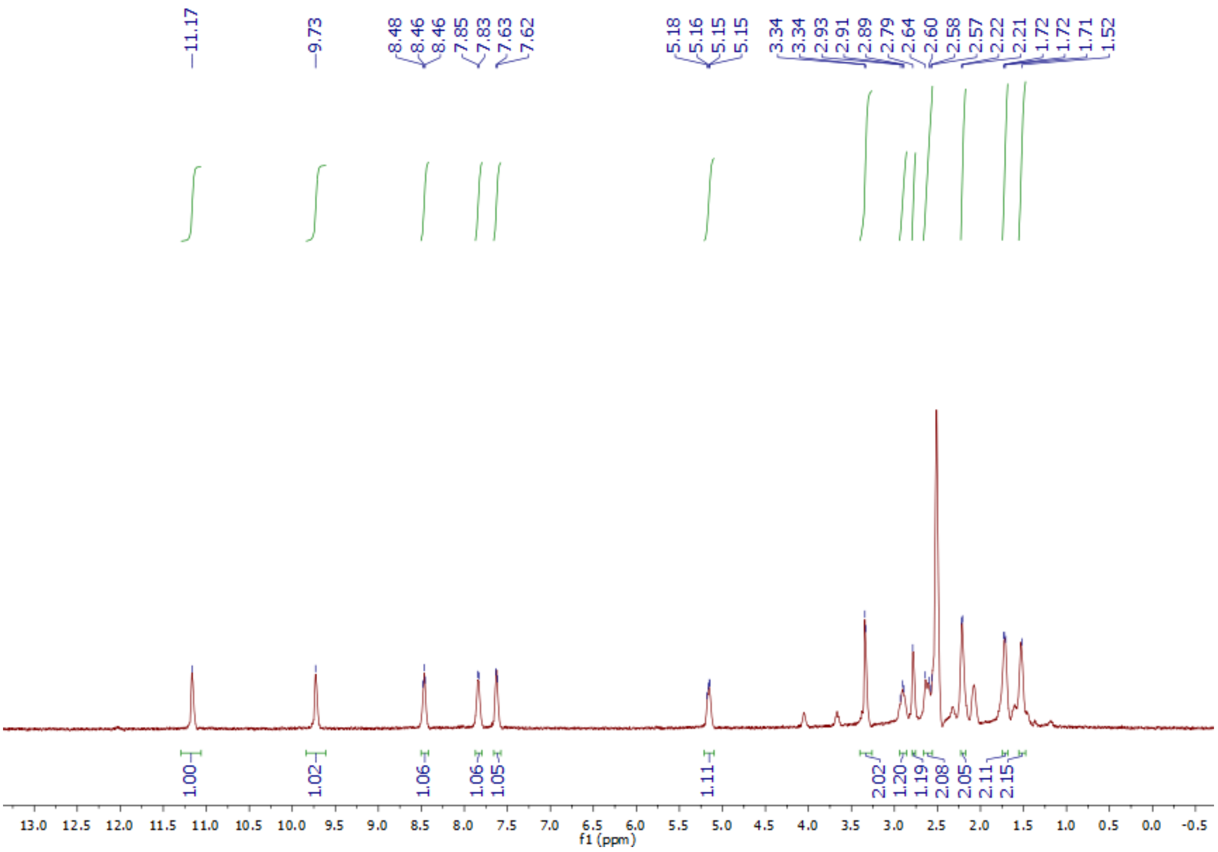

18d

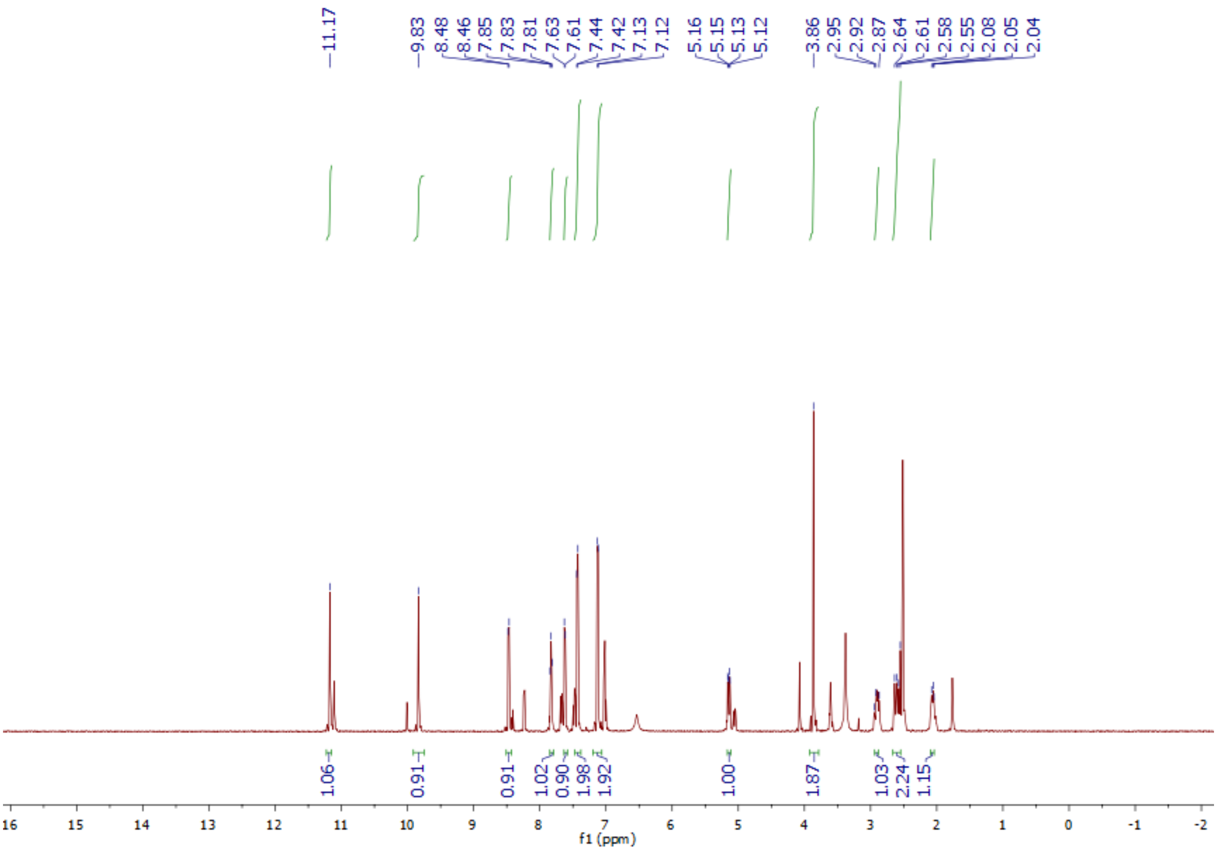

11.17
9.83
8.48
8.46
7.85
7.83
7.81
7.63
7.61
7.44
7.42
7.13
7.12
5.16
5.15
5.13
5.12
3.86
2.95
2.92
2.87
2.64
2.61
2.58
2.55
2.08
2.05
2.04
1.06
0.91
0.91
1.02
0.90
1.98
1.92
1.00
1.87
1.03
2.24
1.15
f1 (ppm)

MD1

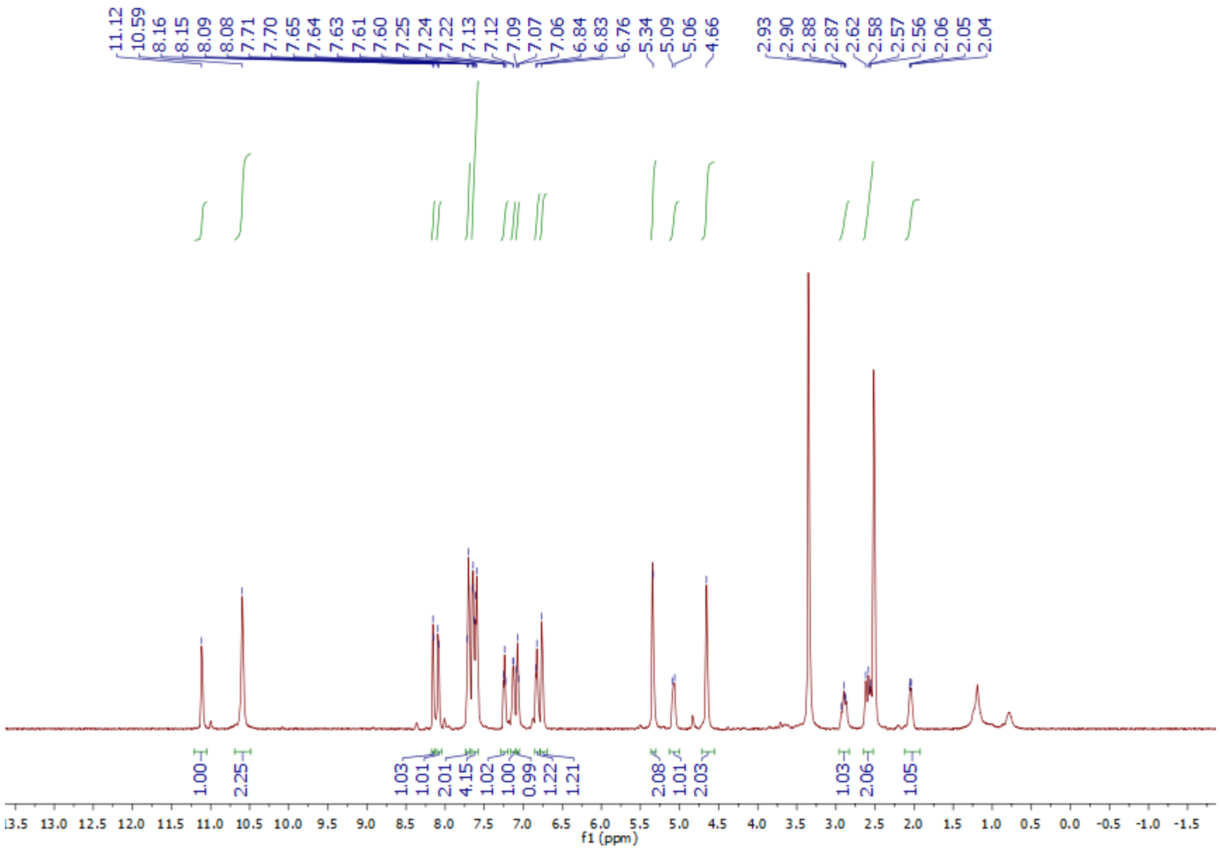

11.12
10.59
8.16
8.15
8.09
8.08
7.71
7.70
7.65
7.64
7.63
7.61
7.60
7.25
7.24
7.22
7.13
7.12
7.09
7.07
7.06
6.84
6.83
6.76
5.34
5.09
5.06
4.66
2.93
2.90
2.88
2.87
2.62
2.58
2.57
2.56
2.06
2.05
2.04
1.00
2.25
1.03
1.01
2.01
4.15
1.02
1.00
0.99
1.22
1.21
2.08
1.01
2.03
1.03
2.06
1.05
f1 (ppm)

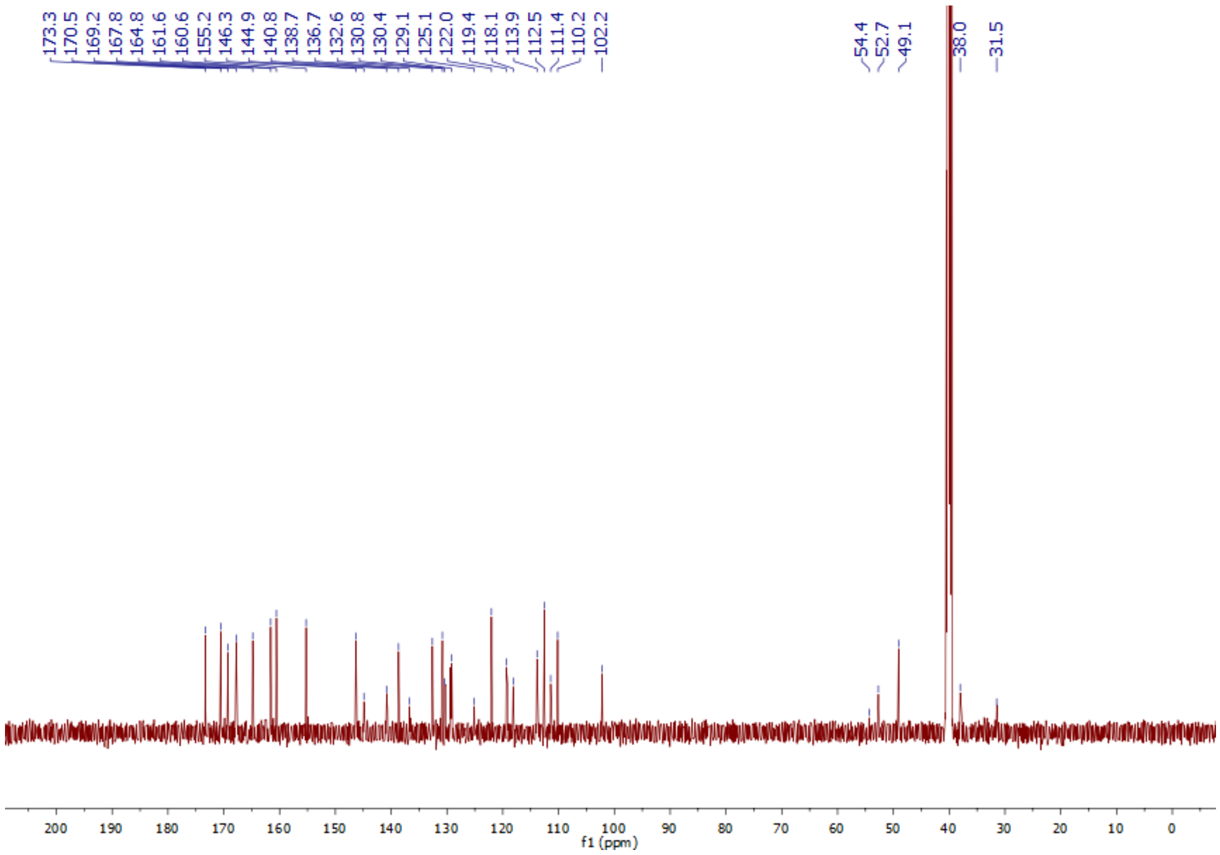

173.3
170.5
169.2
167.8
164.8
161.6
160.6
155.2
146.3
144.9
140.8
138.7
136.7
132.6
130.8
130.4
129.1
125.1
122.0
119.4
118.1
113.9
112.5
111.4
110.2
102.2
54.4
52.7
49.1
38.0
31.5
f1 (ppm)

MD2

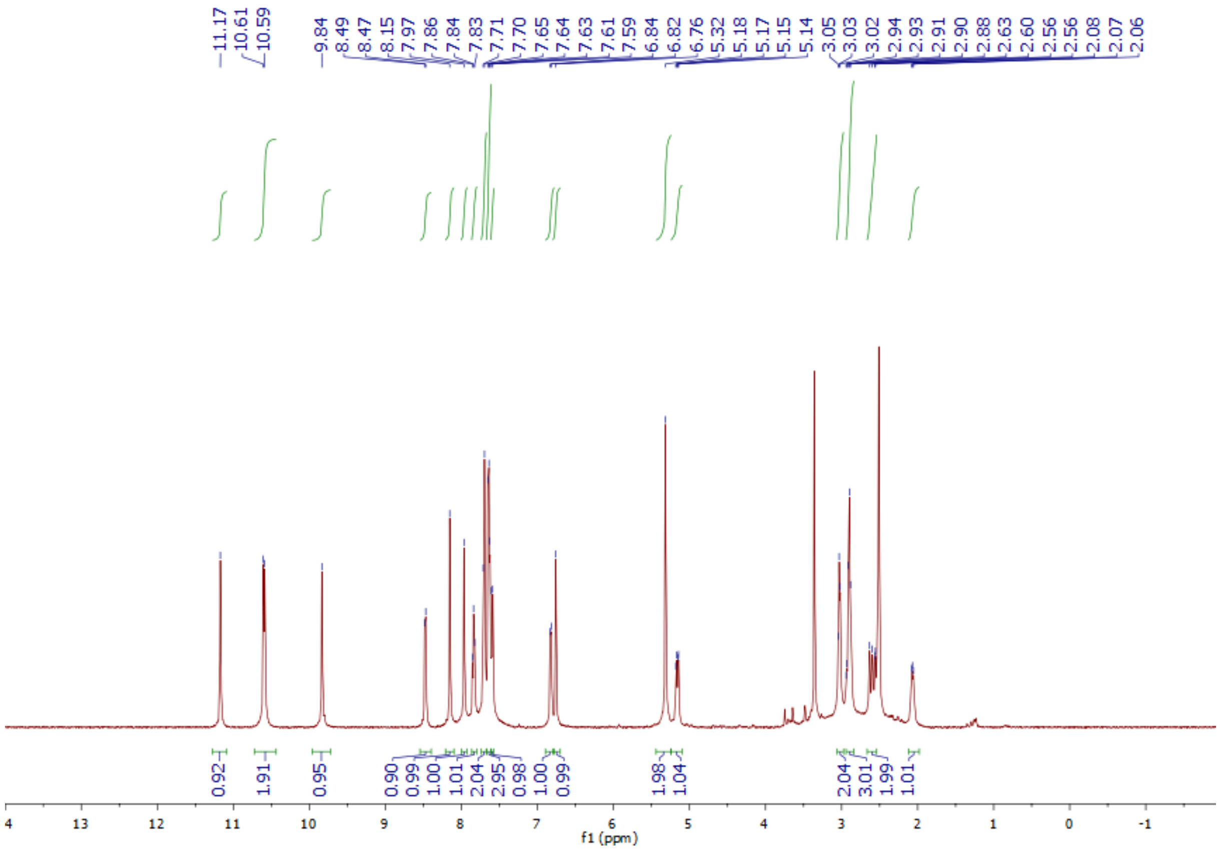

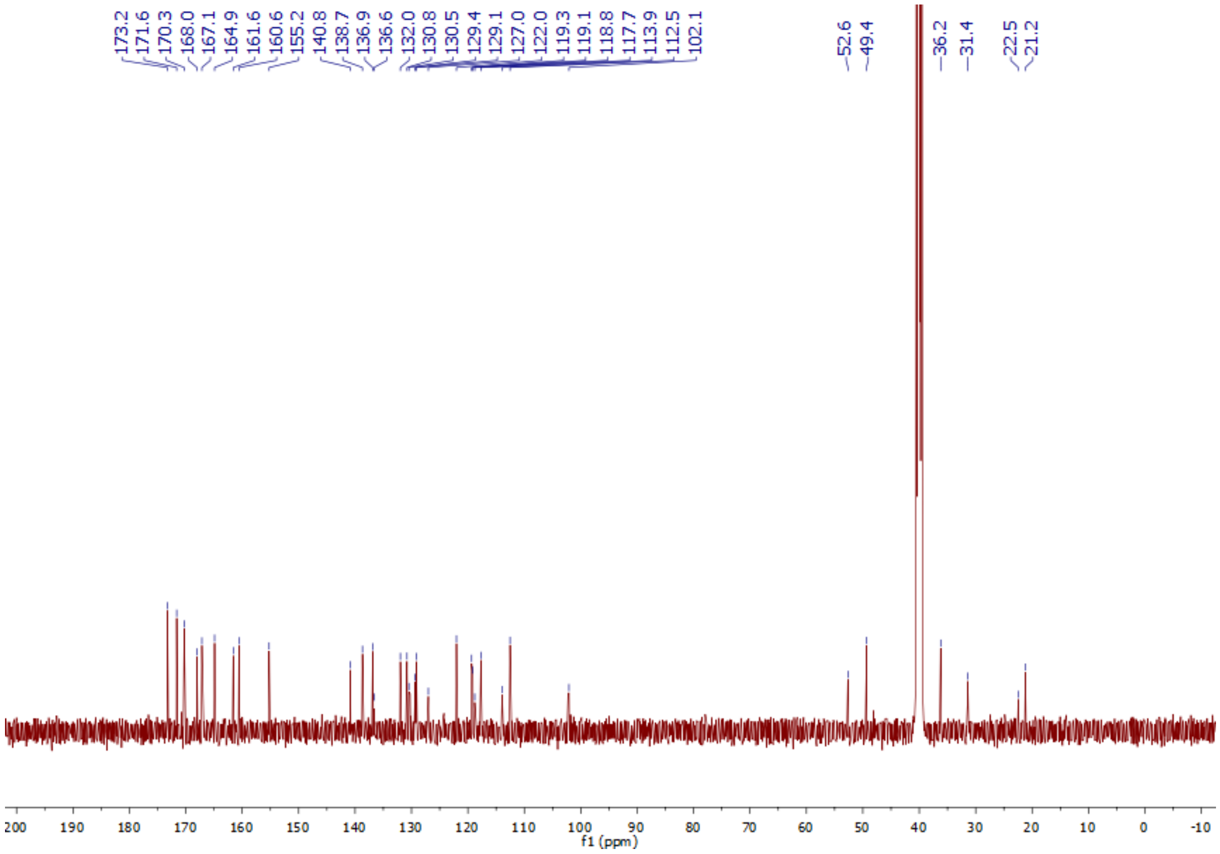

MD3

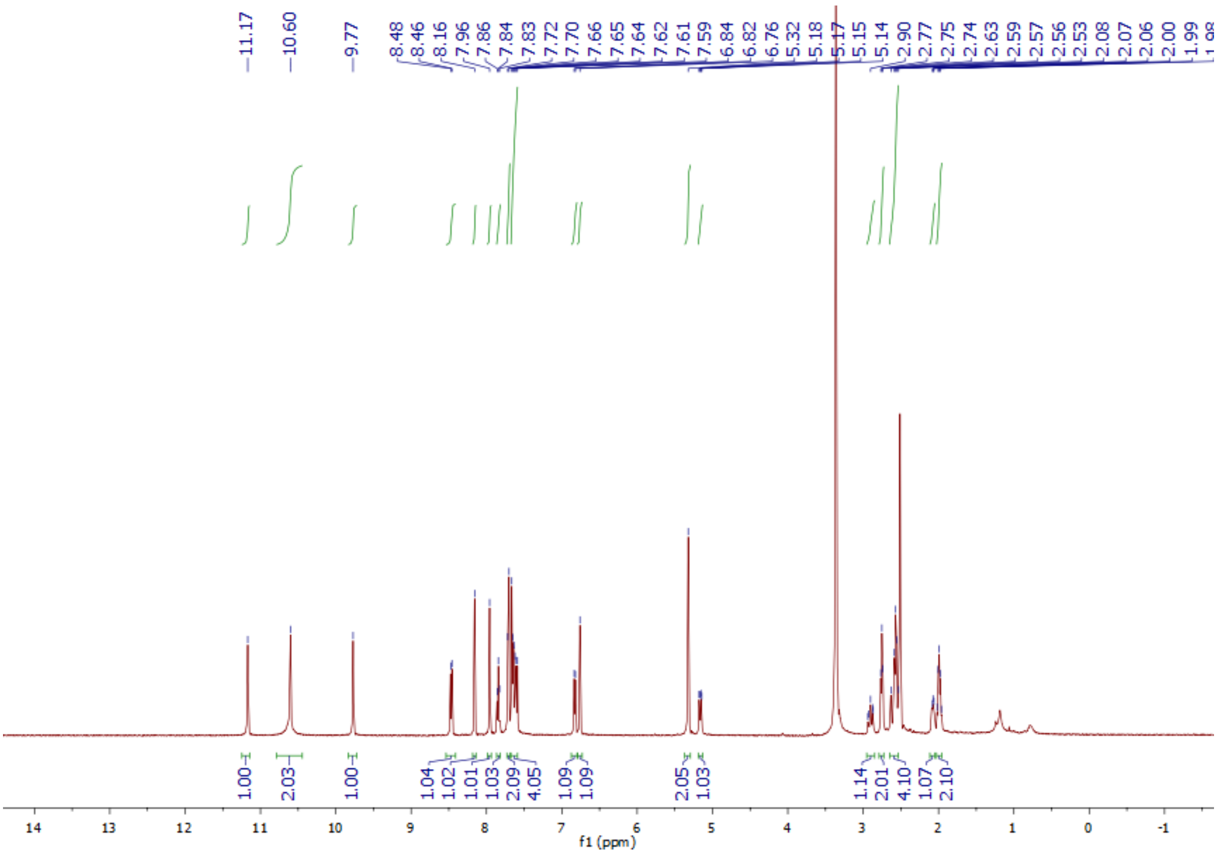

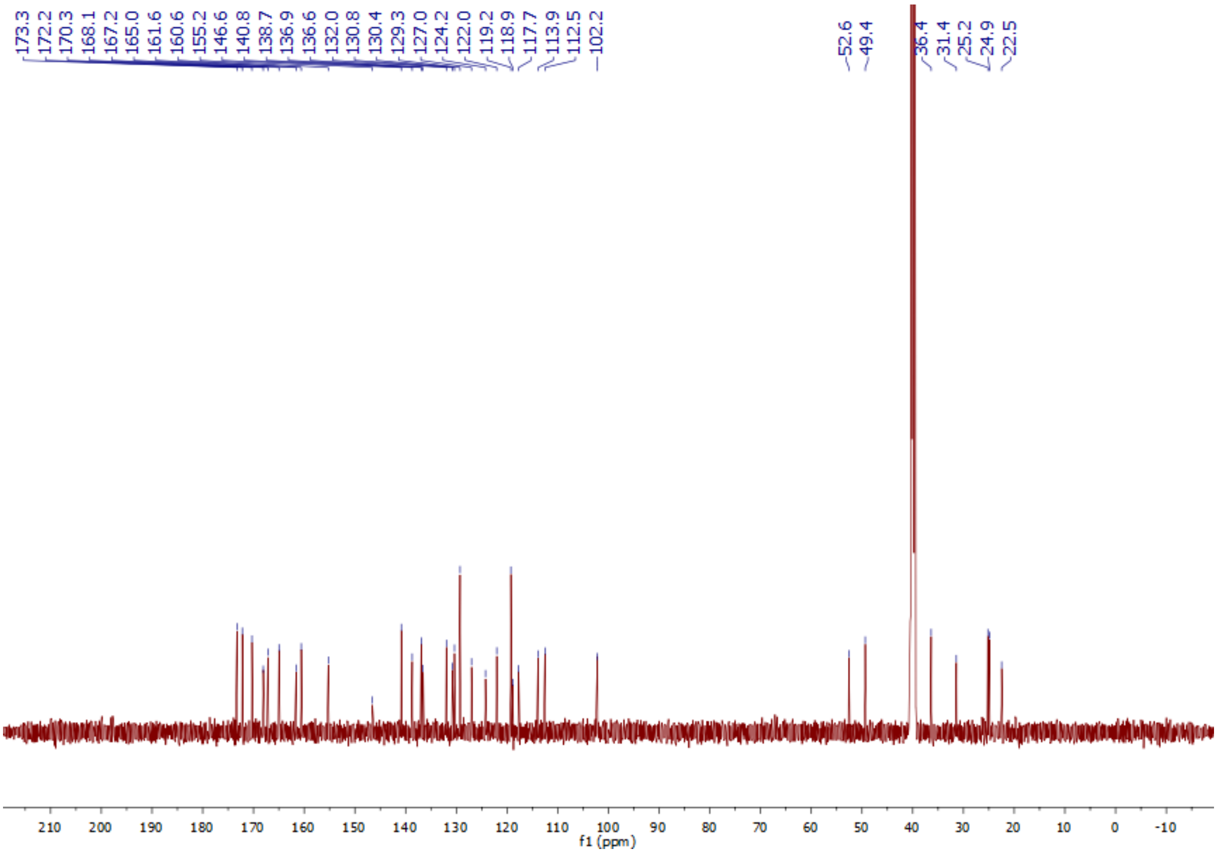

MD4

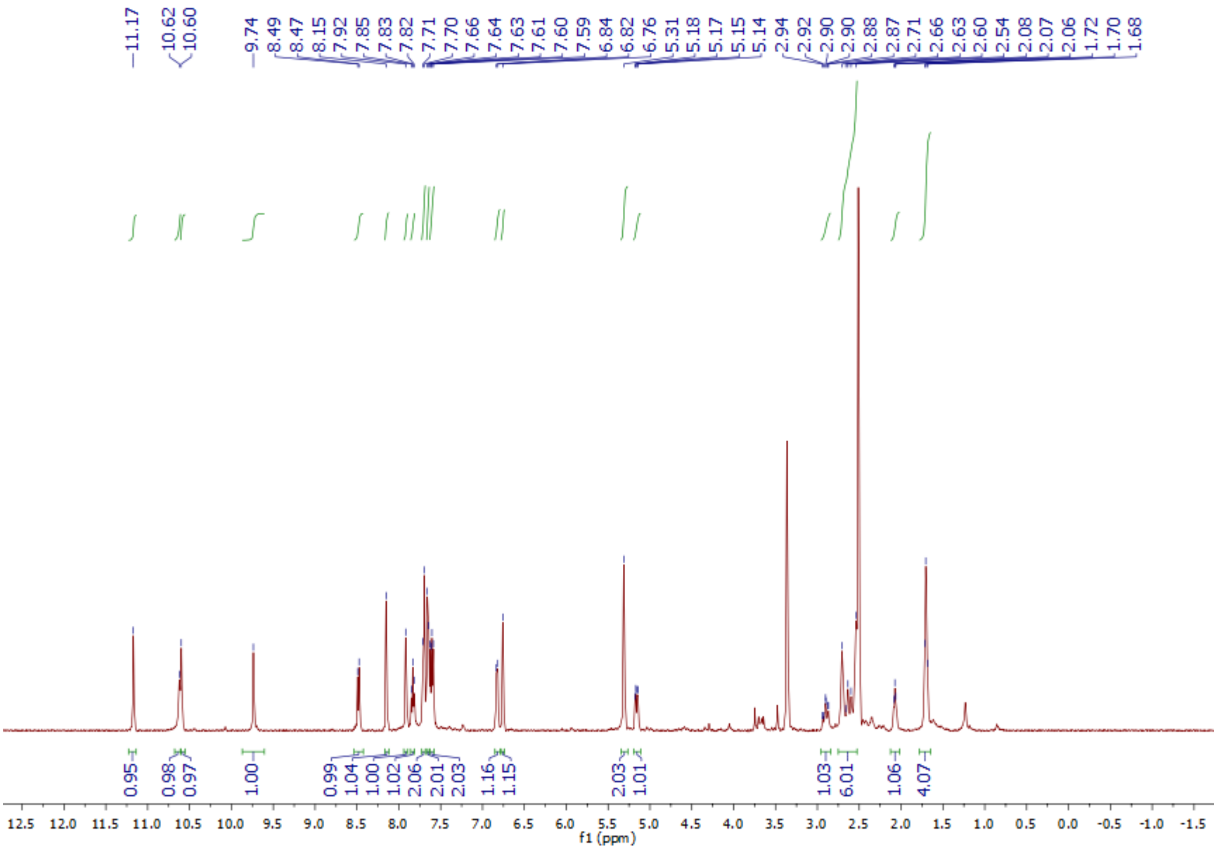

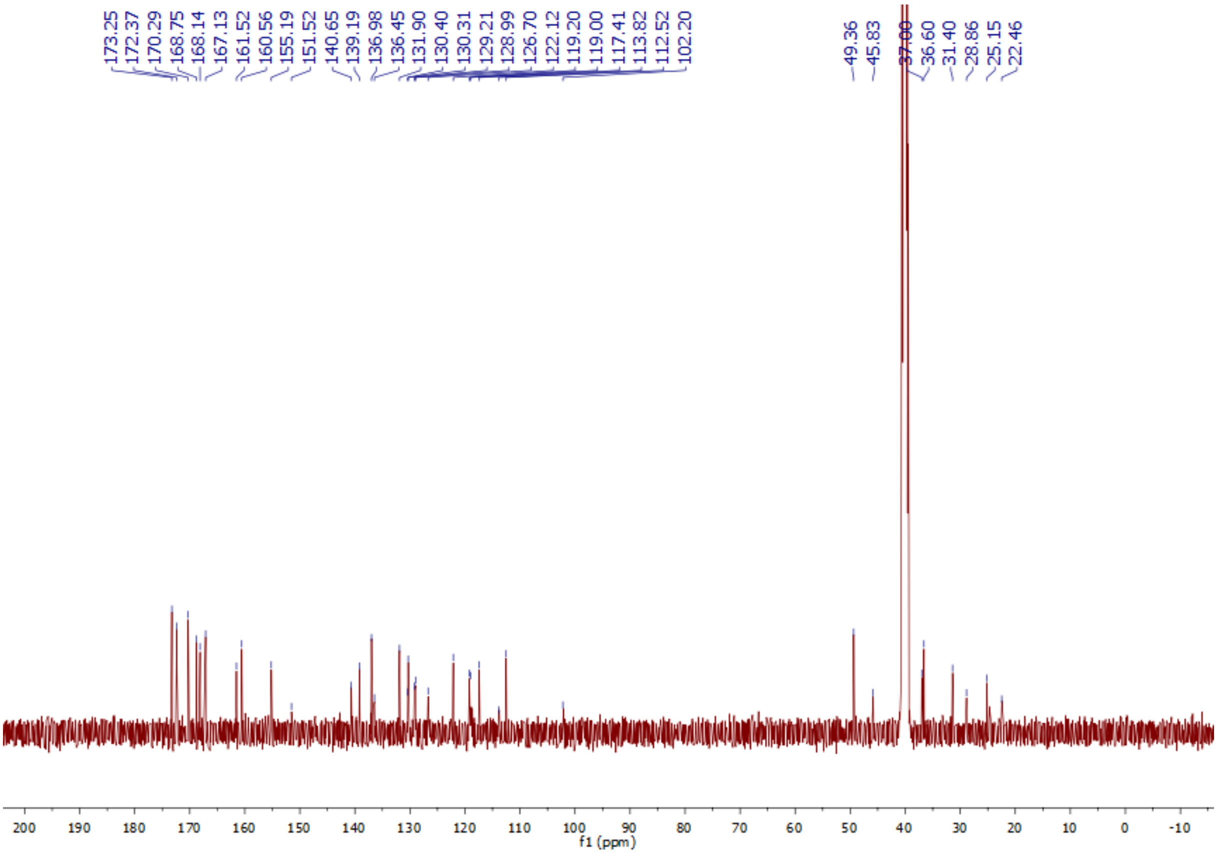

MD5

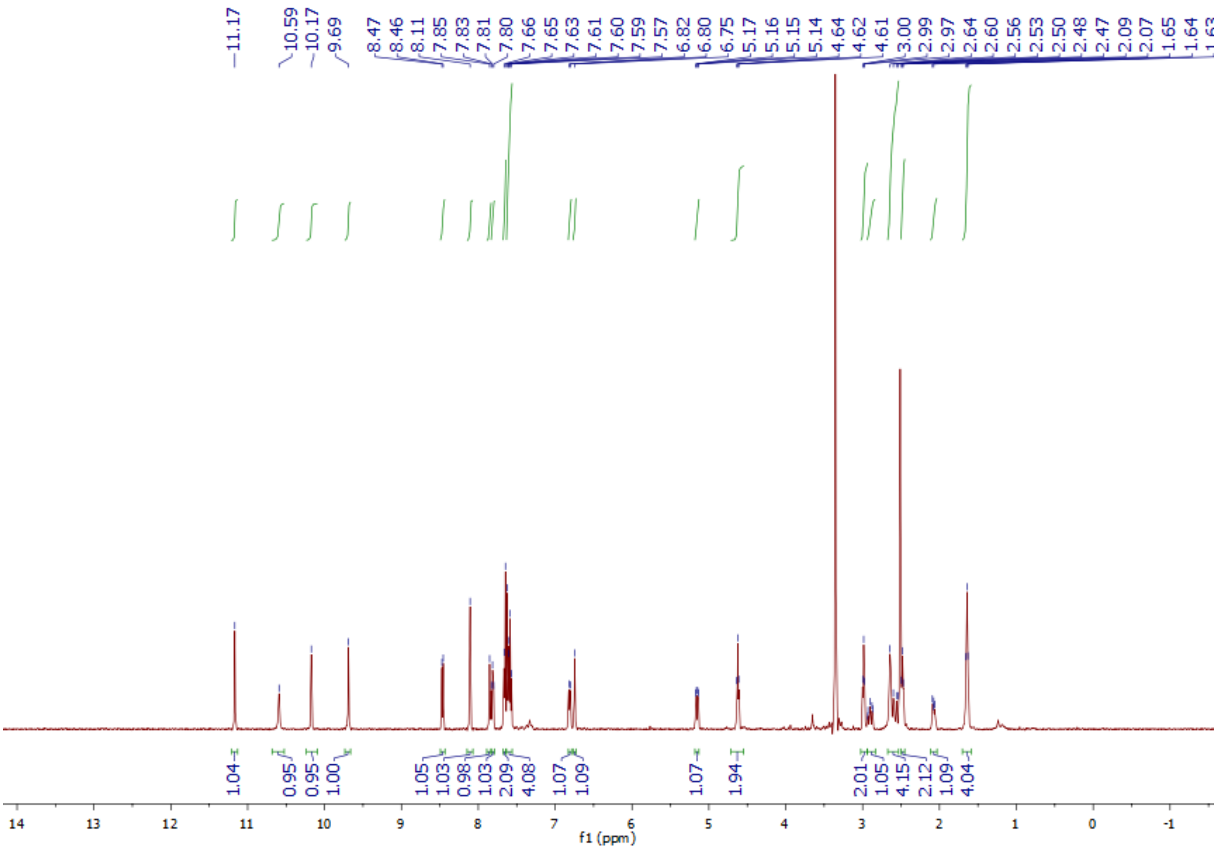

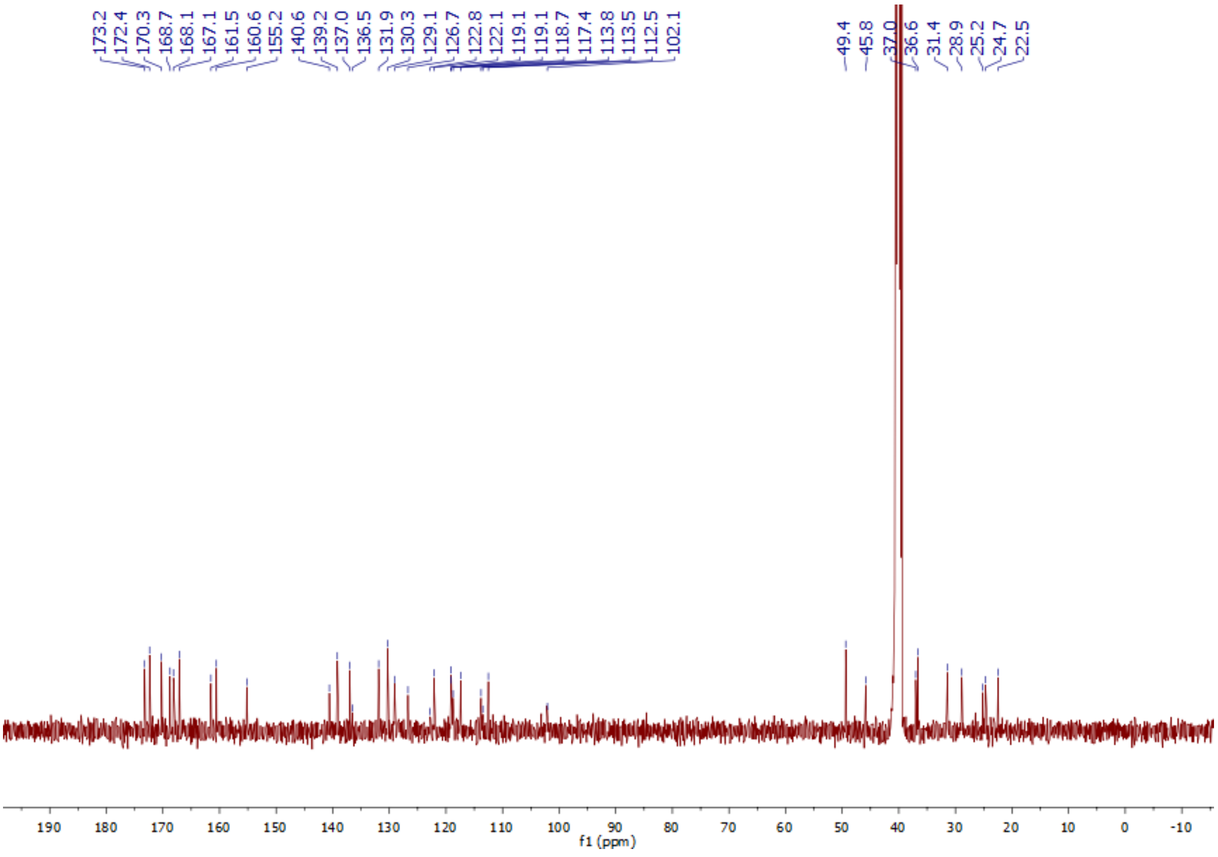

MD6

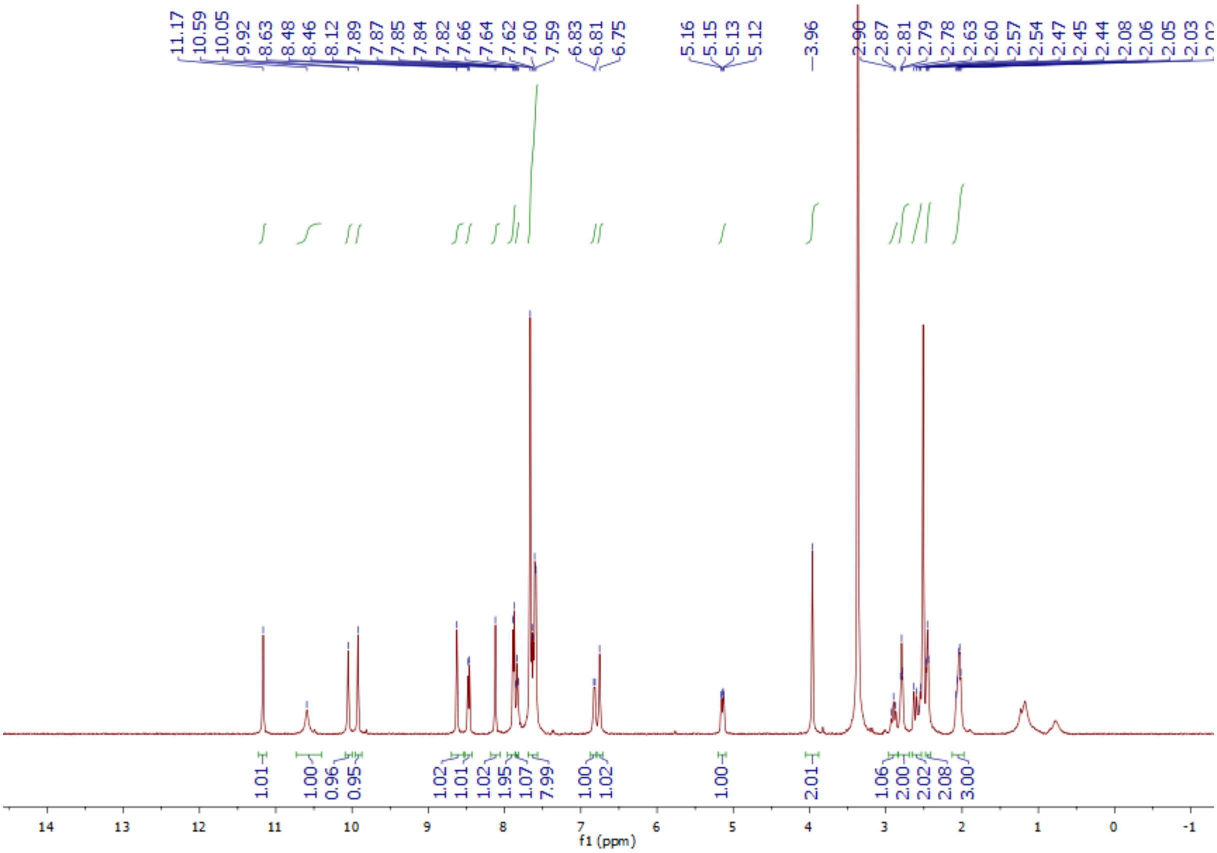
11.17
10.59
10.05
9.92
8.63
8.48
8.46
8.12
7.89
7.87
7.85
7.84
7.82
7.66
7.64
7.62
7.60
7.59
6.83
6.81
6.75
5.16
5.15
5.13
5.12
3.96
2.90
2.87
2.81
2.79
2.78
2.63
2.60
2.57
2.54
2.47
2.45
2.44
2.08
2.06
2.05
2.03
1.01
1.00
0.96
0.95
1.02
1.01
1.02
1.95
1.07
7.99
1.00
1.02
1.00
2.01
1.06
2.00
2.02
2.08
3.00
f1 (ppm)

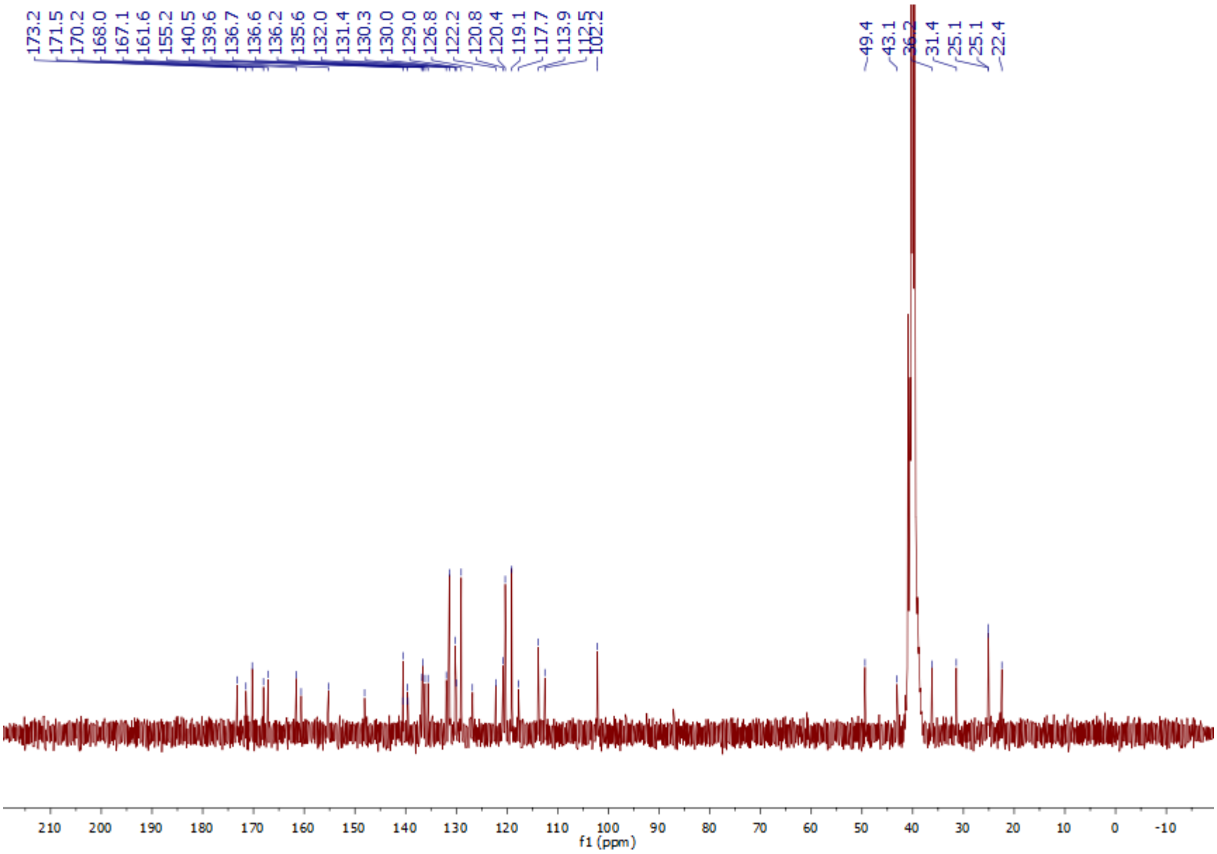
173.2
171.5
170.2
168.0
167.1
161.6
155.2
140.5
139.6
136.7
136.6
136.2
135.6
132.0
131.4
130.3
130.0
129.0
126.8
122.2
120.8
120.4
119.1
117.7
113.9
112.5
102.2
49.4
43.1
36.2
31.4
25.1
25.1
22.4
f1 (ppm)

MD7

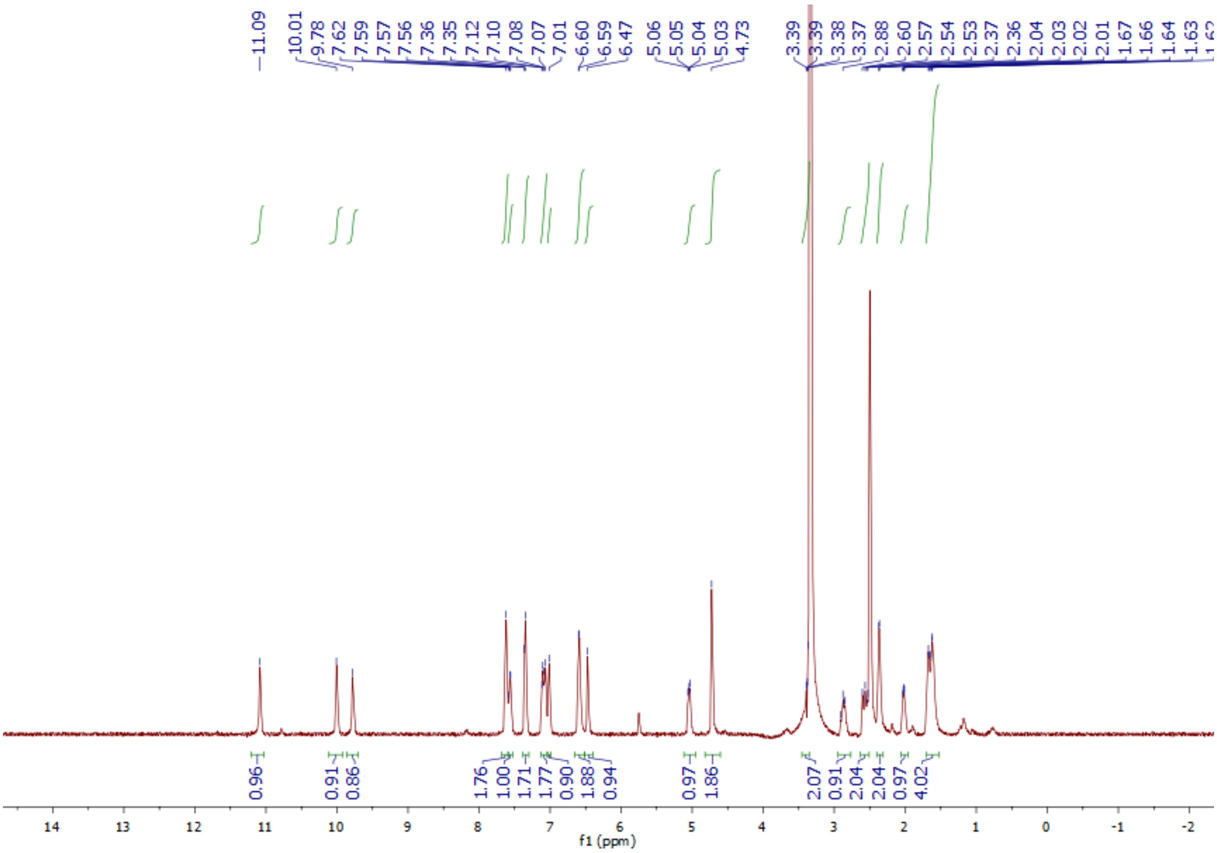
11.09
10.01
9.78
7.62
7.59
7.57
7.56
7.36
7.35
7.12
7.10
7.08
7.07
7.01
6.60
6.59
6.47
5.06
5.05
5.04
5.03
4.73
3.39
3.39
3.38
3.37
2.88
2.60
2.57
2.54
2.53
2.37
2.36
2.04
2.03
2.02
2.01
1.67
1.66
1.64
1.63
0.96
0.91
0.86
1.76
1.00
1.71
1.77
0.90
1.88
0.94
0.97
1.86
2.07
0.91
2.04
2.04
0.97
4.02
f1 (ppm)

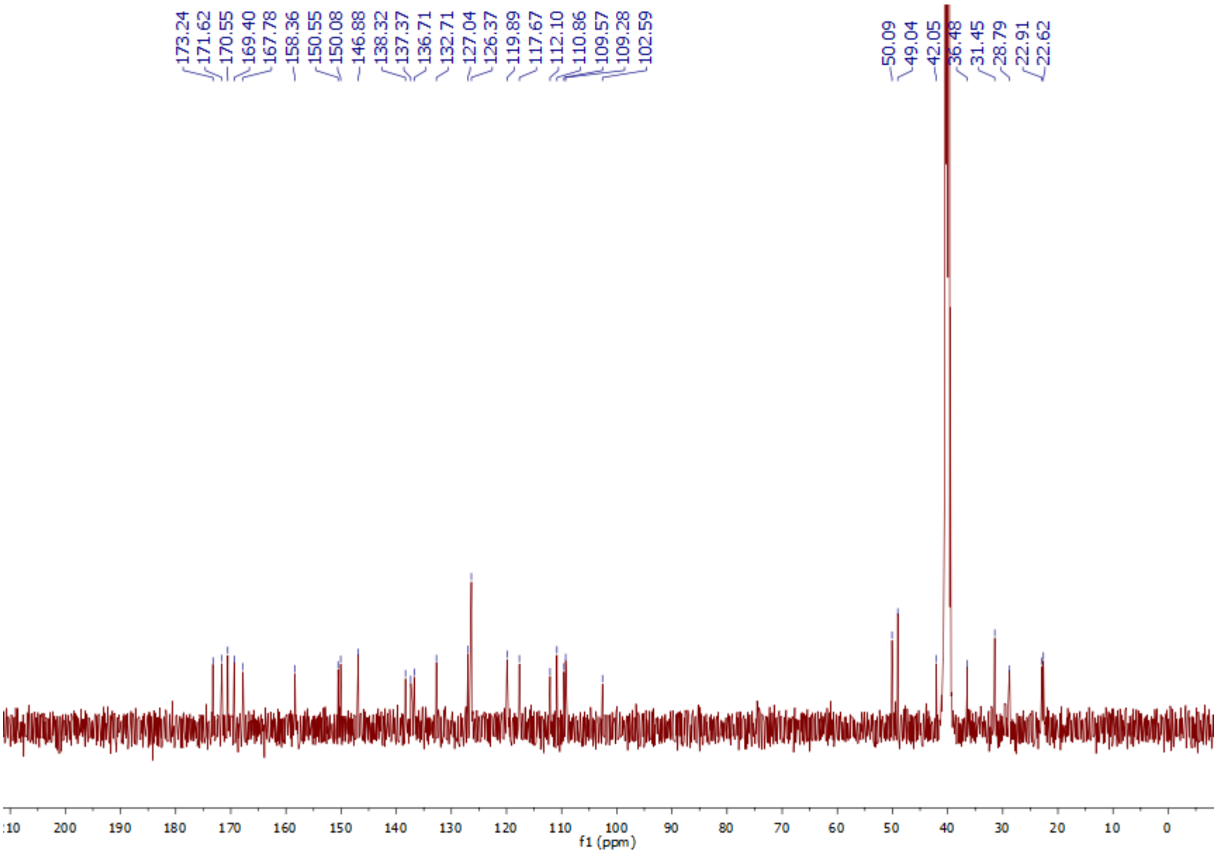
173.24
171.62
170.55
169.40
167.78
158.36
150.55
150.08
146.88
138.32
137.37
136.71
132.71
127.04
126.37
119.89
117.67
112.10
110.86
109.57
109.28
102.59
50.09
49.04
42.05
36.48
31.45
28.79
22.91
22.62
f1 (ppm)

MD8

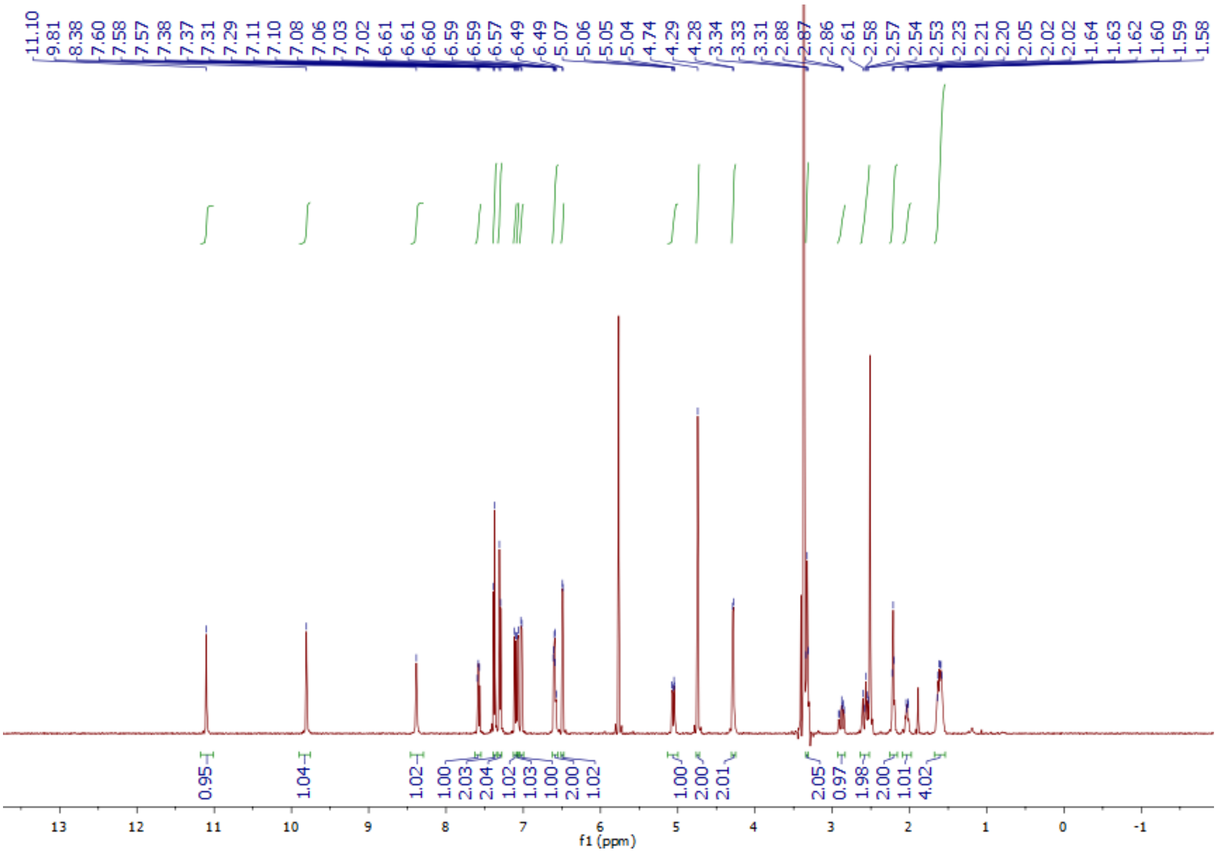

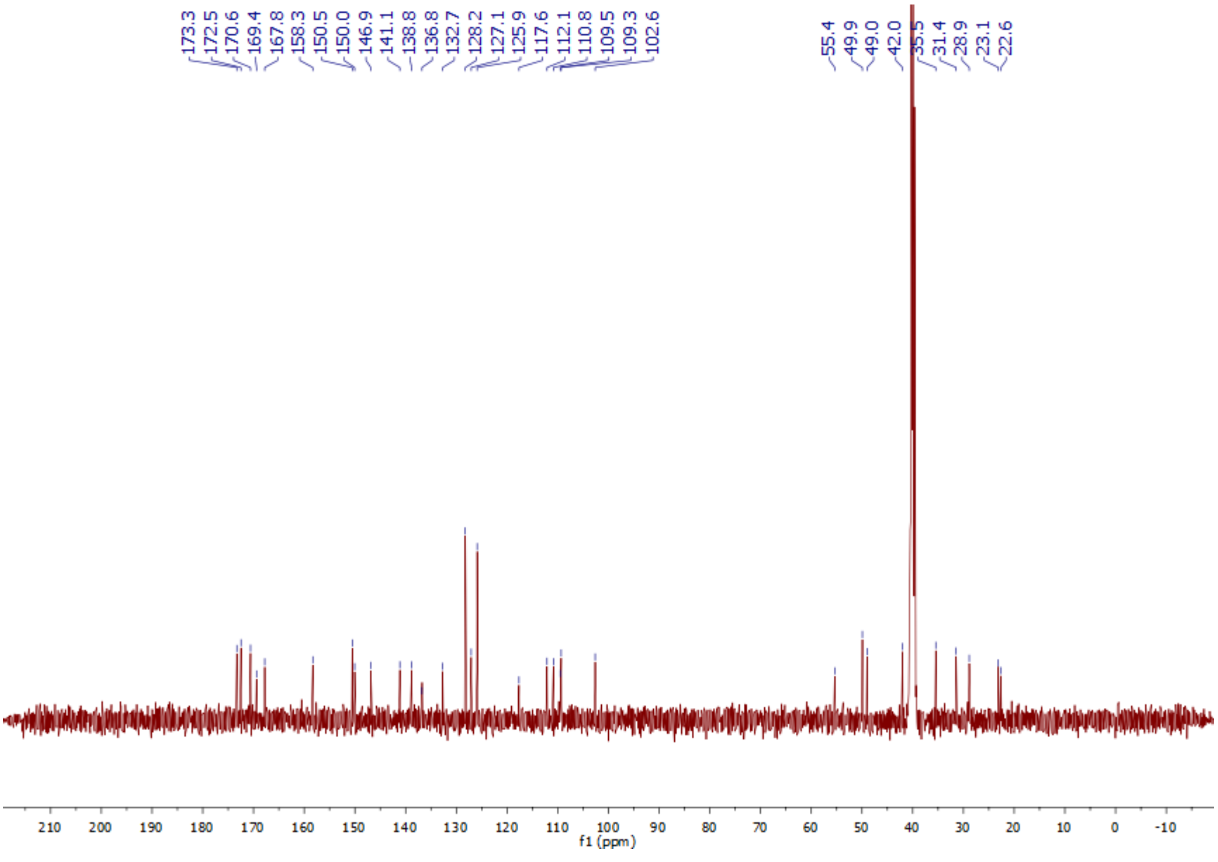

MD9

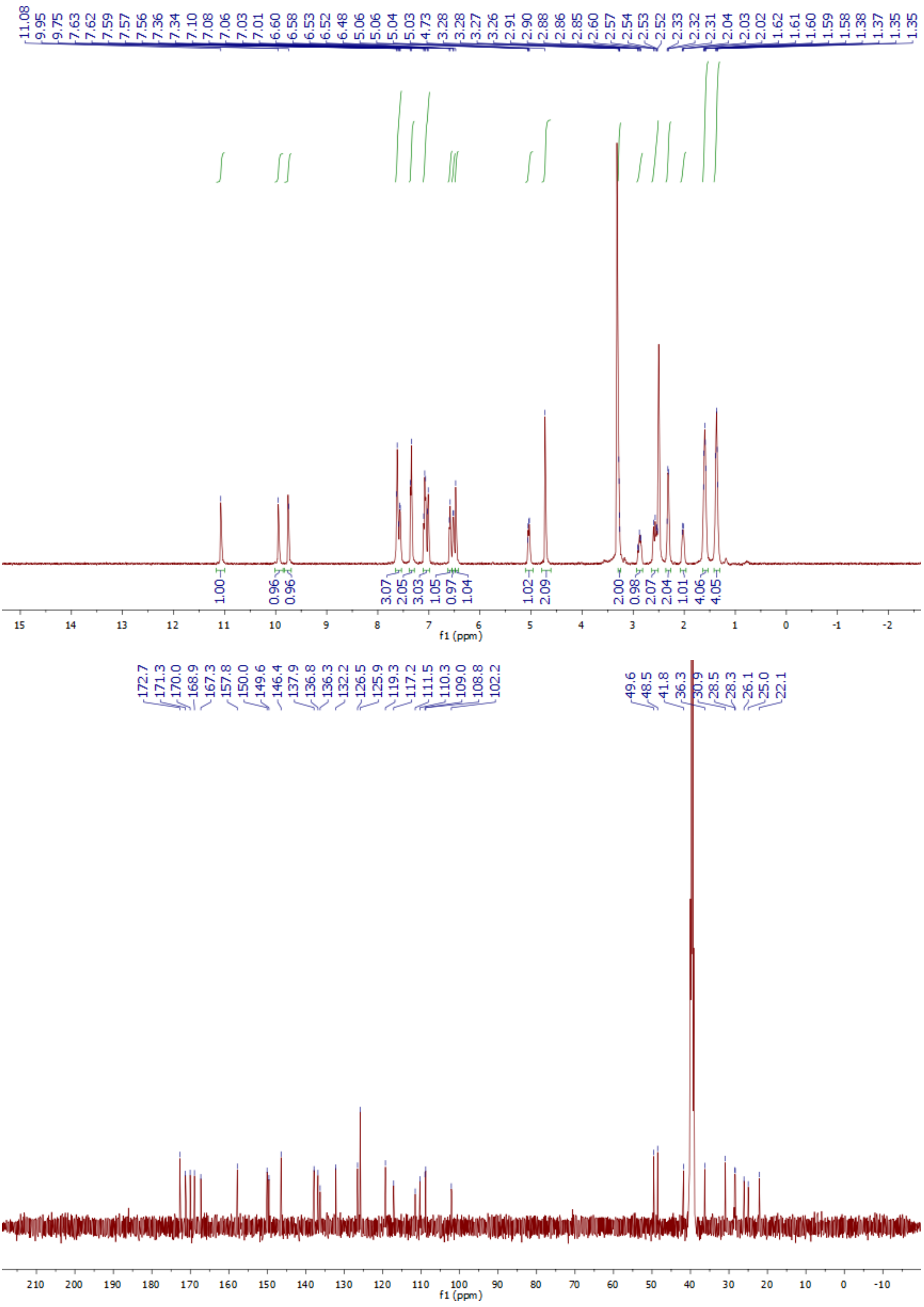
11.08
9.95
9.75
7.63
7.62
7.59
7.57
7.56
7.36
7.34
7.10
7.08
7.06
7.03
7.01
6.60
6.58
6.53
6.52
6.48
5.06
5.06
5.04
5.03
4.73
3.28
3.28
3.27
3.26
2.91
2.90
2.88
2.86
2.85
2.60
2.57
2.54
2.53
2.52
2.33
2.32
2.31
2.04
2.03
2.02
1.62
1.61
1.60
1.59
1.58
1.38
1.37
1.35
1.35
1.00
0.96
0.96
3.07
2.05
3.03
1.05
0.97
1.04
1.02
2.09
2.00
0.98
2.07
2.04
1.01
4.06
4.05
f1 (ppm)
172.7
171.3
170.0
168.9
167.3
157.8
150.0
149.6
146.4
137.9
136.8
136.3
132.2
126.5
125.9
119.3
117.2
111.5
110.3
109.0
108.8
102.2
49.6
48.5
41.8
36.3
30.9
28.5
28.3
26.1
25.0
22.1
f1 (ppm)

MD10

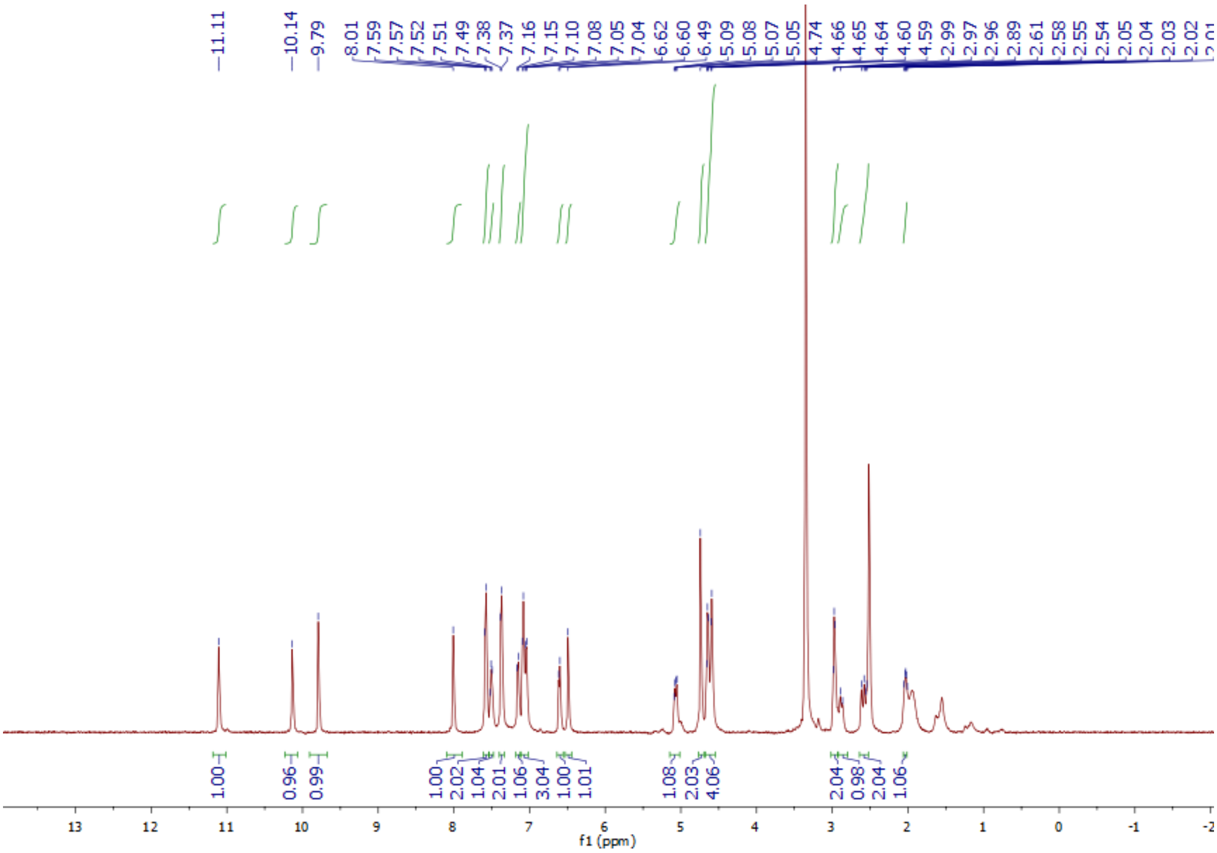
11.11
10.14
9.79
8.01
7.59
7.57
7.52
7.51
7.49
7.38
7.37
7.16
7.15
7.10
7.08
7.05
7.04
6.62
6.60
6.49
5.09
5.08
5.07
5.05
4.74
4.66
4.65
4.64
4.60
4.59
2.99
2.97
2.96
2.89
2.61
2.58
2.55
2.54
2.05
2.04
2.03
2.02
1.00
0.96
0.99
1.00
2.02
1.04
2.01
1.06
3.04
1.00
1.01
1.08
2.03
4.06
2.04
0.98
2.04
1.06
f1 (ppm)

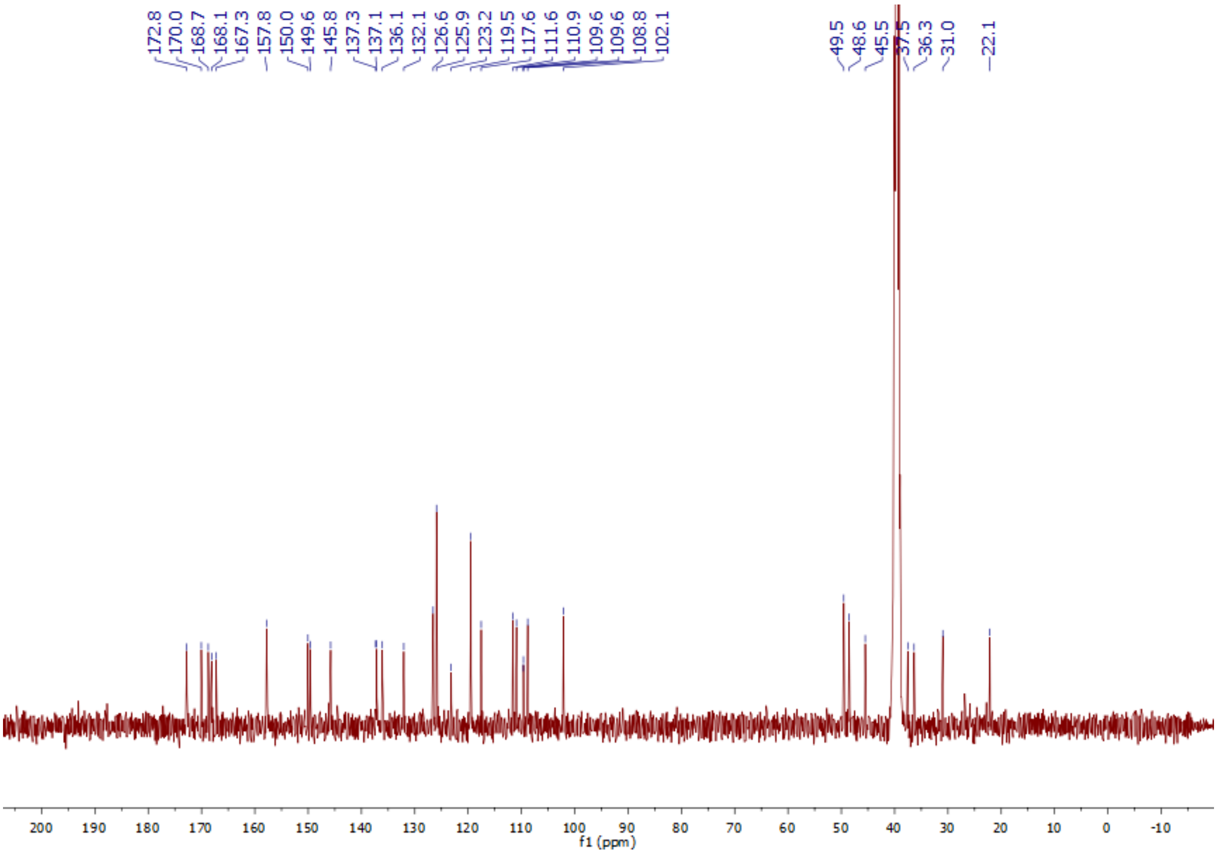
172.8
170.0
168.7
168.1
167.3
157.8
150.0
149.6
145.8
137.3
137.1
136.1
132.1
126.6
125.9
123.2
119.5
117.6
111.6
110.9
109.6
109.6
108.8
102.1
49.5
48.6
45.5
37.5
36.3
31.0
22.1
f1 (ppm)

MD11

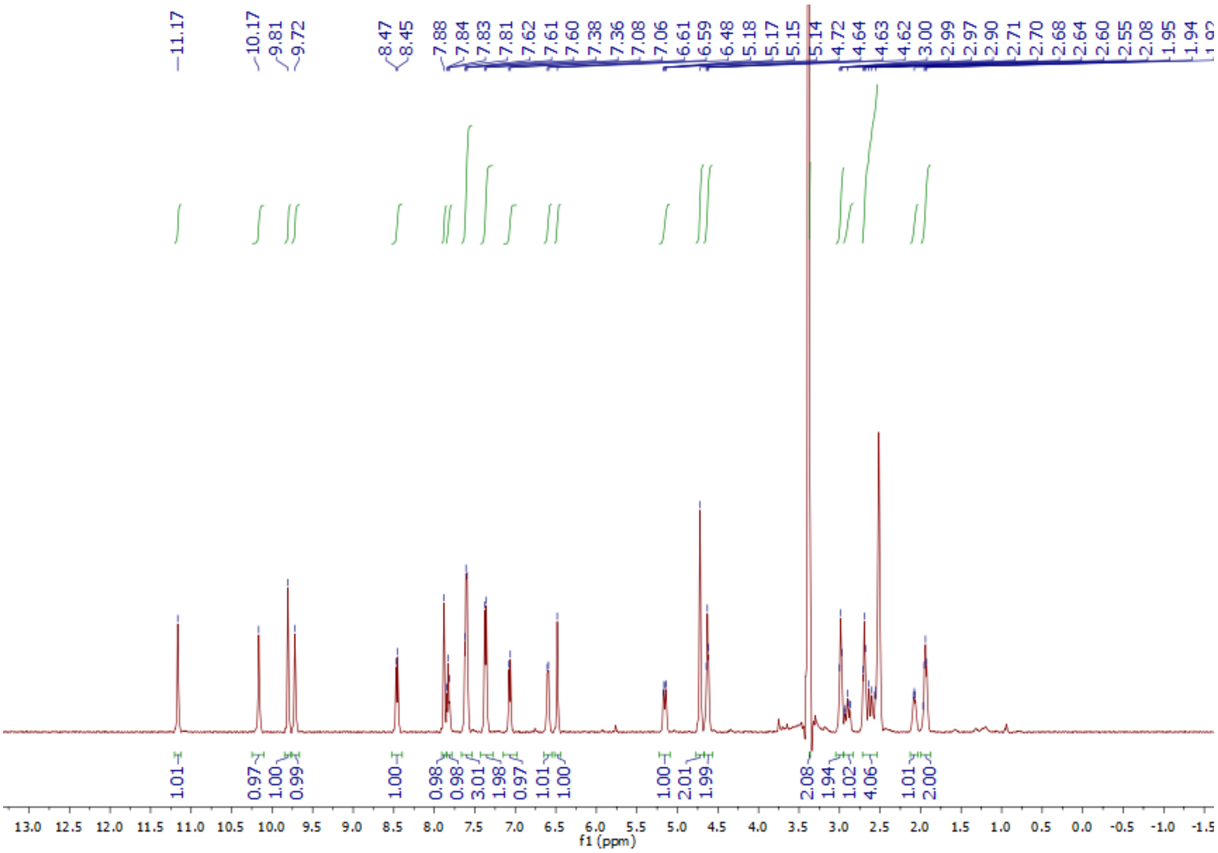
11.17
10.17
9.81
9.72
8.47
8.45
7.88
7.84
7.83
7.81
7.62
7.61
7.60
7.38
7.36
7.08
7.06
6.61
6.59
6.48
5.18
5.17
5.15
5.14
4.72
4.64
4.63
4.62
3.00
2.99
2.97
2.90
2.71
2.70
2.68
2.64
2.60
2.55
2.08
1.95
1.94
1.01
0.97
1.00
0.99
1.00
0.98
0.98
3.01
1.98
0.97
1.01
1.00
1.00
2.01
1.99
2.08
1.94
1.02
4.06
1.01
2.00
13.0 12.5 12.0 11.5 11.0 10.5 10.0 9.5 9.0 8.5 8.0 7.5 7.0 6.5 6.0 5.5 5.0 4.5 4.0 3.5 3.0 2.5 2.0 1.5 1.0 0.5 0.0 -0.5 -1.0
f1 (ppm)

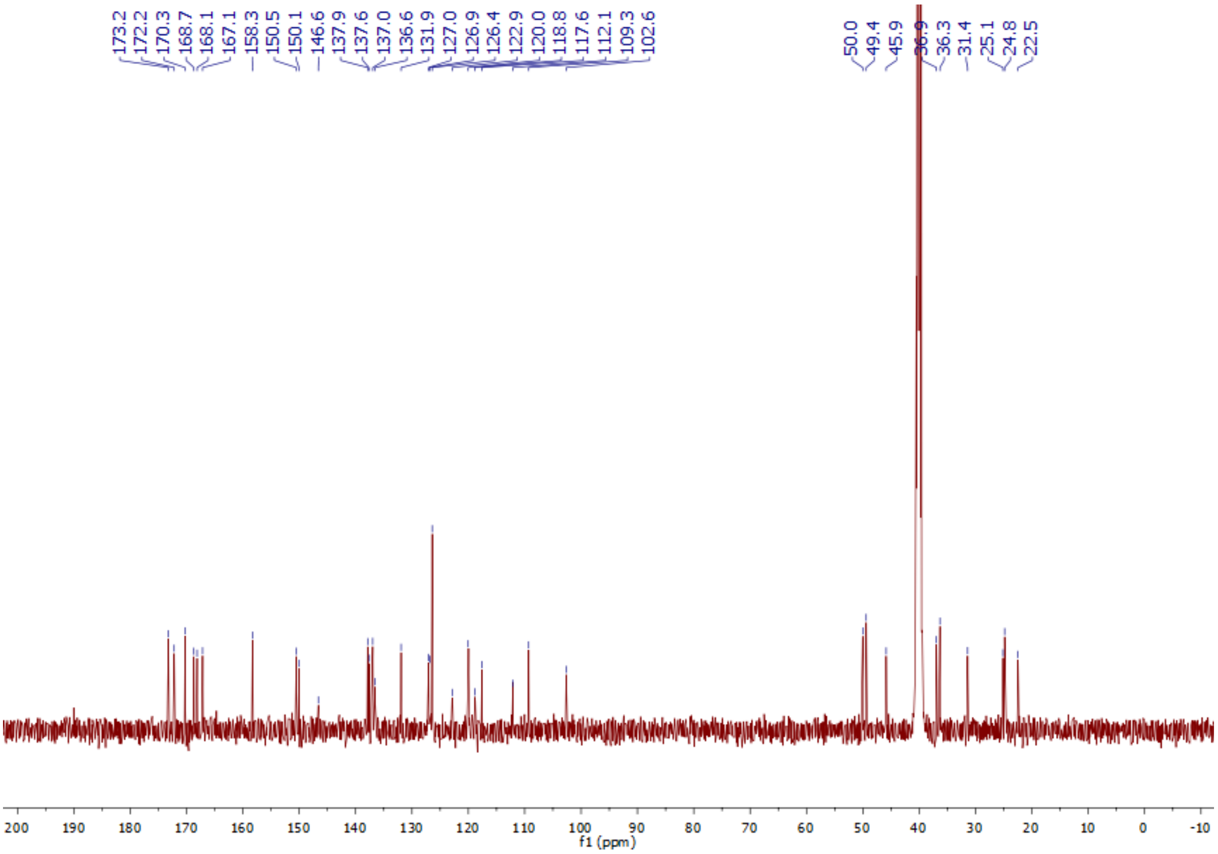
173.2
172.2
170.3
168.7
168.1
167.1
158.3
150.5
150.1
146.6
137.9
137.6
137.0
136.6
131.9
127.0
126.9
126.4
122.9
120.0
118.8
117.6
112.1
109.3
102.6
50.0
49.4
45.9
36.9
36.3
31.4
25.1
24.8
22.5
200 190 180 170 160 150 140 130 120 110 100 90 80 70 60 50 40 30 20 10 0 -10
f1 (ppm)

MD12

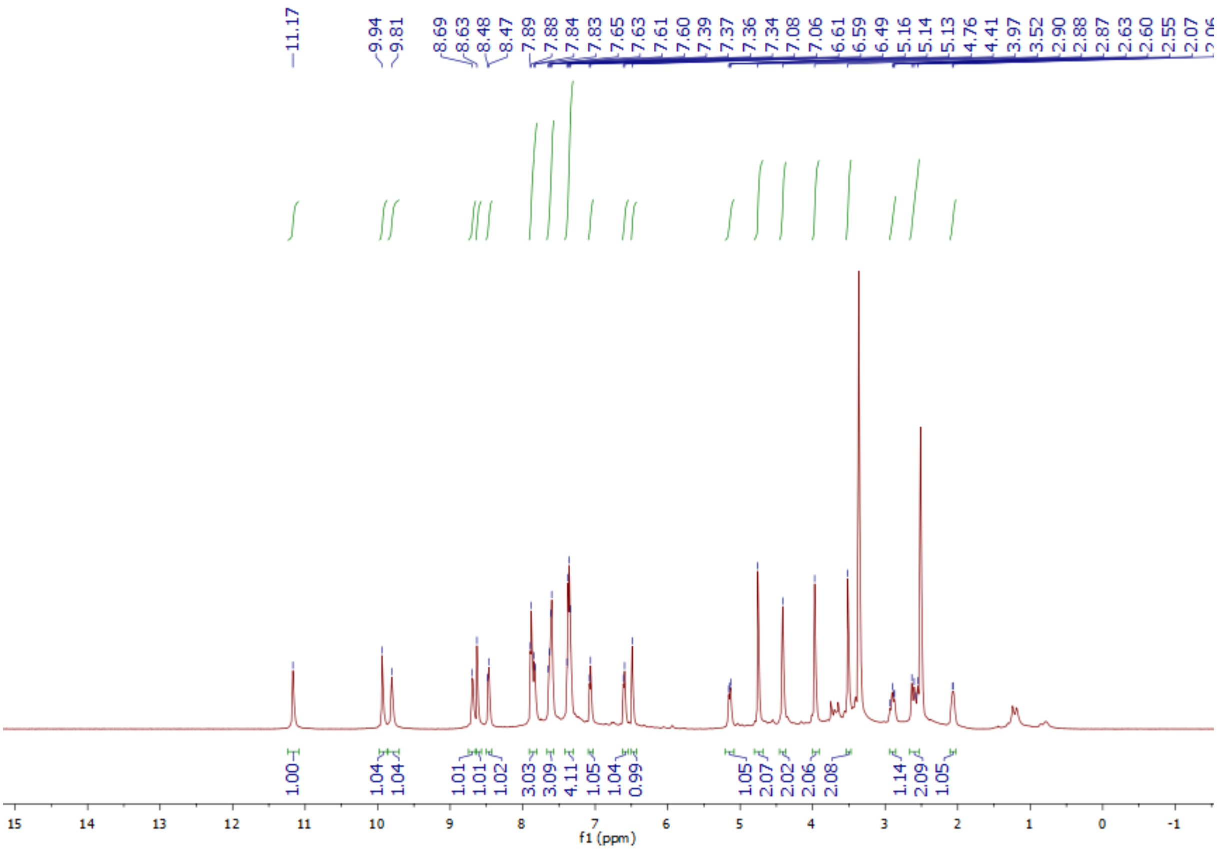

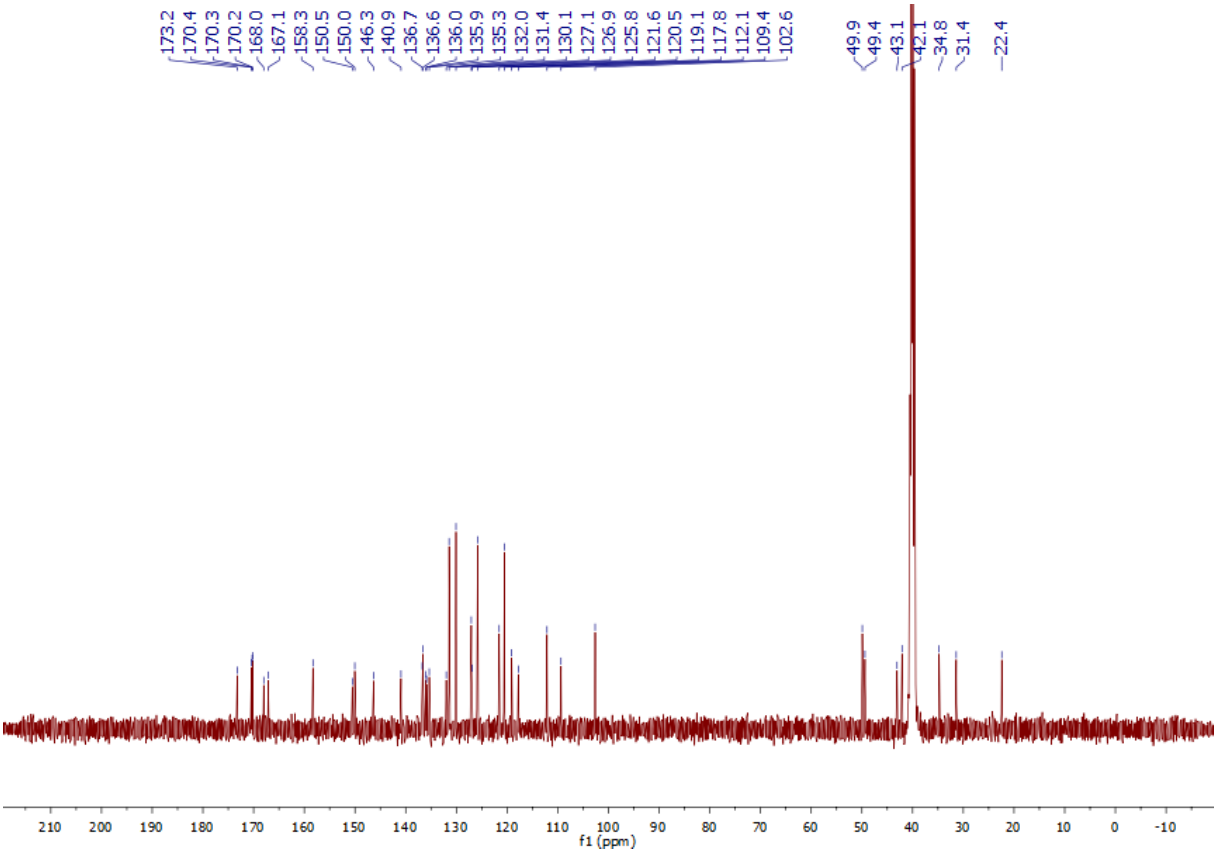

MD13

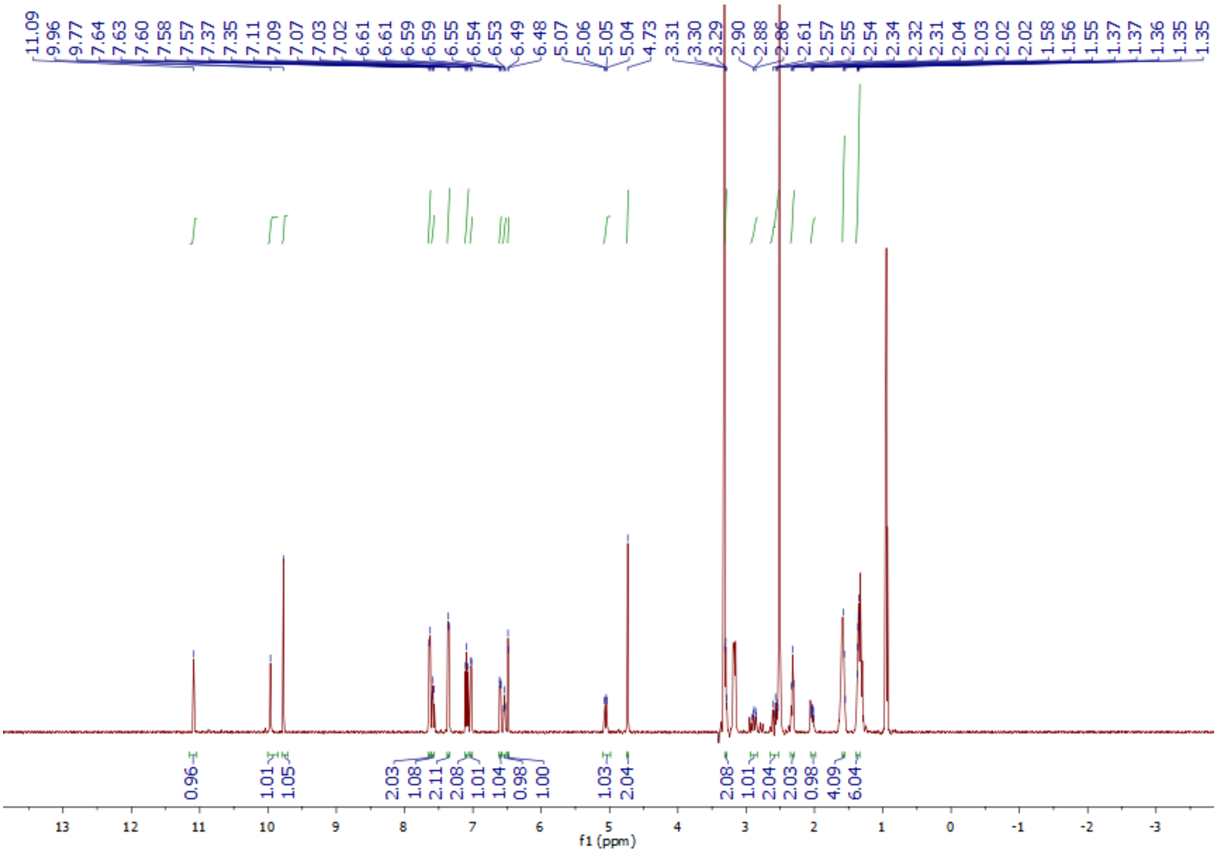

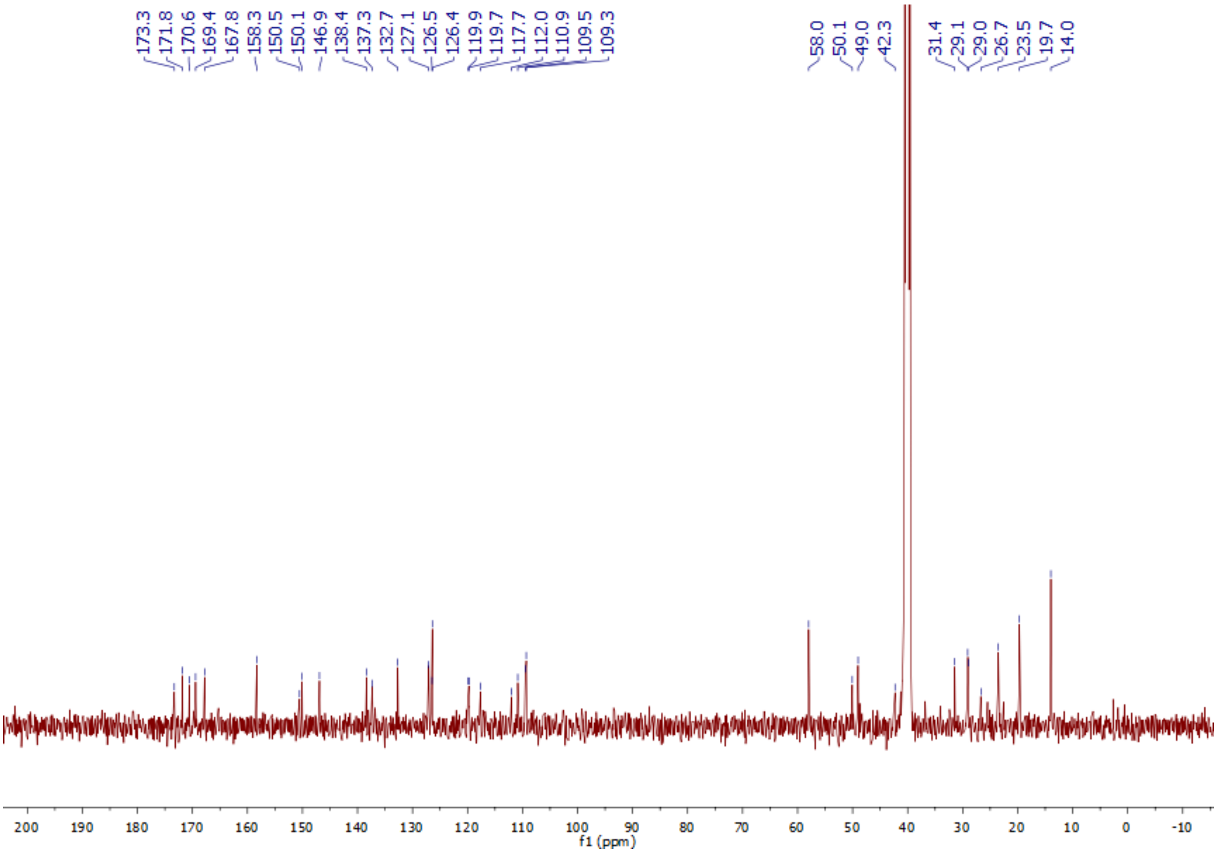

MD14

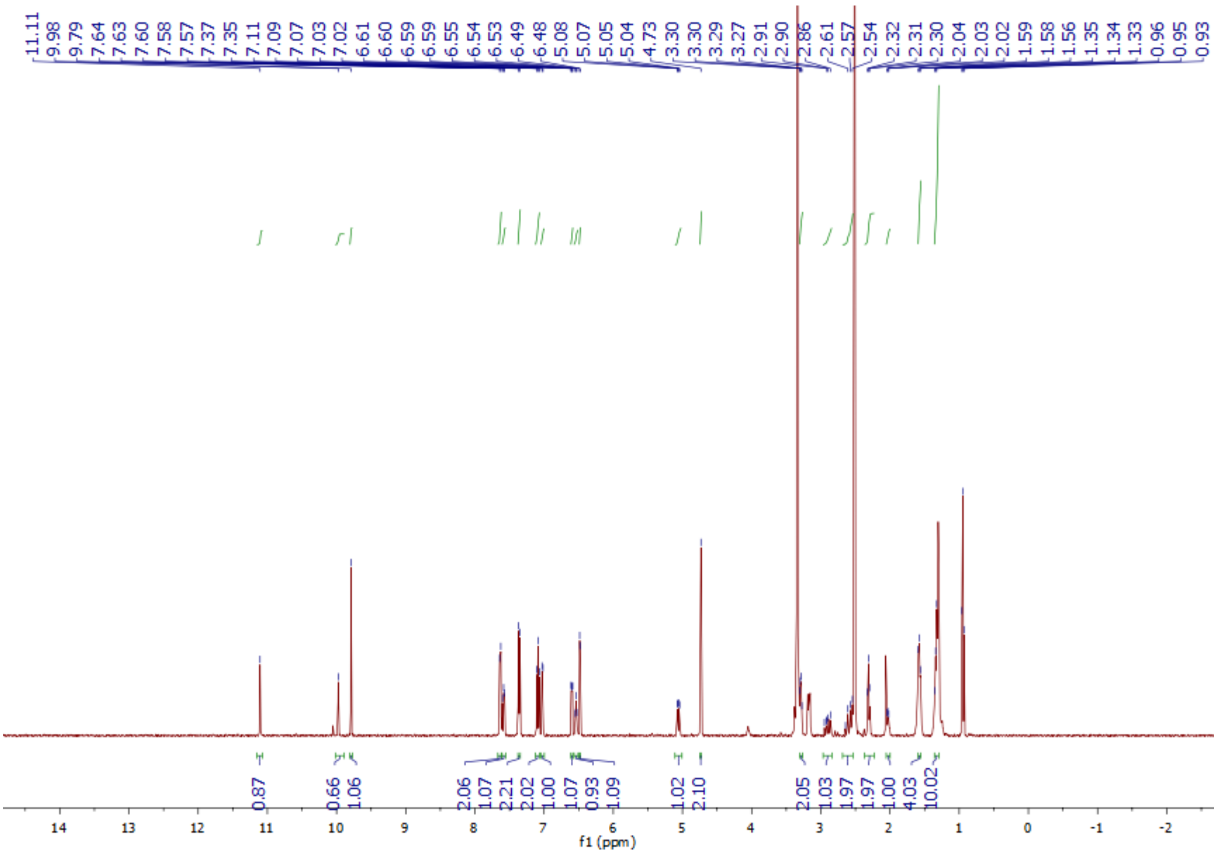
11.11 9.98 9.79 7.64 7.63 7.60 7.58 7.57 7.37 7.35 7.11 7.09 7.07 7.03 7.02 6.61 6.60 6.59 6.59 6.55 6.54 6.53 6.49 6.48 5.08 5.07 5.05 5.04 4.73 3.30 3.30 3.29 3.27 2.91 2.90 2.86 2.61 2.57 2.54 2.32 2.31 2.30 2.04 2.03 2.02 1.59 1.58 1.56 1.35 1.34 1.33 0.96 0.95 0.93
0.87 0.66 1.06 2.06 1.07 2.21 2.02 1.00 1.07 0.93 1.09 1.02 2.10 2.05 1.03 1.97 1.97 1.00 4.03 10.02
f1 (ppm)

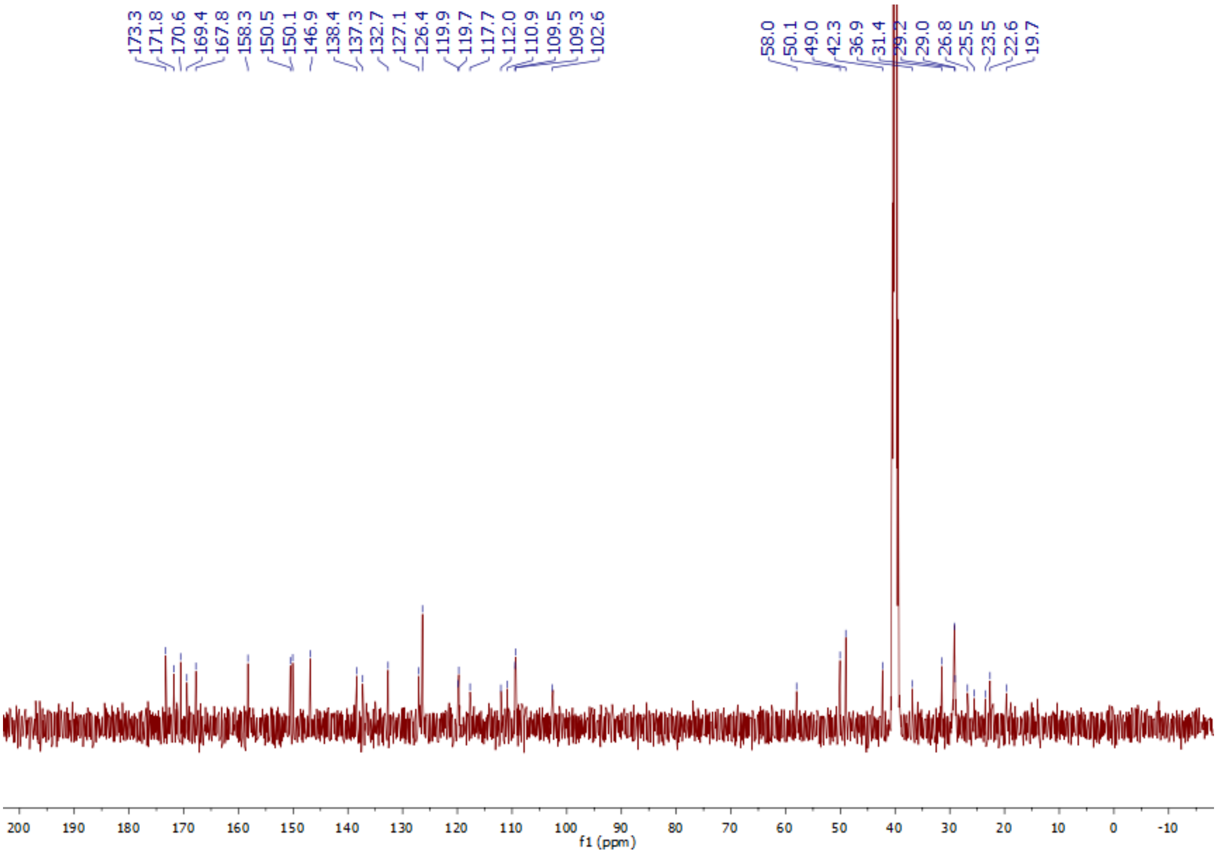
173.3 171.8 170.6 169.4 167.8 158.3 150.5 150.1 146.9 138.4 137.3 132.7 127.1 126.4 119.9 119.7 117.7 112.0 110.9 109.5 109.3 102.6
58.0 50.1 49.0 42.3 36.9 31.4 29.2 29.0 26.8 25.5 23.5 22.6 19.7
f1 (ppm)

MD15

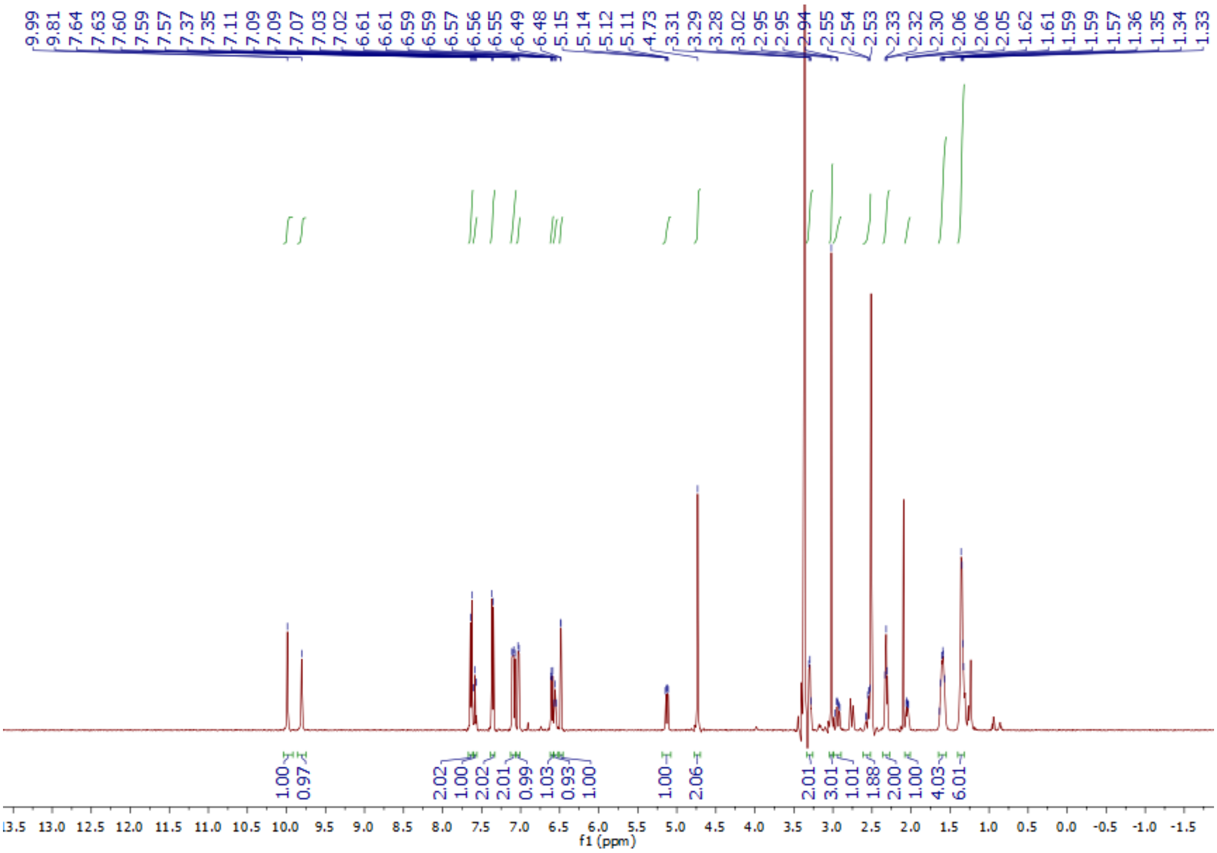
9.99
9.81
7.64
7.63
7.60
7.59
7.57
7.37
7.35
7.11
7.09
7.09
7.07
7.03
7.02
6.61
6.61
6.59
6.59
6.57
6.56
6.55
6.49
6.48
5.15
5.14
5.12
5.11
4.73
3.31
3.29
3.28
3.02
2.95
2.95
2.94
2.55
2.54
2.53
2.33
2.32
2.30
2.06
2.06
2.05
1.62
1.61
1.59
1.59
1.57
1.36
1.35
1.34
1.33
1.00
0.97
2.02
1.00
2.02
2.01
0.99
1.03
0.93
1.00
1.00
2.06
2.01
3.01
1.01
1.88
2.00
1.00
4.03
6.01
f1 (ppm)

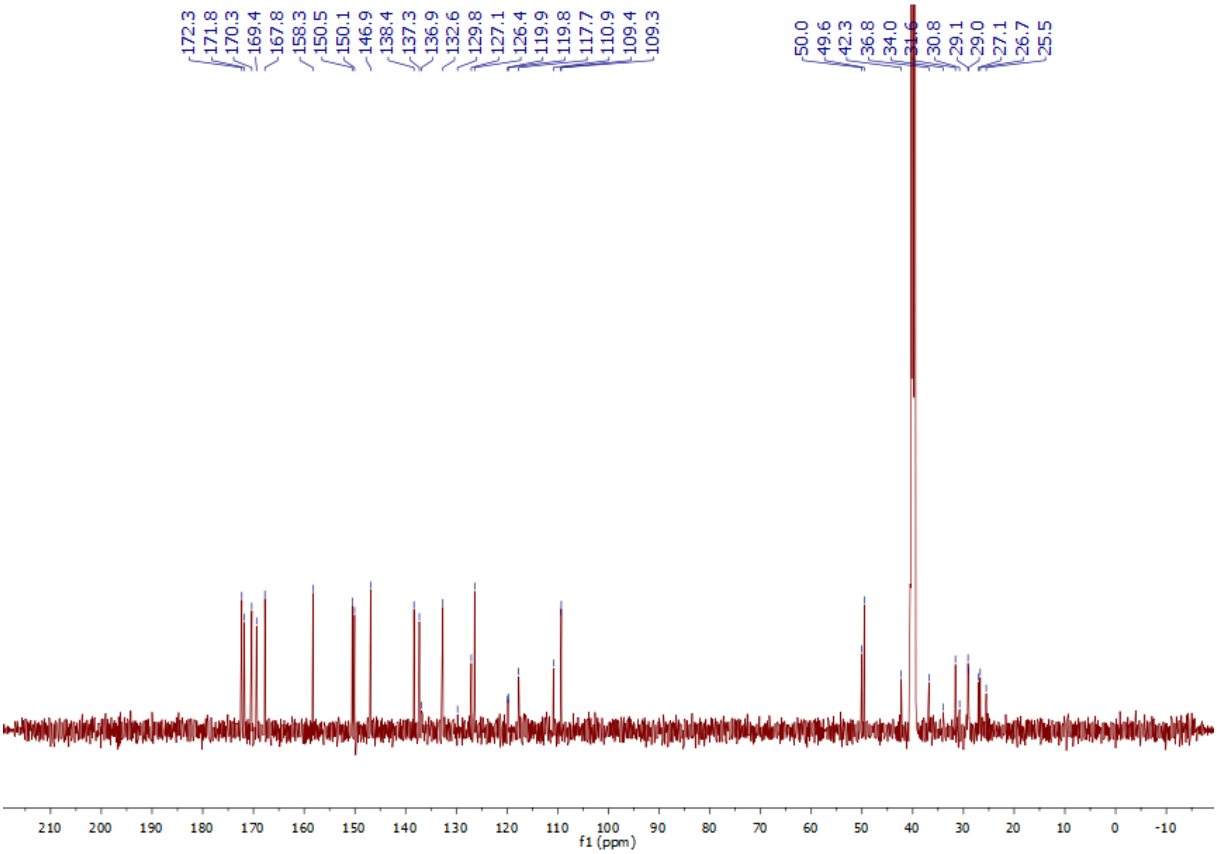
172.3
171.8
170.3
169.4
167.8
158.3
150.5
150.1
146.9
138.4
137.3
136.9
132.6
129.8
127.1
126.4
119.9
119.8
117.7
110.9
109.4
109.3
50.0
49.6
42.3
36.8
34.0
31.6
30.8
29.1
29.0
27.1
26.7
25.5
f1 (ppm)
